# Comparative effectiveness of nonpharmacological interventions in reducing psychological symptoms among patients with chronic low back pain

**DOI:** 10.1097/JS9.0000000000000798

**Published:** 2023-09-26

**Authors:** Lu-Ping Zhou, Ren-Jie Zhang, Jin Shang, Liang Kang, Zhi-Gang Zhang, Bo Zhang, Jia-Qi Wang, Chong-Yu Jia, Chen-Hao Zhao, Huang-Qing Zhang, Xian-Liang Zhang, Cai-Liang Shen

**Affiliations:** a Department of Orthopedics and Spine Surgery; b Laboratory of Spinal and Spinal Cord Injury Regeneration and Repair, The First Affiliated Hospital of Anhui Medical University; c Department of Radiology, the First Affiliated Hospital of University of Science and Technology of China, Hefei, Anhui, People’s Republic of China

**Keywords:** anxiety, chronic low back pain, depression, mental health, psychological symptom

## Abstract

**Objectives:**

Chronic low back pain (CLBP) can seriously impair the quality of life of patients and has a remarkable comorbidity with psychological symptoms, which, in turn, can further exacerbate the symptoms of CLBP. Psychological treatments are critical and nonnegligent for the management of CLBP, and thus, should attract sufficient attention. However, current evidence does not suggest the superiority and effectiveness of nonpharmacological interventions in reducing psychological symptoms among patients with CLBP.

Thus, this study was designed to compare the effectiveness of nonpharmacological interventions for depression, anxiety, and mental health among patients with CLBP and to recommend preferred strategies for attenuating psychological symptoms in clinical practice.

**Methods:**

In this systematic review and network meta-analysis (NMA), PubMed, Embase Database, Web of Science, and Cochrane Library were searched from database inception until March 2022. Randomized clinical trials (RCTs) that compare different nonpharmacological interventions for depression, anxiety, and mental health among patients with CLBP were eligible. The Preferred Reporting Items for Systematic Reviews and Meta-analyses statement was used. Four reviewers in pairs and divided into two groups independently performed literature selection, data extraction, and risk of bias, and certainty of evidence assessments. This NMA was conducted with a random effects model under a frequentist framework. The major outcomes were depression, anxiety, and mental health presented as the standardized mean difference (SMD) with the corresponding 95% CI.

**Results:**

A total of 66 RCTs that randomized 4806 patients with CLBP met the inclusion criteria. The quality of evidence was typically low or some risks of bias (47 out of 66 trials, 71.3%), and the precision of summary estimates for effectiveness varied substantially. In addition, 7 categories of interventions with 26 specific treatments were evaluated. For depression, mind body therapy (pooled SMD = −1.20, 95% CI: −1.63 to −0.78), biopsychosocial approach (pooled SMD = −0.41, 95% CI: −0.70 to −0.12), and physical therapy (pooled SMD = −0.26, 95% CI: −0.50 to −0.02) exhibited remarkable effectiveness in reducing depression compared with the control group. For managing anxiety, mind body therapy (pooled SMD = −1.35, 95% CI: −1.90 to −0.80), multicomponent intervention (pooled SMD = −0.47, 95% CI: −0.88 to −0.06), and a biopsychosocial approach (pooled SMD = −0.46, 95% CI: −0.79 to −0.14) were substantially superior to the control group. For improving mental health, multicomponent intervention (pooled SMD = 0.77, 95% CI: 0.14 to 1.39), exercise (pooled SMD = 0.60, 95% CI: 0.08 to 1.11), and physical therapy (pooled SMD = 0.47, 95% CI: 0.02–0.92) demonstrated statistically substantial effectiveness compared with the control group. The rank probability indicated that mind body therapy achieved the highest effectiveness in reducing depression and anxiety among patients with CLBP. Besides, the combined results should be interpreted cautiously based on the results of analyses evaluating the inconsistency and certainty of the evidence.

**Conclusion:**

This systemic review and NMA suggested that nonpharmacological interventions show promise for reducing psychological symptoms among patients with CLBP. In particular, mind body therapy and a biopsychosocial approach show considerable promise, and mind body therapy can be considered a priority choice in reducing depression and anxiety. These findings can aid clinicians in assessing the potential risks and benefits of available treatments for CLBP comorbidity with psychological symptoms and provide evidence for selecting interventions in clinical practice. More RCTs involving different interventions with rigorous methodology and an adequate sample size should be conducted in future research.

## Introduction

Highlights

- Chronic low back pain (CLBP) can seriously impair the quality of life of patients and has a remarkable comorbidity with psychological symptoms, which, in turn, can further exacerbate the symptoms of CLBP.
- Current evidence does not suggest the effectiveness and superiority of nonpharmacological interventions in reducing psychological symptoms among patients with CLBP.
- Nonpharmacological interventions are effective in reducing psychological symptoms among patients with CLBP.
- Specifically, mind body therapy and psychological therapy appear to be remarkably effective in reducing psychological symptoms among patients with CLBP, and mind body therapy can be considered a priority choice in reducing depression and anxiety.
- These findings can aid clinicians in assessing the potential risks and benefits of available treatments for CLBP comorbidity with psychological symptoms and provide evidence for selecting interventions in clinical practice.

Chronic low back pain (CLBP) is one of the major public health problems worldwide, conferring considerable discomfort, disability, medical cost, and economic burden^1,2^. In 2019, low back pain (LBP) was the most prevalent musculoskeletal disorder among adolescents and young adults globally, accounting for 76.4% of the incident cases and 45.2% of the prevalent cases in 204 countries and territories^3^. CLBP can seriously decrease quality of life and has a remarkable comorbidity with mental health; hence, it not only restricts an individual’s ability for physical activities and daily tasks, but also impairs his/her psychological well-being, causing higher levels of subclinical depression and anxiety^4,5^. Emerging evidence suggests that existing psychological disturbances, in turn, can exaggerate the symptomatology of CLBP; meanwhile, the reduction of depression and anxiety, and improvement in mental health, can help patients manage and control CLBP. Accordingly, psychological treatments are critical and nonnegligent for managing CLBP, and thus, should attract sufficient attention.

Nonpharmacological interventions have been primarily recommended for managing CLBP. Some categories of interventions, such as exercise, mind body therapy, education, telemedicine, biopsychosocial approach, physical therapy, and multicomponent intervention, have been proposed and demonstrated satisfactory clinical effectiveness. However, the existing literature has focused on the effectiveness of specific treatments for LBP relief, while evidence for psychological outcomes remains relatively limited. Furthermore, the meta-analysis conducted by Anheyer *et al*.^6^ reported that yoga, which is one form of mind body therapy, provided short-term improvement in mental health compared with passive control. By contrast, the meta-analysis performed by Wieland *et al*.^7^ found little to no difference in depression with yoga compared with nonexercise at 3 months, but medium improvement in depression at 12 months. Wen *et al*.^8^ compared mind body therapy with a nonactive and an active control groups in a meta-analysis and found no difference in the mental component summary at the final follow-up. Thus, the effectiveness of these interventions in reducing psychological symptoms, including the depression, anxiety, and other mental disorders, remains controversial. Moreover, although pairwise meta-analyses regarding different types of interventions, such as mind body therapy^6–8^, exercise^9^, biopsychosocial approach^10^, and physical therapy^11^, have been conducted to determine their effectiveness in reducing the psychological symptoms of patients with LBP, the superiority of different interventions in psychotherapy remains unclear. Meanwhile, no network meta-analysis (NMA) has been conducted on this topic thus far.

Psychological symptoms refer to the abnormal manifestations in emotions, thoughts, behaviors, and social interactions^12^. Among the psychological symptoms, depression and anxiety were the most extensively studied emotional problems during the treatment of LBP. Therefore, the primary objective of the current systematic review and NMA was to demonstrate the comparative effectiveness of nonpharmacological interventions for depression and anxiety among patients with CLBP. Besides, the data on mental health, which serves as the secondary outcome, were also extracted from the mental component of the Short Form (SF-12 or SF-36), aiming to provide further insight into the effectiveness of nonpharmacological interventions for mental disorders and recommend the preferred strategies for attenuating psychological symptoms in clinical practice.

## Methods

This NMA was performed by following the Preferred Reporting Items for Systematic Reviews (PRISMA) criteria^13^ (Supplemental Digital Content 1, http://links.lww.com/JS9/B119) (Supplemental Digital Content 2, http://links.lww.com/JS9/B120) and PRISMA-NMA statement^14^, (Supplemental Digital Content 3, http://links.lww.com/JS9/B121) and Assessing the Methodological Quality of Systematic Reviews 2 (AMSTAR 2) guidelines^15^, (Supplemental Digital Content 4, http://links.lww.com/JS9/B122). The protocol of this study was registered in PROSPERO.

### Search strategy

We conducted a systematic and comprehensive search for eligible studies from the following databases: PubMed, Embase, Web of Science, and Cochrane Library (from inception until 4 May 2023). The detailed search strategies and terms searched in the databases are provided in Appendix 1 (Supplemental Digital Content 5, http://links.lww.com/JS9/B123). During study selection, four reviewers in pairs and divided into two groups independently conducted the literature search. They screened the titles, abstracts, and relevant full texts, and then assessed the eligibility of the studies. Furthermore, a manual review of reference lists and other related studies was conducted to identity additional eligible literature that met the inclusion criteria. Any discrepancies were initially addressed through a consensus meeting, and another investigator was consulted if necessary.

### Eligibility criteria

The Population, Intervention, Comparison, Outcomes, and Study (PICOS) principle was adopted to identify potentially relevant randomized clinical trials (RCTs). (1) Population: Patients who suffered from CLBP (pain in the area between the lower borders of the ribcage and the creases of the buttocks persisting for more than 3 months)^16^. Specific CLBP attributed to a history of spinal surgery or an underlying pathology, such as fractures or malignancies, was excluded. (2) Intervention: All nonpharmacological treatments for CLBP were eligible. Studies that evaluated surgical treatments were excluded. (3) Comparison: Comparators that were considered eligible consisted of active controls, usual care, or other nonpharmacological interventions, including standard/routine care, no treatment, placebo, wait-list control, sham treatment, low-intensity regular exercise programs, and regular programmed health education. (4) Outcomes: Studies that were eligible for inclusion provided data on at least one of the following outcomes: depression, anxiety, or overall mental health. Besides, the data of mental health were extracted from the mental component of the short form SF -12 or SF-36 in the included RCTs. These outcomes were presented in the form of mean and SD, or in the format that these values could be derived from. (5) Study design: Only RCTs were regarded as eligible, whereas comparative cohort studies, case reports, reviews, letters, and conference reports were excluded. No limitations were set on age, sex, or publication year.

### Data extraction

The data of RCT characteristics (author, publication year, country/region, intervention and comparator, sample size, and follow-up duration), participant characteristics (age, BMI, and sex), and outcome measures (depression, anxiety, and mental health) from eligible RCTs were extracted independently by four reviewers in pairs and divided into two groups. Any disagreement was resolved by a fifth reviewer. For different follow-up periods or multiple publications reporting data from the same RCT, the one with the most extended follow-up period was selected. Moreover, the time end point of study follow-up and the criteria for assessing outcome results were extracted.

### Risk of bias and certainty of evidence assessments

The assessment of the risk of bias for the included RCTs was performed using the revised Cochrane risk of bias tool for randomized trials (RoB 2) including the items of randomization process, deviation from intended interventions, missing outcome data, measurement of the outcome, and selection of the reported result^17^. The certainty of evidence was evaluated on the basis of the grading of recommendation assessment, development, and evaluation (GRADE) approach for NMA^18–21^. The assessment of the certainty of mixed or indirect evidence included the within-study bias, reporting bias, indirectness, imprecision, heterogeneity, and incoherence. Four reviewers in pairs were divided into two groups to rate the risk of bias, and discrepancies were resolved through discussion. A fifth investigator was invited to adjudicate if necessary.

### Statistical analysis

#### Data analysis

The continuous outcomes were pooled by the standardized mean difference (SMD) with the corresponding 95% CI for the outcomes of depression, anxiety, and mental health.

Data were pooled when studies explored similar therapies with identical objectives or the same working mechanism. For example, acceptance and commitment therapy and behavioral activation therapy are two forms of cognitive behavioral therapy (CBT). Furthermore, the methods outlined in the *Cochrane Handbook* were used to estimate SDs from standard errors (SEs), *P*-values, or 95% CIs^22^.

#### Pairwise meta-analysis

Pairwise meta-analyses were conducted using Revman version 5.4.1 software (Cochrane Collaborating). The *I*^2^ test was used for assessing heterogeneity. *I*^2^ less than 50% was considered no evidence of heterogeneity, and a fixed effects model was chosen. Otherwise, a random effects model was employed.

#### NMA

NMAs were performed using a frequentist framework of the multivariate random effects meta-analysis model with the ʻmvmetaʼ and ʻnetworkʼ commands in Stata SE 15.0^23–25^. The inconsistency of NMAs was assessed in terms of global, local, and loop inconsistencies. Global inconsistency was evaluated using the design-by-treatment interaction model; local inconsistency was evaluated using the node-splitting approach; loop inconsistency was assessed using the loop-specific method within each closed triangular loop in the network^26–28^. A *P*-value above 0.05 suggested no significant inconsistency between indirect and direct comparisons, and the consistency model will be preferred for comparing all interventions. Moreover, the evaluation of the transitivity assumption was based on comparing the distribution or frequency of potential effect modifiers, including the distribution of included RCTs and patient characteristics across treatment comparisons. Moreover, league tables with relative treatment effect sizes were utilized to display the comparisons of network estimations. The effectiveness of nonpharmacological interventions for each outcome was computed and ranked with the surface under the cumulative ranking curve (SUCRA) and mean ranks^29^.

#### Subgroup and sensitivity analyses

Subgroup and sensitivity analyses were conducted to explore the sources of heterogeneity and the robustness of the outcomes. Subgroup analyses were performed to investigate the effectiveness of specific types of treatment on the psychological symptoms (depression, anxiety, and mental health) of patients with CLBP. For specific interventions, pairwise meta-analysis and NMA were also performed. Moreover, NMA for subgroup analyses included only treatments that were reported in a minimum of two studies. In addition, sensitivity analyses were performed by including RCTs with a low overall risk of bias.

#### Publication Bias

If more than six RCTs were included for a pairwise meta-analysis, then the existence of publication bias was assessed by employing Egger’s tests via the ʻmetabiasʼ command in Stata SE 15.0^30,31^. For NMA, comparison-adjusted funnel plots were applied to evaluate publication bias via the ʻnetfunnelʼ command in Stata SE 15.0^32^.

## Results

### Study search

**Figure 1:**
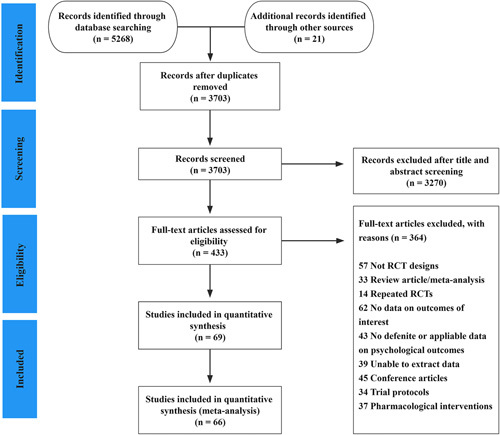
PRISMA-NMA flow diagram for literature search and selection.

The detailed process for the literature selection is illustrated in Figure 1. We identified 5268 potential records from all the databases and 21 additional studies were examined through the reference lists. After the removal of duplicates, 3703 records were screened, undergoing title and abstract evaluation. Finally, 66 RCTs^33–98^ were deemed eligible for the current study.

### Characteristics of the included studies

The summarized characteristics of the included RCTs and patients are provided in Appendix 2 (Supplemental Digital Content 5, http://links.lww.com/JS9/B123). The included 66 RCTs involving 5388 recruited participants were eligible for inclusion in the current study. Considering lost during follow-up, the remaining 4806 participants who finished the final follow-up estimation were analyzed for this meta-analysis. The included studies were published between 1993 and 2023. From 2008 to 2023, a continual increase was recorded in the total number of included publications, with the largest proportion published from 2017 to 2023 (*n*=41, 62.1%). In addition, the NMA included 52 two-arm RCTs, 12 three-arm RCTs, and two four-arm RCTs. The studies included in this NMA employed a parallel RCT design. Moreover, the largest proportion of RCTs were conducted in Europe (*n*=25, 37.9%), followed by Asia (*n*=24, 36.4%), North America (*n*=10, 15.2%), South America (*n*=3, 4.5%), Africa (*n*=2, 3.0%), and Oceania (*n*=2, 3.0%). The sample size of the included RCTs for the final follow-up ranged from 16 to 444, with the majority of RCTs less than or equal to 100 (*n*=54, 81.8%), 11 (16.7%) ranging from 101 to 400, and 1 (1.5%) greater than 400.

### Interventions

Seven categories of interventions were identified to reduce psychological symptoms in patients with CLBP: exercise, mind body therapy, education, telemedicine, biopsychosocial approach, physical therapy, and multicomponent intervention. Moreover, the aforementioned 7 categories of interventions can be classified into 26 specific treatments. Therefore, we conducted subgroup analyses to explore the effectiveness of these specific treatments further. The summary of the seven categories of interventions and 26 specific treatments is found in Appendix 3 (Supplemental Digital Content 5, http://links.lww.com/JS9/B123).

### Risk of bias and certainty of evidence

**Figure 2:**
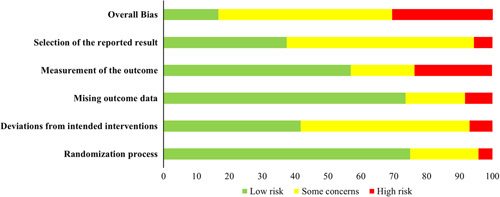
Summary results on risk of bias (using RoB 2) of including RCTs.

Among the included RCTs, 10 (15.2%) studies^42,43,47,49,54,57,67,81,88,98^ had a low overall risk of bias, 37 (56.1%)^33,35,36,38–40,44,45,48,50–52,59–61,63–66,68–71,73,75–78,84–87,91–94,97^ exhibited some concerns, and 19 (28.7%)^34,37,41,46,53,55,56,58,62,72,74,79,80,82,83,89,90,95,96^ had a high overall risk of bias. The majority of RCTs presented low risks of missing outcome data (72.7%) and randomization (75.8%). The largest proportion of high risk arose from the measurement of outcomes (21.2%), followed by missing outcome data (9.1%, Fig. 2). The outcomes of risk of bias and certainty of evidence are provided in Appendices 4–14 (Supplemental Digital Content 5, http://links.lww.com/JS9/B123).

### Nonpharmacological interventions for psychological symptoms

#### Depression

**Figure 3:**
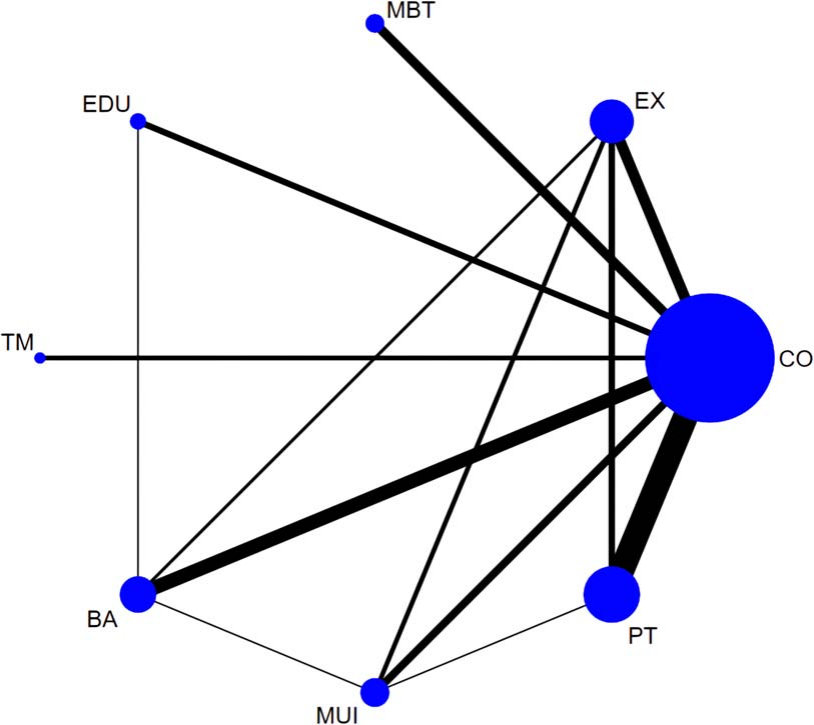
Network plot of comparisons in the network meta-analysis of different categories of interventions for depression. Nodes’ sizes and line widths represent the number of randomized patients and controlled trials for each treatment, respectively. The size of the node corresponds to the number of patients randomized to each treatment, whereas the line width indicates the number of randomized controlled trials comparing each pair of treatments. BA, biopsychosocial approach; CO, control; EX, exercise; EDU, education; MBT, mind body therapy; MUI, multicomponent intervention; PT, physical therapy; TM, telemedicine. Interventions details are described in Appendix 3 (Supplemental Digital Content 5, http://links.lww.com/JS9/B123).

A total of 50 RCTs^33–36,38,40,41,43–46,48,49,51–54,56–62,64,65,67–70,72,74–76,78–84,88–91,93–95,97,98^ involving 3733 participants and 7 categories of interventions were included in the NMA for depression with eight loops (Fig. 3). The pooled results of the NMA indicated that mind body therapy (pooled SMD = −1.20, 95% CI: −1.63 to −0.78), biopsychosocial approach (pooled SMD = −0.41, 95% CI: −0.70 to −0.12), and physical therapy (pooled SMD = −0.26, 95% CI: −0.50 to −0.02) exhibited remarkable effectiveness in reducing depression compared with the control group. In accordance with SUCRA, mind body therapy (100.0%) had the highest probability to be the most effective intervention in reducing depression, followed by the biopsychosocial approach (73.2%), physical therapy (54.0%), multicomponent intervention (51.0%), exercise (46.1%), education (45.3%), and telemedicine (16.9%). In terms of subgroup analyses based on specific treatments, 47 RCTs with 3455 participants and 15 specific treatments were included in the NMA for depression. The pooled outcomes indicated that mind body therapy was associated with a decrease in depression compared with CBT (pooled SMD = −0.77, 95% CI: −1.39 to −0.15), psychosocial intervention (PI, pooled SMD = −0.82, 95% CI: −1.48 to −0.16), active control (pooled SMD = −1.22, 95% CI: −1.72 to −0.72), and usual care (pooled SMD = −1.22, 95% CI: −1.74 to −0.71). The SUCRA ranking indicated that mind body therapy (98.2%) and massage (81.4%) had the highest ranking probabilities, and kinesiology taping (KT, 24.8%) and telemedicine (24.7%) had the lowest ranking probabilities.

The pooled outcomes of the pairwise meta-analyses showed that mind body therapy (pooled SMD = −1.28, 95% CI: −2.11 to −0.44), biopsychosocial approach (pooled SMD = −0.42, 95% CI: −0.73 to −0.12), physical therapy (pooled SMD = −0.25, 95% CI: −0.39 to −0.10), and exercise (pooled SMD = −0.21, 95% CI: −0.40 to −0.01) were associated with remarkable effectiveness in reducing depression compared with the control group. For specific treatment, mind body therapy (pooled SMD = −1.03, 95% CI: −1.68 to −0.37) and CBT (pooled SMD = −0.82, 95% CI: −1.59 to −0.05) demonstrated remarkable effectiveness in reducing depression compared with usual care. PI (pooled SMD = −0.43, 95% CI: −0.82 to −0.05) for depression management was superior to the active control. The detailed outcomes of the pairwise meta-analysis and NMA for depression are provided in Appendices 15–19 (Supplemental Digital Content 5, http://links.lww.com/JS9/B123).

#### Anxiety

**Figure 4:**
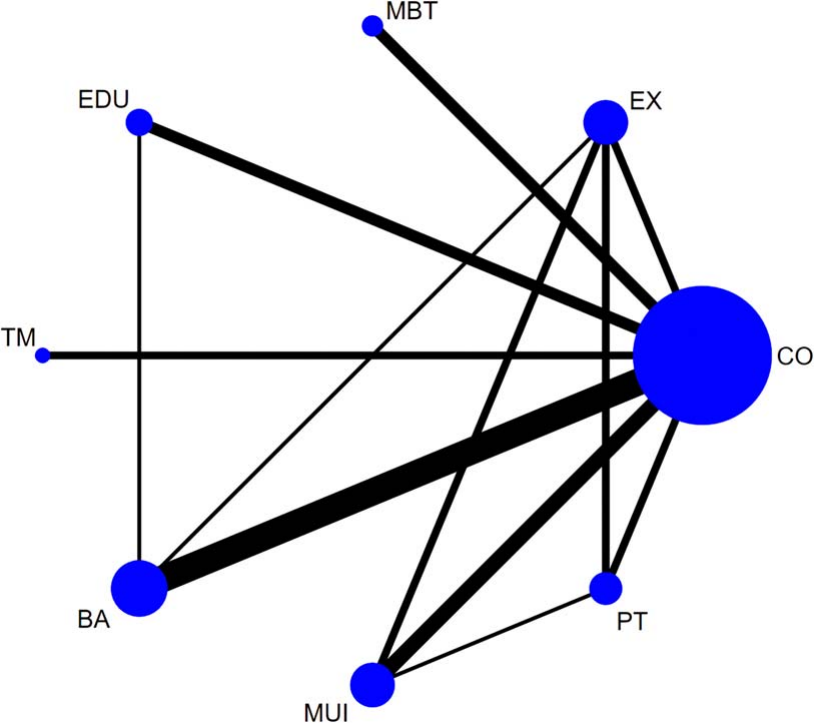
Network plot of comparisons in the network meta-analysis of different categories of interventions for anxiety. Nodes’ sizes and line widths represent the number of randomized patients and controlled trials for each treatment, respectively. The size of the node corresponds to the number of patients randomized to each treatment, whereas the line width indicates the number of randomized controlled trials comparing each pair of treatments. BA, biopsychosocial approach; CO, control; EX, exercise; EDU, education; MBT, mind body therapy; MUI, multicomponent intervention; PT, physical therapy; TM, telemedicine. Interventions details are described in Appendix 3 (Supplemental Digital Content 5, http://links.lww.com/JS9/B123).

In the NMA involving 24 RCTs^34–38,43,45,49,51,52,54,59,68–70,72,75–79,84,88,97^, 1722 participants, and 7 categories of interventions with six loops (Fig. 4), mind body therapy (pooled SMD = −1.35, 95% CI: −1.90 to −0.80), multicomponent intervention (pooled SMD = −0.47, 95% CI: −0.88 to −0.06), and biopsychosocial approach (pooled SMD = −0.46, 95% CI: −0.79 to −0.14) were superior to the control group in managing anxiety. The SUCRA ranking suggested that mind body therapy (99.8%) was the optimal intervention for anxiety management, followed by multicomponent intervention (68.8%), biopsychosocial approach (68.6%), education (64.6%), telemedicine (30.0%), and physical therapy (17.9%). For the subgroup analyses of specific treatments with 24 RCTs, 1713 participants, and 10 specific methods, mind body therapy exhibited remarkable effectiveness in reducing anxiety compared with usual care (pooled SMD = −1.52, 95% CI: −2.14 to −0.90) and active control (pooled SMD = −1.30, 95% CI: −1.83 to −0.76). Moreover, CBT (pooled SMD = −0.62, 95% CI: −1.06 to −0.17), PI (pooled SMD = −0.57, 95% CI: −1.06 to −0.08), multicomponent intervention (pooled SMD = −0.52, 95% CI: −0.94 to −0.10), and education (pooled SMD = −0.51, 95% CI: −0.95 to −0.06) were superior to usual care in anxiety reduction. SUCRA ranking indicated that mind body therapy (99.8%), CBT (73.5%), and PI (69.8%) were the top three recommended treatments for anxiety.

The pooled results of the pairwise meta-analyses reported that mind body therapy (pooled SMD = −1.34, 95% CI: −1.66 to −1.02), multicomponent intervention (pooled SMD = −0.41, 95% CI: −0.63 to −0.19), biopsychosocial approach (pooled SMD = −0.47, 95% CI: −0.88 to −0.06), and education (pooled SMD = −0.55, 95% CI: −1.04 to −0.06) were superior to the control group. For specific treatments, mind body therapy (pooled SMD = −1.34, 95% CI: −1.69 to −0.99) was superior to active control, and multicomponent intervention (pooled SMD = −0.51, 95% CI: −0.78 to −0.24) was superior to usual care. The detailed outcomes of the pairwise meta-analysis and NMA for anxiety are provided in Appendices 20–24 (Supplemental Digital Content 5, http://links.lww.com/JS9/B123).

#### Mental health

**Figure 5:**
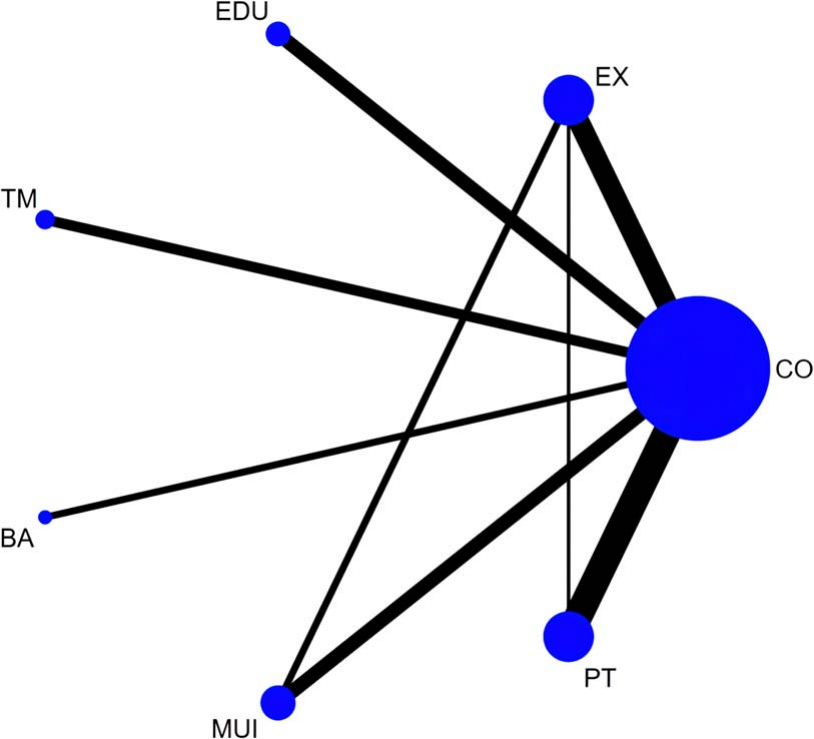
Network plot of comparisons in the network meta-analysis of different categories of interventions for mental health. Nodes’ sizes and line widths represent the number of randomized patients and controlled trials for each treatment, respectively. The size of the node corresponds to the number of patients randomized to each treatment, whereas the line width indicates the number of randomized controlled trials comparing each pair of treatments. BA, biopsychosocial approach; CO, control; EX, exercise; EDU, education; MBT, mind body therapy; MUI, multicomponent intervention; PT, physical therapy; TM, telemedicine. Interventions details are described in Appendix 3 (Supplemental Digital Content 5, http://links.lww.com/JS9/B123).

A total of RCTs^42,45–47,51,53–56,58,60,63,65–67,70–72,78,86,87,92,96,97^ involving 2061 participants and 6 categories of interventions provided data for the intervention of mental health with two loops (Fig. 5). Compared with the control group, multicomponent intervention (pooled SMD = 0.77, 95% CI: 0.14 to 1.39), exercise (pooled SMD = 0.60, 95% CI: 0.08 to 1.11), and physical therapy (pooled SMD = 0.47, 95% CI: 0.02–0.92) demonstrated statistically substantial effectiveness on improvement in mental health. In accordance with SUCRA, multicomponent intervention (79.8%) had the highest probability to be the most effective intervention for mental health, followed by exercise (66.3%), physical therapy (54.1%), biopsychosocial approach (50.6%), telemedicine (49.9%), and education (40.6%). For the subgroup analyses involving 16 RCTs, 1397 participants, and 5 specific treatments, multicomponent intervention (pooled SMD = 1.20, 95% CI: 0.19–2.22) and aerobic training (AT, pooled SMD = 0.97, 95% CI: 0.25–1.69) were superior to active control. The SUCRA ranking showed that multicomponent intervention (83.0%), AT (67.5%), and meditation (66.4%) were the top three recommended treatments for mental health.

The pooled outcomes of the pairwise meta-analyses demonstrated that multicomponent intervention (pooled SMD = 0.60, 95% CI: 0.01–1.19) and physical therapy (pooled SMD = 0.47, 95% CI: 0.11–0.83) were superior to the control group for improvement in mental health. For the specific treatments, multicomponent intervention (pooled SMD = 0.60, 95% CI: 0.01–1.19) exhibited substantial effectiveness on mental health compared with usual care. Meanwhile, AT (pooled SMD = 1.03, 95% CI: 0.03–2.02) was superior to active control for improvement in mental health. The detailed outcomes of the pairwise meta-analysis and NMA for mental health are provided in Appendices 25–29 (Supplemental Digital Content 5, http://links.lww.com/JS9/B123).

#### Inconsistency analyses, sensitivity analyses, and publication bias

No serious inconsistency was found in the NMA. Global and local inconsistencies were insignificant in the direct and indirect comparisons for depression, anxiety, and mental health. For loop inconsistency, no significant inconsistencies were observed in the outcome measurement of mental health and its subgroup analysis. In the outcome measurements of interventions for depression, subgroup analysis for depression, and subgroup analysis for anxiety, we found evidence of loop inconsistencies in 1/8 (12.5), 7/44 (15.9%), 1/6 (16.7%), and 2/23 (8.7%) loops, and no reason and evidence were found to explain these loop inconsistencies. The detailed outcomes of the inconsistency analysis are presented in Appendices 30–42 (Supplemental Digital Content 5, http://links.lww.com/JS9/B123). For the pairwise meta-analyses with high heterogeneity, a random effect models was used. However, sensitivity analyses were not conducted because only 16.4% of the included RCTs were judged as low risk for overall bias. The comparison-adjusted funnel plots and Egger’s tests indicated no evidence of publication bias in the NMA and pairwise meta-analyses. The detailed information are presented in Appendices 43–49 (Supplemental Digital Content 5, http://links.lww.com/JS9/B123).

## Discussion

This systemic review and NMA included 66 RCTs involving 26 specific treatments in 7 categories, summarizing the most up-to-date evidence on the comparative effectiveness of nonpharmacological interventions in reducing psychological symptoms among patients with CLBP. The pooled outcomes indicated that the majority of nonpharmacological therapies were effective in reducing depression and anxiety and in improving mental health among patients with CLBP. In particular, mind body therapy, biopsychosocial approach, and physical therapy were the top three efficacious interventions for reducing depression compared with the control group. The subgroup analyses showed that mind body therapy, massage, CBT, and PI exhibited a higher probability in reducing depression than the other specific treatments. For the management of anxiety, mind body therapy, multicomponent intervention, and biopsychosocial approach were remarkably efficacious than the control. Among specific treatments, mind body therapy, CBT, PI, multicomponent intervention, and education presented superior probability. For the improvement of mental health, mind body therapy was not included in the NMA due to limited RCTs reporting the outcomes of mind body therapy. The pooled results of the remaining six interventions showed that multicomponent intervention, exercise, and physical therapy were substantially efficacious than the control, and AT had the highest probability within the exercise category. However, rank probability also suggested that physical therapy was ineffective in reducing anxiety compared with the control. For specific treatments, telemedicine and KT were associated with less effectiveness than the control cohorts in reducing depression, while electrical stimulation and KT were associated with lower effectiveness than the control cohorts in reducing anxiety. Thus, the clinical application of these therapeutic treatments requires careful consideration.

Mind body therapy (e.g. yoga, pilates, and Tai Chi) is classified under the categories of complementary medicine and alternative medicine, emphasizing the physical postures of relaxing and stretching the skeletal muscles, breath regulation, and mindfulness or contemplative state^99,100^. Mind body therapy has been used as a therapeutic treatment for CLBP by promoting physical and mental health with promising clinical outcomes^35,88,94^. Our study also indicated that mind body therapy was the most effective type of intervention in reducing depression and anxiety among the included treatments. The results may be attributed to the following reasons. First, the experience of pain is closely tied to emotions, and hypervigilance may be induced in patients with CLBP, increasing psychological symptoms^101,102^. In contrast with aerobic exercise, mind body therapy focuses on controlled and slow movements and regulation of awareness through breathing and meditation, and thus, it has a deep effect not only on the body, but also on the mind and emotions. The combined effects of bodily practices, relaxation of the mind, and control of hypervigilance can have a significant value in mitigating symptoms of depression and anxiety. Second, mind body therapy may alleviate depression and anxiety through the interaction of the psycho-neuro-endocrino-immunological system^35,103^. In typical circumstances, the hypothalamic-pituitary-adrenal axis can be activated by mental stress and emotions related to tension, and cortisol is subsequently released^104^. However, prolonged LBP may lead to a persistent stimulation of this pathway, ultimately resulting in the dysfunction of the endocrine and immune systems^8,105^. Feng *et al*.^106^ found that mind body therapy can alter the sympathetic-adrenal-medullary axis by focusing attention on breathing and reduce the reactivity of the hypothalamic-pituitary-adrenal axis. Thus, mind body therapy is recommended to reduce CLBP and decrease psychological symptoms.

The biopsychosocial approach includes a wide variety of psychotherapies, such as CBT, psychological interventions, motivational interview, and goal setting; it achieves effective outcomes for the treatment of chronic pain and depression^36,107^. This NMA revealed that a biopsychosocial approach was remarkably associated with reducing depression and anxiety in CLBP. In particular, CBT and PI were effective specific treatments that should be prioritized in clinical practice. CBT emphasizes the use of active and structured techniques to educate patients on how to recognize, monitor, and alter maladaptive thoughts, emotions, and behavior^108^. To date, CBT has become one of the most widely applied psychosocial treatments for patients with CLBP, and different forms of CBT, including acceptance and commitment therapy and behavioral activation therapy, have been developed and appeared beneficial for CLBP with depressive symptoms^10,33,34,36–38^. Furthermore, exercise, especially aerobic exercise, can substantially improve mental health, as reported in this study. However, physical therapy demonstrated remarkable effectiveness in reducing depression but was ineffective on anxiety and mental health. The subgroup analyses further indicated that KT was ineffective in depression and anxiety. In Celenay *et al*.^109^, KT was not recommended as a singular intervention for individuals experiencing CLBP. Hence, drawing attention to the application of KT and assessing the psychological symptoms of patients before and after physical intervention are crucial. The assessment of psychological symptoms should be timely to facilitate effective treatment planning of CLBP.

This NMA exhibits the following strengths. First, a comprehensive and up-to-date search for studies that report nonpharmacological interventions for CLBP in reducing psychological symptoms was conducted. The outcomes of this study may provide evidence for the selection of interventions to clinicians in clinical practice. Second, the literature selection, data extraction, and risk of bias assessment were rigorously performed by four reviewers in pairs and divided into two groups. Third, 26 specific treatments for CLBP were classified from the 7 categories of interventions. Furthermore, subgroup analyses were conducted on the basis of specific treatments to provide clinicians with detailed information. Fourth, the certainty of the evidence was evaluated on the basis of GRADE methods for all comparisons and outcomes. Fifth, this study encompassed a broad spectrum of geographical areas, suggesting a robust extrapolation of pooled outcomes.

This NMA also has several limitations. First, most of the included RCTs exhibited concerns for the risk of bias assessment, particularly for deviations from intended interventions and the selection of reported results. Only 15.2% of the RCTs were evaluated to exhibit a low overall risk of bias. Second, a small number of loops in this study demonstrated loop inconsistency between direct and indirect sources of evidence. Thus, the pooled outcomes regarding these comparisons should be interpreted cautiously. Third, most of the certainties of evidence varied from moderate to extremely low, reminding clinicians to consider the effectiveness and side effects of interventions thoroughly in accordance with the quality of evidence while making decisions for CLBP comorbidity with psychological symptoms. Fourth, the number of included RCTs in the subgroup analyses for each specific treatment was relatively small. When interpreting the results of the present study, considering these limitations is important. Fifth, the majority of the included RCTs were conducted in Europe, Asia, and North America. Therefore, attention should be paid to the generalizability of the findings.

## Conclusion

This systemic review and NMA suggested that nonpharmacological interventions show promise for reducing psychological symptoms among patients with CLBP. In particular, mind body therapy and psychological therapy show considerable promise, and mind body therapy can be considered a priority choice in reducing depression and anxiety. These findings can aid clinicians in assessing the potential risks and benefits of available treatments for CLBP comorbidity with psychological symptoms and provide evidence for selecting interventions in clinical practice. More RCTs involving different interventions with rigorous methodology and adequate sample size should be conducted in future research Tables 1–3.

**Table 1:**
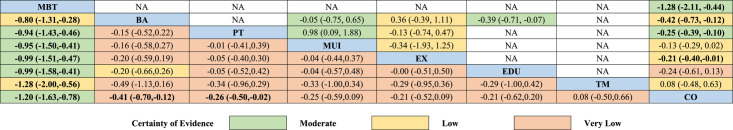
Comparative effectiveness of different categories of interventions for depression.

The league tables show the pooled outcomes of the network meta-analyses (lower diagonal) and pairwise meta-analyses (upper diagonal) for comparative effectiveness of different categories of interventions for depression. The relative effect sizes of each approach were measured as a standardized mean difference and 95% CI. The bold indicates statistical significance. Comparisons between treatments should be read from left to right, and the estimate is in the cell in common between the column-defining treatment and the row-defining treatment. The imprecision for the rating of Certainty of Evidence on direct evidence was not considered. (According to the GRADE, recommended ‘consideration of imprecision is not necessary when rating the direct and indirect estimates to inform the rating of the network estimates’.) The detailed of Certainty of Evidence were presented in Appendices 7 and 9. BA, biopsychosocial approach; CO, control; EX, exercise; EDU, education; MBT, mind body therapy; MUI, multicomponent intervention; NA, not available; PT, physical therapy; TM, telemedicine. Interventions details are described in Appendix 3.

**Table 2:**
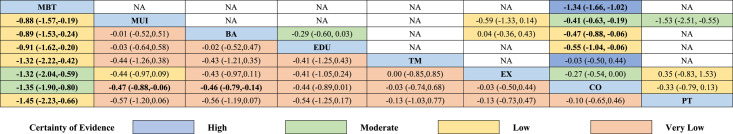
Comparative effectiveness of different categories of interventions for anxiety.

The league tables show the pooled outcomes of the network meta-analyses (lower diagonal) and pairwise meta-analyses (upper diagonal) for comparative effectiveness of different categories of interventions for anxiety. The relative effect sizes of each approach were measured as a standardized mean difference and 95% CI. Bold indicates statistical significance. Comparisons between treatments should be read from left to right, and the estimate is in the cell in common between the column-defining treatment and the row-defining treatment. The imprecision for the rating of Certainty of Evidence on direct evidence was not considered. (According to the GRADE, recommended ‘consideration of imprecision is not necessary when rating the direct and indirect estimates to inform the rating of the network estimates’.) The detailed of Certainty of Evidence were presented in Appendices 7 and 10. BA, biopsychosocial approach; CO, control; EX, exercise; EDU, education; MBT, mind body therapy; MUI, multicomponent intervention; NA, not available; PT, physical therapy; TM, telemedicine. Interventions details are described in Appendix 3.

**Table 3:**
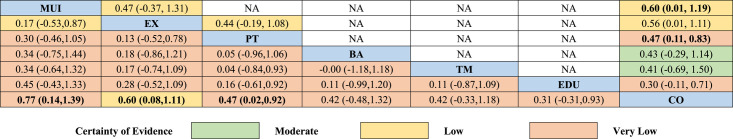
Comparative effectiveness of different categories of interventions for mental health.

Notes: The league tables show the pooled outcomes of the network meta-analyses (lower diagonal) and pairwise meta-analyses (upper diagonal) for comparative effectiveness of different categories of interventions for mental health. The relative effect sizes of each approach were measured as a standardized mean difference and 95% CI. The bold indicates statistical significance. Comparisons between treatments should be read from left to right, and the estimate is in the cell in common between the column-defining treatment and the row-defining treatment. The imprecision for the rating of Certainty of Evidence on direct evidence was not considered. (According to the GRADE, recommended ‘consideration of imprecision is not necessary when rating the direct and indirect estimates to inform the rating of the network estimates’.) The detailed of Certainty of Evidence were presented in Appendices 7 and 11. BA, biopsychosocial approach; CO, control; EX, exercise; EDU, education; MBT, mind body therapy; MUI, multicomponent intervention; NA, not available; PT, physical therapy; TM, telemedicine. Interventions details are described in Appendix 3.

## Ethical approval

This trial is a meta-analysis, which we collected data from other included studies. Ethics approval and consent to participate is not applicable.

## Patient consent

This trial is a meta-analysis, which we collected data from other included studies. Ethics approval and consent to participate is not applicable.

## Sources of funding

This study was supported by the grant from National Key Research and Development Program of China (No.2022YFC2407504).

## Author contribution

L.-P.Z., L.K., J.S., and Z.-G.Z.: performed the study screening, and data extraction; L.-P.Z., L.K., B.Z., and J.-Q.W.: contributed to the assessments for risk of bias and certainty of evidence; L.-P.Z., J.S., C.-Y.J., and H.-Q.Z.: performed data analysis; L.-P.Z., L.K., J.S., Z.-G.Z., C.-H.Z., and X.-L.Z.: contributed to the interpretation of data; L.-P.Z., L.K., J.S., B.Z., J.-Q.W., and C.-Y.J.: prepared the figures and tables; L.-P.Z. drafted the manuscript; C.-L.S. and R.-J.Z.: contributed equally in the design and coordination of the study; C.-L.S., L.-P.Z., R.-J.Z., L.K., and J.S.: conceived the study, and participated in its design; all authors reviewed the manuscript for important intellectual content, and approved the final version of the manuscript; C.-L.S.: is the corresponding author and the study guarantor. The corresponding author attests that all listed authors meet authorship criteria and no others meeting the criteria have been omitted.

## Conflicts of interest disclosure

All authors confirmed that there is no conflict of interest regarding the submitted manuscript.

## Research registration unique identifying number (UIN)

This NMA was performed by following the Preferred Reporting Items for Systematic Reviews (PRISMA) criteria and PRISMA-NMA statement and Assessing the Methodological Quality of Systematic Reviews 2 (AMSTAR 2) guidelines. The protocol of this study was registered in PROSPERO (CRD42023418912).

## Guarantor

Cai-Liang Shen.

## Provenance and peer review

Not commissioned, externally peer-reviewed.

## Data availability statement

All data generated or analyzed during this study are included in this published article and its supplementary information files.

## Supplementary Material

SUPPLEMENTARY MATERIAL

## References

[1] Chen N Fong DYT Wong JYH . Health and economic outcomes associated with musculoskeletal disorders attributable to high body mass index in 192 countries and territories in 2019. JAMA Netw Open 2023;6:e2250674. 36662529 10.1001/jamanetworkopen.2022.50674 PMC9860530

[2] Fatoye F Gebrye T Mbada CE . Clinical and economic burden of low back pain in low- and middle-income countries: a systematic review. BMJ Open 2023;13:e064119. 10.1136/bmjopen-2022-064119 PMC10151982 37185180

[3] Guan SY Zheng JX Sam NB . Global burden and risk factors of musculoskeletal disorders among adolescents and young adults in 204 countries and territories, 1990-2019. Autoimmun Rev 2023;22:103361. 37230312 10.1016/j.autrev.2023.103361

[4] Dunbar MS Rodriguez A Edelen MO . Longitudinal associations of PROMIS-29 anxiety and depression symptoms with low back pain impact in a sample of U.S. military service members. Mil Med 2023;188:e630–e636. 34417805 10.1093/milmed/usab339 PMC10226420

[5] Yang QH Zhang YH Du SH . Association between smoking and pain, functional disability, anxiety and depression in patients with chronic low back pain. Int J Public Health 2023;68:1605583. 36960408 10.3389/ijph.2023.1605583 PMC10027735

[6] Anheyer D Haller H Lauche R . Yoga for treating low back pain: a systematic review and meta-analysis. Pain 2022;163:E504–E17. 34326296 10.1097/j.pain.0000000000002416

[7] Wieland LS Skoetz N Pilkington K . Yoga for chronic non-specific low back pain. Cochrane Database Syst Rev 2022;11:CD010671. 36398843 10.1002/14651858.CD010671.pub3 PMC9673466

[8] Wen YR Shi J Wang YF . Are mind-body exercise beneficial for treating pain, function, and quality of life in middle-aged and old people with chronic pain? A systematic review and meta-analysis. Front Aging Neurosci 2022;14:921069. 35800981 10.3389/fnagi.2022.921069 PMC9255956

[9] Wang XQ Xiong HY Du SH . The effect and mechanism of traditional Chinese exercise for chronic low back pain in middle-aged and elderly patients: a systematic review. Front Aging Neurosci 2022;14:935925. 36299610 10.3389/fnagi.2022.935925 PMC9590689

[10] Khoja O Koculu E Taylor A . The effectiveness of cognitive functional therapy for patients with chronic non-specific low back pain. Arch Phys Med Rehab 2019;100:e153.

[11] Ma J Zhang T He Y . Effect of aquatic physical therapy on chronic low back pain: a systematic review and meta-analysis. BMC Musculoskel Disord 2022;23:1050. 10.1186/s12891-022-05981-8 PMC9717486 36460993

[12] Kogan CS Maj M Rebello TJ . A global field study of the international classification of diseases (ICD-11) mood disorders clinical descriptions and diagnostic guidelines. J Affect Disord 2021;295:1138–1150. 34706426 10.1016/j.jad.2021.08.050

[13] Page MJ McKenzie JE Bossuyt PM . The PRISMA 2020 statement: an updated guideline for reporting systematic reviews. Int J Surg 2021;88:105906. 33789826 10.1016/j.ijsu.2021.105906

[14] Hutton B Salanti G Caldwell DM . The PRISMA extension statement for reporting of systematic reviews incorporating network meta-analyses of health care interventions: checklist and explanations. Ann Intern Med 2015;162:777–784. 26030634 10.7326/M14-2385

[15] Shea BJ Reeves BC Wells G . AMSTAR 2: a critical appraisal tool for systematic reviews that include randomised or non-randomised studies of healthcare interventions, or both. BMJ 2017;358:j4008. 28935701 10.1136/bmj.j4008 PMC5833365

[16] Qaseem A Wilt TJ McLean RM . Noninvasive treatments for acute, subacute, and chronic low back pain: a clinical practice guideline from the american college of physicians. Ann Intern Med 2017;166:514–530. 28192789 10.7326/M16-2367

[17] Sterne JAC Savović J Page MJ . RoB 2: a revised tool for assessing risk of bias in randomised trials. BMJ 2019;366:l4898. 31462531 10.1136/bmj.l4898

[18] Puhan MA Schünemann HJ Murad MH . A GRADE Working Group approach for rating the quality of treatment effect estimates from network meta-analysis. BMJ 2014;349:g5630. 25252733 10.1136/bmj.g5630

[19] Brignardello-Petersen R Bonner A Alexander PE . Advances in the GRADE approach to rate the certainty in estimates from a network meta-analysis. J Clin Epidemiol 2018;93:36–44. 29051107 10.1016/j.jclinepi.2017.10.005

[20] Papakonstantinou T Nikolakopoulou A Higgins JPT . CINeMA: software for semiautomated assessment of the confidence in the results of network meta-analysis. Campbell Syst Rev 2020;16:e1080. 37131978 10.1002/cl2.1080 PMC8356302

[21] Nikolakopoulou A Higgins JPT Papakonstantinou T . CINeMA: an approach for assessing confidence in the results of a network meta-analysis. PLoS Med 2020;17:e1003082. 32243458 10.1371/journal.pmed.1003082 PMC7122720

[22] Higgins JPT Thomas J Chandler J . Cochrane Handbook for Systematic Reviews of Interventions. John Wiley & Sons; 2019.

[23] White IR . Multivariate random-effects metaregression: updates to mvmeta. Stata J 2011;11:255.

[24] White IR . Network meta-analysis. Stata J 2015;15:951–985.

[25] Mutz J Vipulananthan V Carter B . Comparative efficacy and acceptability of non-surgical brain stimulation for the acute treatment of major depressive episodes in adults: systematic review and network meta-analysis. BMJ 2019;364:l1079. 30917990 10.1136/bmj.l1079 PMC6435996

[26] Dias S Welton NJ Caldwell DM . Checking consistency in mixed treatment comparison meta-analysis. Stat Med 2010;29:932–944. 20213715 10.1002/sim.3767

[27] Higgins JP Jackson D Barrett JK . Consistency and inconsistency in network meta-analysis: concepts and models for multi-arm studies. Res Synth Methods 2012;3:98–110. 26062084 10.1002/jrsm.1044 PMC4433772

[28] Bucher HC Guyatt GH Griffith LE . The results of direct and indirect treatment comparisons in meta-analysis of randomized controlled trials. J Clin Epidemiol 1997;50:683–691. 9250266 10.1016/s0895-4356(97)00049-8

[29] Jackson D Riley RD . A refined method for multivariate meta-analysis and meta-regression. Stat Med 2014;33:541–554. 23996351 10.1002/sim.5957 PMC4285306

[30] Begg CB Mazumdar M . Operating characteristics of a rank correlation test for publication bias. Biometrics 1994;50:1088–1101. 7786990

[31] Macaskill P Walter SD Irwig L . A comparison of methods to detect publication bias in meta-analysis. Stat Med 2001;20:641–654. 11223905 10.1002/sim.698

[32] Chaimani A Higgins JP Mavridis D . Graphical tools for network meta-analysis in STATA. PLoS One 2013;8:e76654. 24098547 10.1371/journal.pone.0076654 PMC3789683

[33] Glombiewski JA Hartwich-Tersek J Rief W . Two psychological interventions are effective in severely disabled, chronic back pain patients: a randomised controlled trial. Int J Behav Med 2010;17:97–107. 19967572 10.1007/s12529-009-9070-4

[34] Harris A Moe TF Eriksen HR . Brief intervention, physical exercise and cognitive behavioural group therapy for patients with chronic low back pain (The CINS trial). Eur J Pain (London, England) 2017;21:1397–1407. 10.1002/ejp.1041 28449303

[35] Kuvacic G Fratini P Padulo J . Effectiveness of yoga and educational intervention on disability, anxiety, depression, and pain in people with CLBP: a randomized controlled trial. Complement Ther Clin Pract 2018;31:262–267. 29705466 10.1016/j.ctcp.2018.03.008

[36] Sanabria-Mazo JP Colomer-Carbonell A Borràs X Efficacy of videoconference group . Acceptance and Commitment Therapy (ACT) and Behavioral Activation Therapy for Depression (BATD) for chronic low back pain (CLBP) plus comorbid depressive symptoms: A randomized controlled trial (IMPACT study). J Pain 2023;24:1522–1540. 37105508 10.1016/j.jpain.2023.04.008

[37] Schlicker S Baumeister H Buntrock C . A web- And mobile-based intervention for comorbid, recurrent depression in patients with chronic back pain on sick leave (get.back): Pilot randomized controlled trial on feasibility, user satisfaction, and effectiveness. JMIR Mental Health 2020;7:e16398. 32293577 10.2196/16398 PMC7191351

[38] Soleymani A Arani AM Raeissadat SA . Rumination-focused cognitive-behavioral therapy for chronic low back pain: a randomized controlled trial. Galen Med J 2020;9:e1722. 34466577 10.31661/gmj.v9i0.1722 PMC8343985

[39] Aguilar-Ferrándiz ME Matarán-Peñarrocha GA Tapia-Haro RM . Effects of a supervised exercise program in addition to electrical stimulation or kinesio taping in low back pain: a randomized controlled trial. Scient Rep 2022;12:11430. 10.1038/s41598-022-14154-5 PMC9259681 35794120

[40] Alzahrani H Mackey M Stamatakis E . Wearables-based walking program in addition to usual physiotherapy care for the management of patients with low back pain at medium or high risk of chronicity: a pilot randomized controlled trial. PloS One 2021;16:e0256459. 34437607 10.1371/journal.pone.0256459 PMC8389429

[41] Batibay S Külcü DG Kaleoğlu Ö . Effect of Pilates mat exercise and home exercise programs on pain, functional level, and core muscle thickness in women with chronic low back pain. J Orthop Sci 2021;26:979–985. 33386201 10.1016/j.jos.2020.10.026

[42] Cuesta-Vargas AI Adams N Salazar JA . Deep water running and general practice in primary care for non-specific low back pain versus general practice alone: randomized controlled trial. Clin Rheumatol 2012;31:1073–1078. 22453844 10.1007/s10067-012-1977-5

[43] Darnall BD Roy A Chen AL . Comparison of a single-session pain management skills intervention with a single-session health education intervention and 8 sessions of cognitive behavioral therapy in adults with chronic low back pain: a randomized clinical trial. JAMA Network Open 2021;4:e2113401. 34398206 10.1001/jamanetworkopen.2021.13401 PMC8369357

[44] de Oliveira Meirelles F de Oliveira Muniz Cunha JC da Silva EB . Osteopathic manipulation treatment versus therapeutic exercises in patients with chronic nonspecific low back pain: a randomized, controlled and double-blind study. J Back Musculoskel Rehab 2020;33:367–377. 10.3233/BMR-181355 31658037

[45] Diez GG Anitua E Castellanos N . The effect of mindfulness on the inflammatory, psychological and biomechanical domains of adult patients with low back pain: a randomized controlled clinical trial. PloS One 2022;17:e0276734. 36350802 10.1371/journal.pone.0276734 PMC9645607

[46] Durmus D Durmaz Y Canturk F . Effects of therapeutic ultrasound and electrical stimulation program on pain, trunk muscle strength, disability, walking performance, quality of life, and depression in patients with low back pain: a randomized-controlled trial. Rheumatol Int 2010;30:901–910. 19644691 10.1007/s00296-009-1072-7

[47] Engbert K Weber M . The effects of therapeutic climbing in patients with chronic low back pain: a randomized controlled study. Spine 2011;36:842–849. 21192296 10.1097/BRS.0b013e3181e23cd1

[48] Galantino ML Bzdewka TM Eissler-Russo JL . The impact of modified hatha yoga on chronic low back pain: a pilot study. Alt Ther Health Med 2004;10:56–59. 15055095

[49] Gardner T Refshauge K McAuley J . Combined education and patient-led goal setting intervention reduced chronic low back pain disability and intensity at 12 months: a randomised controlled trial. Br J Sports Med 2019;53:1424–1431. 30808666 10.1136/bjsports-2018-100080

[50] Glombiewski JA Holzapfel S Riecke J . Exposure and CBT for chronic back pain: an RCT on differential efficacy and optimal length of treatment. J Consult Clin Psychol 2018;86:533–545. 29781651 10.1037/ccp0000298

[51] Groenveld TD Smits MLM Knoop J . Effect of a behavioural therapy-based virtual reality application on quality of life in chronic low back pain. Clin J Pain 2023;39:278–285. 37002877 10.1097/AJP.0000000000001110 PMC10205123

[52] Hernandez-Reif M Field T Krasnegor J . Lower back pain is reduced and range of motion increased after massage therapy. Int J Neurosci 2001;106:131–145. 11264915 10.3109/00207450109149744

[53] Hohmann CD Stange R Steckhan N . The effectiveness of leech therapy in chronic low back pain. Dtsch Arztebl Int 2018;115:785–792. 30636672 10.3238/arztebl.2018.0785 PMC6334223

[54] Hrkać A Bilić D Černy-Obrdalj E . Comparison of supervised exercise therapy with or without biopsychosocial approach for chronic nonspecific low back pain: a randomized controlled trial. BMC Musculoskel Dis 2022;23:966. 10.1186/s12891-022-05908-3 PMC9641911 36348309

[55] Huber D Grafetstaetter C Prossegger J . Green exercise and mg-ca-SO4 thermal balneotherapy for the treatment of non-specific chronic low back pain: a randomized controlled clinical trial. BMC Musculoskel Dis 2019;20:221. 10.1186/s12891-019-2582-4 PMC6524239 31096958

[56] Hüppe A Zeuner C Karstens S . Feasibility and long-term efficacy of a proactive health program in the treatment of chronic back pain: a randomized controlled trial. BMC Health Serv Res 2019;19:714. 31639016 10.1186/s12913-019-4561-8 PMC6805578

[57] Kaapa EH Frantsi K Sarna S . Multidisciplinary group rehabilitation versus individual physiotherapy for chronic nonspecific low back pain - a randomized trial. Spine 2006;31:371–376. 16481945 10.1097/01.brs.0000200104.90759.8c

[58] Kader D Radha S Smith F . Evaluation of perifacet injections and paraspinal muscle rehabilitation in treatment of low back pain. A randomised controlled trial. Ortop Traumatol Rehabil 2012;14:251–259. 22764337 10.5604/15093492.1002264

[59] Kanaan SF Alhendi ZM Almhdawi KA . Evaluating the effectiveness of a comprehensive education on low back pain treatment outcomes: a controlled clinical study. Clin Rehabil 2023;37:98–108. 36071623 10.1177/02692155221122661

[60] Karaarslan F Yilmaz H Akkurt HE . Effectiveness of peloid therapy in patients with chronic low back pain: a single-blind controlled study. Int J Biometeorol 2021;65:1799–1809. 33931829 10.1007/s00484-021-02137-6

[61] Kim TH Park SK Cho IY . Substantiating the therapeutic effects of simultaneous heat massage combined with conventional physical therapy for treatment of lower back pain: a randomized controlled feasibility trial. Healthcare (Basel) 2023;11:991. 37046917 10.3390/healthcare11070991 PMC10093909

[62] Kiziltaş Ö Okçu M Tuncay F . Comparison of the effectiveness of conventional physical therapy and extracorporeal shock wave therapy on pain, disability, functional status, and depression in patients with chronic low back pain. Turk J Phys Med Rehabil 2022;68:399–408. 36475112 10.5606/tftrd.2022.8905 PMC9706798

[63] Kogure A Kotani K Katada S . A randomized, single-blind, placebo-controlled study on the efficacy of the arthrokinematic approach-hakata method in patients with chronic nonspecific low back pain. PloS One 2015;10:e0144325. 26646534 10.1371/journal.pone.0144325 PMC4672908

[64] Koldaş Doğan S Sonel Tur B Kurtaiş Y . Comparison of three different approaches in the treatment of chronic low back pain. Clin Rheumatol 2008;27:873–881. 18188660 10.1007/s10067-007-0815-7

[65] Kumar S Rampp T Kessler C . Effectiveness of ayurvedic massage (sahacharadi taila) in patients with chronic low back pain: a randomized controlled trial. J Alt Complem Med 2017;23:109–115. 10.1089/acm.2015.0272 27704865

[66] Lara-Palomo IC Antequera-Soler E Mataran-Penarrocha GA . Comparison of the effectiveness of an e-health program versus a home rehabilitation program in patients with chronic low back pain: a double blind randomized controlled trial. Digit Health 2022;8:20552076221074482. 35111332 10.1177/20552076221074482 PMC8801654

[67] Lawand P Lombardi Junior I Jones A . Effect of a muscle stretching program using the global postural reeducation method for patients with chronic low back pain: a randomized controlled trial. Joint bone spine 2015;82:272–277; Date of publication: 01 jul 2015. 2015. 25881758 10.1016/j.jbspin.2015.01.015

[68] Lazaridou A Paschali M Vilsmark ES . Biofeedback EMG alternative therapy for chronic low back pain (the BEAT-pain study). Digit Health 2023;9:20552076231154386. 36776410 10.1177/20552076231154386 PMC9909059

[69] Mariano TY Burgess FW Bowker M . Transcranial direct current stimulation for affective symptoms and functioning in chronic low back pain: a pilot double-blinded, randomized, placebo-controlled trial. Pain Med (United States) 2019;20:1166–1177. 10.1093/pm/pny188 PMC6544554 30358864

[70] Michalsen A Kunz N Jeitler M . Effectiveness of focused meditation for patients with chronic low back pain-A randomized controlled clinical trial. Complement Ther Med 2016;26:79–84. 27261986 10.1016/j.ctim.2016.03.010

[71] Monticone M Ambrosini E Rocca B . A multidisciplinary rehabilitation programme improves disability, kinesiophobia and walking ability in subjects with chronic low back pain: results of a randomised controlled pilot study. Eur Spine J 2014;23:2105–2113. 25064093 10.1007/s00586-014-3478-5

[72] Nayback-Beebe AM Yoder LH Goff BJ . The effect of pulsed electromagnetic frequency therapy on health-related quality of life in military service members with chronic low back pain. Nursing outlook S 2017;65:S26–S33. 10.1016/j.outlook.2017.07.012 28893387

[73] Newton-John TR Spence SH Schotte D . Cognitive-behavioural therapy versus EMG biofeedback in the treatment of chronic low back pain. Behav Res Ther 1995;33:691–697. 7654161 10.1016/0005-7967(95)00008-l

[74] Niemistö L Lahtinen-Suopanki T Rissanen P . A randomized trial of combined manipulation, stabilizing exercises, and physician consultation compared to physician consultation alone for chronic low back pain. Spine 2003;28:2185–2191. 14520029 10.1097/01.BRS.0000085096.62603.61

[75] Ogunniran IA Akodu AK Odebiyi DO . Effects of kinesiology taping and core stability exercise on clinical variables in patients with non-specific chronic low back pain: a randomized controlled trial. J Bodywork Movement Ther 2023;33:20–27. 10.1016/j.jbmt.2022.09.013 36775519

[76] Petrozzi MJ Leaver A Ferreira PH . Addition of MoodGYM to physical treatments for chronic low back pain: a randomized controlled trial. Chiropract Man Ther 2019;27:54. 10.1186/s12998-019-0277-4 PMC6814139 31673330

[77] Polaski AM Phelps AL Smith TJ . Integrated meditation and exercise therapy: a randomized controlled pilot of a combined nonpharmacological intervention focused on reducing disability and pain in patients with chronic low back pain. Pain Med (Malden, Mass) 2021;22:444–458. 10.1093/pm/pnaa403 PMC7901850 33621332

[78] Ribeiro LH Jennings F Jones A . Effectiveness of a back school program in low back pain. Clin Exp Rheumatol 2008;26:81–88. 18328151

[79] Rim M Leila R Aicha BT . Efficiency of associating therapeutic patient education with rehabilitation in the management of chronic low back pain: a randomized controlled trial. Korean J Fam Med 2022;43:367–373. 36444121 10.4082/kjfm.21.0223 PMC9708858

[80] Schmidt AM Laurberg TB Moll LT . The effect of an integrated multidisciplinary rehabilitation programme for patients with chronic low back pain: long-term follow up of a randomised controlled trial. Clin Rehabil 2021;35:232–241. 33040598 10.1177/0269215520963856 PMC7874370

[81] Seo BK Han K Kwon O . Efficacy of bee venom acupuncture for chronic low back pain: a randomized, double-blinded, sham-controlled trial. Toxins 2017;9:361. 29112155 10.3390/toxins9110361 PMC5705976

[82] Sertpoyraz F Eyigor S Karapolat H . Comparison of isokinetic exercise versus standard exercise training in patients with chronic low back pain: a randomized controlled study. Clin Rehabil 2009;23:238–247. 19218298 10.1177/0269215508099862

[83] Shaygan M Jaberi A Firozian R . Effect of a multimedia training programme for pain management on pain intensity and depression in patients with non-specific chronic back pain. Invest Educ Enferm 2022;40:e13. 10.17533/udea.iee.v40n1e13 PMC9052722 35485626

[84] Singphow C Purohit S Tekur P . Effect of yoga on stress, anxiety, depression, and spinal mobility in computer users with chronic low back pain. Int J Yoga 2022;15:114–121. 36329769 10.4103/ijoy.ijoy_9_22 PMC9623884

[85] Suh JH Kim H Jung GP . The effect of lumbar stabilization and walking exercises on chronic low back pain: a randomized controlled trial. Medicine 2019;98:e16173. 31261549 10.1097/MD.0000000000016173 PMC6616307

[86] Tavafian SS Jamshidi A Mohammad K . Low back pain education and short term quality of life: a randomized trial. BMC Musculoskel Disord 2007;8:21. 10.1186/1471-2474-8-21 PMC1808456 17328809

[87] Tavafian SS Jamshidi AR Mohammad K . Treatment of chronic low back pain: a randomized clinical trial comparing multidisciplinary group-based rehabilitation program and oral drug treatment with oral drug treatment alone. Clin J Pain 2011;27:811–818. 21642845 10.1097/AJP.0b013e31821e7930

[88] Tekur P Nagarathna R Chametcha S . A comprehensive yoga programs improves pain, anxiety and depression in chronic low back pain patients more than exercise: an RCT. Complement Therap Med 2012;20:107–118. 22500659 10.1016/j.ctim.2011.12.009

[89] Trapp W Weinberger M Erk S . A brief intervention utilising visual feedback reduces pain and enhances tactile acuity in CLBP patients. J Back Musculoskelet Rehabil 2015;28:651–660. 25391329 10.3233/BMR-140561

[90] Turner JA Jensen MP . Efficacy of cognitive therapy for chronic low back pain. Pain 1993;52:169–177. 8455964 10.1016/0304-3959(93)90128-C

[91] Tüzün EH Gildir S Angin E . Effectiveness of dry needling versus a classical physiotherapy program in patients with chronic low-back pain: a single-blind, randomized, controlled trial. J Phys Ther Sci 2017;29:1502–1509. 28931976 10.1589/jpts.29.1502 PMC5599809

[92] Ünal M Evci̇k E Kocatürk M . Investigating the effects of myofascial induction therapy techniques on pain, function and quality of life in patients with chronic low back pain. J Bodywork Movement Therap 2020;24:188–195. 10.1016/j.jbmt.2020.07.014 33218510

[93] Weiner DK Rudy TE Glick RM . Efficacy of percutaneous electrical nerve stimulation for the treatment of chronic low back pain in older adults. J Am Geriatr Soci 2003;51:599–608. 10.1034/j.1600-0579.2003.00202.x 12752833

[94] Williams K Abildso C Steinberg L . Evaluation of the effectiveness and efficacy of Iyengar yoga therapy on chronic low back pain. Spine (Phila Pa 1976) 2009;34:2066–2076. 19701112 10.1097/BRS.0b013e3181b315cc PMC4393557

[95] Yaksi E Ketenci A Baslo MB . Does transcutaneous electrical nerve stimulation affect pain, neuropathic pain, and sympathetic skin responses in the treatment of chronic low back pain? A randomized, placebo-controlled study. Kor J Pain 2021;34:217–228. 10.3344/kjp.2021.34.2.217 PMC8019954 33785674

[96] Zhang Y Wan L Wang X . The effect of health education in patients with chronic low back pain. J Int Med Res 2014;42:815–820. 24781721 10.1177/0300060514527059

[97] Zheng F Liu S Zhang S . Does m-health-based exercise (guidance plus education) improve efficacy in patients with chronic low-back pain? A preliminary report on the intervention’s significance. Trials 2022;23:190. 35241140 10.1186/s13063-022-06116-z PMC8892411

[98] Siğlan Ü Çolak S . Effects of diaphragmatic and iliopsoas myofascial release in patients with chronic low back pain: a randomized controlled study. J Bodyw Mov Ther 2023;33:120–127. 36775506 10.1016/j.jbmt.2022.09.029

[99] Zou L Yeung A Li C . Effects of meditative movements on major depressive disorder: a systematic review and meta-analysis of randomized controlled trials. J Clin Med 2018;7:195. 30071662 10.3390/jcm7080195 PMC6111244

[100] Bower JE Irwin MR . Mind-body therapies and control of inflammatory biology: a descriptive review. Brain Behav Immun 2016;51:1–11. 26116436 10.1016/j.bbi.2015.06.012 PMC4679419

[101] Trocoli TO Botelho RV . Prevalence of anxiety, depression and kinesiophobia in patients with low back pain and their association with the symptoms of low back spinal pain. Rev Bras Reumatol Engl Ed 2016;56:330–336. 27476626 10.1016/j.rbre.2016.02.010

[102] Ong AD Zautra AJ Reid MC . Chronic pain and the adaptive significance of positive emotions. Am Psychol 2015;70:283–284. 25844656 10.1037/a0038816

[103] De Giorgio A Dante A Cavioni V . The IARA Model as an integrative approach to promote autonomy in COPD Patients through improvement of self-efficacy beliefs and illness perception: a mixed-method pilot study. Front Psychol 2017;8:1682. 29062286 10.3389/fpsyg.2017.01682 PMC5640890

[104] Ryu H Lee HS Shin YS . Acute effect of qigong training on stress hormonal levels in man. Am J Chin Med 1996;24:193–198. 8874677 10.1142/S0192415X96000256

[105] Yeung A Chan JSM Cheung JC . Qigong and Tai-Chi for mood regulation. Focus (Am Psychiatr Publ) 2018;16:40–47. 31975898 10.1176/appi.focus.20170042 PMC6519567

[106] Feng F Tuchman S Denninger JW . Qigong for the prevention, treatment, and rehabilitation of COVID-19 infection in older adults. Am J Geriatr Psychiatry 2020;28:812–819. 32425471 10.1016/j.jagp.2020.05.012 PMC7227578

[107] Gandy M Pang STY Scott AJ . Internet-delivered cognitive and behavioural based interventions for adults with chronic pain: a systematic review and meta-analysis of randomized controlled trials. Pain 2022;163:e1041–e1053. 35121696 10.1097/j.pain.0000000000002606

[108] Cherkin DC Sherman KJ Balderson BH . Comparison of complementary and alternative medicine with conventional mind-body therapies for chronic back pain: protocol for the Mind-body Approaches to Pain (MAP) randomized controlled trial. Trials 2014;15:211. 24906419 10.1186/1745-6215-15-211 PMC4052284

[109] Celenay TS Kaya DO . Immediate effects of kinesio taping on pain and postural stability in patients with chronic low back pain. J Bodyw Mov Ther 2019;23:206–210. 30691754 10.1016/j.jbmt.2017.12.010

