# Supplementary figures and images for: Comparative effectiveness of nonpharmacological interventions in reducing psychological symptoms among patients with chronic low back pain
Source: Int J Surg. 2023 Sep 26;110(1):478–89. doi: 10.1097/JS9.0000000000000798 (PMC10793751; doi:10.1097/JS9.0000000000000798)

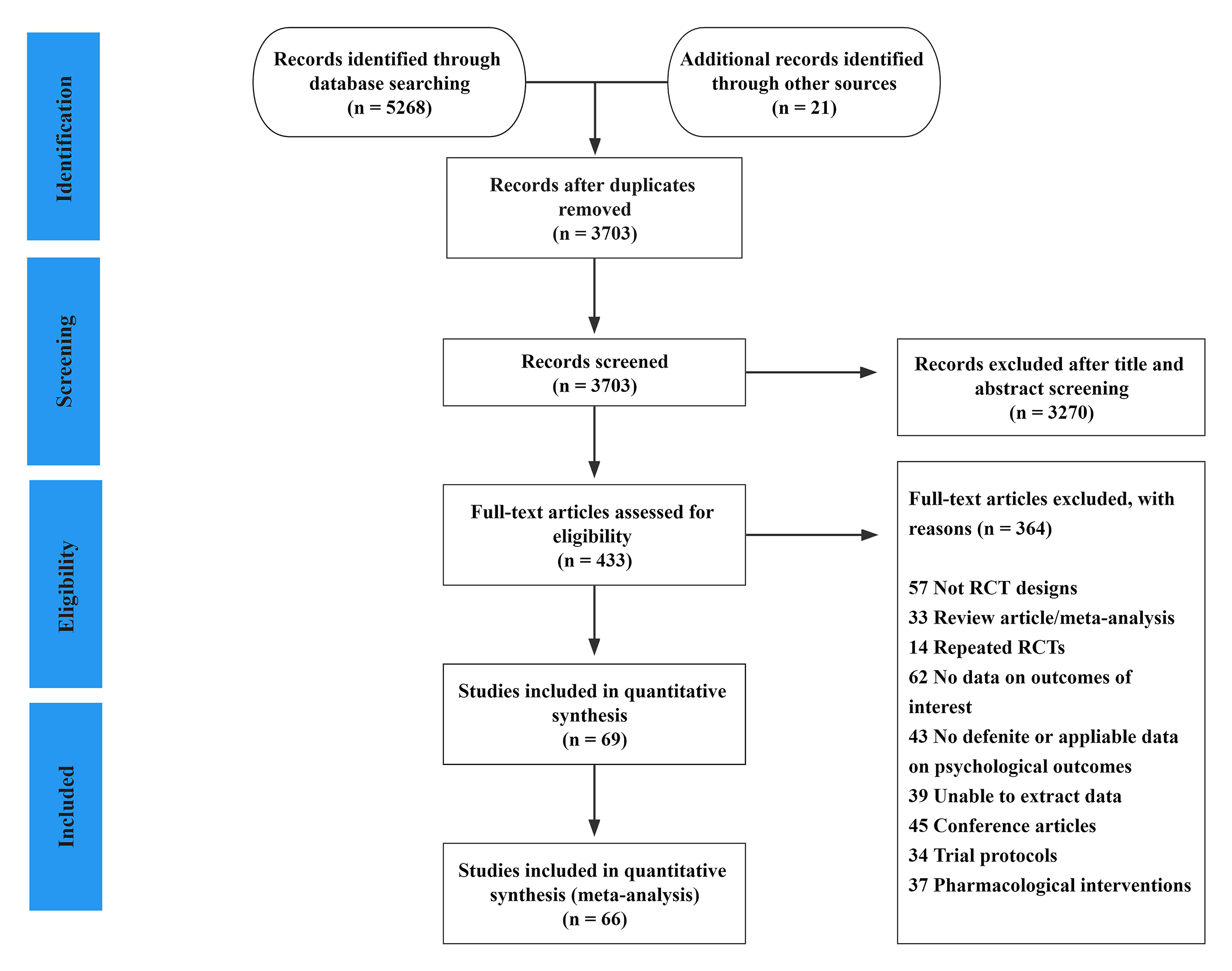

Supplement: SUPPLEMENTARY MATERIAL [file js9-110-478-s002.tif]
