# Supplementary material for: Comparative effectiveness of nonpharmacological interventions in reducing psychological symptoms among patients with chronic low back pain
Source: Int J Surg. 2023 Sep 26;110(1):478–89. doi: 10.1097/JS9.0000000000000798 (PMC10793751; doi:10.1097/JS9.0000000000000798)

Content

Appendix 1. Search terms and database search results..1

Appendix 2. Main characteristics of included randomized controlled trials............................5

Appendix 3. Coding guide and description of different interventions...................................10

Appendix 4. Summary of risk of bias assessment using Risk of bias 2 on included randomized controlled trials...13

Appendix 5. Detailed results of each domain on risk of bias assessment using Risk of bias 2 on included randomized controlled trials...17

Appendix 6. Detailed information of each trial for the risk of bias assessment.....................20

Appendix 7. Certainty of evidence ratings on direct evidence of different categories of interventions for depression, anxiety, and mental health...86

Appendix 8. Certainty of evidence ratings on direct evidence of different specific treatments for depression, anxiety, and mental health..88

Appendix 9. Certainty of evidence ratings on indirect and network evidence of different categories of interventions for depression..92

Appendix 10. Certainty of evidence ratings on indirect and network evidence of different categories of interventions for anxiety..93

Appendix 11. Certainty of evidence ratings on indirect and network evidence of different categories of interventions for mental health...94

Appendix 12. Certainty of evidence ratings on indirect and network evidence of different specific treatments for depression..95

Appendix 13. Certainty of evidence ratings on indirect and network evidence of different specific treatments for anxiety...98

Appendix 14. Certainty of evidence ratings on indirect and network evidence of different specific treatments for mental health...100

Appendix 15. Pooled standardized mean difference and heterogeneity for each direct comparison of different categories of interventions for depression.......................................101

Appendix 16. Comparative effectiveness of different categories of interventions: surface under the cumulative ranking curves (SUCRA) for depression..102

Appendix 17. Network plot of subgroup comparisons in the network meta-analysis of specific treatments for depression..103

Appendix 18. Subgroup analysis of comparative effectiveness of specific treatments for depression...104

Appendix 19. Subgroup analysis of comparative effectiveness of specific treatments: surface under the cumulative ranking curves (SUCRA) for depression..106

Appendix 20. Pooled standardized mean difference and heterogeneity for each direct comparison of different categories of interventions for anxiety..107

Appendix 21. Comparative effectiveness of different categories of interventions: surface under the cumulative ranking curves (SUCRA) for anxiety..108

Appendix 22. Network plot of subgroup comparisons in the network meta-analysis of specific treatments for anxiety...109

Appendix 23. Subgroup analysis of comparative effectiveness of specific treatments for anxiety...110

Appendix 24. Subgroup analysis of comparative effectiveness of specific treatments: surface under the cumulative ranking curves (SUCRA) for anxiety..111

Appendix 25. Pooled standardized mean difference and heterogeneity for each direct comparison of different categories of interventions for mental health.................................112

Appendix 26. Comparative effectiveness of different categories of interventions: surface under the cumulative ranking curves (SUCRA) for mental health.......................................113

Appendix 27. Network plot of subgroup comparisons in the network meta-analysis of specific treatments for mental health..114

Appendix 28. Subgroup analysis of comparative effectiveness of specific treatments for mental health...115

Appendix 29. Subgroup analysis of comparative effectiveness of specific treatments: surface under the cumulative ranking curves (SUCRA) for mental health.......................................116

Appendix 30. Evaluation of global inconsistency for different categories of interventions and specific treatments...117

Appendix 31. Node-splitting method in comparison between direct and indirect evidence of different categories of interventions for depression..118

Appendix 32. Node-splitting method in comparison between direct and indirect evidence of different categories of interventions for anxiety..119

Appendix 33. Node-splitting method in comparison between direct and indirect evidence of different categories of interventions for mental health..120

Appendix 34. Subgroup analysis of node-splitting method in comparison between direct and indirect evidence of specific treatments for depression..121

Appendix 35. Subgroup analysis of node-splitting method in comparison between direct and indirect evidence of specific treatments for anxiety...122

Appendix 36. Subgroup analysis of node-splitting method in comparison between direct and indirect evidence of specific treatments for mental health...123

Appendix 37. Evaluation of inconsistency using loop-specific heterogeneity estimates of different categories of interventions for depression...124

Appendix 38. Evaluation of inconsistency using loop-specific heterogeneity estimates of different categories of interventions for anxiety..125

Appendix 39. Evaluation of inconsistency using loop-specific heterogeneity estimates of different categories of interventions for mental health..126

Appendix 40. Subgroup analysis of evaluation of inconsistency using loop-specific heterogeneity estimates of specific treatments for depression...127

Appendix 41. Subgroup analysis of evaluation of inconsistency using loop-specific heterogeneity estimates of specific treatments for anxiety..129

Appendix 42. Subgroup analysis of evaluation of inconsistency using loop-specific heterogeneity estimates of specific treatments for mental health..130

Appendix 43. Egger's test for the assessment of publication bias of pairwise meta-analysis...131

Appendix 44. Network meta-analysis funnel plots of different categories of interventions for the assessment of publication bias for depression...132

Appendix 45. Network meta-analysis funnel plots of different categories of interventions for the assessment of publication bias for anxiety...133

Appendix 46. Network meta-analysis funnel plots of different categories of interventions for the assessment of publication bias for mental health..134

Appendix 47. Subgroup analysis of meta-analysis funnel plots of specific treatments for the assessment of publication bias for depression..135
Appendix 48. Subgroup analysis of network meta-analysis funnel plots of specific treatments for the assessment of publication bias for anxiety..136
Appendix 49. Subgroup analysis of network meta-analysis funnel plots of specific treatments for the assessment of publication bias for mental health...137

Appendix 1. Search terms and database search results (from database inception to 4 May 2023)

Search	Query	Records
**PubMed**		
#1	"Back Pain"[MeSH Terms] OR "Low Back Pain"[MeSH Terms] OR "Back Pain"[Title/Abstract] OR "Lumbago"[Title/Abstract] OR "Backache"[Title/Abstract] OR "back ache*"[Title/Abstract] OR "spinal stenosis"[Title/Abstract] OR "Canal Stenosis"[Title/Abstract] OR "Lumbar Stenosis"[Title/Abstract] OR "Lateral Stenosis "[Title/Abstract] OR "Neurogenic Claudication"[Title/Abstract] OR "Radiculopathy"[Title/Abstract] OR "Radicular Pain"[Title/Abstract] OR "Spondylolisthesis"[Title/Abstract] OR "Spondylosis"[Title/Abstract] OR "Sciatica"[Title/Abstract] OR "Intervertebral Disc Displacement"[Title/Abstract] OR "Spinal Nerve Roots"[Title/Abstract] OR "Neurologic Signs"[Title/Abstract] OR "Paresthesia"[Title/Abstract] OR "Paraesthesia"[Title/Abstract] OR "Numbness"[Title/Abstract]	119,441
#2	"Randomized Controlled Trial" [Publication Type] OR Randomized[Title/Abstract] OR Randomised[Title/Abstract] OR Randomization[Title/Abstract] OR Randomisation[Title/Abstract]	1,009,563
#3	"Anxiety"[Mesh] OR anxiety[Title/Abstract] OR Angst[Title/Abstract] OR Nervousness[Title/Abstract] OR Hypervigilance[Title/Abstract] OR Anxiousness[Title/Abstract] OR "Social Anxiety"[Title/Abstract] OR "Social Anxieties"[Title/Abstract]	287,998
#4	"Depression"[Mesh] OR Depression[Title/Abstract] OR "Depressive Symptom"[Title/Abstract] OR "Emotional Depression"[Title/Abstract]	458,489
#5	"psychology"[Mesh] OR psychology[Title/Abstract] OR "Psychological Side Effect"[Title/Abstract] OR "Psychosocial Factor"[Title/Abstract] OR Psychologist[Title/Abstract]	124,378
#6	"Mental Health"[Mesh] OR "Mental Health"[Title/Abstract] OR "Mental hygiene"[Title/Abstract]	238,489

#7	"Psychotic Disorders"[Mesh] OR "Psychotic Disorder"[Title/Abstract] OR Psychosis[Title/Abstract] OR Psychoses[Title/Abstract] OR "Schizoaffective Disorder"[Title/Abstract] OR "Schizophreniform Disorder"[Title/Abstract] OR "Brief Reactive Psychoses"[Title/Abstract] OR "Brief Reactive Psychosis"[Title/Abstract]	87,045
#8	#3 OR #4 OR #5 OR #6 OR #7	961,032
#9	#1 AND #2 AND #8	899
**Web of Science**		
#1	TS=("back pain" OR "low back pain" OR lumbago OR backache OR "back ache" OR "spinal stenosis" OR "canal stenosis" OR "lumbar stenosis" OR "lateral stenosis " OR "neurogenic claudication" OR radiculopathy OR "radicular pain" OR spondylolisthesis OR spondylosis OR sciatica OR "intervertebral disc displacement" OR "spinal nerve roots" OR "neurologic signs" OR paresthesia OR paraesthesia OR numbness)	236,441
#2	TS=(randomized controlled trial)	725,392
#3	TS=(anxiety OR angst OR nervousness OR hypervigilance OR anxiousness OR "social anxiety" OR "social anxieties")	555,643
#4	TS=(depression OR "depressive Symptom" OR "emotional depression")	981,681
#5	TS=(psychology OR "psychological side effect" OR "psychosocial factor" OR psychologist)	1,914,965
#6	TS=("mental health" OR "mental hygiene")	430,370
#7	TS=("psychotic disorders" OR psychosis OR psychoses OR "schizoaffective disorder" OR "schizophreniform disorder" OR "brief reactive psychoses" OR "brief reactive psychosis")	147,586
#8	#1 AND #2 AND (#3 OR #4 OR #5 OR #6 OR #7)	2,479
**EMBASE**		
#1	'back pain'/exp OR 'low back pain'/exp OR 'back pain*':ab,ti OR 'lumbago':ab,ti OR 'backache*':ab,ti OR 'back ache*':ab,ti OR 'spinal	210,281

	stenosis':ab,ti OR 'canal stenosis':ab,ti OR 'lumbar stenosis':ab,ti OR 'lateral stenosis':ab,ti OR 'neurogenic claudication':ab,ti OR 'radiculopathy':ab,ti OR 'radicular pain':ab,ti OR 'spondylolisthesis':ab,ti OR 'spondylosis':ab,ti OR 'sciatica':ab,ti OR 'intervertebral disc displacement':ab,ti OR 'spinal nerve roots':ab,ti OR 'neurologic signs':ab,ti OR 'paresthesia':ab,ti OR 'paraesthesia':ab,ti OR 'numbness':ab,ti	
#2	'randomized controlled trial'/exp OR 'randomized controlled trial':ti,ab OR 'randomized':ti,ab OR 'randomised':ti,ab OR 'randomisation':ti,ab OR 'randomization':ti,ab OR 'rct':ti,ab OR 'randomly':ti,ab OR 'placebo':ti,ab	1,800,224
#3	'anxiety'/exp OR 'anxiety' OR 'anxiety':ti,ab OR 'angst':ti,ab OR 'nervousness':ti,ab OR 'hypervigilance':ti,ab OR 'anxiousness':ti,ab OR 'social anxiety':ti,ab OR 'social anxieties':ti,ab	503,287
#4	'depression'/exp OR 'central depression':ti,ab OR 'clinical depression':ti,ab OR 'depressive disease':ti,ab OR 'depressive disorder':ti,ab OR 'depressive episode':ti,ab OR 'depressive illness':ti,ab OR 'depressive personality disorder':ti,ab OR 'depressive state':ti,ab OR 'depressive symptom':ti,ab OR 'depressive syndrome':ti,ab OR 'mental depression':ti,ab OR 'parental depression':ti,ab	632,235
#5	'psychology'/exp OR 'psychology':ti,ab OR 'cognitive science':ti,ab OR 'schizophrenic psychology':ti,ab	470,988
#6	'mental health'/exp OR 'mental care':ti,ab OR 'mental condition':ti,ab OR 'mental factor':ti,ab OR 'mental help':ti,ab OR 'mental service':ti,ab OR 'mental state':ti,ab OR 'mental status':ti,ab OR 'mental status schedule':ti,ab OR 'psychic health':ti,ab	268,694
#7	'psychosis'/exp	346,169
#8	#3 OR #4 OR #5 OR #6 OR #7	1,743,151
#9	#1 AND #2 AND #8	3,264
**Cochrane**		
#1	"back pain":ti,ab,kw OR "low back pain":ti,ab,kw OR "back pain":ti,ab,kw	29,864

	OR "lumbago":ti,ab,kw OR "backache":ti,ab,kw OR "back ache":ti,ab,kw OR "spinal stenosis":ti,ab,kw OR "canal stenosis":ti,ab,kw OR "lumbar stenosis":ti,ab,kw OR "lateral stenosis ":ti,ab,kw OR "neurogenic claudication":ti,ab,kw OR "radiculopathy":ti,ab,kw OR "radicular pain":ti,ab,kw OR "spondylolisthesis":ti,ab,kw OR "spondylosis":ti,ab,kw OR "sciatica":ti,ab,kw OR "intervertebral disc displacement":ti,ab,kw OR "spinal nerve roots":ti,ab,kw OR "neurologic signs ":ti,ab,kw OR "paresthesia":ti,ab,kw OR "paraesthesia":ti,ab,kw OR "numbness":ti,ab,kw	
#2	"randomized":ti,ab,kw OR "randomised":ti,ab,kw OR "randomization":ti,ab,kw OR "randomisation":ti,ab,kw OR "randomly":ti,ab,kw OR "placebo":ti,ab,kw OR "trial":ti,ab,kw OR "Random Allocation":ti,ab,kw OR "Randomized Controlled Trial":ti,ab,kw	1,447,708
#3	"anxiety":ti,ab,kw OR "angst":ti,ab,kw OR "nervousness":ti,ab,kw OR "hypervigilance":ti,ab,kw OR "Anxiousness":ti,ab,kw OR "social anxiety":ti,ab,kw OR "social anxieties":ti,ab,kw	97,215
#4	"depression":ti,ab,kw OR "depressive symptom":ti,ab,kw OR "emotional depression":ti,ab,kw	105,781
#5	"psychology":ti,ab,kw OR "psychological side effect":ti,ab,kw OR "psychosocial factor":ti,ab,kw OR "psychologist":ti,ab,kw	125,845
#6	"mental health":ti,ab,kw OR "mental hygiene":ti,ab,kw	27,858
#7	"psychotic disorder":ti,ab,kw OR "psychosis":ti,ab,kw OR "psychoses":ti,ab,kw OR "schizoaffective disorder":ti,ab,kw OR "schizophreniform disorder":ti,ab,kw OR "brief reactive psychoses":ti,ab,kw OR "brief reactive psychosis":ti,ab,kw	
#8	#3 OR #4 OR #5 OR #6 OR #7	259,509
#9	#1 AND #2 AND #8	4,354

Appendix 2. Main characteristics of included randomized controlled trials

Study	Country/ Region	Intervention Group				Comparison Group				Follow-up	Outcome Measures
		Group (No. of Patients)	Age (M ± SD)	BMI (M ± SD)	Female, n (%)	Group (No. of Patients)	Age (M ± SD)	BMI (M ± SD)	Female, n (%)		
Sığlan 2023	Turkey	Myofascial group (21)	38.0 (7.78)	26.42 (4.57)	9 (42.86)	Control group (21)	38.76 (8.96)	26.31 (5.65)	13 (61.90)	4 weeks	Depression (BDI)
Sanabria-Mazo 2023	Spain	Acceptance and commitment therapy group (78)	54.9 (8.3)	NR	54 (69.2)	Treatment-as-usual group (78)	53.8 (10.0)	NR	51 (65.4)	12 weeks	Depression (DASS) and Anxiety (DASS)
		Behavioral activation therapy group (78)	54.9 (10.2)	NR	53 (67.9)						
Ogunniran 2023	Nigeria	Kinesiology taping and core-stabilization exercises group (13)	42.15 (12.05)	26.15 (3.09)	NR	Core-stabilization exercises group (17)	45.29 (10.79)	26.48 (4.16)	NR	8 weeks	Depression (HADS) and Anxiety (HADS)
		Kinesiology taping group (13)	43.69 (9.53)	27.97 (2.99)	NR						
Lazaridou 2023	U.S.A.	Biofeedback EMG alternative therapy (37)	46.0 (13.9)	NR	NR	Usual care group (29)	43.5 (13.9)	NR	NR	12 weeks	Depression (HADS) and Anxiety (HADS)
Kim 2023	South Korea	Heat massage group (20)	56.30 (12.28)	25.83 (2.62)	11 (55.0)	Physical therapy group (20)	56.15 (9.75)	25.93 (4.10)	10 (50.0)	4 weeks	Depression (BDI)
Kanaan 2023	Jordan	Comprehensive education (27)	46.44 (10.88)	28.28 (10.88)	22 (81)	Standard physical therapy (27)	47.07 (11.53)	NR	21 (78)	3 months	Depression (DASS) and Anxiety (DASS)
Groenveld 2023	The Netherlands	Self-administered behavioural therapy-based virtual reality (VR) application (20)	51 (2.9)	NR	17 (85)	Standard care (20)	52 (2.5)	NR	16 (80)	4 months	Depression (HADS), Anxiety (HADS), and Mental Health (SF-12)
Zheng 2022	China	M-health-based exercise (20)	34.0 (14.4)	21.5 (2.7)	14 (70)	Exercise only (20)	34.9 (14.5)	22.3 (3.6)	12 (60)	18 weeks	Depression (SDS), Anxiety (GAD-7), and Mental Health (SF-36)
Singphow 2022	India	Yoga group (39)	43.74 (7.26)	27.77 (3.67)	15 (38.5)	Physical exercise group (38)	41.47 (9.53)	27.34 (2.91)	13 (34.2)	16 weeks	Depression (DASS) and Anxiety (DASS)
Shaygan 2022	Iran	Multimedia method (30)	51.0 (9.7)	NR	20 (66.7)	Routine training (30)	53.2 (12.6)	NR	25 (83.3)	Control group: 9.73 (6.2) weeks; and Intervention group: 7.83 (5.26) weeks	Depression (BDI)
Rim 2022	Tunisia	Therapeutic patient education (50)	45.6 (6.7)	NR	10 (20)	Usual care (50)	42.9 (8.7)	NR	14 (24)	One year	Depression (HADS) and Anxiety (HADS)
Lara-Palomo 2022	Spain	Internet-based E-Health program group (39)	41.9 (9.4)	NR	22 (56.4)	Home rehabilitation program group (35)	54.6 (12.9)	NR	21 (60.0)	6 months	Mental Health (SF-36)

Kızıltaş 2022	Turkey	Extracorporeal shock wave therapy (36)	47.4 (14.3)	28.6 (4.9)	13 (36.1)	Conventional physical therapy (34)	45.3 (12.2)	35.2 (36.2)	20 (58.8)	12 weeks	Depression (BDI)
Hrkać 2022	Croatia	Cognitive-behavioral therapy, group-based combined exercise therapy and education (59)	49.2 (11.6)	26.4 (4.1)	34 (57.6)	Usual care (58)	50.2 (11.2)	27.0 (3.7)	40 (69.0)	6 months	Depression (HADS), Anxiety (HADS), and Mental Health (SF-12)
		Supervised group-based combined exercise therapy and education (63)	48.6 (12.3)	26.3 (3.7)	40 (63.5)						
Diez 2022	Spain	Mindfulness based intervention (36)	NR	NR	NR	Usual care (34)	NR	NR	NR	8 weeks	Depression (DASS), Anxiety (DASS), and Mental Health (SF-36)
Aguilar-Ferrándiz 2022	Spain	Exercises-kinesio taping group (29)	44 (9)	NR	17 (58.62)	Exercises-analgesic current group (29)	46 (5)	NR	21 (72.41)	One month	Depression (BDI) and Anxiety (BDI)
Yakşi 2021	Turkey	Burst transcutaneous electrical nerve stimulation group (25)	45.6 (9.4)	28.6 (4.8)	16 (64.0)	Placebo transcutaneous electrical nerve stimulation group (23)	40.8 (11.5)	29.9 (4.6)	18 (78.0)	3 months	Depression (BDI)
		Conventional transcutaneous electrical nerve stimulation group (25)	43.2 (12.8)	27.4 (4.1)	13 (52.0)						
Schmidt 2021	Denmark	Integrated multidisciplinary rehabilitation programme group (65)	51 (12)	NR	50 (77)	Existing programme group (67)	54 (12)	NR	49 (73)	One year	Depression (MDI)
Polaski 2021	U.S.A.	Integrated meditation and exercise therapy group (18)	36.3 (14.1)	24.5 (2.9)	13 (72.2)	Audiobook control group (20)	38.7 (16.8)	26.3 (2.7)	13 (65.0)	48 hours	Anxiety (STAI)
Karaarslan 2021	Turkey	Peloid therapy and home exercise program (53)	49.66 (9.26)	28.70 (26.25–33.05)	39 (73.6)	Only home exercise program (53)	44.74 (11.92)	27.30 (23.95–30.20)	39 (73.6)	One month	Depression (BDI) and Mental Health (SF-36)
Darnall 2021	U.S.A.	Empowered relief group (87)	49.7 (15.0)	27.3 (6.0)	44 (50.6)	Health education group (88)	48.0 (13.2)	26.7 (6.3)	47 (53.4)	3 months	Depression (PROMIS) and Anxiety (PROMIS)
		Cognitive behavioral therapy group (88)	45.9 (13.1)	27.0 (6.5)	40 (45.5)						
Batıbay 2021	Turkey	Pilates group (28)	49.3 (10.4)	25.0 (2.6)	NR	home exercise group (25)	48.4 (9.3)	26.3 (2.7)	NR	8 weeks	Depression (BDI)
Alzahrani 2021	Saudi Arabia	Wearables-based walking intervention and usual physiotherapy care (12)	49.0 (13.4)	29.30 (7.59)	3 (11.5)	Usual physiotherapy care (14)	39.0 (13.8)	29.44 (6.25)	8 (30.8)	26 weeks	Depression (BDI)
Ünal 2020	Turkey	Myofascial induction therapy (20)	41.25 (9.12)	24.65 (2.24)	10 (50)	Pain neuroscience education (20)	42.6 (7.96)	25.85 (3.36)	10 (50)	8 weeks	Mental Health (SF-36)
Soleymani 2020	Iran	Rumination-focused cognitive-behavioral therapy (15)	NR	NR	11 (36.6)	Usual care (15)	NR	NR	10 (33.3)	3 months	Depression (DASS) and Anxiety (DASS)
Schlicker 2020	Germany	Cognitive behavioral therapy and Web- and mobile-based guided self-help intervention (40)	51.3 (8.60)	NR	26 (65)	Waitlist control group (36)	50.1 (7.00)	NR	29 (81)	6 months	Anxiety (HADS)
de Oliveira Meirelles 2020	Brazil	Osteopathic manipulation treatment group (20)	46.0 (10.4)	27.1 (4.2)	16 (57)	Active control group (18)	50.1 (9.3)	26.5 (4.0)	12 (43)	Measurement at the end of treatment	Depression (BDI)
Suh 2019	South Korea	Flexibility exercise group (13)	53.54 (15.69)	NR	8 (61.5)	Walking exercise group (13)	54.15 (13.89)	NR	11 (84.6)	6 weeks	Depression (BDI)
		Stabilization exercise group (10)	57.40 (15.88)	NR	6 (60.0)						

		Stabilization with walking exercise group (12)	54.75 (14.98)	NR	8 (66.7)						
Petrozzi 2019	Australia	MoodGYM plus physical treatments (54)	50.1 (12.8)	27.0 (5.0)	29 (53.7)	physical treatments alone (54)	50.6 (14.4)	26.7 (4.0)	25 (59.3)	12 months	Depression (DASS) and Anxiety (DASS)
Mariano 2019	U.S.A.	Transcranial direct current stimulation (10)	65.7 (8.8)	NR	1 (10.0)	Sham group (11)	60.7 (11.8)	NR	2 (18.2)	6 weeks	Depression (PHQ-9) and Anxiety (GAD-7)
Hüppe 2019	Germany	Comprehensive health program (189)	53.4 (8.1)	27.6 (4.8)	60 (31.7)	Control group (255)	53.6 (8.7)	27.2 (4.9)	103 (40.4)	24 months	Depression (PHQ-4) and Mental Health (SF-12)
Huber 2019	Austria	Green exercise group (27)	52.85 (6.43)	24.78 (2.73)	14 (51.9)	Control group without any intervention (27)	43.81 (12.07)	25.06 (3.18)	17 (63.0)	120 days	Mental Health (SF-36)
		Green exercise and balneotherapy group (26)	53.35 (8.26)	26.32 (4.47)	14 (53.8)						
Gardner 2019	Australia	Education combined with patientled goal setting group (37)	44 (12.5)	NR	18 (48.6)	Standardised exercise programme group (38)	45 (13.8)	NR	25 (65.8)	12 months	Depression (DASS) and Anxiety (DASS)
Kuvacic 2018	Croatia	Yoga group (15)	33.6 (4.30)	NR	6 (40.0)	Pamphlet group (15)	34.7 (4.83)	NR	8 (53.3)	one week	Depression (SDS) and Anxiety (SAS)
Hohmann 2018	Germany	Leech therapy (25)	59.29 (6.99)	27.69 (5)	NR	Exercise therapy (19)	56.53 (7.8)	25.53 (5.2)	NR	56±5 days	Depression (CES) and Mental Health (SF-36)
Glombiewski 2018	Germany	Exposure-long group (30)<br>Exposure-short group (26)	52.7 (9.4)<br>51.8 (9.2)	NR<br>NR	12 (38.5)<br>13 (50.0)	Cognitive-behavioral therapy group (32)	53.5 (9.0)	NR	23 (71.9)	6 months	Depression (HADS)
Tüzün 2017	Northern Cyprus	Dry needling therapy (18)	50.1 (11.8)	29.6 (6.1)	8 (44.4)	Classical physiotherapy (16)	50.9 (12.5)	27.9 (4.4)	12 (75.0)	Measurement at the end of treatment	Depression (BDI)
Seo 2017	South Korea	Bee venom acupuncture group (27)	49.85 (14.44)	NR	18 (66.67)	Sham group (27)	50.07 (11.06)	NR	23 (85.19)	12 weeks	Depression (BDI)
Nayback-Beebe 2017	U.S.A.	Pulsed electromagnetic frequency therapy and usual care group (36)	19-60	NR	NR	Usual care group (32)	19-60	NR	NR	8 weeks	Depression (PHQ-9) and Anxiety (GAD-7)
Kumar 2017	Germany	Ayurvedic massage group (32)	55.4 (11.2)	25.8 (4.2)	26 (81.25)	Control group (32)	54.2 (13.8)	26.9 (4.4)	23 (71.88)	4 weeks	Depression (POMS) and Mental Health (SF-36)
Harris 2017	Norway	Cognitive-behavioural treatment and a brief cognitive intervention (55)	45.5 (9.1)	NR	NR	Brief cognitive intervention (99)	44.8 (9.7)	NR	NR	12 months	Depression (HADS) and Anxiety (HADS)
		Physical exercise and a brief cognitive intervention (60)	44.2 (10.6)	NR	NR						
Michalsen 2016	Germany	Focused meditation group (32)	55.5 (10.6)	NR	29 (90.6)	Self-care exercise group (36)	54.8 (10.6)	NR	23 (63.9)	8 Weeks	Depression (HADS), Anxiety (HADS), and Mental Health (SF-36)
Trapp 2015	Germany	Visual feedback group (15)	45.53 (7.05)	NR	5 (33.3)	Control group (15)	40.60 (10.67)	NR	9 (60.0)	Measurement at the end of treatment	Depression (BDI)
Lawand 2015	Brazil	Muscle stretching program using global postural reeducation method (31)	49.4 (12.0)	26.17 (2.95)	25 (80.6)	Control group (30)	47.5 (11.9)	26.22 (3.18)	22 (73.3)	6 months	Depression (BDI) and Mental Health (SF-36)
Kogure 2015	Japan	Arthrokinematic Approach-Hakata group (90)	60.0 (12.7)	23.7 (5.4)	54 (60.0)	Sham group (89)	59.6 (13.3)	22.6 (4.7)	57 (64.0)	6 months	Mental Health (SF-36)
Zhang 2014	China	Health education group (27)	22.29	21.02	9 (33.3)	Exercise group (27)	23.04	21.41	11 (40.7)	Measurement at the	Mental Health (SF-36)

			(2.85)	(2.85)			(2.24)	()1.95		end of treatment	
Monticone 2014	Italy	Multidisciplinary rehabilitation programme group (10)	58.9 (16.4)	27.4 (4.9)	7 (70)	Usual-care alone group (10)	56.6 (14.4)	25.2 (3.1)	4 (40)	3 months	Mental Health (SF-36)
Tekur 2012	India	Yoga group (40)	49 (3.6)	NR	21 (52.5)	Control group (40)	48 (4.0)	NR	15 (37.5)	Measurement at the end of treatment	Depression (BDI) and Anxiety (STAI)
Kader 2012	U.K.	Back education and gym ball exercise (20)	18-65	NR	NR	Back education and standard physiotherapy (17)	18-65	NR	NR	10 Weeks	Depression (Modified Zung epression index) and Mental Health (SF-36)
		Perifacet injection (19)	18-65	NR	NR						
Cuesta-Vargas 2012	Spain	Deep water running and standard general practice group (29)	38.6 (12.2)	26.2 (3.5)	17 (59)	Standard general practice alone group (29)	37.8 (13.2)	25.2 (4.1)	16 (56)	12 months	Mental Health (SF-12)
Tavafian 2011	Iran	Multidisciplinary rehabilitation program (97)	44.6 (10.2)	NR	71 (73.2)	Control group (100)	45.9 (11.3)	NR	83 (83.0)	6 months	Mental Health (SF-36)
Engbert 2011	Germany	Therapeutic climbing group (10)	51.9	NR	6 (60.0)	Standard exercise group (13)	50.4	NR	6 (46.2)	Measurement at the end of treatment	Mental Health (SF-36)
Glombiewski 2010	Germany	Cognitive-behavioural therapy including biofeedback tools group (62)	48.9 (10.5)	NR	41 (66.1)	Cognitive-behavioural therapy group (54)	48.6 (13.1)	NR	36 (66.7)	6 months	Depression (BDI)
Durmus 2010	Turkey	Electrical stimulation and exercises group (20)	49.00 (7.87)	30.50 (5.37)	NR	Only exercises group (20)	47.05 (12.46)	28.50 (1.84)	NR	6 weeks	Depression (BDI) and Mental Health (SF-36)
		Ultrasound therapy and exercises group (19)	48.31 (8.95)	28.89 (3.98)	NR						
Williams 2009	Canada	Iyengar yoga group (43)	48.4 (1.86)	25.8 (0.57)	32 (74.4)	Control group receiving standard medical care (47)	47.6 (1.47)	27.4 (0.60)	37 (78.7)	6 months	Depression (BDI)
Sertpoyraz 2009	Turkey	Isokinetic exercise group (20)	38.75 (7.81)	NR	16 (80)	Standard exercise group (20)	38.25(7.36)	NR	15 (75)	7 weeks	Depression (BDI)
Ribeiro 2008	Brazil	Back school program group (26)	48.1 (14,1)	27.1 (4.8)	19 (73.1)	control group for weekly medical visits (29)	52.8 (10)	27.1 (3.2)	26 (89.7)	120 days	Depression (BDI) and Anxiety (STAI)
Koldaş Doğan 2008	Turkey	Aerobic exercise and home exercise group (19)	37.1 (6.5)	NR	15 (78.9)	Home exercise only group (18)	42.1 (9.5)	NR	14 (77.8)	1 month	Depression (BDI)
		Physical therapy (hot pack, ultrasound, TENS) and home exercise group (18)	41.5 (8.3)	NR	14 (77.8)						
Tavafian 2007	Iran	Back school group (50)	42.9 (10.7)	NR	NR	Clinic group (52)	44.7 (10.8)	NR	NR	3 months	Mental Health (SF-36)
Kaapa 2006	Finland	Multidisciplinary rehabilitation group (59)	46 (7.9)	25 (4.8)	59 (100)	Individual physiotherapy group (61)	46.5 (7.0)	26.5 (4.7)	61 (100)	24 months	Depression (DEPS)
Galantino 2004	U.S.A.	Modified hatha yoga group (11)	30-65	NR	NR	Wait-list group (11)	30-65	NR	NR	3 months	Depression (BDI)
Weiner 2003	U.S.A.	Electrical nerve stimulation and physical therapy group (17)	74.1 (4.6)	NR	11 (64.7)	Sham electrical nerve stimulation and physical therapy group (17)	73.5 (5.7)	NR	7 (41.2)	3 months	Depression (Geriatric Depression Scale)
Niemisto 2003	Finland	Combined Manipulation, stabilizing exercises, and physician consultation	37.3 (5.6)	NR	56 (55)	Physician consultation alone (102)	36.7 (5.6)	NR	54 (53)	12 months	Depression (DEPS)

		(102)									
Hernandez-Reif 2001	U.S.A.	Massage group (12)	43.8 (13.7)	NR	7 (58.3)	Relaxation training group (12)	36.7 (16.1)	NR	6 (50.0)	5 weeks	Depression (POMS) and Anxiety (STAI)
Newton-John 1995	U.K.	Cognitive-behaviourai therapy group (16)	44.37 (12.64)	NR	NR	Wait-list group (12)	47.72 (9.76)	NR	NR	6 months	Depression (BDI) and Anxiety (STAI)
		Electromyographic biofeedback group (16)	44.93 (11.91)	NR	NR						
Turner 1993	U.S.A.	Cognitive therapy group (23)	22-62	NR	NR	Wait-list group (30)	22-62	NR	NR	12 months	Depression (BDI)
		Relaxation training group (24)	22-62	NR	NR						
		Cognitive therapy and Relaxation training group (25)	22-62	NR	NR						

Notes: Continuous were reported as mean±standard deviation (M ± SD), and dichotomous are reported as n (%), n is the number of participants with chronic low back pain. BMI, Body mass index; No., Number; NR: not reported; BDI, the Beck Depression Inventory; DASS, the Depression Anxiety and Stress Scale; HADS, the Hospital Anxiety and Depression Scale; SF-12/36, 12/36-item short form health survey; GAD-7, Generalized Anxiety Disorder-7; MDI, Major Depression Inventory; STAI, the State/Trait Anxiety Inventory; PROMIS, Patient-Reported Outcomes Measurement Information System short-form measures; PHQ-4/9, the Patient Health Questionnaire-4/9; GAD-7, Generalized Anxiety Disorder-7; SDS, the Self-Rating Depression Scale; SAS, Zung self-rating anxiety scale; CES, Center for Epidemiologic Studies; POMS, the Profile of Mood states; DEPS, Finnish Depression Questionnaire.

Appendix 3. Coding guide and description of different interventions

Categories of non-pharmacological intervention	Specific treatments	Code	Definitions of coding an experimental intervention under the corresponding component
**Control**		CO	Active control or usual care
	Active control	AC	A regular exercise program of low intensity or regular programmed health education
	Usual care	UC	Standard/routine care, no treatment, placebo, wait-list control, or sham treatment
**Exercise**		EX	Any physical activity that involves the use of skeletal muscles and requires energy expenditure
	Aerobic training	AT	Exercising with adequate oxygen provision (such as walking, cycling, climbing etc)
	Relaxation training	RT	Systematic progressive muscle relaxation training, which involved practice sessions
	Stretching training	ST	Exercise emphasizes muscle lengthening through static or isometric movements
	Flexibility training	FT	Exercise focuses on the enhancing the range of motion of joint systems through active movements, without including a stretching component
	Stabilization exercise	SE	Exercise that focuses on modifiable intensity level based on the exercise capacities of each participant, including supine, dead bug, side lying, prone, bird dog, bridge, and plank
	Mixed exercise training	ME	A combination of two or more specific treatments of exercise types
**Mind body therapy**		MBT	Interventions that aim at addressing both physical and mental aspects, such as Yoga, Pilates, and Qigong
**Education**		EDU	Interventions that prioritize enhancing awareness and promoting positive attitudes and knowledge pertaining to health enhancement
**Telemedicine**		TM	Internet- or mobile-based interventions, including the application of virtual reality, web-based system, and mobile apps
**Biopsychosocial Approach**		BA	Interventions that aim to modify behavior, emotional state, or feelings, without providing any particular knowledge or information about a specific field

	Cognitive-behavioural treatments	CBT	Enhancing physical performance by enhancing the mental processing and manipulation of information
	Psychosocial intervention	PI	Interventions that integrate psychological and social elements in its framework, such as counselling, problem adaptation therapy, goal setting, feedback, motivational interview, etc)
	Meditation	Medi	Sustained recognition of the knowing quality of awareness itself, which focuses on orientation to the present moment with openness and acceptance
**Physical therapy**		PT	Interventions that aim to improve or recover one's health by utilizing physical factors such as heat, vibration, light, electricity, and orthotic appliances.
	Massage	Mass	A healthcare method that stimulates acupuncture points and regulates bodily functions by applying pressure, kneading, rubbing, and other techniques using the hands, fingers, palms, and elbows, to enhance the physiological state of the body
	Thermal therapy	TT	The therapy that utilizes temperature to improve physical health, by relieving muscle stiffness and pain, reducing inflammation, improving blood circulation and metabolism
	Manipulative treatment	MT	A typical physical therapy intervention that aims to ameliorate or eradicate complications related to muscles, bones, or joints, such as pain, stiffness, inflammation, and related anomalies
	Acupuncture	Acup	A traditional Chinese medicine technique that involves the insertion of thin needles into specific points on the body to balance the flow of energy, or Qi, within the body, and stimulate the body's natural healing processes
	Electrical stimulation	ES	The transcutaneous electrical nerve stimulation that utilizes low frequency electrical impulse currents through the skin to stimulate the peripheral nerves and produce various physiological effects
	Ultrasound therapy	US	The intervention that promotes tissue recovery and pain relief through the acoustic force and thermal effect of ultrasound waves by promoting blood and lymph circulation, allowing the tissues to receive sufficient oxygen and nutrients, thus accelerating tissue repair and recovery
	Kinesiology taping	KT	A physical therapy procedure that utilizes a unique adhesive tape to facilitate the therapy, rehabilitation, and prevention of muscular, joint, and soft tissue disorders
	Leech therapy	LT	A conventional medical procedure that utilizes

			medicinal leeches to extract blood from patients' bodies
	Myofascial induction therapy	MIT	A form of manual therapy that aims to promote healing and reduce pain by enhancing blood flow, reducing inflammation, and breaking down adhesions in the fascial tissue
	Pulsed electromagnetic frequency therapy	PEF	A complementary therapy that delivers short bursts of electrical microamperes, which are millionths of an ampere, to injured tissues without producing heat or interfering with nerve or muscle function
	Perifacet injection therapy	PIT	The intervention that relieves the pain from the source of lumbar facet joints and multifidus muscle
	Extracorporeal shock wave therapy	ESWT	A non-invasive medical treatment that utilizes shock waves to treat an array of musculoskeletal conditions
	Mixed physical therapies	MPT	A combination of two or more specific treatments of physical therapy
**Multicomponent intervention**	Multicomponent intervention	MUI	Interventions that involve a combination of two or more categories mentioned above

Appendix 4. Summary of risk of bias assessment using Risk of bias 2 on included randomized controlled trials

Author	1. Randomization process				2. Deviations from intended interventions								3. Missing outcome data					4. Measurement of the outcome						5. Selection of the reported result				Overall Bias
	1.1	1.2	1.3	RoB	2.1	2.2	2.3	2.4	2.5	2.6	2.7	RoB	3.1	3.2	3.3	3.4	RoB	4.1	4.2	4.3	4.4	4.5	RoB	5.1	5.2	5.3	RoB	RoB
Sığlan 2023	Y	PY	N	Low	N	N	NA	NA	NA	PY	NA	Low	PY	NA	NA	NA	Low	N	PN	N	NA	NA	Low	Y	PN	PN	Low	Low
Sanabria-Mazo 2023	Y	Y	N	Low	PY	PY	PN	NA	NA	PY	NA	Low	PN	NA	NA	NA	Low	PN	PN	PY	PN	NA	Low	PY	NI	NI	Some concerns	Some concerns
Ogunniran 2023	Y	Y	PN	Low	PY	Y	PN	NA	NA	PN	PN	Some concerns	PN	PY	NA	NA	Low	PN	PN	PY	PN	NA	Low	PY	PN	PN	Low	Some concerns
Lazaridou 2023	Y	NI	NI	Some concerns	NI	PY	PN	NA	NA	PY	NA	Low	PN	PN	PY	PN	Some concerns	PN	PN	PY	PN	NA	Low	PY	NI	NI	Some concerns	Some concerns
Kim 2023	Y	Y	PN	Low	PN	PY	PY	PY	PY	NI	PN	Some concerns	PY	NA	NA	NA	Low	PN	PN	Y	PN	NA	Low	PY	PN	PN	Low	Some concerns
Kanaan 2023	Y	PY	PN	Low	PY	PY	PY	PN	NA	PY	NA	Some concerns	PN	PY	NA	NA	Low	PN	PN	PN	NA	NA	Low	PY	PN	PN	Low	Some concerns
Groenveld 2023	Y	PY	PN	Low	PY	PN	PY	PN	NA	PY	NA	Some concerns	PN	PN	PY	PN	Some concerns	PN	PN	PY	PN	NA	Low	PY	PN	PN	Low	Some concerns
Zheng 2022	Y	Y	PN	Low	PY	PN	PY	PN	NA	PY	NA	Some concerns	PY	NA	NA	NA	Low	PN	PN	N	NA	NA	Low	PY	PN	PN	Low	Some concerns
Singphow 2022	Y	Y	PN	Low	PY	PY	PY	PN	NA	PY	NA	Some concerns	PY	NA	NA	NA	Low	PN	PN	N	NA	NA	Low	PY	PN	PN	Low	Some concerns
Shaygan 2022	Y	N	PN	High	Y	Y	PY	PY	PN	PY	NA	High	NI	PN	PY	PY	High	PN	PN	PY	PN	NA	Low	PN	NI	NI	Some concerns	High
Rim 2022	Y	PY	PN	Low	PY	PY	PY	PN	NA	PY	NA	Some concerns	PN	PN	PY	PN	Some concerns	PN	PN	PY	NI	NI	High	PY	NI	NI	Some concerns	High
Lara-Palomo 2022	Y	PY	PN	Low	PY	PN	PN	NA	NA	PY	NA	Low	PN	PY	NA	NA	Low	PN	PN	PN	NA	NA	Low	PY	NI	NI	Some concerns	Some concerns
Kızıltaş 2022	Y	PY	PN	Low	PY	PY	PY	PY	PY	PN	PN	Some concerns	PN	PN	PY	PY	High	PN	PN	PY	NI	NI	High	PY	NI	NI	Some concerns	High
Hrkać 2022	Y	PY	PN	Low	PY	PY	PN	NA	NA	PY	NA	Low	PN	PY	NA	NA	Low	PN	PN	PN	NA	NA	Low	PY	PN	PN	Low	Low
Diez 2022	Y	PY	PN	Low	PY	PY	NI	NA	NA	PY	NA	Some concerns	PY	NA	NA	NA	Low	PN	PN	PN	NA	NA	Low	PY	PN	PN	Low	Some concerns
Aguilar- Ferrándiz 2022	Y	PY	PN	Low	PY	PN	PN	NA	NA	PY	NA	Low	Y	NA	NA	NA	Low	PN	PN	PN	NA	NA	Low	PY	NI	NI	Some concerns	Some concerns
Yakşi 2021	Y	Y	PN	Low	N	PY	PN	NA	NA	PY	NA	Low	PY	NA	NA	NA	Low	PN	PN	PY	NI	NI	High	PY	NI	NI	Some concerns	High
Schmidt 2021	Y	PN	PN	High	PY	PY	PY	PN	NA	PY	NA	Some concerns	PN	PN	PY	NI	High	PN	PN	PY	PN	NA	Low	PY	PN	PN	Low	High

Polaski 2021	Y	PY	PN	Low	PY	PN	PN	NA	NA	PY	NA	Low	PN	PY	NA	NA	Low	PN	PN	PY	PY	PN	Some concerns	PY	NI	NI	Some concerns	Some concerns
Karaarslan 2021	Y	PY	PN	Low	PY	PY	PN	NA	NA	PY	NA	Low	PN	PN	PY	PN	Some concerns	PN	PN	N	NA	NA	Low	PY	NI	NI	Some concerns	Some concerns
Darnall 2021	Y	PY	PN	Low	PY	PN	PN	NA	NA	PY	NA	Low	PN	PY	NA	NA	Low	PN	PN	PN	NA	NA	Low	PY	PN	PN	Low	Low
Batıbay 2021	Y	PY	PN	Low	PY	PY	PN	NA	NA	PY	NA	Low	PY	NA	NA	NA	Low	PN	PN	PY	NI	NI	High	PY	NI	NI	Some concerns	High
Alzahrani 2021	Y	PY	PN	Low	PY	PY	PN	NA	NA	PY	NA	Low	PY	NA	NA	NA	Low	PN	PN	PY	NI	PN	Some concerns	PY	NI	NI	Some concerns	Some concerns
Ünal 2020	Y	PY	PN	Low	PY	PY	Y	PN	NA	PN	PN	Some concerns	PY	NA	NA	NA	Low	PN	PN	PN	NA	NA	Low	PY	NI	NI	Some concerns	Some concerns
Soleymani 2020	Y	PY	PN	Low	PY	PY	PN	NA	NA	PY	NA	Low	PN	PN	PY	PN	Some concerns	PN	PN	PY	PY	PN	Some concerns	PY	NI	NI	Some concerns	Some concerns
Schlicker 2020	Y	Y	PN	Low	Y	PY	PY	PY	PY	Y	NA	Some concerns	Y	NA	NA	NA	Low	PN	PN	PY	PY	PY	High	PY	PN	PN	Low	High
de Oliveira Meirelles 2020	Y	Y	PN	Low	PY	N	PN	NA	NA	PY	NA	Low	PY	NA	NA	NA	Low	PN	PN	N	NA	NA	Low	PY	NI	NI	Some concerns	Some concerns
Suh 2019	Y	PY	PY	Some concerns	Y	PY	PY	PY	PY	Y	NA	Some concerns	PN	PY	NA	NA	Low	PN	PN	PY	PN	NA	Low	PY	NI	NI	Some concerns	Some concerns
Petrozzi 2019	Y	Y	PY	Some concerns	PY	PN	PN	NA	NA	PY	NA	Low	PN	PN	PY	PN	Some concerns	PN	PN	PN	NA	NA	Low	PY	NI	NI	Some concerns	Some concerns
Mariano 2019	Y	NI	PN	Some concerns	PY	PY	PN	NA	NA	PY	NA	Low	PY	NA	NA	NA	Low	PN	PN	PY	PN	NA	Low	PY	NI	NI	Some concerns	Some concerns
Hüppe 2019	Y	PY	PN	Low	PY	PY	PY	PY	PY	PN	PN	Some concerns	PN	PN	PY	PY	High	PN	PN	PY	PN	NA	Low	PN	NI	NI	Some concerns	High
Huber 2019	Y	PY	PN	Low	PY	PY	PY	PY	PY	PY	NA	Some concerns	PY	NA	NA	NA	Low	PN	PN	PY	PY	PY	High	PY	NI	NI	Some concerns	High
Gardner 2019	Y	PY	PN	Low	PY	PN	PN	NA	NA	PY	NA	Low	Y	NA	NA	NA	Low	PN	PN	PN	NA	NA	Low	PY	PN	PN	Low	Low
Kuvacic 2018	Y	PY	PY	Some concerns	PY	PY	PY	PN	NA	PY	NA	Some concerns	PY	NA	NA	NA	Low	PN	PN	PY	PN	NA	Low	PY	NI	NI	Some concerns	Some concerns
Hohmann 2018	Y	PY	PY	Some concerns	Y	Y	PY	PN	NA	PN	PY	High	PN	PN	PY	NI	High	PN	PN	Y	PY	PN	Some concerns	PY	PY	PY	High	High
Glombiewski 2018	Y	PY	PY	Some concerns	Y	PN	NI	NA	NA	PY	NA	Some concerns	PY	NA	NA	NA	Low	PN	PN	PN	NA	NA	Low	PY	NI	NI	Some concerns	Some concerns
Tüzün 2017	Y	PY	PY	Some concerns	PY	PN	PN	NA	NA	PY	NA	Low	Y	NA	NA	NA	Low	PN	PN	PY	PN	NA	Low	PY	NI	NI	Some concerns	Some concerns
Seo 2017	Y	PY	PN	Low	PY	PN	PN	NA	NA	Y	NA	Low	PY	NA	NA	NA	Low	PN	PN	PN	NA	NA	Low	PY	PN	PN	Low	Low
Nayback-Beebe 2017	Y	PY	PY	Some concerns	Y	Y	PY	PY	PY	PY	NA	Some concerns	NI	PY	NA	NA	Low	PN	PN	Y	PY	PY	High	PY	NI	NI	Some concerns	High
Kumar 2017	Y	PY	PN	Low	PY	PY	PN	NA	NA	PY	NA	Low	PN	PY	NA	NA	Low	PN	PN	PY	PY	PN	Some concerns	PY	NI	NI	Some concerns	Some concerns

Harris 2017	Y	PY	PN	Low	PY	PN	PY	PN	NA	PY	NA	Some concerns	PN	PN	PY	PN	Some concerns	PN	PN	PY	PY	PY	High	PY	NI	NI	Some concerns	High
Michalsen 2016	Y	Y	PN	Low	Y	PY	PY	PY	PY	PY	NA	Some concerns	PN	PN	PY	PN	Some concerns	PN	PN	PY	PY	PN	Some concerns	PY	PN	PN	Low	Some concerns
Trapp 2015	Y	PY	PY	Some concerns	PY	PY	PY	PY	PN	PY	NA	High	PY	NA	NA	NA	Low	PN	PN	PY	NI	NI	High	PY	PN	PN	Low	High
Lawand 2015	Y	PY	PN	Low	PY	PN	PN	NA	NA	PY	NA	Low	PY	NA	NA	NA	Low	PN	PN	PN	NA	NA	Low	PY	PN	PN	Low	Low
Kogure 2015	Y	PY	PN	Low	N	Y	PN	NA	NA	PY	NA	Low	PN	PY	NA	NA	Low	PN	PN	PY	PY	PN	Some concerns	PY	NI	NI	Some concerns	Some concerns
Zhang 2014	Y	Y	PN	Low	PY	PY	PY	PY	PY	PY	NA	Some concerns	PY	NA	NA	NA	Low	PN	PN	PY	PY	PY	High	PY	PY	PY	High	High
Monticone 2014	Y	PY	PN	Low	Y	Y	PY	PY	PY	PY	NA	Some concerns	PY	NA	NA	NA	Low	PN	PN	Y	PY	PN	Some concerns	Y	NI	NI	Some concerns	Some concerns
Tekur 2012	Y	PY	PN	Low	PY	N	PN	NA	NA	PY	NA	Low	Y	NA	NA	NA	Low	PN	PN	N	NA	NA	Low	PY	PN	PN	Low	Low
Kader 2012	Y	PY	PY	Some concerns	PY	PY	PY	PY	PN	PY	NA	High	PN	PN	PY	PN	Some concerns	PN	PN	PY	PY	PY	High	PY	NI	NI	Some concerns	High
Cuesta-Vargas 2012	Y	PY	PN	Low	PY	PY	PN	NA	NA	PY	NA	Low	PY	NA	NA	NA	Low	PN	PN	PY	PN	NA	Low	PY	PN	PN	Low	Low
Tavafian 2011	Y	Y	N	Low	Y	N	PN	NA	NA	PY	NA	Low	PN	PY	NA	NA	Low	PN	PN	N	NA	NA	Low	PY	NI	NI	Some concerns	Some concerns
Engbert 2011	Y	PY	PN	Low	PY	PN	PN	NA	NA	PY	NA	Low	PN	PY	NA	NA	Low	PN	PN	PY	PN	NA	Low	PY	PN	PN	Low	Low
Glombiewski 2010	Y	PY	PN	Low	PY	PY	PY	PN	NA	PY	NA	Some concerns	PY	NA	NA	NA	Low	PN	PN	PY	PY	PN	Some concerns	PY	NI	NI	Some concerns	Some concerns
Durmus 2010	PY	PY	PN	Low	PY	PY	PY	PY	PY	PY	NA	Some concerns	PN	PN	PN	NA	Low	PN	PN	PY	PY	PY	High	PY	PY	PY	High	High
Williams 2009	Y	PY	PN	Low	PY	PY	PY	PY	PY	PY	NA	Some concerns	PN	PN	PY	PN	Some concerns	PN	PN	PY	PY	PN	Some concerns	PY	PN	PN	Low	Some concerns
Sertpoyraz 2009	PY	PY	PN	Low	PY	PY	PY	PY	PY	PN	PY	High	Y	NA	NA	NA	Low	PN	PN	PY	PY	PY	High	PY	NI	NI	Some concerns	High
Ribeiro 2008	PY	PY	PY	Some concerns	Y	PN	PY	PY	PY	PY	NA	Some concerns	PN	PY	NA	NA	Low	PN	PN	PY	PN	NA	Low	PY	NI	NI	Some concerns	Some concerns
Koldaş Doğan 2008	Y	PY	PN	Low	PY	PY	PY	PY	PY	PY	NA	Some concerns	PY	NA	NA	NA	Low	PN	PN	PY	PY	PN	Some concerns	PY	NI	NI	Some concerns	Some concerns
Tavafian 2007	Y	PY	PN	Low	Y	PY	PY	PY	PY	PY	NA	Some concerns	PY	NA	NA	NA	Low	PN	PN	PY	PY	PN	Some concerns	PY	PN	PN	Low	Some concerns
Kaapa 2006	Y	PY	NI	Low	PY	PN	PN	NA	NA	PY	NA	Low	PN	PY	NA	NA	Low	PN	PN	NI	PN	NA	Low	PY	PN	PN	Low	Low
Galantino 2004	Y	Y	PN	Low	PY	PY	PY	PY	PY	PY	NA	Some concerns	PN	PY	NA	NA	Low	PN	PN	PY	PY	PN	Some concerns	PY	NI	NI	Some concerns	Some concerns
Weiner 2003	Y	PY	PN	Low	PY	PY	PY	PN	NA	PY	NA	Some concerns	PY	NA	NA	NA	Low	PN	PN	PY	PN	NA	Low	PY	PN	PN	Low	Some concerns
Niemisto 2003	Y	PY	PN	Low	PY	PY	PY	PY	PY	PY	NA	Some concerns	PN	PN	PY	PN	Some concerns	PN	PY	NA	NA	NA	High	PY	NI	NI	Some concerns	High

Hernandez-Reif 2001	Y	NI	PN	Some concerns	PY	PY	PY	PN	NA	PY	NA	Some concerns	PY	NA	NA	NA	Low	PN	PN	PY	PN	NA	Low	PY	NI	NI	Some concerns	Some concerns
Newton-John 1995	PY	PY	NI	Low	PY	PY	PY	PY	PY	PN	PN	Some concerns	PN	PN	PY	PN	Some concerns	PN	PN	PY	PN	NA	Low	PY	NI	NI	Some concerns	Some concerns
Turner 1993	PY	PY	PY	Some concerns	PY	PY	PN	NA	NA	PY	NA	Low	PN	PN	Y	PY	High	PN	PN	PY	PY	PN	Some concerns	PY	NI	NI	Some concerns	High

Notes: RoB, risk of bias; High, High risk of bias; Low, Low risk of bias; NI, no information; NA, not applicable; N, No; PN, Probably No; PY, Probably Yes; Y, Yes.

Appendix 5. Detailed results of each domain on risk of bias assessment using Risk of bias 2 on included randomized controlled trials

Author	D1	D2	D3	D4	D5	Overall
Sığlan 2023	+	+	+	+	+	+
Sanabria-Mazo 2023	+	+	+	+	!	!
Ogunniran 2023	+	!	+	+	+	!
Lazaridou 2023	!	+	!	+	!	!
Kim 2023	+	!	+	+	+	!
Kanaan 2023	+	!	+	+	+	!
Groenveld 2023	+	!	!	+	+	!
Zheng 2022	+	!	+	+	+	!
Singphow 2022	+	!	+	+	+	!
Shaygan 2022	-	-	-	+	!	-
Rim 2022	+	!	!	-	!	-
Lara-Palomo 2022	+	+	+	+	!	!
Kızıltaş 2022	+	!	-	-	!	-
Hrkać 2022	+	+	+	+	+	+
Diez 2022	+	!	+	+	+	!
Aguilar-Ferrándiz 2022	+	+	+	+	!	!
Yakşi 2021	+	+	+	-	!	-
Schmidt 2021	-	!	-	+	+	-
Polaski 2021	+	+	+	!	!	!
Karaarslan 2021	+	+	!	+	!	!
Darnall 2021	+	+	+	+	+	+
Batıbay 2021	+	+	+	-	!	-
Alzahrani 2021	+	+	+	!	!	!
Ünal 2020	+	!	+	+	!	!
Soleymani 2020	+	+	!	!	!	!
Schlicker 2020	+	!	+	-	+	-
de Oliveira Meirelles 2020	+	+	+	+	!	!
Suh 2019	!	!	+	+	!	!

**Petrozzi 2019**	!	+	!	+	!	!
**Mariano 2019**	!	+	+	+	!	!
**Hüppe 2019**	+	!	-	+	!	-
**Huber 2019**	+	!	+	-	!	-
**Gardner 2019**	+	+	+	+	+	+
**Kuvacic 2018**	!	!	+	+	!	!
**Hohmann 2018**	!	-	-	!	-	-
**Glombiewski 2018**	!	!	+	+	!	!
**Tüzün 2017**	!	+	+	+	!	!
**Seo 2017**	+	+	+	+	+	+
**Nayback-Beebe 2017**	!	!	+	-	!	-
**Kumar 2017**	+	+	+	!	!	!
**Harris 2017**	+	!	!	-	!	-
**Michalsen 2016**	+	!	!	!	+	!
**Trapp 2015**	!	-	+	-	+	-
**Lawand 2015**	+	+	+	+	+	+
**Kogure 2015**	+	+	+	!	!	!
**Zhang 2014**	+	!	+	-	-	-
**Monticone 2014**	+	!	+	!	!	!
**Tekur 2012**	+	+	+	+	+	+
**Kader 2012**	!	-	!	-	!	-
**Cuesta-Vargas 2012**	+	+	+	+	+	+
**Tavafian 2011**	+	+	+	+	!	!
**Engbert 2011**	+	+	+	+	+	+
**Glombiewski 2010**	+	!	+	!	!	!
**Durmus 2010**	+	!	+	-	-	-
**Williams 2009**	+	!	!	!	+	!
**Sertpoyraz 2009**	+	-	+	-	!	-
**Ribeiro 2008**	!	!	+	+	!	!
**Koldaş Doğan 2008**	+	!	+	!	!	!

**Tavafian 2007**	+	!	+	!	+	!
**Kaapa 2006**	+	+	+	+	+	+
**Galantino 2004**	+	!	+	!	!	!
**Weiner 2003**	+	!	+	+	+	!
**Niemisto 2003**	+	!	!	-	!	-
**Hernandez-Reif 2001**	!	!	+	+	!	!
**Newton-John 1995**	+	!	!	+	!	!
**Turner 1993**	!	+	-	!	!	-

+ **Low risk**

! **Some concerns**

- **High risk**

D1 **Randomisation process**

D2 **Deviations from the intended interventions**

D3 **Missing outcome data**

D4 **Measurement of the outcome**

D5 **Selection of the reported result**

Appendix 6. Detailed information of each trial for the risk of bias assessment

Study	Sığlan 2023	
**Domain**	**Signalling question**	**Response**
**Bias arising from the randomization process**	1.1 Was the allocation sequence random?	Y
	1.2 Was the allocation sequence concealed until participants were enrolled and assigned to interventions?	PY
	1.3 Did baseline differences between intervention groups suggest a problem with the randomization process?	N
	**Risk of bias judgement**	**Low**
**Bias due to deviations from intended interventions**	2.1.Were participants aware of their assigned intervention during the trial?	N
	2.2.Were carers and people delivering the interventions aware of participants' assigned intervention during the trial?	N
	2.3. If Y/PY/NI to 2.1 or 2.2: Were there deviations from the intended intervention that arose because of the experimental context?	NA
	2.4 If Y/PY to 2.3: Were these deviations likely to have affected the outcome?	NA
	2.5. If Y/PY/NI to 2.4: Were these deviations from intended intervention balanced between groups?	NA
	2.6 Was an appropriate analysis used to estimate the effect of assignment to intervention?	PY
	2.7 If N/PN/NI to 2.6: Was there potential for a substantial impact (on the result) of the failure to analyse participants in the group to which they were randomized?	NA
	**Risk of bias judgement**	**Low**
**Bias due to missing outcome data**	3.1 Were data for this outcome available for all, or nearly all, participants randomized?	PY
	3.2 If N/PN/NI to 3.1: Is there evidence that result was not biased by missing outcome data?	NA
	3.3 If N/PN to 3.2: Could missingness in the outcome depend on its true value?	NA
	3.4 If Y/PY/NI to 3.3: Is it likely that missingness in the outcome depended on its true value?	NA
	**Risk of bias judgement**	**Low**
**Bias in measurement of the outcome**	4.1 Was the method of measuring the outcome inappropriate?	N
	4.2 Could measurement or ascertainment of the outcome have differed between intervention groups?	PN
	4.3 Were outcome assessors aware of the intervention received by study participants?	N
	4.4 If Y/PY/NI to 4.3: Could assessment of the outcome have been influenced by knowledge of intervention received?	NA
	4.5 If Y/PY/NI to 4.4: Is it likely that assessment of the outcome was influenced by knowledge of intervention received?	NA
	**Risk of bias judgement**	**Low**
**Bias in selection of the reported result**	5.1 Were the data that produced this result analysed in accordance with a pre-specified analysis plan that was finalized before unblinded outcome data were available for analysis?	Y
	5.2 ... multiple eligible outcome measurements (e.g. scales, definitions, time points) within the outcome domain?	PN
	5.3 ... multiple eligible analyses of the data?	PN
	**Risk of bias judgement**	**Low**
**Overall bias**	**Risk of bias judgement**	**Low**

Study	Sanabria-Mazo 2023	
**Domain**	**Signalling question**	**Response**
**Bias arising from the randomization process**	1.1 Was the allocation sequence random?	Y
	1.2 Was the allocation sequence concealed until participants were enrolled and assigned to interventions?	Y
	1.3 Did baseline differences between intervention groups suggest a problem with the randomization process?	N
	**Risk of bias judgement**	**Low**
**Bias due to deviations from intended interventions**	2.1.Were participants aware of their assigned intervention during the trial?	PY
	2.2.Were carers and people delivering the interventions aware of participants' assigned intervention during the trial?	PY
	2.3. If Y/PY/NI to 2.1 or 2.2: Were there deviations from the intended intervention that arose because of the experimental context?	PN
	2.4 If Y/PY to 2.3: Were these deviations likely to have affected the outcome?	NA
	2.5. If Y/PY/NI to 2.4: Were these deviations from intended intervention balanced between groups?	NA
	2.6 Was an appropriate analysis used to estimate the effect of assignment to intervention?	PY
	2.7 If N/PN/NI to 2.6: Was there potential for a substantial impact (on the result) of the failure to analyse participants in the group to which they were randomized?	NA
	**Risk of bias judgement**	**Low**
**Bias due to missing outcome data**	3.1 Were data for this outcome available for all, or nearly all, participants randomized?	PY
	3.2 If N/PN/NI to 3.1: Is there evidence that result was not biased by missing outcome data?	NA
	3.3 If N/PN to 3.2: Could missingness in the outcome depend on its true value?	NA
	3.4 If Y/PY/NI to 3.3: Is it likely that missingness in the outcome depended on its true value?	NA
	**Risk of bias judgement**	**Low**
**Bias in measurement of the outcome**	4.1 Was the method of measuring the outcome inappropriate?	PN
	4.2 Could measurement or ascertainment of the outcome have differed between intervention groups?	PN
	4.3 Were outcome assessors aware of the intervention received by study participants?	PY
	4.4 If Y/PY/NI to 4.3: Could assessment of the outcome have been influenced by knowledge of intervention received?	PN
	4.5 If Y/PY/NI to 4.4: Is it likely that assessment of the outcome was influenced by knowledge of intervention received?	NA
	**Risk of bias judgement**	**Low**
**Bias in selection of the reported result**	5.1 Were the data that produced this result analysed in accordance with a pre-specified analysis plan that was finalized before unblinded outcome data were available for analysis?	PY
	5.2 ... multiple eligible outcome measurements (e.g. scales, definitions, time points) within the outcome domain?	NI
	5.3 ... multiple eligible analyses of the data?	NI
	**Risk of bias judgement**	**Some concerns**
**Overall bias**	**Risk of bias judgement**	**Some concerns**

Study	Ogunniran 2023	
**Domain**	**Signalling question**	**Response**
**Bias arising from the randomization process**	1.1 Was the allocation sequence random?	Y
	1.2 Was the allocation sequence concealed until participants were enrolled and assigned to interventions?	Y
	1.3 Did baseline differences between intervention groups suggest a problem with the randomization process?	PN
	**Risk of bias judgement**	**Low**
**Bias due to deviations from intended interventions**	2.1.Were participants aware of their assigned intervention during the trial?	PY
	2.2.Were carers and people delivering the interventions aware of participants' assigned intervention during the trial?	Y
	2.3. If Y/PY/NI to 2.1 or 2.2: Were there deviations from the intended intervention that arose because of the experimental context?	PN
	2.4 If Y/PY to 2.3: Were these deviations likely to have affected the outcome?	NA
	2.5. If Y/PY/NI to 2.4: Were these deviations from intended intervention balanced between groups?	NA
	2.6 Was an appropriate analysis used to estimate the effect of assignment to intervention?	PN
	2.7 If N/PN/NI to 2.6: Was there potential for a substantial impact (on the result) of the failure to analyse participants in the group to which they were randomized?	PN
	**Risk of bias judgement**	**Some concerns**
**Bias due to missing outcome data**	3.1 Were data for this outcome available for all, or nearly all, participants randomized?	PN
	3.2 If N/PN/NI to 3.1: Is there evidence that result was not biased by missing outcome data?	PY
	3.3 If N/PN to 3.2: Could missingness in the outcome depend on its true value?	NA
	3.4 If Y/PY/NI to 3.3: Is it likely that missingness in the outcome depended on its true value?	NA
	**Risk of bias judgement**	**Low**
**Bias in measurement of the outcome**	4.1 Was the method of measuring the outcome inappropriate?	PN
	4.2 Could measurement or ascertainment of the outcome have differed between intervention groups?	PN
	4.3 Were outcome assessors aware of the intervention received by study participants?	PY
	4.4 If Y/PY/NI to 4.3: Could assessment of the outcome have been influenced by knowledge of intervention received?	PN
	4.5 If Y/PY/NI to 4.4: Is it likely that assessment of the outcome was influenced by knowledge of intervention received?	NA
	**Risk of bias judgement**	**Low**
**Bias in selection of the reported result**	5.1 Were the data that produced this result analysed in accordance with a pre-specified analysis plan that was finalized before unblinded outcome data were available for analysis?	PY
	5.2 ... multiple eligible outcome measurements (e.g. scales, definitions, time points) within the outcome domain?	PN
	5.3 ... multiple eligible analyses of the data?	PN
	**Risk of bias judgement**	**Low**
**Overall bias**	**Risk of bias judgement**	**Some concerns**

Study	Lazaridou 2023	
**Domain**	**Signalling question**	**Response**
**Bias arising from the randomization process**	1.1 Was the allocation sequence random?	Y
	1.2 Was the allocation sequence concealed until participants were enrolled and assigned to interventions?	NI
	1.3 Did baseline differences between intervention groups suggest a problem with the randomization process?	NI
	**Risk of bias judgement**	**Some concerns**
**Bias due to deviations from intended interventions**	2.1.Were participants aware of their assigned intervention during the trial?	NI
	2.2.Were carers and people delivering the interventions aware of participants' assigned intervention during the trial?	PY
	2.3. If Y/PY/NI to 2.1 or 2.2: Were there deviations from the intended intervention that arose because of the experimental context?	PN
	2.4 If Y/PY to 2.3: Were these deviations likely to have affected the outcome?	NA
	2.5. If Y/PY/NI to 2.4: Were these deviations from intended intervention balanced between groups?	NA
	2.6 Was an appropriate analysis used to estimate the effect of assignment to intervention?	PY
	2.7 If N/PN/NI to 2.6: Was there potential for a substantial impact (on the result) of the failure to analyse participants in the group to which they were randomized?	NA
	**Risk of bias judgement**	**Low**
**Bias due to missing outcome data**	3.1 Were data for this outcome available for all, or nearly all, participants randomized?	PN
	3.2 If N/PN/NI to 3.1: Is there evidence that result was not biased by missing outcome data?	PN
	3.3 If N/PN to 3.2: Could missingness in the outcome depend on its true value?	PY
	3.4 If Y/PY/NI to 3.3: Is it likely that missingness in the outcome depended on its true value?	PN
	**Risk of bias judgement**	**Some concerns**
**Bias in measurement of the outcome**	4.1 Was the method of measuring the outcome inappropriate?	PN
	4.2 Could measurement or ascertainment of the outcome have differed between intervention groups?	PN
	4.3 Were outcome assessors aware of the intervention received by study participants?	PY
	4.4 If Y/PY/NI to 4.3: Could assessment of the outcome have been influenced by knowledge of intervention received?	PN
	4.5 If Y/PY/NI to 4.4: Is it likely that assessment of the outcome was influenced by knowledge of intervention received?	NA
	**Risk of bias judgement**	**Low**
**Bias in selection of the reported result**	5.1 Were the data that produced this result analysed in accordance with a pre-specified analysis plan that was finalized before unblinded outcome data were available for analysis?	PY
	5.2 ... multiple eligible outcome measurements (e.g. scales, definitions, time points) within the outcome domain?	NI
	5.3 ... multiple eligible analyses of the data?	NI
	**Risk of bias judgement**	**Some concerns**
**Overall bias**	**Risk of bias judgement**	**Some concerns**

Study	Kim 2023	
**Domain**	**Signalling question**	**Response**
**Bias arising from the randomization process**	1.1 Was the allocation sequence random?	Y
	1.2 Was the allocation sequence concealed until participants were enrolled and assigned to interventions?	Y
	1.3 Did baseline differences between intervention groups suggest a problem with the randomization process?	PN
	**Risk of bias judgement**	**Low**
**Bias due to deviations from intended interventions**	2.1.Were participants aware of their assigned intervention during the trial?	PN
	2.2.Were carers and people delivering the interventions aware of participants' assigned intervention during the trial?	PY
	2.3. If Y/PY/NI to 2.1 or 2.2: Were there deviations from the intended intervention that arose because of the experimental context?	PY
	2.4 If Y/PY to 2.3: Were these deviations likely to have affected the outcome?	PY
	2.5. If Y/PY/NI to 2.4: Were these deviations from intended intervention balanced between groups?	PY
	2.6 Was an appropriate analysis used to estimate the effect of assignment to intervention?	NI
	2.7 If N/PN/NI to 2.6: Was there potential for a substantial impact (on the result) of the failure to analyse participants in the group to which they were randomized?	PN
	**Risk of bias judgement**	**Some concerns**
**Bias due to missing outcome data**	3.1 Were data for this outcome available for all, or nearly all, participants randomized?	PY
	3.2 If N/PN/NI to 3.1: Is there evidence that result was not biased by missing outcome data?	NA
	3.3 If N/PN to 3.2: Could missingness in the outcome depend on its true value?	NA
	3.4 If Y/PY/NI to 3.3: Is it likely that missingness in the outcome depended on its true value?	NA
	**Risk of bias judgement**	**Low**
**Bias in measurement of the outcome**	4.1 Was the method of measuring the outcome inappropriate?	PN
	4.2 Could measurement or ascertainment of the outcome have differed between intervention groups?	PN
	4.3 Were outcome assessors aware of the intervention received by study participants?	Y
	4.4 If Y/PY/NI to 4.3: Could assessment of the outcome have been influenced by knowledge of intervention received?	PN
	4.5 If Y/PY/NI to 4.4: Is it likely that assessment of the outcome was influenced by knowledge of intervention received?	NA
	**Risk of bias judgement**	**Low**
**Bias in selection of the reported result**	5.1 Were the data that produced this result analysed in accordance with a pre-specified analysis plan that was finalized before unblinded outcome data were available for analysis?	PY
	5.2 ... multiple eligible outcome measurements (e.g. scales, definitions, time points) within the outcome domain?	PN
	5.3 ... multiple eligible analyses of the data?	PN
	**Risk of bias judgement**	**Low**
**Overall bias**	**Risk of bias judgement**	**Some concerns**

Study	Kanaan 2023	
**Domain**	**Signalling question**	**Response**
**Bias arising from the randomization process**	1.1 Was the allocation sequence random?	Y
	1.2 Was the allocation sequence concealed until participants were enrolled and assigned to interventions?	PY
	1.3 Did baseline differences between intervention groups suggest a problem with the randomization process?	PN
	**Risk of bias judgement**	**Low**
**Bias due to deviations from intended interventions**	2.1.Were participants aware of their assigned intervention during the trial?	PY
	2.2.Were carers and people delivering the interventions aware of participants' assigned intervention during the trial?	PY
	2.3. If Y/PY/NI to 2.1 or 2.2: Were there deviations from the intended intervention that arose because of the experimental context?	PY
	2.4 If Y/PY to 2.3: Were these deviations likely to have affected the outcome?	PN
	2.5. If Y/PY/NI to 2.4: Were these deviations from intended intervention balanced between groups?	NA
	2.6 Was an appropriate analysis used to estimate the effect of assignment to intervention?	PY
	2.7 If N/PN/NI to 2.6: Was there potential for a substantial impact (on the result) of the failure to analyse participants in the group to which they were randomized?	NA
	**Risk of bias judgement**	**Some concerns**
**Bias due to missing outcome data**	3.1 Were data for this outcome available for all, or nearly all, participants randomized?	PN
	3.2 If N/PN/NI to 3.1: Is there evidence that result was not biased by missing outcome data?	PY
	3.3 If N/PN to 3.2: Could missingness in the outcome depend on its true value?	NA
	3.4 If Y/PY/NI to 3.3: Is it likely that missingness in the outcome depended on its true value?	NA
	**Risk of bias judgement**	**Low**
**Bias in measurement of the outcome**	4.1 Was the method of measuring the outcome inappropriate?	PN
	4.2 Could measurement or ascertainment of the outcome have differed between intervention groups?	PN
	4.3 Were outcome assessors aware of the intervention received by study participants?	PN
	4.4 If Y/PY/NI to 4.3: Could assessment of the outcome have been influenced by knowledge of intervention received?	NA
	4.5 If Y/PY/NI to 4.4: Is it likely that assessment of the outcome was influenced by knowledge of intervention received?	NA
	**Risk of bias judgement**	**Low**
**Bias in selection of the reported result**	5.1 Were the data that produced this result analysed in accordance with a pre-specified analysis plan that was finalized before unblinded outcome data were available for analysis?	PY
	5.2 ... multiple eligible outcome measurements (e.g. scales, definitions, time points) within the outcome domain?	PN
	5.3 ... multiple eligible analyses of the data?	PN
	**Risk of bias judgement**	**Low**
**Overall bias**	**Risk of bias judgement**	**Some concerns**

Study	Groenveld 2023	
**Domain**	**Signalling question**	**Response**
**Bias arising from the randomization process**	1.1 Was the allocation sequence random?	Y
	1.2 Was the allocation sequence concealed until participants were enrolled and assigned to interventions?	PY
	1.3 Did baseline differences between intervention groups suggest a problem with the randomization process?	PN
	**Risk of bias judgement**	**Low**
**Bias due to deviations from intended interventions**	2.1.Were participants aware of their assigned intervention during the trial?	PY
	2.2.Were carers and people delivering the interventions aware of participants' assigned intervention during the trial?	PN
	2.3. If Y/PY/NI to 2.1 or 2.2: Were there deviations from the intended intervention that arose because of the experimental context?	PY
	2.4 If Y/PY to 2.3: Were these deviations likely to have affected the outcome?	PN
	2.5. If Y/PY/NI to 2.4: Were these deviations from intended intervention balanced between groups?	NA
	2.6 Was an appropriate analysis used to estimate the effect of assignment to intervention?	PY
	2.7 If N/PN/NI to 2.6: Was there potential for a substantial impact (on the result) of the failure to analyse participants in the group to which they were randomized?	NA
	**Risk of bias judgement**	**Some concerns**
**Bias due to missing outcome data**	3.1 Were data for this outcome available for all, or nearly all, participants randomized?	PN
	3.2 If N/PN/NI to 3.1: Is there evidence that result was not biased by missing outcome data?	PN
	3.3 If N/PN to 3.2: Could missingness in the outcome depend on its true value?	PY
	3.4 If Y/PY/NI to 3.3: Is it likely that missingness in the outcome depended on its true value?	PN
	**Risk of bias judgement**	**Some concerns**
**Bias in measurement of the outcome**	4.1 Was the method of measuring the outcome inappropriate?	PN
	4.2 Could measurement or ascertainment of the outcome have differed between intervention groups?	PN
	4.3 Were outcome assessors aware of the intervention received by study participants?	PY
	4.4 If Y/PY/NI to 4.3: Could assessment of the outcome have been influenced by knowledge of intervention received?	PN
	4.5 If Y/PY/NI to 4.4: Is it likely that assessment of the outcome was influenced by knowledge of intervention received?	NA
	**Risk of bias judgement**	**Low**
**Bias in selection of the reported result**	5.1 Were the data that produced this result analysed in accordance with a pre-specified analysis plan that was finalized before unblinded outcome data were available for analysis?	PY
	5.2 ... multiple eligible outcome measurements (e.g. scales, definitions, time points) within the outcome domain?	PN
	5.3 ... multiple eligible analyses of the data?	PN
	**Risk of bias judgement**	**Low**
**Overall bias**	**Risk of bias judgement**	**Some concerns**

Study	Zheng 2022	
**Domain**	**Signalling question**	**Response**
**Bias arising from the randomization process**	1.1 Was the allocation sequence random?	Y
	1.2 Was the allocation sequence concealed until participants were enrolled and assigned to interventions?	Y
	1.3 Did baseline differences between intervention groups suggest a problem with the randomization process?	PN
	**Risk of bias judgement**	**Low**
**Bias due to deviations from intended interventions**	2.1.Were participants aware of their assigned intervention during the trial?	PY
	2.2.Were carers and people delivering the interventions aware of participants' assigned intervention during the trial?	PN
	2.3. If Y/PY/NI to 2.1 or 2.2: Were there deviations from the intended intervention that arose because of the experimental context?	PY
	2.4 If Y/PY to 2.3: Were these deviations likely to have affected the outcome?	PN
	2.5. If Y/PY/NI to 2.4: Were these deviations from intended intervention balanced between groups?	NA
	2.6 Was an appropriate analysis used to estimate the effect of assignment to intervention?	PY
	2.7 If N/PN/NI to 2.6: Was there potential for a substantial impact (on the result) of the failure to analyse participants in the group to which they were randomized?	NA
	**Risk of bias judgement**	**Some concerns**
**Bias due to missing outcome data**	3.1 Were data for this outcome available for all, or nearly all, participants randomized?	PY
	3.2 If N/PN/NI to 3.1: Is there evidence that result was not biased by missing outcome data?	NA
	3.3 If N/PN to 3.2: Could missingness in the outcome depend on its true value?	NA
	3.4 If Y/PY/NI to 3.3: Is it likely that missingness in the outcome depended on its true value?	NA
	**Risk of bias judgement**	**Low**
**Bias in measurement of the outcome**	4.1 Was the method of measuring the outcome inappropriate?	PN
	4.2 Could measurement or ascertainment of the outcome have differed between intervention groups?	PN
	4.3 Were outcome assessors aware of the intervention received by study participants?	N
	4.4 If Y/PY/NI to 4.3: Could assessment of the outcome have been influenced by knowledge of intervention received?	NA
	4.5 If Y/PY/NI to 4.4: Is it likely that assessment of the outcome was influenced by knowledge of intervention received?	NA
	**Risk of bias judgement**	**Low**
**Bias in selection of the reported result**	5.1 Were the data that produced this result analysed in accordance with a pre-specified analysis plan that was finalized before unblinded outcome data were available for analysis?	PY
	5.2 ... multiple eligible outcome measurements (e.g. scales, definitions, time points) within the outcome domain?	PN
	5.3 ... multiple eligible analyses of the data?	PN
	**Risk of bias judgement**	**Low**
**Overall bias**	**Risk of bias judgement**	**Some concerns**

Study	Singphow 2022	
**Domain**	**Signalling question**	**Response**
**Bias arising from the randomization process**	1.1 Was the allocation sequence random?	Y
	1.2 Was the allocation sequence concealed until participants were enrolled and assigned to interventions?	Y
	1.3 Did baseline differences between intervention groups suggest a problem with the randomization process?	PN
	**Risk of bias judgement**	**Low**
**Bias due to deviations from intended interventions**	2.1.Were participants aware of their assigned intervention during the trial?	PY
	2.2.Were carers and people delivering the interventions aware of participants' assigned intervention during the trial?	PY
	2.3. If Y/PY/NI to 2.1 or 2.2: Were there deviations from the intended intervention that arose because of the experimental context?	PY
	2.4 If Y/PY to 2.3: Were these deviations likely to have affected the outcome?	PN
	2.5. If Y/PY/NI to 2.4: Were these deviations from intended intervention balanced between groups?	NA
	2.6 Was an appropriate analysis used to estimate the effect of assignment to intervention?	PY
	2.7 If N/PN/NI to 2.6: Was there potential for a substantial impact (on the result) of the failure to analyse participants in the group to which they were randomized?	NA
	**Risk of bias judgement**	**Some concerns**
**Bias due to missing outcome data**	3.1 Were data for this outcome available for all, or nearly all, participants randomized?	PY
	3.2 If N/PN/NI to 3.1: Is there evidence that result was not biased by missing outcome data?	NA
	3.3 If N/PN to 3.2: Could missingness in the outcome depend on its true value?	NA
	3.4 If Y/PY/NI to 3.3: Is it likely that missingness in the outcome depended on its true value?	NA
	**Risk of bias judgement**	**Low**
**Bias in measurement of the outcome**	4.1 Was the method of measuring the outcome inappropriate?	PN
	4.2 Could measurement or ascertainment of the outcome have differed between intervention groups?	PN
	4.3 Were outcome assessors aware of the intervention received by study participants?	N
	4.4 If Y/PY/NI to 4.3: Could assessment of the outcome have been influenced by knowledge of intervention received?	NA
	4.5 If Y/PY/NI to 4.4: Is it likely that assessment of the outcome was influenced by knowledge of intervention received?	NA
	**Risk of bias judgement**	**Low**
**Bias in selection of the reported result**	5.1 Were the data that produced this result analysed in accordance with a pre-specified analysis plan that was finalized before unblinded outcome data were available for analysis?	PY
	5.2 ... multiple eligible outcome measurements (e.g. scales, definitions, time points) within the outcome domain?	PN
	5.3 ... multiple eligible analyses of the data?	PN
	**Risk of bias judgement**	**Low**
**Overall bias**	**Risk of bias judgement**	**Some concerns**

Study	Shaygan 2022	
**Domain**	**Signalling question**	**Response**
**Bias arising from the randomization process**	1.1 Was the allocation sequence random?	Y
	1.2 Was the allocation sequence concealed until participants were enrolled and assigned to interventions?	N
	1.3 Did baseline differences between intervention groups suggest a problem with the randomization process?	PN
	**Risk of bias judgement**	**High**
**Bias due to deviations from intended interventions**	2.1.Were participants aware of their assigned intervention during the trial?	Y
	2.2.Were carers and people delivering the interventions aware of participants' assigned intervention during the trial?	Y
	2.3. If Y/PY/NI to 2.1 or 2.2: Were there deviations from the intended intervention that arose because of the experimental context?	PY
	2.4 If Y/PY to 2.3: Were these deviations likely to have affected the outcome?	PY
	2.5. If Y/PY/NI to 2.4: Were these deviations from intended intervention balanced between groups?	PN
	2.6 Was an appropriate analysis used to estimate the effect of assignment to intervention?	PY
	2.7 If N/PN/NI to 2.6: Was there potential for a substantial impact (on the result) of the failure to analyse participants in the group to which they were randomized?	NA
	**Risk of bias judgement**	**High**
**Bias due to missing outcome data**	3.1 Were data for this outcome available for all, or nearly all, participants randomized?	NI
	3.2 If N/PN/NI to 3.1: Is there evidence that result was not biased by missing outcome data?	PN
	3.3 If N/PN to 3.2: Could missingness in the outcome depend on its true value?	PY
	3.4 If Y/PY/NI to 3.3: Is it likely that missingness in the outcome depended on its true value?	PY
	**Risk of bias judgement**	**High**
**Bias in measurement of the outcome**	4.1 Was the method of measuring the outcome inappropriate?	PN
	4.2 Could measurement or ascertainment of the outcome have differed between intervention groups?	PN
	4.3 Were outcome assessors aware of the intervention received by study participants?	PY
	4.4 If Y/PY/NI to 4.3: Could assessment of the outcome have been influenced by knowledge of intervention received?	PN
	4.5 If Y/PY/NI to 4.4: Is it likely that assessment of the outcome was influenced by knowledge of intervention received?	NA
	**Risk of bias judgement**	**Low**
**Bias in selection of the reported result**	5.1 Were the data that produced this result analysed in accordance with a pre-specified analysis plan that was finalized before unblinded outcome data were available for analysis?	PN
	5.2 ... multiple eligible outcome measurements (e.g. scales, definitions, time points) within the outcome domain?	NI
	5.3 ... multiple eligible analyses of the data?	NI
	**Risk of bias judgement**	**Some concerns**
**Overall bias**	**Risk of bias judgement**	**High**

Study	Rim 2022	
**Domain**	**Signalling question**	**Response**
**Bias arising from the randomization process**	1.1 Was the allocation sequence random?	Y
	1.2 Was the allocation sequence concealed until participants were enrolled and assigned to interventions?	PY
	1.3 Did baseline differences between intervention groups suggest a problem with the randomization process?	PN
	**Risk of bias judgement**	**Low**
**Bias due to deviations from intended interventions**	2.1.Were participants aware of their assigned intervention during the trial?	PY
	2.2.Were carers and people delivering the interventions aware of participants' assigned intervention during the trial?	PY
	2.3. If Y/PY/NI to 2.1 or 2.2: Were there deviations from the intended intervention that arose because of the experimental context?	PY
	2.4 If Y/PY to 2.3: Were these deviations likely to have affected the outcome?	PN
	2.5. If Y/PY/NI to 2.4: Were these deviations from intended intervention balanced between groups?	NA
	2.6 Was an appropriate analysis used to estimate the effect of assignment to intervention?	PY
	2.7 If N/PN/NI to 2.6: Was there potential for a substantial impact (on the result) of the failure to analyse participants in the group to which they were randomized?	NA
	**Risk of bias judgement**	**Some concerns**
**Bias due to missing outcome data**	3.1 Were data for this outcome available for all, or nearly all, participants randomized?	PN
	3.2 If N/PN/NI to 3.1: Is there evidence that result was not biased by missing outcome data?	PN
	3.3 If N/PN to 3.2: Could missingness in the outcome depend on its true value?	PY
	3.4 If Y/PY/NI to 3.3: Is it likely that missingness in the outcome depended on its true value?	PN
	**Risk of bias judgement**	**Some concerns**
**Bias in measurement of the outcome**	4.1 Was the method of measuring the outcome inappropriate?	PN
	4.2 Could measurement or ascertainment of the outcome have differed between intervention groups?	PN
	4.3 Were outcome assessors aware of the intervention received by study participants?	PY
	4.4 If Y/PY/NI to 4.3: Could assessment of the outcome have been influenced by knowledge of intervention received?	NI
	4.5 If Y/PY/NI to 4.4: Is it likely that assessment of the outcome was influenced by knowledge of intervention received?	NI
	**Risk of bias judgement**	**High**
**Bias in selection of the reported result**	5.1 Were the data that produced this result analysed in accordance with a pre-specified analysis plan that was finalized before unblinded outcome data were available for analysis?	PY
	5.2 ... multiple eligible outcome measurements (e.g. scales, definitions, time points) within the outcome domain?	NI
	5.3 ... multiple eligible analyses of the data?	NI
	**Risk of bias judgement**	**Some concerns**
**Overall bias**	**Risk of bias judgement**	**High**

Study	Lara-Palomo 2022	
**Domain**	**Signalling question**	**Response**
**Bias arising from the randomization process**	1.1 Was the allocation sequence random?	Y
	1.2 Was the allocation sequence concealed until participants were enrolled and assigned to interventions?	PY
	1.3 Did baseline differences between intervention groups suggest a problem with the randomization process?	PN
	**Risk of bias judgement**	**Low**
**Bias due to deviations from intended interventions**	2.1.Were participants aware of their assigned intervention during the trial?	PY
	2.2.Were carers and people delivering the interventions aware of participants' assigned intervention during the trial?	PN
	2.3. If Y/PY/NI to 2.1 or 2.2: Were there deviations from the intended intervention that arose because of the experimental context?	PN
	2.4 If Y/PY to 2.3: Were these deviations likely to have affected the outcome?	NA
	2.5. If Y/PY/NI to 2.4: Were these deviations from intended intervention balanced between groups?	NA
	2.6 Was an appropriate analysis used to estimate the effect of assignment to intervention?	PY
	2.7 If N/PN/NI to 2.6: Was there potential for a substantial impact (on the result) of the failure to analyse participants in the group to which they were randomized?	NA
	**Risk of bias judgement**	**Low**
**Bias due to missing outcome data**	3.1 Were data for this outcome available for all, or nearly all, participants randomized?	PN
	3.2 If N/PN/NI to 3.1: Is there evidence that result was not biased by missing outcome data?	PY
	3.3 If N/PN to 3.2: Could missingness in the outcome depend on its true value?	NA
	3.4 If Y/PY/NI to 3.3: Is it likely that missingness in the outcome depended on its true value?	NA
	**Risk of bias judgement**	**Low**
**Bias in measurement of the outcome**	4.1 Was the method of measuring the outcome inappropriate?	PN
	4.2 Could measurement or ascertainment of the outcome have differed between intervention groups?	PN
	4.3 Were outcome assessors aware of the intervention received by study participants?	PN
	4.4 If Y/PY/NI to 4.3: Could assessment of the outcome have been influenced by knowledge of intervention received?	NA
	4.5 If Y/PY/NI to 4.4: Is it likely that assessment of the outcome was influenced by knowledge of intervention received?	NA
	**Risk of bias judgement**	**Low**
**Bias in selection of the reported result**	5.1 Were the data that produced this result analysed in accordance with a pre-specified analysis plan that was finalized before unblinded outcome data were available for analysis?	PY
	5.2 ... multiple eligible outcome measurements (e.g. scales, definitions, time points) within the outcome domain?	NI
	5.3 ... multiple eligible analyses of the data?	NI
	**Risk of bias judgement**	**Some concerns**
**Overall bias**	**Risk of bias judgement**	**Some concerns**

Study	Kızıltaş 2022	
**Domain**	**Signalling question**	**Response**
**Bias arising from the randomization process**	1.1 Was the allocation sequence random?	Y
	1.2 Was the allocation sequence concealed until participants were enrolled and assigned to interventions?	PY
	1.3 Did baseline differences between intervention groups suggest a problem with the randomization process?	PN
	**Risk of bias judgement**	**Low**
**Bias due to deviations from intended interventions**	2.1.Were participants aware of their assigned intervention during the trial?	PY
	2.2.Were carers and people delivering the interventions aware of participants' assigned intervention during the trial?	PY
	2.3. If Y/PY/NI to 2.1 or 2.2: Were there deviations from the intended intervention that arose because of the experimental context?	PY
	2.4 If Y/PY to 2.3: Were these deviations likely to have affected the outcome?	PY
	2.5. If Y/PY/NI to 2.4: Were these deviations from intended intervention balanced between groups?	PY
	2.6 Was an appropriate analysis used to estimate the effect of assignment to intervention?	PN
	2.7 If N/PN/NI to 2.6: Was there potential for a substantial impact (on the result) of the failure to analyse participants in the group to which they were randomized?	PN
	**Risk of bias judgement**	**Some concerns**
**Bias due to missing outcome data**	3.1 Were data for this outcome available for all, or nearly all, participants randomized?	PN
	3.2 If N/PN/NI to 3.1: Is there evidence that result was not biased by missing outcome data?	PN
	3.3 If N/PN to 3.2: Could missingness in the outcome depend on its true value?	PY
	3.4 If Y/PY/NI to 3.3: Is it likely that missingness in the outcome depended on its true value?	PY
	**Risk of bias judgement**	**High**
**Bias in measurement of the outcome**	4.1 Was the method of measuring the outcome inappropriate?	PN
	4.2 Could measurement or ascertainment of the outcome have differed between intervention groups?	PN
	4.3 Were outcome assessors aware of the intervention received by study participants?	PY
	4.4 If Y/PY/NI to 4.3: Could assessment of the outcome have been influenced by knowledge of intervention received?	NI
	4.5 If Y/PY/NI to 4.4: Is it likely that assessment of the outcome was influenced by knowledge of intervention received?	NI
	**Risk of bias judgement**	**High**
**Bias in selection of the reported result**	5.1 Were the data that produced this result analysed in accordance with a pre-specified analysis plan that was finalized before unblinded outcome data were available for analysis?	PY
	5.2 ... multiple eligible outcome measurements (e.g. scales, definitions, time points) within the outcome domain?	NI
	5.3 ... multiple eligible analyses of the data?	NI
	**Risk of bias judgement**	**Some concerns**
**Overall bias**	**Risk of bias judgement**	**High**

Study	Hrkać 2022	
**Domain**	**Signalling question**	**Response**
**Bias arising from the randomization process**	1.1 Was the allocation sequence random?	Y
	1.2 Was the allocation sequence concealed until participants were enrolled and assigned to interventions?	PY
	1.3 Did baseline differences between intervention groups suggest a problem with the randomization process?	PN
	**Risk of bias judgement**	**Low**
**Bias due to deviations from intended interventions**	2.1.Were participants aware of their assigned intervention during the trial?	PY
	2.2.Were carers and people delivering the interventions aware of participants' assigned intervention during the trial?	PY
	2.3. If Y/PY/NI to 2.1 or 2.2: Were there deviations from the intended intervention that arose because of the experimental context?	PN
	2.4 If Y/PY to 2.3: Were these deviations likely to have affected the outcome?	NA
	2.5. If Y/PY/NI to 2.4: Were these deviations from intended intervention balanced between groups?	NA
	2.6 Was an appropriate analysis used to estimate the effect of assignment to intervention?	PY
	2.7 If N/PN/NI to 2.6: Was there potential for a substantial impact (on the result) of the failure to analyse participants in the group to which they were randomized?	NA
	**Risk of bias judgement**	**Low**
**Bias due to missing outcome data**	3.1 Were data for this outcome available for all, or nearly all, participants randomized?	PN
	3.2 If N/PN/NI to 3.1: Is there evidence that result was not biased by missing outcome data?	PY
	3.3 If N/PN to 3.2: Could missingness in the outcome depend on its true value?	NA
	3.4 If Y/PY/NI to 3.3: Is it likely that missingness in the outcome depended on its true value?	NA
	**Risk of bias judgement**	**Low**
**Bias in measurement of the outcome**	4.1 Was the method of measuring the outcome inappropriate?	PN
	4.2 Could measurement or ascertainment of the outcome have differed between intervention groups?	PN
	4.3 Were outcome assessors aware of the intervention received by study participants?	PN
	4.4 If Y/PY/NI to 4.3: Could assessment of the outcome have been influenced by knowledge of intervention received?	NA
	4.5 If Y/PY/NI to 4.4: Is it likely that assessment of the outcome was influenced by knowledge of intervention received?	NA
	**Risk of bias judgement**	**Low**
**Bias in selection of the reported result**	5.1 Were the data that produced this result analysed in accordance with a pre-specified analysis plan that was finalized before unblinded outcome data were available for analysis?	PY
	5.2 ... multiple eligible outcome measurements (e.g. scales, definitions, time points) within the outcome domain?	PN
	5.3 ... multiple eligible analyses of the data?	PN
	**Risk of bias judgement**	**Low**
**Overall bias**	**Risk of bias judgement**	**Low**

Study	Diez 2022	
**Domain**	**Signalling question**	**Response**
**Bias arising from the randomization process**	1.1 Was the allocation sequence random?	Y
	1.2 Was the allocation sequence concealed until participants were enrolled and assigned to interventions?	PY
	1.3 Did baseline differences between intervention groups suggest a problem with the randomization process?	PN
	**Risk of bias judgement**	**Low**
**Bias due to deviations from intended interventions**	2.1.Were participants aware of their assigned intervention during the trial?	PY
	2.2.Were carers and people delivering the interventions aware of participants' assigned intervention during the trial?	PY
	2.3. If Y/PY/NI to 2.1 or 2.2: Were there deviations from the intended intervention that arose because of the experimental context?	NI
	2.4 If Y/PY to 2.3: Were these deviations likely to have affected the outcome?	NA
	2.5. If Y/PY/NI to 2.4: Were these deviations from intended intervention balanced between groups?	NA
	2.6 Was an appropriate analysis used to estimate the effect of assignment to intervention?	PY
	2.7 If N/PN/NI to 2.6: Was there potential for a substantial impact (on the result) of the failure to analyse participants in the group to which they were randomized?	NA
	**Risk of bias judgement**	**Some concerns**
**Bias due to missing outcome data**	3.1 Were data for this outcome available for all, or nearly all, participants randomized?	PY
	3.2 If N/PN/NI to 3.1: Is there evidence that result was not biased by missing outcome data?	NA
	3.3 If N/PN to 3.2: Could missingness in the outcome depend on its true value?	NA
	3.4 If Y/PY/NI to 3.3: Is it likely that missingness in the outcome depended on its true value?	NA
	**Risk of bias judgement**	**Low**
**Bias in measurement of the outcome**	4.1 Was the method of measuring the outcome inappropriate?	PN
	4.2 Could measurement or ascertainment of the outcome have differed between intervention groups?	PN
	4.3 Were outcome assessors aware of the intervention received by study participants?	PN
	4.4 If Y/PY/NI to 4.3: Could assessment of the outcome have been influenced by knowledge of intervention received?	NA
	4.5 If Y/PY/NI to 4.4: Is it likely that assessment of the outcome was influenced by knowledge of intervention received?	NA
	**Risk of bias judgement**	**Low**
**Bias in selection of the reported result**	5.1 Were the data that produced this result analysed in accordance with a pre-specified analysis plan that was finalized before unblinded outcome data were available for analysis?	PY
	5.2 ... multiple eligible outcome measurements (e.g. scales, definitions, time points) within the outcome domain?	PN
	5.3 ... multiple eligible analyses of the data?	PN
	**Risk of bias judgement**	**Low**
**Overall bias**	**Risk of bias judgement**	**Some concerns**

Study	Aguilar-Ferrándiz 2022	
**Domain**	**Signalling question**	**Response**
**Bias arising from the randomization process**	1.1 Was the allocation sequence random?	Y
	1.2 Was the allocation sequence concealed until participants were enrolled and assigned to interventions?	PY
	1.3 Did baseline differences between intervention groups suggest a problem with the randomization process?	PN
	**Risk of bias judgement**	**Low**
**Bias due to deviations from intended interventions**	2.1.Were participants aware of their assigned intervention during the trial?	PY
	2.2.Were carers and people delivering the interventions aware of participants' assigned intervention during the trial?	PN
	2.3. If Y/PY/NI to 2.1 or 2.2: Were there deviations from the intended intervention that arose because of the experimental context?	PN
	2.4 If Y/PY to 2.3: Were these deviations likely to have affected the outcome?	NA
	2.5. If Y/PY/NI to 2.4: Were these deviations from intended intervention balanced between groups?	NA
	2.6 Was an appropriate analysis used to estimate the effect of assignment to intervention?	PY
	2.7 If N/PN/NI to 2.6: Was there potential for a substantial impact (on the result) of the failure to analyse participants in the group to which they were randomized?	NA
	**Risk of bias judgement**	**Low**
**Bias due to missing outcome data**	3.1 Were data for this outcome available for all, or nearly all, participants randomized?	Y
	3.2 If N/PN/NI to 3.1: Is there evidence that result was not biased by missing outcome data?	NA
	3.3 If N/PN to 3.2: Could missingness in the outcome depend on its true value?	NA
	3.4 If Y/PY/NI to 3.3: Is it likely that missingness in the outcome depended on its true value?	NA
	**Risk of bias judgement**	**Low**
**Bias in measurement of the outcome**	4.1 Was the method of measuring the outcome inappropriate?	PN
	4.2 Could measurement or ascertainment of the outcome have differed between intervention groups?	PN
	4.3 Were outcome assessors aware of the intervention received by study participants?	PN
	4.4 If Y/PY/NI to 4.3: Could assessment of the outcome have been influenced by knowledge of intervention received?	NA
	4.5 If Y/PY/NI to 4.4: Is it likely that assessment of the outcome was influenced by knowledge of intervention received?	NA
	**Risk of bias judgement**	**Low**
**Bias in selection of the reported result**	5.1 Were the data that produced this result analysed in accordance with a pre-specified analysis plan that was finalized before unblinded outcome data were available for analysis?	PY
	5.2 ... multiple eligible outcome measurements (e.g. scales, definitions, time points) within the outcome domain?	NI
	5.3 ... multiple eligible analyses of the data?	NI
	**Risk of bias judgement**	**Some concerns**
**Overall bias**	**Risk of bias judgement**	**Some concerns**

Study	Yakşi 2021	
**Domain**	**Signalling question**	**Response**
**Bias arising from the randomization process**	1.1 Was the allocation sequence random?	Y
	1.2 Was the allocation sequence concealed until participants were enrolled and assigned to interventions?	Y
	1.3 Did baseline differences between intervention groups suggest a problem with the randomization process?	PN
	**Risk of bias judgement**	**Low**
**Bias due to deviations from intended interventions**	2.1.Were participants aware of their assigned intervention during the trial?	N
	2.2.Were carers and people delivering the interventions aware of participants' assigned intervention during the trial?	PY
	2.3. If Y/PY/NI to 2.1 or 2.2: Were there deviations from the intended intervention that arose because of the experimental context?	PN
	2.4 If Y/PY to 2.3: Were these deviations likely to have affected the outcome?	NA
	2.5. If Y/PY/NI to 2.4: Were these deviations from intended intervention balanced between groups?	NA
	2.6 Was an appropriate analysis used to estimate the effect of assignment to intervention?	PY
	2.7 If N/PN/NI to 2.6: Was there potential for a substantial impact (on the result) of the failure to analyse participants in the group to which they were randomized?	NA
	**Risk of bias judgement**	**Low**
**Bias due to missing outcome data**	3.1 Were data for this outcome available for all, or nearly all, participants randomized?	PY
	3.2 If N/PN/NI to 3.1: Is there evidence that result was not biased by missing outcome data?	NA
	3.3 If N/PN to 3.2: Could missingness in the outcome depend on its true value?	NA
	3.4 If Y/PY/NI to 3.3: Is it likely that missingness in the outcome depended on its true value?	NA
	**Risk of bias judgement**	**Low**
**Bias in measurement of the outcome**	4.1 Was the method of measuring the outcome inappropriate?	PN
	4.2 Could measurement or ascertainment of the outcome have differed between intervention groups?	PN
	4.3 Were outcome assessors aware of the intervention received by study participants?	PY
	4.4 If Y/PY/NI to 4.3: Could assessment of the outcome have been influenced by knowledge of intervention received?	NI
	4.5 If Y/PY/NI to 4.4: Is it likely that assessment of the outcome was influenced by knowledge of intervention received?	NI
	**Risk of bias judgement**	**High**
**Bias in selection of the reported result**	5.1 Were the data that produced this result analysed in accordance with a pre-specified analysis plan that was finalized before unblinded outcome data were available for analysis?	PY
	5.2 ... multiple eligible outcome measurements (e.g. scales, definitions, time points) within the outcome domain?	NI
	5.3 ... multiple eligible analyses of the data?	NI
	**Risk of bias judgement**	**Some concerns**
**Overall bias**	**Risk of bias judgement**	**High**

Study	Schmidt 2021	
**Domain**	**Signalling question**	**Response**
**Bias arising from the randomization process**	1.1 Was the allocation sequence random?	Y
	1.2 Was the allocation sequence concealed until participants were enrolled and assigned to interventions?	PN
	1.3 Did baseline differences between intervention groups suggest a problem with the randomization process?	PN
	**Risk of bias judgement**	**High**
**Bias due to deviations from intended interventions**	2.1.Were participants aware of their assigned intervention during the trial?	PY
	2.2.Were carers and people delivering the interventions aware of participants' assigned intervention during the trial?	PY
	2.3. If Y/PY/NI to 2.1 or 2.2: Were there deviations from the intended intervention that arose because of the experimental context?	PY
	2.4 If Y/PY to 2.3: Were these deviations likely to have affected the outcome?	PN
	2.5. If Y/PY/NI to 2.4: Were these deviations from intended intervention balanced between groups?	NA
	2.6 Was an appropriate analysis used to estimate the effect of assignment to intervention?	PY
	2.7 If N/PN/NI to 2.6: Was there potential for a substantial impact (on the result) of the failure to analyse participants in the group to which they were randomized?	NA
	**Risk of bias judgement**	**Some concerns**
**Bias due to missing outcome data**	3.1 Were data for this outcome available for all, or nearly all, participants randomized?	PN
	3.2 If N/PN/NI to 3.1: Is there evidence that result was not biased by missing outcome data?	PN
	3.3 If N/PN to 3.2: Could missingness in the outcome depend on its true value?	PY
	3.4 If Y/PY/NI to 3.3: Is it likely that missingness in the outcome depended on its true value?	NI
	**Risk of bias judgement**	**High**
**Bias in measurement of the outcome**	4.1 Was the method of measuring the outcome inappropriate?	PN
	4.2 Could measurement or ascertainment of the outcome have differed between intervention groups?	PN
	4.3 Were outcome assessors aware of the intervention received by study participants?	PY
	4.4 If Y/PY/NI to 4.3: Could assessment of the outcome have been influenced by knowledge of intervention received?	PN
	4.5 If Y/PY/NI to 4.4: Is it likely that assessment of the outcome was influenced by knowledge of intervention received?	NA
	**Risk of bias judgement**	**Low**
**Bias in selection of the reported result**	5.1 Were the data that produced this result analysed in accordance with a pre-specified analysis plan that was finalized before unblinded outcome data were available for analysis?	PY
	5.2 ... multiple eligible outcome measurements (e.g. scales, definitions, time points) within the outcome domain?	PN
	5.3 ... multiple eligible analyses of the data?	PN
	**Risk of bias judgement**	**Low**
**Overall bias**	**Risk of bias judgement**	**High**

Study	Polaski 2021	
**Domain**	**Signalling question**	**Response**
**Bias arising from the randomization process**	1.1 Was the allocation sequence random?	Y
	1.2 Was the allocation sequence concealed until participants were enrolled and assigned to interventions?	PY
	1.3 Did baseline differences between intervention groups suggest a problem with the randomization process?	PN
	**Risk of bias judgement**	**Low**
**Bias due to deviations from intended interventions**	2.1.Were participants aware of their assigned intervention during the trial?	PY
	2.2.Were carers and people delivering the interventions aware of participants' assigned intervention during the trial?	PN
	2.3. If Y/PY/NI to 2.1 or 2.2: Were there deviations from the intended intervention that arose because of the experimental context?	PN
	2.4 If Y/PY to 2.3: Were these deviations likely to have affected the outcome?	NA
	2.5. If Y/PY/NI to 2.4: Were these deviations from intended intervention balanced between groups?	NA
	2.6 Was an appropriate analysis used to estimate the effect of assignment to intervention?	PY
	2.7 If N/PN/NI to 2.6: Was there potential for a substantial impact (on the result) of the failure to analyse participants in the group to which they were randomized?	NA
	**Risk of bias judgement**	**Low**
**Bias due to missing outcome data**	3.1 Were data for this outcome available for all, or nearly all, participants randomized?	PN
	3.2 If N/PN/NI to 3.1: Is there evidence that result was not biased by missing outcome data?	PY
	3.3 If N/PN to 3.2: Could missingness in the outcome depend on its true value?	NA
	3.4 If Y/PY/NI to 3.3: Is it likely that missingness in the outcome depended on its true value?	NA
	**Risk of bias judgement**	**Low**
**Bias in measurement of the outcome**	4.1 Was the method of measuring the outcome inappropriate?	PN
	4.2 Could measurement or ascertainment of the outcome have differed between intervention groups?	PN
	4.3 Were outcome assessors aware of the intervention received by study participants?	PY
	4.4 If Y/PY/NI to 4.3: Could assessment of the outcome have been influenced by knowledge of intervention received?	PY
	4.5 If Y/PY/NI to 4.4: Is it likely that assessment of the outcome was influenced by knowledge of intervention received?	PN
	**Risk of bias judgement**	**Some concerns**
**Bias in selection of the reported result**	5.1 Were the data that produced this result analysed in accordance with a pre-specified analysis plan that was finalized before unblinded outcome data were available for analysis?	PY
	5.2 ... multiple eligible outcome measurements (e.g. scales, definitions, time points) within the outcome domain?	NI
	5.3 ... multiple eligible analyses of the data?	NI
	**Risk of bias judgement**	**Some concerns**
**Overall bias**	**Risk of bias judgement**	**Some concerns**

Study	Karaarslan 2021	
**Domain**	**Signalling question**	**Response**
**Bias arising from the randomization process**	1.1 Was the allocation sequence random?	Y
	1.2 Was the allocation sequence concealed until participants were enrolled and assigned to interventions?	PY
	1.3 Did baseline differences between intervention groups suggest a problem with the randomization process?	PN
	**Risk of bias judgement**	**Low**
**Bias due to deviations from intended interventions**	2.1.Were participants aware of their assigned intervention during the trial?	PY
	2.2.Were carers and people delivering the interventions aware of participants' assigned intervention during the trial?	PY
	2.3. If Y/PY/NI to 2.1 or 2.2: Were there deviations from the intended intervention that arose because of the experimental context?	PN
	2.4 If Y/PY to 2.3: Were these deviations likely to have affected the outcome?	NA
	2.5. If Y/PY/NI to 2.4: Were these deviations from intended intervention balanced between groups?	NA
	2.6 Was an appropriate analysis used to estimate the effect of assignment to intervention?	PY
	2.7 If N/PN/NI to 2.6: Was there potential for a substantial impact (on the result) of the failure to analyse participants in the group to which they were randomized?	NA
	**Risk of bias judgement**	**Low**
**Bias due to missing outcome data**	3.1 Were data for this outcome available for all, or nearly all, participants randomized?	PN
	3.2 If N/PN/NI to 3.1: Is there evidence that result was not biased by missing outcome data?	PN
	3.3 If N/PN to 3.2: Could missingness in the outcome depend on its true value?	PY
	3.4 If Y/PY/NI to 3.3: Is it likely that missingness in the outcome depended on its true value?	PN
	**Risk of bias judgement**	**Some concerns**
**Bias in measurement of the outcome**	4.1 Was the method of measuring the outcome inappropriate?	PN
	4.2 Could measurement or ascertainment of the outcome have differed between intervention groups?	PN
	4.3 Were outcome assessors aware of the intervention received by study participants?	N
	4.4 If Y/PY/NI to 4.3: Could assessment of the outcome have been influenced by knowledge of intervention received?	NA
	4.5 If Y/PY/NI to 4.4: Is it likely that assessment of the outcome was influenced by knowledge of intervention received?	NA
	**Risk of bias judgement**	**Low**
**Bias in selection of the reported result**	5.1 Were the data that produced this result analysed in accordance with a pre-specified analysis plan that was finalized before unblinded outcome data were available for analysis?	PY
	5.2 ... multiple eligible outcome measurements (e.g. scales, definitions, time points) within the outcome domain?	NI
	5.3 ... multiple eligible analyses of the data?	NI
	**Risk of bias judgement**	**Some concerns**
**Overall bias**	**Risk of bias judgement**	**Some concerns**

Study	Darnall 2021	
**Domain**	**Signalling question**	**Response**
**Bias arising from the randomization process**	1.1 Was the allocation sequence random?	Y
	1.2 Was the allocation sequence concealed until participants were enrolled and assigned to interventions?	PY
	1.3 Did baseline differences between intervention groups suggest a problem with the randomization process?	PN
	**Risk of bias judgement**	**Low**
**Bias due to deviations from intended interventions**	2.1.Were participants aware of their assigned intervention during the trial?	PY
	2.2.Were carers and people delivering the interventions aware of participants' assigned intervention during the trial?	PN
	2.3. If Y/PY/NI to 2.1 or 2.2: Were there deviations from the intended intervention that arose because of the experimental context?	PN
	2.4 If Y/PY to 2.3: Were these deviations likely to have affected the outcome?	NA
	2.5. If Y/PY/NI to 2.4: Were these deviations from intended intervention balanced between groups?	NA
	2.6 Was an appropriate analysis used to estimate the effect of assignment to intervention?	PY
	2.7 If N/PN/NI to 2.6: Was there potential for a substantial impact (on the result) of the failure to analyse participants in the group to which they were randomized?	NA
	**Risk of bias judgement**	**Low**
**Bias due to missing outcome data**	3.1 Were data for this outcome available for all, or nearly all, participants randomized?	PN
	3.2 If N/PN/NI to 3.1: Is there evidence that result was not biased by missing outcome data?	PY
	3.3 If N/PN to 3.2: Could missingness in the outcome depend on its true value?	NA
	3.4 If Y/PY/NI to 3.3: Is it likely that missingness in the outcome depended on its true value?	NA
	**Risk of bias judgement**	**Low**
**Bias in measurement of the outcome**	4.1 Was the method of measuring the outcome inappropriate?	PN
	4.2 Could measurement or ascertainment of the outcome have differed between intervention groups?	PN
	4.3 Were outcome assessors aware of the intervention received by study participants?	PN
	4.4 If Y/PY/NI to 4.3: Could assessment of the outcome have been influenced by knowledge of intervention received?	NA
	4.5 If Y/PY/NI to 4.4: Is it likely that assessment of the outcome was influenced by knowledge of intervention received?	NA
	**Risk of bias judgement**	**Low**
**Bias in selection of the reported result**	5.1 Were the data that produced this result analysed in accordance with a pre-specified analysis plan that was finalized before unblinded outcome data were available for analysis?	PY
	5.2 ... multiple eligible outcome measurements (e.g. scales, definitions, time points) within the outcome domain?	PN
	5.3 ... multiple eligible analyses of the data?	PN
	**Risk of bias judgement**	**Low**
**Overall bias**	**Risk of bias judgement**	**Low**

Study	Batıbay 2021	
**Domain**	**Signalling question**	**Response**
**Bias arising from the randomization process**	1.1 Was the allocation sequence random?	Y
	1.2 Was the allocation sequence concealed until participants were enrolled and assigned to interventions?	PY
	1.3 Did baseline differences between intervention groups suggest a problem with the randomization process?	PN
	**Risk of bias judgement**	**Low**
**Bias due to deviations from intended interventions**	2.1.Were participants aware of their assigned intervention during the trial?	PY
	2.2.Were carers and people delivering the interventions aware of participants' assigned intervention during the trial?	PY
	2.3. If Y/PY/NI to 2.1 or 2.2: Were there deviations from the intended intervention that arose because of the experimental context?	PN
	2.4 If Y/PY to 2.3: Were these deviations likely to have affected the outcome?	NA
	2.5. If Y/PY/NI to 2.4: Were these deviations from intended intervention balanced between groups?	NA
	2.6 Was an appropriate analysis used to estimate the effect of assignment to intervention?	PY
	2.7 If N/PN/NI to 2.6: Was there potential for a substantial impact (on the result) of the failure to analyse participants in the group to which they were randomized?	NA
	**Risk of bias judgement**	**Low**
**Bias due to missing outcome data**	3.1 Were data for this outcome available for all, or nearly all, participants randomized?	PY
	3.2 If N/PN/NI to 3.1: Is there evidence that result was not biased by missing outcome data?	NA
	3.3 If N/PN to 3.2: Could missingness in the outcome depend on its true value?	NA
	3.4 If Y/PY/NI to 3.3: Is it likely that missingness in the outcome depended on its true value?	NA
	**Risk of bias judgement**	**Low**
**Bias in measurement of the outcome**	4.1 Was the method of measuring the outcome inappropriate?	PN
	4.2 Could measurement or ascertainment of the outcome have differed between intervention groups?	PN
	4.3 Were outcome assessors aware of the intervention received by study participants?	PY
	4.4 If Y/PY/NI to 4.3: Could assessment of the outcome have been influenced by knowledge of intervention received?	NI
	4.5 If Y/PY/NI to 4.4: Is it likely that assessment of the outcome was influenced by knowledge of intervention received?	NI
	**Risk of bias judgement**	**High**
**Bias in selection of the reported result**	5.1 Were the data that produced this result analysed in accordance with a pre-specified analysis plan that was finalized before unblinded outcome data were available for analysis?	PY
	5.2 ... multiple eligible outcome measurements (e.g. scales, definitions, time points) within the outcome domain?	NI
	5.3 ... multiple eligible analyses of the data?	NI
	**Risk of bias judgement**	**Some concerns**
**Overall bias**	**Risk of bias judgement**	**High**

Study	Alzahrani 2021	
**Domain**	**Signalling question**	**Response**
**Bias arising from the randomization process**	1.1 Was the allocation sequence random?	Y
	1.2 Was the allocation sequence concealed until participants were enrolled and assigned to interventions?	PY
	1.3 Did baseline differences between intervention groups suggest a problem with the randomization process?	PN
	**Risk of bias judgement**	**Low**
**Bias due to deviations from intended interventions**	2.1.Were participants aware of their assigned intervention during the trial?	PY
	2.2.Were carers and people delivering the interventions aware of participants' assigned intervention during the trial?	PY
	2.3. If Y/PY/NI to 2.1 or 2.2: Were there deviations from the intended intervention that arose because of the experimental context?	PN
	2.4 If Y/PY to 2.3: Were these deviations likely to have affected the outcome?	NA
	2.5. If Y/PY/NI to 2.4: Were these deviations from intended intervention balanced between groups?	NA
	2.6 Was an appropriate analysis used to estimate the effect of assignment to intervention?	PY
	2.7 If N/PN/NI to 2.6: Was there potential for a substantial impact (on the result) of the failure to analyse participants in the group to which they were randomized?	NA
	**Risk of bias judgement**	**Low**
**Bias due to missing outcome data**	3.1 Were data for this outcome available for all, or nearly all, participants randomized?	PY
	3.2 If N/PN/NI to 3.1: Is there evidence that result was not biased by missing outcome data?	NA
	3.3 If N/PN to 3.2: Could missingness in the outcome depend on its true value?	NA
	3.4 If Y/PY/NI to 3.3: Is it likely that missingness in the outcome depended on its true value?	NA
	**Risk of bias judgement**	**Low**
**Bias in measurement of the outcome**	4.1 Was the method of measuring the outcome inappropriate?	PN
	4.2 Could measurement or ascertainment of the outcome have differed between intervention groups?	PN
	4.3 Were outcome assessors aware of the intervention received by study participants?	PY
	4.4 If Y/PY/NI to 4.3: Could assessment of the outcome have been influenced by knowledge of intervention received?	NI
	4.5 If Y/PY/NI to 4.4: Is it likely that assessment of the outcome was influenced by knowledge of intervention received?	PN
	**Risk of bias judgement**	**Some concerns**
**Bias in selection of the reported result**	5.1 Were the data that produced this result analysed in accordance with a pre-specified analysis plan that was finalized before unblinded outcome data were available for analysis?	PY
	5.2 ... multiple eligible outcome measurements (e.g. scales, definitions, time points) within the outcome domain?	NI
	5.3 ... multiple eligible analyses of the data?	NI
	**Risk of bias judgement**	**Some concerns**
**Overall bias**	**Risk of bias judgement**	**Some concerns**

Study	Ünal 2020	
**Domain**	**Signalling question**	**Response**
**Bias arising from the randomization process**	1.1 Was the allocation sequence random?	Y
	1.2 Was the allocation sequence concealed until participants were enrolled and assigned to interventions?	PY
	1.3 Did baseline differences between intervention groups suggest a problem with the randomization process?	PN
	**Risk of bias judgement**	**Low**
**Bias due to deviations from intended interventions**	2.1.Were participants aware of their assigned intervention during the trial?	PY
	2.2.Were carers and people delivering the interventions aware of participants' assigned intervention during the trial?	PY
	2.3. If Y/PY/NI to 2.1 or 2.2: Were there deviations from the intended intervention that arose because of the experimental context?	Y
	2.4 If Y/PY to 2.3: Were these deviations likely to have affected the outcome?	PN
	2.5. If Y/PY/NI to 2.4: Were these deviations from intended intervention balanced between groups?	NA
	2.6 Was an appropriate analysis used to estimate the effect of assignment to intervention?	PN
	2.7 If N/PN/NI to 2.6: Was there potential for a substantial impact (on the result) of the failure to analyse participants in the group to which they were randomized?	PN
	**Risk of bias judgement**	**Some concerns**
**Bias due to missing outcome data**	3.1 Were data for this outcome available for all, or nearly all, participants randomized?	PY
	3.2 If N/PN/NI to 3.1: Is there evidence that result was not biased by missing outcome data?	NA
	3.3 If N/PN to 3.2: Could missingness in the outcome depend on its true value?	NA
	3.4 If Y/PY/NI to 3.3: Is it likely that missingness in the outcome depended on its true value?	NA
	**Risk of bias judgement**	**Low**
**Bias in measurement of the outcome**	4.1 Was the method of measuring the outcome inappropriate?	PN
	4.2 Could measurement or ascertainment of the outcome have differed between intervention groups?	PN
	4.3 Were outcome assessors aware of the intervention received by study participants?	PN
	4.4 If Y/PY/NI to 4.3: Could assessment of the outcome have been influenced by knowledge of intervention received?	NA
	4.5 If Y/PY/NI to 4.4: Is it likely that assessment of the outcome was influenced by knowledge of intervention received?	NA
	**Risk of bias judgement**	**Low**
**Bias in selection of the reported result**	5.1 Were the data that produced this result analysed in accordance with a pre-specified analysis plan that was finalized before unblinded outcome data were available for analysis?	PY
	5.2 ... multiple eligible outcome measurements (e.g. scales, definitions, time points) within the outcome domain?	NI
	5.3 ... multiple eligible analyses of the data?	NI
	**Risk of bias judgement**	**Some concerns**
**Overall bias**	**Risk of bias judgement**	**Some concerns**

Study	Soleymani 2020	
**Domain**	**Signalling question**	**Response**
**Bias arising from the randomization process**	1.1 Was the allocation sequence random?	Y
	1.2 Was the allocation sequence concealed until participants were enrolled and assigned to interventions?	PY
	1.3 Did baseline differences between intervention groups suggest a problem with the randomization process?	PN
	**Risk of bias judgement**	**Low**
**Bias due to deviations from intended interventions**	2.1.Were participants aware of their assigned intervention during the trial?	PY
	2.2.Were carers and people delivering the interventions aware of participants' assigned intervention during the trial?	PY
	2.3. If Y/PY/NI to 2.1 or 2.2: Were there deviations from the intended intervention that arose because of the experimental context?	PN
	2.4 If Y/PY to 2.3: Were these deviations likely to have affected the outcome?	NA
	2.5. If Y/PY/NI to 2.4: Were these deviations from intended intervention balanced between groups?	NA
	2.6 Was an appropriate analysis used to estimate the effect of assignment to intervention?	PY
	2.7 If N/PN/NI to 2.6: Was there potential for a substantial impact (on the result) of the failure to analyse participants in the group to which they were randomized?	NA
	**Risk of bias judgement**	**Low**
**Bias due to missing outcome data**	3.1 Were data for this outcome available for all, or nearly all, participants randomized?	PN
	3.2 If N/PN/NI to 3.1: Is there evidence that result was not biased by missing outcome data?	PN
	3.3 If N/PN to 3.2: Could missingness in the outcome depend on its true value?	PY
	3.4 If Y/PY/NI to 3.3: Is it likely that missingness in the outcome depended on its true value?	PN
	**Risk of bias judgement**	**Some concerns**
**Bias in measurement of the outcome**	4.1 Was the method of measuring the outcome inappropriate?	PN
	4.2 Could measurement or ascertainment of the outcome have differed between intervention groups?	PN
	4.3 Were outcome assessors aware of the intervention received by study participants?	PY
	4.4 If Y/PY/NI to 4.3: Could assessment of the outcome have been influenced by knowledge of intervention received?	PY
	4.5 If Y/PY/NI to 4.4: Is it likely that assessment of the outcome was influenced by knowledge of intervention received?	PN
	**Risk of bias judgement**	**Some concerns**
**Bias in selection of the reported result**	5.1 Were the data that produced this result analysed in accordance with a pre-specified analysis plan that was finalized before unblinded outcome data were available for analysis?	PY
	5.2 ... multiple eligible outcome measurements (e.g. scales, definitions, time points) within the outcome domain?	NI
	5.3 ... multiple eligible analyses of the data?	NI
	**Risk of bias judgement**	**Some concerns**
**Overall bias**	**Risk of bias judgement**	**Some concerns**

Study	Schlicker 2020	
**Domain**	**Signalling question**	**Response**
**Bias arising from the randomization process**	1.1 Was the allocation sequence random?	Y
	1.2 Was the allocation sequence concealed until participants were enrolled and assigned to interventions?	Y
	1.3 Did baseline differences between intervention groups suggest a problem with the randomization process?	PN
	**Risk of bias judgement**	**Low**
**Bias due to deviations from intended interventions**	2.1.Were participants aware of their assigned intervention during the trial?	Y
	2.2.Were carers and people delivering the interventions aware of participants' assigned intervention during the trial?	PY
	2.3. If Y/PY/NI to 2.1 or 2.2: Were there deviations from the intended intervention that arose because of the experimental context?	PY
	2.4 If Y/PY to 2.3: Were these deviations likely to have affected the outcome?	PY
	2.5. If Y/PY/NI to 2.4: Were these deviations from intended intervention balanced between groups?	PY
	2.6 Was an appropriate analysis used to estimate the effect of assignment to intervention?	Y
	2.7 If N/PN/NI to 2.6: Was there potential for a substantial impact (on the result) of the failure to analyse participants in the group to which they were randomized?	NA
	**Risk of bias judgement**	**Some concerns**
**Bias due to missing outcome data**	3.1 Were data for this outcome available for all, or nearly all, participants randomized?	Y
	3.2 If N/PN/NI to 3.1: Is there evidence that result was not biased by missing outcome data?	NA
	3.3 If N/PN to 3.2: Could missingness in the outcome depend on its true value?	NA
	3.4 If Y/PY/NI to 3.3: Is it likely that missingness in the outcome depended on its true value?	NA
	**Risk of bias judgement**	**Low**
**Bias in measurement of the outcome**	4.1 Was the method of measuring the outcome inappropriate?	PN
	4.2 Could measurement or ascertainment of the outcome have differed between intervention groups?	PN
	4.3 Were outcome assessors aware of the intervention received by study participants?	PY
	4.4 If Y/PY/NI to 4.3: Could assessment of the outcome have been influenced by knowledge of intervention received?	PY
	4.5 If Y/PY/NI to 4.4: Is it likely that assessment of the outcome was influenced by knowledge of intervention received?	PY
	**Risk of bias judgement**	**High**
**Bias in selection of the reported result**	5.1 Were the data that produced this result analysed in accordance with a pre-specified analysis plan that was finalized before unblinded outcome data were available for analysis?	PY
	5.2 ... multiple eligible outcome measurements (e.g. scales, definitions, time points) within the outcome domain?	PN
	5.3 ... multiple eligible analyses of the data?	PN
	**Risk of bias judgement**	**Low**
**Overall bias**	**Risk of bias judgement**	**High**

Study	de Oliveira Meirelles 2020	
**Domain**	**Signalling question**	**Response**
**Bias arising from the randomization process**	1.1 Was the allocation sequence random?	Y
	1.2 Was the allocation sequence concealed until participants were enrolled and assigned to interventions?	Y
	1.3 Did baseline differences between intervention groups suggest a problem with the randomization process?	PN
	**Risk of bias judgement**	**Low**
**Bias due to deviations from intended interventions**	2.1.Were participants aware of their assigned intervention during the trial?	PY
	2.2.Were carers and people delivering the interventions aware of participants' assigned intervention during the trial?	N
	2.3. If Y/PY/NI to 2.1 or 2.2: Were there deviations from the intended intervention that arose because of the experimental context?	PN
	2.4 If Y/PY to 2.3: Were these deviations likely to have affected the outcome?	NA
	2.5. If Y/PY/NI to 2.4: Were these deviations from intended intervention balanced between groups?	NA
	2.6 Was an appropriate analysis used to estimate the effect of assignment to intervention?	PY
	2.7 If N/PN/NI to 2.6: Was there potential for a substantial impact (on the result) of the failure to analyse participants in the group to which they were randomized?	NA
	**Risk of bias judgement**	**Low**
**Bias due to missing outcome data**	3.1 Were data for this outcome available for all, or nearly all, participants randomized?	PY
	3.2 If N/PN/NI to 3.1: Is there evidence that result was not biased by missing outcome data?	NA
	3.3 If N/PN to 3.2: Could missingness in the outcome depend on its true value?	NA
	3.4 If Y/PY/NI to 3.3: Is it likely that missingness in the outcome depended on its true value?	NA
	**Risk of bias judgement**	**Low**
**Bias in measurement of the outcome**	4.1 Was the method of measuring the outcome inappropriate?	PN
	4.2 Could measurement or ascertainment of the outcome have differed between intervention groups?	PN
	4.3 Were outcome assessors aware of the intervention received by study participants?	N
	4.4 If Y/PY/NI to 4.3: Could assessment of the outcome have been influenced by knowledge of intervention received?	NA
	4.5 If Y/PY/NI to 4.4: Is it likely that assessment of the outcome was influenced by knowledge of intervention received?	NA
	**Risk of bias judgement**	**Low**
**Bias in selection of the reported result**	5.1 Were the data that produced this result analysed in accordance with a pre-specified analysis plan that was finalized before unblinded outcome data were available for analysis?	PY
	5.2 ... multiple eligible outcome measurements (e.g. scales, definitions, time points) within the outcome domain?	NI
	5.3 ... multiple eligible analyses of the data?	NI
	**Risk of bias judgement**	**Some concerns**
**Overall bias**	**Risk of bias judgement**	**Some concerns**

Study	Suh 2019	
**Domain**	**Signalling question**	**Response**
**Bias arising from the randomization process**	1.1 Was the allocation sequence random?	Y
	1.2 Was the allocation sequence concealed until participants were enrolled and assigned to interventions?	PY
	1.3 Did baseline differences between intervention groups suggest a problem with the randomization process?	PY
	**Risk of bias judgement**	**Some concerns**
**Bias due to deviations from intended interventions**	2.1.Were participants aware of their assigned intervention during the trial?	Y
	2.2.Were carers and people delivering the interventions aware of participants' assigned intervention during the trial?	PY
	2.3. If Y/PY/NI to 2.1 or 2.2: Were there deviations from the intended intervention that arose because of the experimental context?	PY
	2.4 If Y/PY to 2.3: Were these deviations likely to have affected the outcome?	PY
	2.5. If Y/PY/NI to 2.4: Were these deviations from intended intervention balanced between groups?	PY
	2.6 Was an appropriate analysis used to estimate the effect of assignment to intervention?	Y
	2.7 If N/PN/NI to 2.6: Was there potential for a substantial impact (on the result) of the failure to analyse participants in the group to which they were randomized?	NA
	**Risk of bias judgement**	**Some concerns**
**Bias due to missing outcome data**	3.1 Were data for this outcome available for all, or nearly all, participants randomized?	PN
	3.2 If N/PN/NI to 3.1: Is there evidence that result was not biased by missing outcome data?	PY
	3.3 If N/PN to 3.2: Could missingness in the outcome depend on its true value?	NA
	3.4 If Y/PY/NI to 3.3: Is it likely that missingness in the outcome depended on its true value?	NA
	**Risk of bias judgement**	**Low**
**Bias in measurement of the outcome**	4.1 Was the method of measuring the outcome inappropriate?	PN
	4.2 Could measurement or ascertainment of the outcome have differed between intervention groups?	PN
	4.3 Were outcome assessors aware of the intervention received by study participants?	PY
	4.4 If Y/PY/NI to 4.3: Could assessment of the outcome have been influenced by knowledge of intervention received?	PN
	4.5 If Y/PY/NI to 4.4: Is it likely that assessment of the outcome was influenced by knowledge of intervention received?	NA
	**Risk of bias judgement**	**Low**
**Bias in selection of the reported result**	5.1 Were the data that produced this result analysed in accordance with a pre-specified analysis plan that was finalized before unblinded outcome data were available for analysis?	PY
	5.2 ... multiple eligible outcome measurements (e.g. scales, definitions, time points) within the outcome domain?	NI
	5.3 ... multiple eligible analyses of the data?	NI
	**Risk of bias judgement**	**Some concerns**
**Overall bias**	**Risk of bias judgement**	**Some concerns**

Study	Petrozzi 2019	
**Domain**	**Signalling question**	**Response**
**Bias arising from the randomization process**	1.1 Was the allocation sequence random?	Y
	1.2 Was the allocation sequence concealed until participants were enrolled and assigned to interventions?	Y
	1.3 Did baseline differences between intervention groups suggest a problem with the randomization process?	PY
	**Risk of bias judgement**	**Some concerns**
**Bias due to deviations from intended interventions**	2.1.Were participants aware of their assigned intervention during the trial?	PY
	2.2.Were carers and people delivering the interventions aware of participants' assigned intervention during the trial?	PN
	2.3. If Y/PY/NI to 2.1 or 2.2: Were there deviations from the intended intervention that arose because of the experimental context?	PN
	2.4 If Y/PY to 2.3: Were these deviations likely to have affected the outcome?	NA
	2.5. If Y/PY/NI to 2.4: Were these deviations from intended intervention balanced between groups?	NA
	2.6 Was an appropriate analysis used to estimate the effect of assignment to intervention?	PY
	2.7 If N/PN/NI to 2.6: Was there potential for a substantial impact (on the result) of the failure to analyse participants in the group to which they were randomized?	NA
	**Risk of bias judgement**	**Low**
**Bias due to missing outcome data**	3.1 Were data for this outcome available for all, or nearly all, participants randomized?	PN
	3.2 If N/PN/NI to 3.1: Is there evidence that result was not biased by missing outcome data?	PN
	3.3 If N/PN to 3.2: Could missingness in the outcome depend on its true value?	PY
	3.4 If Y/PY/NI to 3.3: Is it likely that missingness in the outcome depended on its true value?	PN
	**Risk of bias judgement**	**Some concerns**
**Bias in measurement of the outcome**	4.1 Was the method of measuring the outcome inappropriate?	PN
	4.2 Could measurement or ascertainment of the outcome have differed between intervention groups?	PN
	4.3 Were outcome assessors aware of the intervention received by study participants?	PN
	4.4 If Y/PY/NI to 4.3: Could assessment of the outcome have been influenced by knowledge of intervention received?	NA
	4.5 If Y/PY/NI to 4.4: Is it likely that assessment of the outcome was influenced by knowledge of intervention received?	NA
	**Risk of bias judgement**	**Low**
**Bias in selection of the reported result**	5.1 Were the data that produced this result analysed in accordance with a pre-specified analysis plan that was finalized before unblinded outcome data were available for analysis?	PY
	5.2 ... multiple eligible outcome measurements (e.g. scales, definitions, time points) within the outcome domain?	NI
	5.3 ... multiple eligible analyses of the data?	NI
	**Risk of bias judgement**	**Some concerns**
**Overall bias**	**Risk of bias judgement**	**Some concerns**

Study	Mariano 2019	
**Domain**	**Signalling question**	**Response**
**Bias arising from the randomization process**	1.1 Was the allocation sequence random?	Y
	1.2 Was the allocation sequence concealed until participants were enrolled and assigned to interventions?	NI
	1.3 Did baseline differences between intervention groups suggest a problem with the randomization process?	PN
	**Risk of bias judgement**	**Some concerns**
**Bias due to deviations from intended interventions**	2.1.Were participants aware of their assigned intervention during the trial?	PY
	2.2.Were carers and people delivering the interventions aware of participants' assigned intervention during the trial?	PY
	2.3. If Y/PY/NI to 2.1 or 2.2: Were there deviations from the intended intervention that arose because of the experimental context?	PN
	2.4 If Y/PY to 2.3: Were these deviations likely to have affected the outcome?	NA
	2.5. If Y/PY/NI to 2.4: Were these deviations from intended intervention balanced between groups?	NA
	2.6 Was an appropriate analysis used to estimate the effect of assignment to intervention?	PY
	2.7 If N/PN/NI to 2.6: Was there potential for a substantial impact (on the result) of the failure to analyse participants in the group to which they were randomized?	NA
	**Risk of bias judgement**	**Low**
**Bias due to missing outcome data**	3.1 Were data for this outcome available for all, or nearly all, participants randomized?	PY
	3.2 If N/PN/NI to 3.1: Is there evidence that result was not biased by missing outcome data?	NA
	3.3 If N/PN to 3.2: Could missingness in the outcome depend on its true value?	NA
	3.4 If Y/PY/NI to 3.3: Is it likely that missingness in the outcome depended on its true value?	NA
	**Risk of bias judgement**	**Low**
**Bias in measurement of the outcome**	4.1 Was the method of measuring the outcome inappropriate?	PN
	4.2 Could measurement or ascertainment of the outcome have differed between intervention groups?	PN
	4.3 Were outcome assessors aware of the intervention received by study participants?	PY
	4.4 If Y/PY/NI to 4.3: Could assessment of the outcome have been influenced by knowledge of intervention received?	PN
	4.5 If Y/PY/NI to 4.4: Is it likely that assessment of the outcome was influenced by knowledge of intervention received?	NA
	**Risk of bias judgement**	**Low**
**Bias in selection of the reported result**	5.1 Were the data that produced this result analysed in accordance with a pre-specified analysis plan that was finalized before unblinded outcome data were available for analysis?	PY
	5.2 ... multiple eligible outcome measurements (e.g. scales, definitions, time points) within the outcome domain?	NI
	5.3 ... multiple eligible analyses of the data?	NI
	**Risk of bias judgement**	**Some concerns**
**Overall bias**	**Risk of bias judgement**	**Some concerns**

Study	Hüppe 2019	
**Domain**	**Signalling question**	**Response**
**Bias arising from the randomization process**	1.1 Was the allocation sequence random?	Y
	1.2 Was the allocation sequence concealed until participants were enrolled and assigned to interventions?	PY
	1.3 Did baseline differences between intervention groups suggest a problem with the randomization process?	PN
	**Risk of bias judgement**	**Low**
**Bias due to deviations from intended interventions**	2.1.Were participants aware of their assigned intervention during the trial?	PY
	2.2.Were carers and people delivering the interventions aware of participants' assigned intervention during the trial?	PY
	2.3. If Y/PY/NI to 2.1 or 2.2: Were there deviations from the intended intervention that arose because of the experimental context?	PY
	2.4 If Y/PY to 2.3: Were these deviations likely to have affected the outcome?	PY
	2.5. If Y/PY/NI to 2.4: Were these deviations from intended intervention balanced between groups?	PY
	2.6 Was an appropriate analysis used to estimate the effect of assignment to intervention?	PN
	2.7 If N/PN/NI to 2.6: Was there potential for a substantial impact (on the result) of the failure to analyse participants in the group to which they were randomized?	PN
	**Risk of bias judgement**	**Some concerns**
**Bias due to missing outcome data**	3.1 Were data for this outcome available for all, or nearly all, participants randomized?	PN
	3.2 If N/PN/NI to 3.1: Is there evidence that result was not biased by missing outcome data?	PN
	3.3 If N/PN to 3.2: Could missingness in the outcome depend on its true value?	PY
	3.4 If Y/PY/NI to 3.3: Is it likely that missingness in the outcome depended on its true value?	PY
	**Risk of bias judgement**	**High**
**Bias in measurement of the outcome**	4.1 Was the method of measuring the outcome inappropriate?	PN
	4.2 Could measurement or ascertainment of the outcome have differed between intervention groups?	PN
	4.3 Were outcome assessors aware of the intervention received by study participants?	PY
	4.4 If Y/PY/NI to 4.3: Could assessment of the outcome have been influenced by knowledge of intervention received?	PN
	4.5 If Y/PY/NI to 4.4: Is it likely that assessment of the outcome was influenced by knowledge of intervention received?	NA
	**Risk of bias judgement**	**Low**
**Bias in selection of the reported result**	5.1 Were the data that produced this result analysed in accordance with a pre-specified analysis plan that was finalized before unblinded outcome data were available for analysis?	PN
	5.2 ... multiple eligible outcome measurements (e.g. scales, definitions, time points) within the outcome domain?	NI
	5.3 ... multiple eligible analyses of the data?	NI
	**Risk of bias judgement**	**Some concerns**
**Overall bias**	**Risk of bias judgement**	**High**

Study	Huber 2019	
**Domain**	**Signalling question**	**Response**
**Bias arising from the randomization process**	1.1 Was the allocation sequence random?	Y
	1.2 Was the allocation sequence concealed until participants were enrolled and assigned to interventions?	PY
	1.3 Did baseline differences between intervention groups suggest a problem with the randomization process?	PN
	**Risk of bias judgement**	**Low**
**Bias due to deviations from intended interventions**	2.1.Were participants aware of their assigned intervention during the trial?	PY
	2.2.Were carers and people delivering the interventions aware of participants' assigned intervention during the trial?	PY
	2.3. If Y/PY/NI to 2.1 or 2.2: Were there deviations from the intended intervention that arose because of the experimental context?	PY
	2.4 If Y/PY to 2.3: Were these deviations likely to have affected the outcome?	PY
	2.5. If Y/PY/NI to 2.4: Were these deviations from intended intervention balanced between groups?	PY
	2.6 Was an appropriate analysis used to estimate the effect of assignment to intervention?	PY
	2.7 If N/PN/NI to 2.6: Was there potential for a substantial impact (on the result) of the failure to analyse participants in the group to which they were randomized?	NA
	**Risk of bias judgement**	**Some concerns**
**Bias due to missing outcome data**	3.1 Were data for this outcome available for all, or nearly all, participants randomized?	PY
	3.2 If N/PN/NI to 3.1: Is there evidence that result was not biased by missing outcome data?	NA
	3.3 If N/PN to 3.2: Could missingness in the outcome depend on its true value?	NA
	3.4 If Y/PY/NI to 3.3: Is it likely that missingness in the outcome depended on its true value?	NA
	**Risk of bias judgement**	**Low**
**Bias in measurement of the outcome**	4.1 Was the method of measuring the outcome inappropriate?	PN
	4.2 Could measurement or ascertainment of the outcome have differed between intervention groups?	PN
	4.3 Were outcome assessors aware of the intervention received by study participants?	PY
	4.4 If Y/PY/NI to 4.3: Could assessment of the outcome have been influenced by knowledge of intervention received?	PY
	4.5 If Y/PY/NI to 4.4: Is it likely that assessment of the outcome was influenced by knowledge of intervention received?	PY
	**Risk of bias judgement**	**High**
**Bias in selection of the reported result**	5.1 Were the data that produced this result analysed in accordance with a pre-specified analysis plan that was finalized before unblinded outcome data were available for analysis?	PY
	5.2 ... multiple eligible outcome measurements (e.g. scales, definitions, time points) within the outcome domain?	NI
	5.3 ... multiple eligible analyses of the data?	NI
	**Risk of bias judgement**	**Some concerns**
**Overall bias**	**Risk of bias judgement**	**High**

Study	Gardner 2019	
**Domain**	**Signalling question**	**Response**
**Bias arising from the randomization process**	1.1 Was the allocation sequence random?	Y
	1.2 Was the allocation sequence concealed until participants were enrolled and assigned to interventions?	PY
	1.3 Did baseline differences between intervention groups suggest a problem with the randomization process?	PN
	**Risk of bias judgement**	**Low**
**Bias due to deviations from intended interventions**	2.1.Were participants aware of their assigned intervention during the trial?	PY
	2.2.Were carers and people delivering the interventions aware of participants' assigned intervention during the trial?	PN
	2.3. If Y/PY/NI to 2.1 or 2.2: Were there deviations from the intended intervention that arose because of the experimental context?	PN
	2.4 If Y/PY to 2.3: Were these deviations likely to have affected the outcome?	NA
	2.5. If Y/PY/NI to 2.4: Were these deviations from intended intervention balanced between groups?	NA
	2.6 Was an appropriate analysis used to estimate the effect of assignment to intervention?	PY
	2.7 If N/PN/NI to 2.6: Was there potential for a substantial impact (on the result) of the failure to analyse participants in the group to which they were randomized?	NA
	**Risk of bias judgement**	**Low**
**Bias due to missing outcome data**	3.1 Were data for this outcome available for all, or nearly all, participants randomized?	Y
	3.2 If N/PN/NI to 3.1: Is there evidence that result was not biased by missing outcome data?	NA
	3.3 If N/PN to 3.2: Could missingness in the outcome depend on its true value?	NA
	3.4 If Y/PY/NI to 3.3: Is it likely that missingness in the outcome depended on its true value?	NA
	**Risk of bias judgement**	**Low**
**Bias in measurement of the outcome**	4.1 Was the method of measuring the outcome inappropriate?	PN
	4.2 Could measurement or ascertainment of the outcome have differed between intervention groups?	PN
	4.3 Were outcome assessors aware of the intervention received by study participants?	PN
	4.4 If Y/PY/NI to 4.3: Could assessment of the outcome have been influenced by knowledge of intervention received?	NA
	4.5 If Y/PY/NI to 4.4: Is it likely that assessment of the outcome was influenced by knowledge of intervention received?	NA
	**Risk of bias judgement**	**Low**
**Bias in selection of the reported result**	5.1 Were the data that produced this result analysed in accordance with a pre-specified analysis plan that was finalized before unblinded outcome data were available for analysis?	PY
	5.2 ... multiple eligible outcome measurements (e.g. scales, definitions, time points) within the outcome domain?	PN
	5.3 ... multiple eligible analyses of the data?	PN
	**Risk of bias judgement**	**Low**
**Overall bias**	**Risk of bias judgement**	**Low**

Study	Kuvacic 2018	
**Domain**	**Signalling question**	**Response**
**Bias arising from the randomization process**	1.1 Was the allocation sequence random?	Y
	1.2 Was the allocation sequence concealed until participants were enrolled and assigned to interventions?	PY
	1.3 Did baseline differences between intervention groups suggest a problem with the randomization process?	PY
	**Risk of bias judgement**	**Some concerns**
**Bias due to deviations from intended interventions**	2.1.Were participants aware of their assigned intervention during the trial?	PY
	2.2.Were carers and people delivering the interventions aware of participants' assigned intervention during the trial?	PY
	2.3. If Y/PY/NI to 2.1 or 2.2: Were there deviations from the intended intervention that arose because of the experimental context?	PY
	2.4 If Y/PY to 2.3: Were these deviations likely to have affected the outcome?	PN
	2.5. If Y/PY/NI to 2.4: Were these deviations from intended intervention balanced between groups?	NA
	2.6 Was an appropriate analysis used to estimate the effect of assignment to intervention?	PY
	2.7 If N/PN/NI to 2.6: Was there potential for a substantial impact (on the result) of the failure to analyse participants in the group to which they were randomized?	NA
	**Risk of bias judgement**	**Some concerns**
**Bias due to missing outcome data**	3.1 Were data for this outcome available for all, or nearly all, participants randomized?	PY
	3.2 If N/PN/NI to 3.1: Is there evidence that result was not biased by missing outcome data?	NA
	3.3 If N/PN to 3.2: Could missingness in the outcome depend on its true value?	NA
	3.4 If Y/PY/NI to 3.3: Is it likely that missingness in the outcome depended on its true value?	NA
	**Risk of bias judgement**	**Low**
**Bias in measurement of the outcome**	4.1 Was the method of measuring the outcome inappropriate?	PN
	4.2 Could measurement or ascertainment of the outcome have differed between intervention groups?	PN
	4.3 Were outcome assessors aware of the intervention received by study participants?	PY
	4.4 If Y/PY/NI to 4.3: Could assessment of the outcome have been influenced by knowledge of intervention received?	PN
	4.5 If Y/PY/NI to 4.4: Is it likely that assessment of the outcome was influenced by knowledge of intervention received?	NA
	**Risk of bias judgement**	**Low**
**Bias in selection of the reported result**	5.1 Were the data that produced this result analysed in accordance with a pre-specified analysis plan that was finalized before unblinded outcome data were available for analysis?	PY
	5.2 ... multiple eligible outcome measurements (e.g. scales, definitions, time points) within the outcome domain?	NI
	5.3 ... multiple eligible analyses of the data?	NI
	**Risk of bias judgement**	**Some concerns**
**Overall bias**	**Risk of bias judgement**	**Some concerns**

Study	Hohmann 2018	
**Domain**	**Signalling question**	**Response**
**Bias arising from the randomization process**	1.1 Was the allocation sequence random?	Y
	1.2 Was the allocation sequence concealed until participants were enrolled and assigned to interventions?	PY
	1.3 Did baseline differences between intervention groups suggest a problem with the randomization process?	PY
	**Risk of bias judgement**	**Some concerns**
**Bias due to deviations from intended interventions**	2.1.Were participants aware of their assigned intervention during the trial?	Y
	2.2.Were carers and people delivering the interventions aware of participants' assigned intervention during the trial?	Y
	2.3. If Y/PY/NI to 2.1 or 2.2: Were there deviations from the intended intervention that arose because of the experimental context?	PY
	2.4 If Y/PY to 2.3: Were these deviations likely to have affected the outcome?	PN
	2.5. If Y/PY/NI to 2.4: Were these deviations from intended intervention balanced between groups?	NA
	2.6 Was an appropriate analysis used to estimate the effect of assignment to intervention?	PN
	2.7 If N/PN/NI to 2.6: Was there potential for a substantial impact (on the result) of the failure to analyse participants in the group to which they were randomized?	PY
	**Risk of bias judgement**	**High**
**Bias due to missing outcome data**	3.1 Were data for this outcome available for all, or nearly all, participants randomized?	PN
	3.2 If N/PN/NI to 3.1: Is there evidence that result was not biased by missing outcome data?	PN
	3.3 If N/PN to 3.2: Could missingness in the outcome depend on its true value?	PY
	3.4 If Y/PY/NI to 3.3: Is it likely that missingness in the outcome depended on its true value?	NI
	**Risk of bias judgement**	**High**
**Bias in measurement of the outcome**	4.1 Was the method of measuring the outcome inappropriate?	PN
	4.2 Could measurement or ascertainment of the outcome have differed between intervention groups?	PN
	4.3 Were outcome assessors aware of the intervention received by study participants?	Y
	4.4 If Y/PY/NI to 4.3: Could assessment of the outcome have been influenced by knowledge of intervention received?	PY
	4.5 If Y/PY/NI to 4.4: Is it likely that assessment of the outcome was influenced by knowledge of intervention received?	PN
	**Risk of bias judgement**	**Some concerns**
**Bias in selection of the reported result**	5.1 Were the data that produced this result analysed in accordance with a pre-specified analysis plan that was finalized before unblinded outcome data were available for analysis?	PY
	5.2 ... multiple eligible outcome measurements (e.g. scales, definitions, time points) within the outcome domain?	PY
	5.3 ... multiple eligible analyses of the data?	PY
	**Risk of bias judgement**	**High**
**Overall bias**	**Risk of bias judgement**	**High**

Study	Glombiewski 2018	
**Domain**	**Signalling question**	**Response**
**Bias arising from the randomization process**	1.1 Was the allocation sequence random?	Y
	1.2 Was the allocation sequence concealed until participants were enrolled and assigned to interventions?	PY
	1.3 Did baseline differences between intervention groups suggest a problem with the randomization process?	PY
	**Risk of bias judgement**	**Some concerns**
**Bias due to deviations from intended interventions**	2.1.Were participants aware of their assigned intervention during the trial?	Y
	2.2.Were carers and people delivering the interventions aware of participants' assigned intervention during the trial?	PN
	2.3. If Y/PY/NI to 2.1 or 2.2: Were there deviations from the intended intervention that arose because of the experimental context?	NI
	2.4 If Y/PY to 2.3: Were these deviations likely to have affected the outcome?	NA
	2.5. If Y/PY/NI to 2.4: Were these deviations from intended intervention balanced between groups?	NA
	2.6 Was an appropriate analysis used to estimate the effect of assignment to intervention?	PY
	2.7 If N/PN/NI to 2.6: Was there potential for a substantial impact (on the result) of the failure to analyse participants in the group to which they were randomized?	NA
	**Risk of bias judgement**	**Some concerns**
**Bias due to missing outcome data**	3.1 Were data for this outcome available for all, or nearly all, participants randomized?	PY
	3.2 If N/PN/NI to 3.1: Is there evidence that result was not biased by missing outcome data?	NA
	3.3 If N/PN to 3.2: Could missingness in the outcome depend on its true value?	NA
	3.4 If Y/PY/NI to 3.3: Is it likely that missingness in the outcome depended on its true value?	NA
	**Risk of bias judgement**	**Low**
**Bias in measurement of the outcome**	4.1 Was the method of measuring the outcome inappropriate?	PN
	4.2 Could measurement or ascertainment of the outcome have differed between intervention groups?	PN
	4.3 Were outcome assessors aware of the intervention received by study participants?	PN
	4.4 If Y/PY/NI to 4.3: Could assessment of the outcome have been influenced by knowledge of intervention received?	NA
	4.5 If Y/PY/NI to 4.4: Is it likely that assessment of the outcome was influenced by knowledge of intervention received?	NA
	**Risk of bias judgement**	**Low**
**Bias in selection of the reported result**	5.1 Were the data that produced this result analysed in accordance with a pre-specified analysis plan that was finalized before unblinded outcome data were available for analysis?	PY
	5.2 ... multiple eligible outcome measurements (e.g. scales, definitions, time points) within the outcome domain?	NI
	5.3 ... multiple eligible analyses of the data?	NI
	**Risk of bias judgement**	**Some concerns**
**Overall bias**	**Risk of bias judgement**	**Some concerns**

Study	Tüzün 2017	
**Domain**	**Signalling question**	**Response**
**Bias arising from the randomization process**	1.1 Was the allocation sequence random?	Y
	1.2 Was the allocation sequence concealed until participants were enrolled and assigned to interventions?	PY
	1.3 Did baseline differences between intervention groups suggest a problem with the randomization process?	PY
	**Risk of bias judgement**	**Some concerns**
**Bias due to deviations from intended interventions**	2.1.Were participants aware of their assigned intervention during the trial?	PY
	2.2.Were carers and people delivering the interventions aware of participants' assigned intervention during the trial?	PN
	2.3. If Y/PY/NI to 2.1 or 2.2: Were there deviations from the intended intervention that arose because of the experimental context?	PN
	2.4 If Y/PY to 2.3: Were these deviations likely to have affected the outcome?	NA
	2.5. If Y/PY/NI to 2.4: Were these deviations from intended intervention balanced between groups?	NA
	2.6 Was an appropriate analysis used to estimate the effect of assignment to intervention?	PY
	2.7 If N/PN/NI to 2.6: Was there potential for a substantial impact (on the result) of the failure to analyse participants in the group to which they were randomized?	NA
	**Risk of bias judgement**	**Low**
**Bias due to missing outcome data**	3.1 Were data for this outcome available for all, or nearly all, participants randomized?	Y
	3.2 If N/PN/NI to 3.1: Is there evidence that result was not biased by missing outcome data?	NA
	3.3 If N/PN to 3.2: Could missingness in the outcome depend on its true value?	NA
	3.4 If Y/PY/NI to 3.3: Is it likely that missingness in the outcome depended on its true value?	NA
	**Risk of bias judgement**	**Low**
**Bias in measurement of the outcome**	4.1 Was the method of measuring the outcome inappropriate?	PN
	4.2 Could measurement or ascertainment of the outcome have differed between intervention groups?	PN
	4.3 Were outcome assessors aware of the intervention received by study participants?	PY
	4.4 If Y/PY/NI to 4.3: Could assessment of the outcome have been influenced by knowledge of intervention received?	PN
	4.5 If Y/PY/NI to 4.4: Is it likely that assessment of the outcome was influenced by knowledge of intervention received?	NA
	**Risk of bias judgement**	**Low**
**Bias in selection of the reported result**	5.1 Were the data that produced this result analysed in accordance with a pre-specified analysis plan that was finalized before unblinded outcome data were available for analysis?	PY
	5.2 ... multiple eligible outcome measurements (e.g. scales, definitions, time points) within the outcome domain?	NI
	5.3 ... multiple eligible analyses of the data?	NI
	**Risk of bias judgement**	**Some concerns**
**Overall bias**	**Risk of bias judgement**	**Some concerns**

Study	Seo 2017	
**Domain**	**Signalling question**	**Response**
**Bias arising from the randomization process**	1.1 Was the allocation sequence random?	Y
	1.2 Was the allocation sequence concealed until participants were enrolled and assigned to interventions?	PY
	1.3 Did baseline differences between intervention groups suggest a problem with the randomization process?	PN
	**Risk of bias judgement**	**Low**
**Bias due to deviations from intended interventions**	2.1.Were participants aware of their assigned intervention during the trial?	PY
	2.2.Were carers and people delivering the interventions aware of participants' assigned intervention during the trial?	PN
	2.3. If Y/PY/NI to 2.1 or 2.2: Were there deviations from the intended intervention that arose because of the experimental context?	PN
	2.4 If Y/PY to 2.3: Were these deviations likely to have affected the outcome?	NA
	2.5. If Y/PY/NI to 2.4: Were these deviations from intended intervention balanced between groups?	NA
	2.6 Was an appropriate analysis used to estimate the effect of assignment to intervention?	Y
	2.7 If N/PN/NI to 2.6: Was there potential for a substantial impact (on the result) of the failure to analyse participants in the group to which they were randomized?	NA
	**Risk of bias judgement**	**Low**
**Bias due to missing outcome data**	3.1 Were data for this outcome available for all, or nearly all, participants randomized?	PY
	3.2 If N/PN/NI to 3.1: Is there evidence that result was not biased by missing outcome data?	NA
	3.3 If N/PN to 3.2: Could missingness in the outcome depend on its true value?	NA
	3.4 If Y/PY/NI to 3.3: Is it likely that missingness in the outcome depended on its true value?	NA
	**Risk of bias judgement**	**Low**
**Bias in measurement of the outcome**	4.1 Was the method of measuring the outcome inappropriate?	PN
	4.2 Could measurement or ascertainment of the outcome have differed between intervention groups?	PN
	4.3 Were outcome assessors aware of the intervention received by study participants?	PN
	4.4 If Y/PY/NI to 4.3: Could assessment of the outcome have been influenced by knowledge of intervention received?	NA
	4.5 If Y/PY/NI to 4.4: Is it likely that assessment of the outcome was influenced by knowledge of intervention received?	NA
	**Risk of bias judgement**	**Low**
**Bias in selection of the reported result**	5.1 Were the data that produced this result analysed in accordance with a pre-specified analysis plan that was finalized before unblinded outcome data were available for analysis?	PY
	5.2 ... multiple eligible outcome measurements (e.g. scales, definitions, time points) within the outcome domain?	PN
	5.3 ... multiple eligible analyses of the data?	PN
	**Risk of bias judgement**	**Low**
**Overall bias**	**Risk of bias judgement**	**Low**

Study	Nayback-Beebe 2017	
**Domain**	**Signalling question**	**Response**
**Bias arising from the randomization process**	1.1 Was the allocation sequence random?	Y
	1.2 Was the allocation sequence concealed until participants were enrolled and assigned to interventions?	PY
	1.3 Did baseline differences between intervention groups suggest a problem with the randomization process?	PY
	**Risk of bias judgement**	**Some concerns**
**Bias due to deviations from intended interventions**	2.1.Were participants aware of their assigned intervention during the trial?	Y
	2.2.Were carers and people delivering the interventions aware of participants' assigned intervention during the trial?	Y
	2.3. If Y/PY/NI to 2.1 or 2.2: Were there deviations from the intended intervention that arose because of the experimental context?	PY
	2.4 If Y/PY to 2.3: Were these deviations likely to have affected the outcome?	PY
	2.5. If Y/PY/NI to 2.4: Were these deviations from intended intervention balanced between groups?	PY
	2.6 Was an appropriate analysis used to estimate the effect of assignment to intervention?	PY
	2.7 If N/PN/NI to 2.6: Was there potential for a substantial impact (on the result) of the failure to analyse participants in the group to which they were randomized?	NA
	**Risk of bias judgement**	**Some concerns**
**Bias due to missing outcome data**	3.1 Were data for this outcome available for all, or nearly all, participants randomized?	NI
	3.2 If N/PN/NI to 3.1: Is there evidence that result was not biased by missing outcome data?	PY
	3.3 If N/PN to 3.2: Could missingness in the outcome depend on its true value?	NA
	3.4 If Y/PY/NI to 3.3: Is it likely that missingness in the outcome depended on its true value?	NA
	**Risk of bias judgement**	**Low**
**Bias in measurement of the outcome**	4.1 Was the method of measuring the outcome inappropriate?	PN
	4.2 Could measurement or ascertainment of the outcome have differed between intervention groups?	PN
	4.3 Were outcome assessors aware of the intervention received by study participants?	Y
	4.4 If Y/PY/NI to 4.3: Could assessment of the outcome have been influenced by knowledge of intervention received?	PY
	4.5 If Y/PY/NI to 4.4: Is it likely that assessment of the outcome was influenced by knowledge of intervention received?	PY
	**Risk of bias judgement**	**High**
**Bias in selection of the reported result**	5.1 Were the data that produced this result analysed in accordance with a pre-specified analysis plan that was finalized before unblinded outcome data were available for analysis?	PY
	5.2 ... multiple eligible outcome measurements (e.g. scales, definitions, time points) within the outcome domain?	NI
	5.3 ... multiple eligible analyses of the data?	NI
	**Risk of bias judgement**	**Some concerns**
**Overall bias**	**Risk of bias judgement**	**High**

Study	Kumar 2017	
**Domain**	**Signalling question**	**Response**
**Bias arising from the randomization process**	1.1 Was the allocation sequence random?	Y
	1.2 Was the allocation sequence concealed until participants were enrolled and assigned to interventions?	PY
	1.3 Did baseline differences between intervention groups suggest a problem with the randomization process?	PN
	**Risk of bias judgement**	**Low**
**Bias due to deviations from intended interventions**	2.1.Were participants aware of their assigned intervention during the trial?	PY
	2.2.Were carers and people delivering the interventions aware of participants' assigned intervention during the trial?	PY
	2.3. If Y/PY/NI to 2.1 or 2.2: Were there deviations from the intended intervention that arose because of the experimental context?	PN
	2.4 If Y/PY to 2.3: Were these deviations likely to have affected the outcome?	NA
	2.5. If Y/PY/NI to 2.4: Were these deviations from intended intervention balanced between groups?	NA
	2.6 Was an appropriate analysis used to estimate the effect of assignment to intervention?	PY
	2.7 If N/PN/NI to 2.6: Was there potential for a substantial impact (on the result) of the failure to analyse participants in the group to which they were randomized?	NA
	**Risk of bias judgement**	**Low**
**Bias due to missing outcome data**	3.1 Were data for this outcome available for all, or nearly all, participants randomized?	PN
	3.2 If N/PN/NI to 3.1: Is there evidence that result was not biased by missing outcome data?	PY
	3.3 If N/PN to 3.2: Could missingness in the outcome depend on its true value?	NA
	3.4 If Y/PY/NI to 3.3: Is it likely that missingness in the outcome depended on its true value?	NA
	**Risk of bias judgement**	**Low**
**Bias in measurement of the outcome**	4.1 Was the method of measuring the outcome inappropriate?	PN
	4.2 Could measurement or ascertainment of the outcome have differed between intervention groups?	PN
	4.3 Were outcome assessors aware of the intervention received by study participants?	PY
	4.4 If Y/PY/NI to 4.3: Could assessment of the outcome have been influenced by knowledge of intervention received?	PY
	4.5 If Y/PY/NI to 4.4: Is it likely that assessment of the outcome was influenced by knowledge of intervention received?	PN
	**Risk of bias judgement**	**Some concerns**
**Bias in selection of the reported result**	5.1 Were the data that produced this result analysed in accordance with a pre-specified analysis plan that was finalized before unblinded outcome data were available for analysis?	PY
	5.2 ... multiple eligible outcome measurements (e.g. scales, definitions, time points) within the outcome domain?	NI
	5.3 ... multiple eligible analyses of the data?	NI
	**Risk of bias judgement**	**Some concerns**
**Overall bias**	**Risk of bias judgement**	**Some concerns**

Study	Harris 2017	
**Domain**	**Signalling question**	**Response**
**Bias arising from the randomization process**	1.1 Was the allocation sequence random?	Y
	1.2 Was the allocation sequence concealed until participants were enrolled and assigned to interventions?	PY
	1.3 Did baseline differences between intervention groups suggest a problem with the randomization process?	PN
	**Risk of bias judgement**	**Low**
**Bias due to deviations from intended interventions**	2.1.Were participants aware of their assigned intervention during the trial?	PY
	2.2.Were carers and people delivering the interventions aware of participants' assigned intervention during the trial?	PN
	2.3. If Y/PY/NI to 2.1 or 2.2: Were there deviations from the intended intervention that arose because of the experimental context?	PY
	2.4 If Y/PY to 2.3: Were these deviations likely to have affected the outcome?	PN
	2.5. If Y/PY/NI to 2.4: Were these deviations from intended intervention balanced between groups?	NA
	2.6 Was an appropriate analysis used to estimate the effect of assignment to intervention?	PY
	2.7 If N/PN/NI to 2.6: Was there potential for a substantial impact (on the result) of the failure to analyse participants in the group to which they were randomized?	NA
	**Risk of bias judgement**	**Some concerns**
**Bias due to missing outcome data**	3.1 Were data for this outcome available for all, or nearly all, participants randomized?	PN
	3.2 If N/PN/NI to 3.1: Is there evidence that result was not biased by missing outcome data?	PN
	3.3 If N/PN to 3.2: Could missingness in the outcome depend on its true value?	PY
	3.4 If Y/PY/NI to 3.3: Is it likely that missingness in the outcome depended on its true value?	PN
	**Risk of bias judgement**	**Some concerns**
**Bias in measurement of the outcome**	4.1 Was the method of measuring the outcome inappropriate?	PN
	4.2 Could measurement or ascertainment of the outcome have differed between intervention groups?	PN
	4.3 Were outcome assessors aware of the intervention received by study participants?	PY
	4.4 If Y/PY/NI to 4.3: Could assessment of the outcome have been influenced by knowledge of intervention received?	PY
	4.5 If Y/PY/NI to 4.4: Is it likely that assessment of the outcome was influenced by knowledge of intervention received?	PY
	**Risk of bias judgement**	**High**
**Bias in selection of the reported result**	5.1 Were the data that produced this result analysed in accordance with a pre-specified analysis plan that was finalized before unblinded outcome data were available for analysis?	PY
	5.2 ... multiple eligible outcome measurements (e.g. scales, definitions, time points) within the outcome domain?	NI
	5.3 ... multiple eligible analyses of the data?	NI
	**Risk of bias judgement**	**Some concerns**
**Overall bias**	**Risk of bias judgement**	**High**

Study	Michalsen 2016	
**Domain**	**Signalling question**	**Response**
**Bias arising from the randomization process**	1.1 Was the allocation sequence random?	Y
	1.2 Was the allocation sequence concealed until participants were enrolled and assigned to interventions?	Y
	1.3 Did baseline differences between intervention groups suggest a problem with the randomization process?	PN
	**Risk of bias judgement**	**Low**
**Bias due to deviations from intended interventions**	2.1.Were participants aware of their assigned intervention during the trial?	Y
	2.2.Were carers and people delivering the interventions aware of participants' assigned intervention during the trial?	PY
	2.3. If Y/PY/NI to 2.1 or 2.2: Were there deviations from the intended intervention that arose because of the experimental context?	PY
	2.4 If Y/PY to 2.3: Were these deviations likely to have affected the outcome?	PY
	2.5. If Y/PY/NI to 2.4: Were these deviations from intended intervention balanced between groups?	PY
	2.6 Was an appropriate analysis used to estimate the effect of assignment to intervention?	PY
	2.7 If N/PN/NI to 2.6: Was there potential for a substantial impact (on the result) of the failure to analyse participants in the group to which they were randomized?	NA
	**Risk of bias judgement**	**Some concerns**
**Bias due to missing outcome data**	3.1 Were data for this outcome available for all, or nearly all, participants randomized?	PN
	3.2 If N/PN/NI to 3.1: Is there evidence that result was not biased by missing outcome data?	PN
	3.3 If N/PN to 3.2: Could missingness in the outcome depend on its true value?	PY
	3.4 If Y/PY/NI to 3.3: Is it likely that missingness in the outcome depended on its true value?	PN
	**Risk of bias judgement**	**Some concerns**
**Bias in measurement of the outcome**	4.1 Was the method of measuring the outcome inappropriate?	PN
	4.2 Could measurement or ascertainment of the outcome have differed between intervention groups?	PN
	4.3 Were outcome assessors aware of the intervention received by study participants?	PY
	4.4 If Y/PY/NI to 4.3: Could assessment of the outcome have been influenced by knowledge of intervention received?	PY
	4.5 If Y/PY/NI to 4.4: Is it likely that assessment of the outcome was influenced by knowledge of intervention received?	PN
	**Risk of bias judgement**	**Some concerns**
**Bias in selection of the reported result**	5.1 Were the data that produced this result analysed in accordance with a pre-specified analysis plan that was finalized before unblinded outcome data were available for analysis?	PY
	5.2 ... multiple eligible outcome measurements (e.g. scales, definitions, time points) within the outcome domain?	PN
	5.3 ... multiple eligible analyses of the data?	PN
	**Risk of bias judgement**	**Low**
**Overall bias**	**Risk of bias judgement**	**Some concerns**

Study	Trapp 2015	
**Domain**	**Signalling question**	**Response**
**Bias arising from the randomization process**	1.1 Was the allocation sequence random?	Y
	1.2 Was the allocation sequence concealed until participants were enrolled and assigned to interventions?	PY
	1.3 Did baseline differences between intervention groups suggest a problem with the randomization process?	PY
	**Risk of bias judgement**	**Some concerns**
**Bias due to deviations from intended interventions**	2.1.Were participants aware of their assigned intervention during the trial?	PY
	2.2.Were carers and people delivering the interventions aware of participants' assigned intervention during the trial?	PY
	2.3. If Y/PY/NI to 2.1 or 2.2: Were there deviations from the intended intervention that arose because of the experimental context?	PY
	2.4 If Y/PY to 2.3: Were these deviations likely to have affected the outcome?	PY
	2.5. If Y/PY/NI to 2.4: Were these deviations from intended intervention balanced between groups?	PN
	2.6 Was an appropriate analysis used to estimate the effect of assignment to intervention?	PY
	2.7 If N/PN/NI to 2.6: Was there potential for a substantial impact (on the result) of the failure to analyse participants in the group to which they were randomized?	NA
	**Risk of bias judgement**	**High**
**Bias due to missing outcome data**	3.1 Were data for this outcome available for all, or nearly all, participants randomized?	PY
	3.2 If N/PN/NI to 3.1: Is there evidence that result was not biased by missing outcome data?	NA
	3.3 If N/PN to 3.2: Could missingness in the outcome depend on its true value?	NA
	3.4 If Y/PY/NI to 3.3: Is it likely that missingness in the outcome depended on its true value?	NA
	**Risk of bias judgement**	**Low**
**Bias in measurement of the outcome**	4.1 Was the method of measuring the outcome inappropriate?	PN
	4.2 Could measurement or ascertainment of the outcome have differed between intervention groups?	PN
	4.3 Were outcome assessors aware of the intervention received by study participants?	PY
	4.4 If Y/PY/NI to 4.3: Could assessment of the outcome have been influenced by knowledge of intervention received?	NI
	4.5 If Y/PY/NI to 4.4: Is it likely that assessment of the outcome was influenced by knowledge of intervention received?	NI
	**Risk of bias judgement**	**High**
**Bias in selection of the reported result**	5.1 Were the data that produced this result analysed in accordance with a pre-specified analysis plan that was finalized before unblinded outcome data were available for analysis?	PY
	5.2 ... multiple eligible outcome measurements (e.g. scales, definitions, time points) within the outcome domain?	PN
	5.3 ... multiple eligible analyses of the data?	PN
	**Risk of bias judgement**	**Low**
**Overall bias**	**Risk of bias judgement**	**High**

Study	Lawand 2015	
**Domain**	**Signalling question**	**Response**
**Bias arising from the randomization process**	1.1 Was the allocation sequence random?	Y
	1.2 Was the allocation sequence concealed until participants were enrolled and assigned to interventions?	PY
	1.3 Did baseline differences between intervention groups suggest a problem with the randomization process?	PN
	**Risk of bias judgement**	**Low**
**Bias due to deviations from intended interventions**	2.1.Were participants aware of their assigned intervention during the trial?	PY
	2.2.Were carers and people delivering the interventions aware of participants' assigned intervention during the trial?	PN
	2.3. If Y/PY/NI to 2.1 or 2.2: Were there deviations from the intended intervention that arose because of the experimental context?	PN
	2.4 If Y/PY to 2.3: Were these deviations likely to have affected the outcome?	NA
	2.5. If Y/PY/NI to 2.4: Were these deviations from intended intervention balanced between groups?	NA
	2.6 Was an appropriate analysis used to estimate the effect of assignment to intervention?	PY
	2.7 If N/PN/NI to 2.6: Was there potential for a substantial impact (on the result) of the failure to analyse participants in the group to which they were randomized?	NA
	**Risk of bias judgement**	**Low**
**Bias due to missing outcome data**	3.1 Were data for this outcome available for all, or nearly all, participants randomized?	PY
	3.2 If N/PN/NI to 3.1: Is there evidence that result was not biased by missing outcome data?	NA
	3.3 If N/PN to 3.2: Could missingness in the outcome depend on its true value?	NA
	3.4 If Y/PY/NI to 3.3: Is it likely that missingness in the outcome depended on its true value?	NA
	**Risk of bias judgement**	**Low**
**Bias in measurement of the outcome**	4.1 Was the method of measuring the outcome inappropriate?	PN
	4.2 Could measurement or ascertainment of the outcome have differed between intervention groups?	PN
	4.3 Were outcome assessors aware of the intervention received by study participants?	PN
	4.4 If Y/PY/NI to 4.3: Could assessment of the outcome have been influenced by knowledge of intervention received?	NA
	4.5 If Y/PY/NI to 4.4: Is it likely that assessment of the outcome was influenced by knowledge of intervention received?	NA
	**Risk of bias judgement**	**Low**
**Bias in selection of the reported result**	5.1 Were the data that produced this result analysed in accordance with a pre-specified analysis plan that was finalized before unblinded outcome data were available for analysis?	PY
	5.2 ... multiple eligible outcome measurements (e.g. scales, definitions, time points) within the outcome domain?	PN
	5.3 ... multiple eligible analyses of the data?	PN
	**Risk of bias judgement**	**Low**
**Overall bias**	**Risk of bias judgement**	**Low**

Study	Kogure 2015	
**Domain**	**Signalling question**	**Response**
**Bias arising from the randomization process**	1.1 Was the allocation sequence random?	Y
	1.2 Was the allocation sequence concealed until participants were enrolled and assigned to interventions?	PY
	1.3 Did baseline differences between intervention groups suggest a problem with the randomization process?	PN
	**Risk of bias judgement**	**Low**
**Bias due to deviations from intended interventions**	2.1.Were participants aware of their assigned intervention during the trial?	N
	2.2.Were carers and people delivering the interventions aware of participants' assigned intervention during the trial?	Y
	2.3. If Y/PY/NI to 2.1 or 2.2: Were there deviations from the intended intervention that arose because of the experimental context?	PN
	2.4 If Y/PY to 2.3: Were these deviations likely to have affected the outcome?	NA
	2.5. If Y/PY/NI to 2.4: Were these deviations from intended intervention balanced between groups?	NA
	2.6 Was an appropriate analysis used to estimate the effect of assignment to intervention?	PY
	2.7 If N/PN/NI to 2.6: Was there potential for a substantial impact (on the result) of the failure to analyse participants in the group to which they were randomized?	NA
	**Risk of bias judgement**	**Low**
**Bias due to missing outcome data**	3.1 Were data for this outcome available for all, or nearly all, participants randomized?	PN
	3.2 If N/PN/NI to 3.1: Is there evidence that result was not biased by missing outcome data?	PY
	3.3 If N/PN to 3.2: Could missingness in the outcome depend on its true value?	NA
	3.4 If Y/PY/NI to 3.3: Is it likely that missingness in the outcome depended on its true value?	NA
	**Risk of bias judgement**	**Low**
**Bias in measurement of the outcome**	4.1 Was the method of measuring the outcome inappropriate?	PN
	4.2 Could measurement or ascertainment of the outcome have differed between intervention groups?	PN
	4.3 Were outcome assessors aware of the intervention received by study participants?	PY
	4.4 If Y/PY/NI to 4.3: Could assessment of the outcome have been influenced by knowledge of intervention received?	PY
	4.5 If Y/PY/NI to 4.4: Is it likely that assessment of the outcome was influenced by knowledge of intervention received?	PN
	**Risk of bias judgement**	**Some concerns**
**Bias in selection of the reported result**	5.1 Were the data that produced this result analysed in accordance with a pre-specified analysis plan that was finalized before unblinded outcome data were available for analysis?	PY
	5.2 ... multiple eligible outcome measurements (e.g. scales, definitions, time points) within the outcome domain?	NI
	5.3 ... multiple eligible analyses of the data?	NI
	**Risk of bias judgement**	**Some concerns**
**Overall bias**	**Risk of bias judgement**	**Some concerns**

Study	Zhang 2014	
**Domain**	**Signalling question**	**Response**
**Bias arising from the randomization process**	1.1 Was the allocation sequence random?	Y
	1.2 Was the allocation sequence concealed until participants were enrolled and assigned to interventions?	Y
	1.3 Did baseline differences between intervention groups suggest a problem with the randomization process?	PN
	**Risk of bias judgement**	**Low**
**Bias due to deviations from intended interventions**	2.1.Were participants aware of their assigned intervention during the trial?	PY
	2.2.Were carers and people delivering the interventions aware of participants' assigned intervention during the trial?	PY
	2.3. If Y/PY/NI to 2.1 or 2.2: Were there deviations from the intended intervention that arose because of the experimental context?	PY
	2.4 If Y/PY to 2.3: Were these deviations likely to have affected the outcome?	PY
	2.5. If Y/PY/NI to 2.4: Were these deviations from intended intervention balanced between groups?	PY
	2.6 Was an appropriate analysis used to estimate the effect of assignment to intervention?	PY
	2.7 If N/PN/NI to 2.6: Was there potential for a substantial impact (on the result) of the failure to analyse participants in the group to which they were randomized?	NA
	**Risk of bias judgement**	**Some concerns**
**Bias due to missing outcome data**	3.1 Were data for this outcome available for all, or nearly all, participants randomized?	PY
	3.2 If N/PN/NI to 3.1: Is there evidence that result was not biased by missing outcome data?	NA
	3.3 If N/PN to 3.2: Could missingness in the outcome depend on its true value?	NA
	3.4 If Y/PY/NI to 3.3: Is it likely that missingness in the outcome depended on its true value?	NA
	**Risk of bias judgement**	**Low**
**Bias in measurement of the outcome**	4.1 Was the method of measuring the outcome inappropriate?	PN
	4.2 Could measurement or ascertainment of the outcome have differed between intervention groups?	PN
	4.3 Were outcome assessors aware of the intervention received by study participants?	PY
	4.4 If Y/PY/NI to 4.3: Could assessment of the outcome have been influenced by knowledge of intervention received?	PY
	4.5 If Y/PY/NI to 4.4: Is it likely that assessment of the outcome was influenced by knowledge of intervention received?	PY
	**Risk of bias judgement**	**High**
**Bias in selection of the reported result**	5.1 Were the data that produced this result analysed in accordance with a pre-specified analysis plan that was finalized before unblinded outcome data were available for analysis?	PY
	5.2 ... multiple eligible outcome measurements (e.g. scales, definitions, time points) within the outcome domain?	PY
	5.3 ... multiple eligible analyses of the data?	PY
	**Risk of bias judgement**	**High**
**Overall bias**	**Risk of bias judgement**	**High**

Study	Monticone 2014	
**Domain**	**Signalling question**	**Response**
**Bias arising from the randomization process**	1.1 Was the allocation sequence random?	Y
	1.2 Was the allocation sequence concealed until participants were enrolled and assigned to interventions?	PY
	1.3 Did baseline differences between intervention groups suggest a problem with the randomization process?	PN
	**Risk of bias judgement**	**Low**
**Bias due to deviations from intended interventions**	2.1.Were participants aware of their assigned intervention during the trial?	Y
	2.2.Were carers and people delivering the interventions aware of participants' assigned intervention during the trial?	Y
	2.3. If Y/PY/NI to 2.1 or 2.2: Were there deviations from the intended intervention that arose because of the experimental context?	PY
	2.4 If Y/PY to 2.3: Were these deviations likely to have affected the outcome?	PY
	2.5. If Y/PY/NI to 2.4: Were these deviations from intended intervention balanced between groups?	PY
	2.6 Was an appropriate analysis used to estimate the effect of assignment to intervention?	PY
	2.7 If N/PN/NI to 2.6: Was there potential for a substantial impact (on the result) of the failure to analyse participants in the group to which they were randomized?	NA
	**Risk of bias judgement**	**Some concerns**
**Bias due to missing outcome data**	3.1 Were data for this outcome available for all, or nearly all, participants randomized?	PY
	3.2 If N/PN/NI to 3.1: Is there evidence that result was not biased by missing outcome data?	NA
	3.3 If N/PN to 3.2: Could missingness in the outcome depend on its true value?	NA
	3.4 If Y/PY/NI to 3.3: Is it likely that missingness in the outcome depended on its true value?	NA
	**Risk of bias judgement**	**Low**
**Bias in measurement of the outcome**	4.1 Was the method of measuring the outcome inappropriate?	PN
	4.2 Could measurement or ascertainment of the outcome have differed between intervention groups?	PN
	4.3 Were outcome assessors aware of the intervention received by study participants?	Y
	4.4 If Y/PY/NI to 4.3: Could assessment of the outcome have been influenced by knowledge of intervention received?	PY
	4.5 If Y/PY/NI to 4.4: Is it likely that assessment of the outcome was influenced by knowledge of intervention received?	PN
	**Risk of bias judgement**	**Some concerns**
**Bias in selection of the reported result**	5.1 Were the data that produced this result analysed in accordance with a pre-specified analysis plan that was finalized before unblinded outcome data were available for analysis?	Y
	5.2 ... multiple eligible outcome measurements (e.g. scales, definitions, time points) within the outcome domain?	NI
	5.3 ... multiple eligible analyses of the data?	NI
	**Risk of bias judgement**	**Some concerns**
**Overall bias**	**Risk of bias judgement**	**Some concerns**

Study	Tekur 2012	
**Domain**	**Signalling question**	**Response**
**Bias arising from the randomization process**	1.1 Was the allocation sequence random?	Y
	1.2 Was the allocation sequence concealed until participants were enrolled and assigned to interventions?	PY
	1.3 Did baseline differences between intervention groups suggest a problem with the randomization process?	PN
	**Risk of bias judgement**	**Low**
**Bias due to deviations from intended interventions**	2.1.Were participants aware of their assigned intervention during the trial?	PY
	2.2.Were carers and people delivering the interventions aware of participants' assigned intervention during the trial?	N
	2.3. If Y/PY/NI to 2.1 or 2.2: Were there deviations from the intended intervention that arose because of the experimental context?	PN
	2.4 If Y/PY to 2.3: Were these deviations likely to have affected the outcome?	NA
	2.5. If Y/PY/NI to 2.4: Were these deviations from intended intervention balanced between groups?	NA
	2.6 Was an appropriate analysis used to estimate the effect of assignment to intervention?	PY
	2.7 If N/PN/NI to 2.6: Was there potential for a substantial impact (on the result) of the failure to analyse participants in the group to which they were randomized?	NA
	**Risk of bias judgement**	**Low**
**Bias due to missing outcome data**	3.1 Were data for this outcome available for all, or nearly all, participants randomized?	Y
	3.2 If N/PN/NI to 3.1: Is there evidence that result was not biased by missing outcome data?	NA
	3.3 If N/PN to 3.2: Could missingness in the outcome depend on its true value?	NA
	3.4 If Y/PY/NI to 3.3: Is it likely that missingness in the outcome depended on its true value?	NA
	**Risk of bias judgement**	**Low**
**Bias in measurement of the outcome**	4.1 Was the method of measuring the outcome inappropriate?	PN
	4.2 Could measurement or ascertainment of the outcome have differed between intervention groups?	PN
	4.3 Were outcome assessors aware of the intervention received by study participants?	N
	4.4 If Y/PY/NI to 4.3: Could assessment of the outcome have been influenced by knowledge of intervention received?	NA
	4.5 If Y/PY/NI to 4.4: Is it likely that assessment of the outcome was influenced by knowledge of intervention received?	NA
	**Risk of bias judgement**	**Low**
**Bias in selection of the reported result**	5.1 Were the data that produced this result analysed in accordance with a pre-specified analysis plan that was finalized before unblinded outcome data were available for analysis?	PY
	5.2 ... multiple eligible outcome measurements (e.g. scales, definitions, time points) within the outcome domain?	PN
	5.3 ... multiple eligible analyses of the data?	PN
	**Risk of bias judgement**	**Low**
**Overall bias**	**Risk of bias judgement**	**Low**

Study	Kader 2012	
**Domain**	**Signalling question**	**Response**
**Bias arising from the randomization process**	1.1 Was the allocation sequence random?	Y
	1.2 Was the allocation sequence concealed until participants were enrolled and assigned to interventions?	PY
	1.3 Did baseline differences between intervention groups suggest a problem with the randomization process?	PY
	**Risk of bias judgement**	**Some concerns**
**Bias due to deviations from intended interventions**	2.1.Were participants aware of their assigned intervention during the trial?	PY
	2.2.Were carers and people delivering the interventions aware of participants' assigned intervention during the trial?	PY
	2.3. If Y/PY/NI to 2.1 or 2.2: Were there deviations from the intended intervention that arose because of the experimental context?	PY
	2.4 If Y/PY to 2.3: Were these deviations likely to have affected the outcome?	PY
	2.5. If Y/PY/NI to 2.4: Were these deviations from intended intervention balanced between groups?	PN
	2.6 Was an appropriate analysis used to estimate the effect of assignment to intervention?	PY
	2.7 If N/PN/NI to 2.6: Was there potential for a substantial impact (on the result) of the failure to analyse participants in the group to which they were randomized?	NA
	**Risk of bias judgement**	**High**
**Bias due to missing outcome data**	3.1 Were data for this outcome available for all, or nearly all, participants randomized?	PN
	3.2 If N/PN/NI to 3.1: Is there evidence that result was not biased by missing outcome data?	PN
	3.3 If N/PN to 3.2: Could missingness in the outcome depend on its true value?	PY
	3.4 If Y/PY/NI to 3.3: Is it likely that missingness in the outcome depended on its true value?	PN
	**Risk of bias judgement**	**Some concerns**
**Bias in measurement of the outcome**	4.1 Was the method of measuring the outcome inappropriate?	PN
	4.2 Could measurement or ascertainment of the outcome have differed between intervention groups?	PN
	4.3 Were outcome assessors aware of the intervention received by study participants?	PY
	4.4 If Y/PY/NI to 4.3: Could assessment of the outcome have been influenced by knowledge of intervention received?	PY
	4.5 If Y/PY/NI to 4.4: Is it likely that assessment of the outcome was influenced by knowledge of intervention received?	PY
	**Risk of bias judgement**	**High**
**Bias in selection of the reported result**	5.1 Were the data that produced this result analysed in accordance with a pre-specified analysis plan that was finalized before unblinded outcome data were available for analysis?	PY
	5.2 ... multiple eligible outcome measurements (e.g. scales, definitions, time points) within the outcome domain?	NI
	5.3 ... multiple eligible analyses of the data?	NI
	**Risk of bias judgement**	**Some concerns**
**Overall bias**	**Risk of bias judgement**	**High**

Study	Cuesta-Vargas 2012	
**Domain**	**Signalling question**	**Response**
**Bias arising from the randomization process**	1.1 Was the allocation sequence random?	Y
	1.2 Was the allocation sequence concealed until participants were enrolled and assigned to interventions?	PY
	1.3 Did baseline differences between intervention groups suggest a problem with the randomization process?	PN
	**Risk of bias judgement**	**Low**
**Bias due to deviations from intended interventions**	2.1.Were participants aware of their assigned intervention during the trial?	PY
	2.2.Were carers and people delivering the interventions aware of participants' assigned intervention during the trial?	PY
	2.3. If Y/PY/NI to 2.1 or 2.2: Were there deviations from the intended intervention that arose because of the experimental context?	PN
	2.4 If Y/PY to 2.3: Were these deviations likely to have affected the outcome?	NA
	2.5. If Y/PY/NI to 2.4: Were these deviations from intended intervention balanced between groups?	NA
	2.6 Was an appropriate analysis used to estimate the effect of assignment to intervention?	PY
	2.7 If N/PN/NI to 2.6: Was there potential for a substantial impact (on the result) of the failure to analyse participants in the group to which they were randomized?	NA
	**Risk of bias judgement**	**Low**
**Bias due to missing outcome data**	3.1 Were data for this outcome available for all, or nearly all, participants randomized?	PY
	3.2 If N/PN/NI to 3.1: Is there evidence that result was not biased by missing outcome data?	NA
	3.3 If N/PN to 3.2: Could missingness in the outcome depend on its true value?	NA
	3.4 If Y/PY/NI to 3.3: Is it likely that missingness in the outcome depended on its true value?	NA
	**Risk of bias judgement**	**Low**
**Bias in measurement of the outcome**	4.1 Was the method of measuring the outcome inappropriate?	PN
	4.2 Could measurement or ascertainment of the outcome have differed between intervention groups?	PN
	4.3 Were outcome assessors aware of the intervention received by study participants?	PY
	4.4 If Y/PY/NI to 4.3: Could assessment of the outcome have been influenced by knowledge of intervention received?	PN
	4.5 If Y/PY/NI to 4.4: Is it likely that assessment of the outcome was influenced by knowledge of intervention received?	NA
	**Risk of bias judgement**	**Low**
**Bias in selection of the reported result**	5.1 Were the data that produced this result analysed in accordance with a pre-specified analysis plan that was finalized before unblinded outcome data were available for analysis?	PY
	5.2 ... multiple eligible outcome measurements (e.g. scales, definitions, time points) within the outcome domain?	PN
	5.3 ... multiple eligible analyses of the data?	PN
	**Risk of bias judgement**	**Low**
**Overall bias**	**Risk of bias judgement**	**Low**

Study	Tavafian 2011	
**Domain**	**Signalling question**	**Response**
**Bias arising from the randomization process**	1.1 Was the allocation sequence random?	Y
	1.2 Was the allocation sequence concealed until participants were enrolled and assigned to interventions?	Y
	1.3 Did baseline differences between intervention groups suggest a problem with the randomization process?	N
	**Risk of bias judgement**	**Low**
**Bias due to deviations from intended interventions**	2.1.Were participants aware of their assigned intervention during the trial?	Y
	2.2.Were carers and people delivering the interventions aware of participants' assigned intervention during the trial?	N
	2.3. If Y/PY/NI to 2.1 or 2.2: Were there deviations from the intended intervention that arose because of the experimental context?	PN
	2.4 If Y/PY to 2.3: Were these deviations likely to have affected the outcome?	NA
	2.5. If Y/PY/NI to 2.4: Were these deviations from intended intervention balanced between groups?	NA
	2.6 Was an appropriate analysis used to estimate the effect of assignment to intervention?	PY
	2.7 If N/PN/NI to 2.6: Was there potential for a substantial impact (on the result) of the failure to analyse participants in the group to which they were randomized?	NA
	**Risk of bias judgement**	**Low**
**Bias due to missing outcome data**	3.1 Were data for this outcome available for all, or nearly all, participants randomized?	PN
	3.2 If N/PN/NI to 3.1: Is there evidence that result was not biased by missing outcome data?	PY
	3.3 If N/PN to 3.2: Could missingness in the outcome depend on its true value?	NA
	3.4 If Y/PY/NI to 3.3: Is it likely that missingness in the outcome depended on its true value?	NA
	**Risk of bias judgement**	**Low**
**Bias in measurement of the outcome**	4.1 Was the method of measuring the outcome inappropriate?	PN
	4.2 Could measurement or ascertainment of the outcome have differed between intervention groups?	PN
	4.3 Were outcome assessors aware of the intervention received by study participants?	N
	4.4 If Y/PY/NI to 4.3: Could assessment of the outcome have been influenced by knowledge of intervention received?	NA
	4.5 If Y/PY/NI to 4.4: Is it likely that assessment of the outcome was influenced by knowledge of intervention received?	NA
	**Risk of bias judgement**	**Low**
**Bias in selection of the reported result**	5.1 Were the data that produced this result analysed in accordance with a pre-specified analysis plan that was finalized before unblinded outcome data were available for analysis?	PY
	5.2 ... multiple eligible outcome measurements (e.g. scales, definitions, time points) within the outcome domain?	NI
	5.3 ... multiple eligible analyses of the data?	NI
	**Risk of bias judgement**	**Some concerns**
**Overall bias**	**Risk of bias judgement**	**Some concerns**

Study	Engbert 2011	
**Domain**	**Signalling question**	**Response**
**Bias arising from the randomization process**	1.1 Was the allocation sequence random?	Y
	1.2 Was the allocation sequence concealed until participants were enrolled and assigned to interventions?	PY
	1.3 Did baseline differences between intervention groups suggest a problem with the randomization process?	PN
	**Risk of bias judgement**	**Low**
**Bias due to deviations from intended interventions**	2.1.Were participants aware of their assigned intervention during the trial?	PY
	2.2.Were carers and people delivering the interventions aware of participants' assigned intervention during the trial?	PN
	2.3. If Y/PY/NI to 2.1 or 2.2: Were there deviations from the intended intervention that arose because of the experimental context?	PN
	2.4 If Y/PY to 2.3: Were these deviations likely to have affected the outcome?	NA
	2.5. If Y/PY/NI to 2.4: Were these deviations from intended intervention balanced between groups?	NA
	2.6 Was an appropriate analysis used to estimate the effect of assignment to intervention?	PY
	2.7 If N/PN/NI to 2.6: Was there potential for a substantial impact (on the result) of the failure to analyse participants in the group to which they were randomized?	NA
	**Risk of bias judgement**	**Low**
**Bias due to missing outcome data**	3.1 Were data for this outcome available for all, or nearly all, participants randomized?	PN
	3.2 If N/PN/NI to 3.1: Is there evidence that result was not biased by missing outcome data?	PY
	3.3 If N/PN to 3.2: Could missingness in the outcome depend on its true value?	NA
	3.4 If Y/PY/NI to 3.3: Is it likely that missingness in the outcome depended on its true value?	NA
	**Risk of bias judgement**	**Low**
**Bias in measurement of the outcome**	4.1 Was the method of measuring the outcome inappropriate?	PN
	4.2 Could measurement or ascertainment of the outcome have differed between intervention groups?	PN
	4.3 Were outcome assessors aware of the intervention received by study participants?	PY
	4.4 If Y/PY/NI to 4.3: Could assessment of the outcome have been influenced by knowledge of intervention received?	PN
	4.5 If Y/PY/NI to 4.4: Is it likely that assessment of the outcome was influenced by knowledge of intervention received?	NA
	**Risk of bias judgement**	**Low**
**Bias in selection of the reported result**	5.1 Were the data that produced this result analysed in accordance with a pre-specified analysis plan that was finalized before unblinded outcome data were available for analysis?	PY
	5.2 ... multiple eligible outcome measurements (e.g. scales, definitions, time points) within the outcome domain?	PN
	5.3 ... multiple eligible analyses of the data?	PN
	**Risk of bias judgement**	**Low**
**Overall bias**	**Risk of bias judgement**	**Low**

Study	Glombiewski 2010	
**Domain**	**Signalling question**	**Response**
**Bias arising from the randomization process**	1.1 Was the allocation sequence random?	Y
	1.2 Was the allocation sequence concealed until participants were enrolled and assigned to interventions?	PY
	1.3 Did baseline differences between intervention groups suggest a problem with the randomization process?	PN
	**Risk of bias judgement**	**Low**
**Bias due to deviations from intended interventions**	2.1.Were participants aware of their assigned intervention during the trial?	PY
	2.2.Were carers and people delivering the interventions aware of participants' assigned intervention during the trial?	PY
	2.3. If Y/PY/NI to 2.1 or 2.2: Were there deviations from the intended intervention that arose because of the experimental context?	PY
	2.4 If Y/PY to 2.3: Were these deviations likely to have affected the outcome?	PN
	2.5. If Y/PY/NI to 2.4: Were these deviations from intended intervention balanced between groups?	NA
	2.6 Was an appropriate analysis used to estimate the effect of assignment to intervention?	PY
	2.7 If N/PN/NI to 2.6: Was there potential for a substantial impact (on the result) of the failure to analyse participants in the group to which they were randomized?	NA
	**Risk of bias judgement**	**Some concerns**
**Bias due to missing outcome data**	3.1 Were data for this outcome available for all, or nearly all, participants randomized?	PY
	3.2 If N/PN/NI to 3.1: Is there evidence that result was not biased by missing outcome data?	NA
	3.3 If N/PN to 3.2: Could missingness in the outcome depend on its true value?	NA
	3.4 If Y/PY/NI to 3.3: Is it likely that missingness in the outcome depended on its true value?	NA
	**Risk of bias judgement**	**Low**
**Bias in measurement of the outcome**	4.1 Was the method of measuring the outcome inappropriate?	PN
	4.2 Could measurement or ascertainment of the outcome have differed between intervention groups?	PN
	4.3 Were outcome assessors aware of the intervention received by study participants?	PY
	4.4 If Y/PY/NI to 4.3: Could assessment of the outcome have been influenced by knowledge of intervention received?	PY
	4.5 If Y/PY/NI to 4.4: Is it likely that assessment of the outcome was influenced by knowledge of intervention received?	PN
	**Risk of bias judgement**	**Some concerns**
**Bias in selection of the reported result**	5.1 Were the data that produced this result analysed in accordance with a pre-specified analysis plan that was finalized before unblinded outcome data were available for analysis?	PY
	5.2 ... multiple eligible outcome measurements (e.g. scales, definitions, time points) within the outcome domain?	NI
	5.3 ... multiple eligible analyses of the data?	NI
	**Risk of bias judgement**	**Some concerns**
**Overall bias**	**Risk of bias judgement**	**Some concerns**

Study	Durmus 2010	
**Domain**	**Signalling question**	**Response**
**Bias arising from the randomization process**	1.1 Was the allocation sequence random?	PY
	1.2 Was the allocation sequence concealed until participants were enrolled and assigned to interventions?	PY
	1.3 Did baseline differences between intervention groups suggest a problem with the randomization process?	PN
	**Risk of bias judgement**	**Low**
**Bias due to deviations from intended interventions**	2.1.Were participants aware of their assigned intervention during the trial?	PY
	2.2.Were carers and people delivering the interventions aware of participants' assigned intervention during the trial?	PY
	2.3. If Y/PY/NI to 2.1 or 2.2: Were there deviations from the intended intervention that arose because of the experimental context?	PY
	2.4 If Y/PY to 2.3: Were these deviations likely to have affected the outcome?	PY
	2.5. If Y/PY/NI to 2.4: Were these deviations from intended intervention balanced between groups?	PY
	2.6 Was an appropriate analysis used to estimate the effect of assignment to intervention?	PY
	2.7 If N/PN/NI to 2.6: Was there potential for a substantial impact (on the result) of the failure to analyse participants in the group to which they were randomized?	NA
	**Risk of bias judgement**	**Some concerns**
**Bias due to missing outcome data**	3.1 Were data for this outcome available for all, or nearly all, participants randomized?	PN
	3.2 If N/PN/NI to 3.1: Is there evidence that result was not biased by missing outcome data?	PN
	3.3 If N/PN to 3.2: Could missingness in the outcome depend on its true value?	PN
	3.4 If Y/PY/NI to 3.3: Is it likely that missingness in the outcome depended on its true value?	NA
	**Risk of bias judgement**	**Low**
**Bias in measurement of the outcome**	4.1 Was the method of measuring the outcome inappropriate?	PN
	4.2 Could measurement or ascertainment of the outcome have differed between intervention groups?	PN
	4.3 Were outcome assessors aware of the intervention received by study participants?	PY
	4.4 If Y/PY/NI to 4.3: Could assessment of the outcome have been influenced by knowledge of intervention received?	PY
	4.5 If Y/PY/NI to 4.4: Is it likely that assessment of the outcome was influenced by knowledge of intervention received?	PY
	**Risk of bias judgement**	**High**
**Bias in selection of the reported result**	5.1 Were the data that produced this result analysed in accordance with a pre-specified analysis plan that was finalized before unblinded outcome data were available for analysis?	PY
	5.2 ... multiple eligible outcome measurements (e.g. scales, definitions, time points) within the outcome domain?	PY
	5.3 ... multiple eligible analyses of the data?	PY
	**Risk of bias judgement**	**High**
**Overall bias**	**Risk of bias judgement**	**High**

Study	Williams 2009	
**Domain**	**Signalling question**	**Response**
**Bias arising from the randomization process**	1.1 Was the allocation sequence random?	Y
	1.2 Was the allocation sequence concealed until participants were enrolled and assigned to interventions?	PY
	1.3 Did baseline differences between intervention groups suggest a problem with the randomization process?	PN
	**Risk of bias judgement**	**Low**
**Bias due to deviations from intended interventions**	2.1.Were participants aware of their assigned intervention during the trial?	PY
	2.2.Were carers and people delivering the interventions aware of participants' assigned intervention during the trial?	PY
	2.3. If Y/PY/NI to 2.1 or 2.2: Were there deviations from the intended intervention that arose because of the experimental context?	PY
	2.4 If Y/PY to 2.3: Were these deviations likely to have affected the outcome?	PY
	2.5. If Y/PY/NI to 2.4: Were these deviations from intended intervention balanced between groups?	PY
	2.6 Was an appropriate analysis used to estimate the effect of assignment to intervention?	PY
	2.7 If N/PN/NI to 2.6: Was there potential for a substantial impact (on the result) of the failure to analyse participants in the group to which they were randomized?	NA
	**Risk of bias judgement**	**Some concerns**
**Bias due to missing outcome data**	3.1 Were data for this outcome available for all, or nearly all, participants randomized?	PN
	3.2 If N/PN/NI to 3.1: Is there evidence that result was not biased by missing outcome data?	PN
	3.3 If N/PN to 3.2: Could missingness in the outcome depend on its true value?	PY
	3.4 If Y/PY/NI to 3.3: Is it likely that missingness in the outcome depended on its true value?	PN
	**Risk of bias judgement**	**Some concerns**
**Bias in measurement of the outcome**	4.1 Was the method of measuring the outcome inappropriate?	PN
	4.2 Could measurement or ascertainment of the outcome have differed between intervention groups?	PN
	4.3 Were outcome assessors aware of the intervention received by study participants?	PY
	4.4 If Y/PY/NI to 4.3: Could assessment of the outcome have been influenced by knowledge of intervention received?	PY
	4.5 If Y/PY/NI to 4.4: Is it likely that assessment of the outcome was influenced by knowledge of intervention received?	PN
	**Risk of bias judgement**	**Some concerns**
**Bias in selection of the reported result**	5.1 Were the data that produced this result analysed in accordance with a pre-specified analysis plan that was finalized before unblinded outcome data were available for analysis?	PY
	5.2 ... multiple eligible outcome measurements (e.g. scales, definitions, time points) within the outcome domain?	PN
	5.3 ... multiple eligible analyses of the data?	PN
	**Risk of bias judgement**	**Low**
**Overall bias**	**Risk of bias judgement**	**Some concerns**

Study	Sertpoyraz 2009	
**Domain**	**Signalling question**	**Response**
**Bias arising from the randomization process**	1.1 Was the allocation sequence random?	PY
	1.2 Was the allocation sequence concealed until participants were enrolled and assigned to interventions?	PY
	1.3 Did baseline differences between intervention groups suggest a problem with the randomization process?	PN
	**Risk of bias judgement**	**Low**
**Bias due to deviations from intended interventions**	2.1.Were participants aware of their assigned intervention during the trial?	PY
	2.2.Were carers and people delivering the interventions aware of participants' assigned intervention during the trial?	PY
	2.3. If Y/PY/NI to 2.1 or 2.2: Were there deviations from the intended intervention that arose because of the experimental context?	PY
	2.4 If Y/PY to 2.3: Were these deviations likely to have affected the outcome?	PY
	2.5. If Y/PY/NI to 2.4: Were these deviations from intended intervention balanced between groups?	PY
	2.6 Was an appropriate analysis used to estimate the effect of assignment to intervention?	PN
	2.7 If N/PN/NI to 2.6: Was there potential for a substantial impact (on the result) of the failure to analyse participants in the group to which they were randomized?	PY
	**Risk of bias judgement**	**High**
**Bias due to missing outcome data**	3.1 Were data for this outcome available for all, or nearly all, participants randomized?	Y
	3.2 If N/PN/NI to 3.1: Is there evidence that result was not biased by missing outcome data?	NA
	3.3 If N/PN to 3.2: Could missingness in the outcome depend on its true value?	NA
	3.4 If Y/PY/NI to 3.3: Is it likely that missingness in the outcome depended on its true value?	NA
	**Risk of bias judgement**	**Low**
**Bias in measurement of the outcome**	4.1 Was the method of measuring the outcome inappropriate?	PN
	4.2 Could measurement or ascertainment of the outcome have differed between intervention groups?	PN
	4.3 Were outcome assessors aware of the intervention received by study participants?	PY
	4.4 If Y/PY/NI to 4.3: Could assessment of the outcome have been influenced by knowledge of intervention received?	PY
	4.5 If Y/PY/NI to 4.4: Is it likely that assessment of the outcome was influenced by knowledge of intervention received?	PY
	**Risk of bias judgement**	**High**
**Bias in selection of the reported result**	5.1 Were the data that produced this result analysed in accordance with a pre-specified analysis plan that was finalized before unblinded outcome data were available for analysis?	PY
	5.2 ... multiple eligible outcome measurements (e.g. scales, definitions, time points) within the outcome domain?	NI
	5.3 ... multiple eligible analyses of the data?	NI
	**Risk of bias judgement**	**Some concerns**
**Overall bias**	**Risk of bias judgement**	**High**

Study	Ribeiro 2008	
**Domain**	**Signalling question**	**Response**
**Bias arising from the randomization process**	1.1 Was the allocation sequence random?	PY
	1.2 Was the allocation sequence concealed until participants were enrolled and assigned to interventions?	PY
	1.3 Did baseline differences between intervention groups suggest a problem with the randomization process?	PY
	**Risk of bias judgement**	**Some concerns**
**Bias due to deviations from intended interventions**	2.1.Were participants aware of their assigned intervention during the trial?	Y
	2.2.Were carers and people delivering the interventions aware of participants' assigned intervention during the trial?	PN
	2.3. If Y/PY/NI to 2.1 or 2.2: Were there deviations from the intended intervention that arose because of the experimental context?	PY
	2.4 If Y/PY to 2.3: Were these deviations likely to have affected the outcome?	PY
	2.5. If Y/PY/NI to 2.4: Were these deviations from intended intervention balanced between groups?	PY
	2.6 Was an appropriate analysis used to estimate the effect of assignment to intervention?	PY
	2.7 If N/PN/NI to 2.6: Was there potential for a substantial impact (on the result) of the failure to analyse participants in the group to which they were randomized?	NA
	**Risk of bias judgement**	**Some concerns**
**Bias due to missing outcome data**	3.1 Were data for this outcome available for all, or nearly all, participants randomized?	PN
	3.2 If N/PN/NI to 3.1: Is there evidence that result was not biased by missing outcome data?	PY
	3.3 If N/PN to 3.2: Could missingness in the outcome depend on its true value?	NA
	3.4 If Y/PY/NI to 3.3: Is it likely that missingness in the outcome depended on its true value?	NA
	**Risk of bias judgement**	**Low**
**Bias in measurement of the outcome**	4.1 Was the method of measuring the outcome inappropriate?	PN
	4.2 Could measurement or ascertainment of the outcome have differed between intervention groups?	PN
	4.3 Were outcome assessors aware of the intervention received by study participants?	PY
	4.4 If Y/PY/NI to 4.3: Could assessment of the outcome have been influenced by knowledge of intervention received?	PN
	4.5 If Y/PY/NI to 4.4: Is it likely that assessment of the outcome was influenced by knowledge of intervention received?	NA
	**Risk of bias judgement**	**Low**
**Bias in selection of the reported result**	5.1 Were the data that produced this result analysed in accordance with a pre-specified analysis plan that was finalized before unblinded outcome data were available for analysis?	PY
	5.2 ... multiple eligible outcome measurements (e.g. scales, definitions, time points) within the outcome domain?	NI
	5.3 ... multiple eligible analyses of the data?	NI
	**Risk of bias judgement**	**Some concerns**
**Overall bias**	**Risk of bias judgement**	**Some concerns**

Study	Koldaş Doğan 2008	
**Domain**	**Signalling question**	**Response**
**Bias arising from the randomization process**	1.1 Was the allocation sequence random?	Y
	1.2 Was the allocation sequence concealed until participants were enrolled and assigned to interventions?	PY
	1.3 Did baseline differences between intervention groups suggest a problem with the randomization process?	PN
	**Risk of bias judgement**	**Low**
**Bias due to deviations from intended interventions**	2.1.Were participants aware of their assigned intervention during the trial?	PY
	2.2.Were carers and people delivering the interventions aware of participants' assigned intervention during the trial?	PY
	2.3. If Y/PY/NI to 2.1 or 2.2: Were there deviations from the intended intervention that arose because of the experimental context?	PY
	2.4 If Y/PY to 2.3: Were these deviations likely to have affected the outcome?	PY
	2.5. If Y/PY/NI to 2.4: Were these deviations from intended intervention balanced between groups?	PY
	2.6 Was an appropriate analysis used to estimate the effect of assignment to intervention?	PY
	2.7 If N/PN/NI to 2.6: Was there potential for a substantial impact (on the result) of the failure to analyse participants in the group to which they were randomized?	NA
	**Risk of bias judgement**	**Some concerns**
**Bias due to missing outcome data**	3.1 Were data for this outcome available for all, or nearly all, participants randomized?	PY
	3.2 If N/PN/NI to 3.1: Is there evidence that result was not biased by missing outcome data?	NA
	3.3 If N/PN to 3.2: Could missingness in the outcome depend on its true value?	NA
	3.4 If Y/PY/NI to 3.3: Is it likely that missingness in the outcome depended on its true value?	NA
	**Risk of bias judgement**	**Low**
**Bias in measurement of the outcome**	4.1 Was the method of measuring the outcome inappropriate?	PN
	4.2 Could measurement or ascertainment of the outcome have differed between intervention groups?	PN
	4.3 Were outcome assessors aware of the intervention received by study participants?	PY
	4.4 If Y/PY/NI to 4.3: Could assessment of the outcome have been influenced by knowledge of intervention received?	PY
	4.5 If Y/PY/NI to 4.4: Is it likely that assessment of the outcome was influenced by knowledge of intervention received?	PN
	**Risk of bias judgement**	**Some concerns**
**Bias in selection of the reported result**	5.1 Were the data that produced this result analysed in accordance with a pre-specified analysis plan that was finalized before unblinded outcome data were available for analysis?	PY
	5.2 ... multiple eligible outcome measurements (e.g. scales, definitions, time points) within the outcome domain?	NI
	5.3 ... multiple eligible analyses of the data?	NI
	**Risk of bias judgement**	**Some concerns**
**Overall bias**	**Risk of bias judgement**	**Some concerns**

Study	Tavafian 2007	
**Domain**	**Signalling question**	**Response**
**Bias arising from the randomization process**	1.1 Was the allocation sequence random?	Y
	1.2 Was the allocation sequence concealed until participants were enrolled and assigned to interventions?	PY
	1.3 Did baseline differences between intervention groups suggest a problem with the randomization process?	PN
	**Risk of bias judgement**	**Low**
**Bias due to deviations from intended interventions**	2.1.Were participants aware of their assigned intervention during the trial?	Y
	2.2.Were carers and people delivering the interventions aware of participants' assigned intervention during the trial?	PY
	2.3. If Y/PY/NI to 2.1 or 2.2: Were there deviations from the intended intervention that arose because of the experimental context?	PY
	2.4 If Y/PY to 2.3: Were these deviations likely to have affected the outcome?	PY
	2.5. If Y/PY/NI to 2.4: Were these deviations from intended intervention balanced between groups?	PY
	2.6 Was an appropriate analysis used to estimate the effect of assignment to intervention?	PY
	2.7 If N/PN/NI to 2.6: Was there potential for a substantial impact (on the result) of the failure to analyse participants in the group to which they were randomized?	NA
	**Risk of bias judgement**	**Some concerns**
**Bias due to missing outcome data**	3.1 Were data for this outcome available for all, or nearly all, participants randomized?	PY
	3.2 If N/PN/NI to 3.1: Is there evidence that result was not biased by missing outcome data?	NA
	3.3 If N/PN to 3.2: Could missingness in the outcome depend on its true value?	NA
	3.4 If Y/PY/NI to 3.3: Is it likely that missingness in the outcome depended on its true value?	NA
	**Risk of bias judgement**	**Low**
**Bias in measurement of the outcome**	4.1 Was the method of measuring the outcome inappropriate?	PN
	4.2 Could measurement or ascertainment of the outcome have differed between intervention groups?	PN
	4.3 Were outcome assessors aware of the intervention received by study participants?	PY
	4.4 If Y/PY/NI to 4.3: Could assessment of the outcome have been influenced by knowledge of intervention received?	PY
	4.5 If Y/PY/NI to 4.4: Is it likely that assessment of the outcome was influenced by knowledge of intervention received?	PN
	**Risk of bias judgement**	**Some concerns**
**Bias in selection of the reported result**	5.1 Were the data that produced this result analysed in accordance with a pre-specified analysis plan that was finalized before unblinded outcome data were available for analysis?	PY
	5.2 ... multiple eligible outcome measurements (e.g. scales, definitions, time points) within the outcome domain?	PN
	5.3 ... multiple eligible analyses of the data?	PN
	**Risk of bias judgement**	**Low**
**Overall bias**	**Risk of bias judgement**	**Some concerns**

Study	Kaapa 2006	
**Domain**	**Signalling question**	**Response**
**Bias arising from the randomization process**	1.1 Was the allocation sequence random?	Y
	1.2 Was the allocation sequence concealed until participants were enrolled and assigned to interventions?	PY
	1.3 Did baseline differences between intervention groups suggest a problem with the randomization process?	NI
	**Risk of bias judgement**	**Low**
**Bias due to deviations from intended interventions**	2.1.Were participants aware of their assigned intervention during the trial?	PY
	2.2.Were carers and people delivering the interventions aware of participants' assigned intervention during the trial?	PN
	2.3. If Y/PY/NI to 2.1 or 2.2: Were there deviations from the intended intervention that arose because of the experimental context?	PN
	2.4 If Y/PY to 2.3: Were these deviations likely to have affected the outcome?	NA
	2.5. If Y/PY/NI to 2.4: Were these deviations from intended intervention balanced between groups?	NA
	2.6 Was an appropriate analysis used to estimate the effect of assignment to intervention?	PY
	2.7 If N/PN/NI to 2.6: Was there potential for a substantial impact (on the result) of the failure to analyse participants in the group to which they were randomized?	NA
	**Risk of bias judgement**	**Low**
**Bias due to missing outcome data**	3.1 Were data for this outcome available for all, or nearly all, participants randomized?	PN
	3.2 If N/PN/NI to 3.1: Is there evidence that result was not biased by missing outcome data?	PY
	3.3 If N/PN to 3.2: Could missingness in the outcome depend on its true value?	NA
	3.4 If Y/PY/NI to 3.3: Is it likely that missingness in the outcome depended on its true value?	NA
	**Risk of bias judgement**	**Low**
**Bias in measurement of the outcome**	4.1 Was the method of measuring the outcome inappropriate?	PN
	4.2 Could measurement or ascertainment of the outcome have differed between intervention groups?	PN
	4.3 Were outcome assessors aware of the intervention received by study participants?	NI
	4.4 If Y/PY/NI to 4.3: Could assessment of the outcome have been influenced by knowledge of intervention received?	PN
	4.5 If Y/PY/NI to 4.4: Is it likely that assessment of the outcome was influenced by knowledge of intervention received?	NA
	**Risk of bias judgement**	**Low**
**Bias in selection of the reported result**	5.1 Were the data that produced this result analysed in accordance with a pre-specified analysis plan that was finalized before unblinded outcome data were available for analysis?	PY
	5.2 ... multiple eligible outcome measurements (e.g. scales, definitions, time points) within the outcome domain?	PN
	5.3 ... multiple eligible analyses of the data?	PN
	**Risk of bias judgement**	**Low**
**Overall bias**	**Risk of bias judgement**	**Low**

Study	Galantino 2004	
**Domain**	**Signalling question**	**Response**
**Bias arising from the randomization process**	1.1 Was the allocation sequence random?	Y
	1.2 Was the allocation sequence concealed until participants were enrolled and assigned to interventions?	Y
	1.3 Did baseline differences between intervention groups suggest a problem with the randomization process?	PN
	**Risk of bias judgement**	**Low**
**Bias due to deviations from intended interventions**	2.1.Were participants aware of their assigned intervention during the trial?	PY
	2.2.Were carers and people delivering the interventions aware of participants' assigned intervention during the trial?	PY
	2.3. If Y/PY/NI to 2.1 or 2.2: Were there deviations from the intended intervention that arose because of the experimental context?	PY
	2.4 If Y/PY to 2.3: Were these deviations likely to have affected the outcome?	PY
	2.5. If Y/PY/NI to 2.4: Were these deviations from intended intervention balanced between groups?	PY
	2.6 Was an appropriate analysis used to estimate the effect of assignment to intervention?	PY
	2.7 If N/PN/NI to 2.6: Was there potential for a substantial impact (on the result) of the failure to analyse participants in the group to which they were randomized?	NA
	**Risk of bias judgement**	**Some concerns**
**Bias due to missing outcome data**	3.1 Were data for this outcome available for all, or nearly all, participants randomized?	PN
	3.2 If N/PN/NI to 3.1: Is there evidence that result was not biased by missing outcome data?	PY
	3.3 If N/PN to 3.2: Could missingness in the outcome depend on its true value?	NA
	3.4 If Y/PY/NI to 3.3: Is it likely that missingness in the outcome depended on its true value?	NA
	**Risk of bias judgement**	**Low**
**Bias in measurement of the outcome**	4.1 Was the method of measuring the outcome inappropriate?	PN
	4.2 Could measurement or ascertainment of the outcome have differed between intervention groups?	PN
	4.3 Were outcome assessors aware of the intervention received by study participants?	PY
	4.4 If Y/PY/NI to 4.3: Could assessment of the outcome have been influenced by knowledge of intervention received?	PY
	4.5 If Y/PY/NI to 4.4: Is it likely that assessment of the outcome was influenced by knowledge of intervention received?	PN
	**Risk of bias judgement**	**Some concerns**
**Bias in selection of the reported result**	5.1 Were the data that produced this result analysed in accordance with a pre-specified analysis plan that was finalized before unblinded outcome data were available for analysis?	PY
	5.2 ... multiple eligible outcome measurements (e.g. scales, definitions, time points) within the outcome domain?	NI
	5.3 ... multiple eligible analyses of the data?	NI
	**Risk of bias judgement**	**Some concerns**
**Overall bias**	**Risk of bias judgement**	**Some concerns**

Study	Weiner 2003	
**Domain**	**Signalling question**	**Response**
**Bias arising from the randomization process**	1.1 Was the allocation sequence random?	Y
	1.2 Was the allocation sequence concealed until participants were enrolled and assigned to interventions?	PY
	1.3 Did baseline differences between intervention groups suggest a problem with the randomization process?	PN
	**Risk of bias judgement**	**Low**
**Bias due to deviations from intended interventions**	2.1.Were participants aware of their assigned intervention during the trial?	PY
	2.2.Were carers and people delivering the interventions aware of participants' assigned intervention during the trial?	PY
	2.3. If Y/PY/NI to 2.1 or 2.2: Were there deviations from the intended intervention that arose because of the experimental context?	PY
	2.4 If Y/PY to 2.3: Were these deviations likely to have affected the outcome?	PN
	2.5. If Y/PY/NI to 2.4: Were these deviations from intended intervention balanced between groups?	NA
	2.6 Was an appropriate analysis used to estimate the effect of assignment to intervention?	PY
	2.7 If N/PN/NI to 2.6: Was there potential for a substantial impact (on the result) of the failure to analyse participants in the group to which they were randomized?	NA
	**Risk of bias judgement**	**Some concerns**
**Bias due to missing outcome data**	3.1 Were data for this outcome available for all, or nearly all, participants randomized?	PY
	3.2 If N/PN/NI to 3.1: Is there evidence that result was not biased by missing outcome data?	NA
	3.3 If N/PN to 3.2: Could missingness in the outcome depend on its true value?	NA
	3.4 If Y/PY/NI to 3.3: Is it likely that missingness in the outcome depended on its true value?	NA
	**Risk of bias judgement**	**Low**
**Bias in measurement of the outcome**	4.1 Was the method of measuring the outcome inappropriate?	PN
	4.2 Could measurement or ascertainment of the outcome have differed between intervention groups?	PN
	4.3 Were outcome assessors aware of the intervention received by study participants?	PY
	4.4 If Y/PY/NI to 4.3: Could assessment of the outcome have been influenced by knowledge of intervention received?	PN
	4.5 If Y/PY/NI to 4.4: Is it likely that assessment of the outcome was influenced by knowledge of intervention received?	NA
	**Risk of bias judgement**	**Low**
**Bias in selection of the reported result**	5.1 Were the data that produced this result analysed in accordance with a pre-specified analysis plan that was finalized before unblinded outcome data were available for analysis?	PY
	5.2 ... multiple eligible outcome measurements (e.g. scales, definitions, time points) within the outcome domain?	PN
	5.3 ... multiple eligible analyses of the data?	PN
	**Risk of bias judgement**	**Low**
**Overall bias**	**Risk of bias judgement**	**Some concerns**

Study	Niemisto 2003	
**Domain**	**Signalling question**	**Response**
**Bias arising from the randomization process**	1.1 Was the allocation sequence random?	Y
	1.2 Was the allocation sequence concealed until participants were enrolled and assigned to interventions?	PY
	1.3 Did baseline differences between intervention groups suggest a problem with the randomization process?	PN
	**Risk of bias judgement**	**Low**
**Bias due to deviations from intended interventions**	2.1.Were participants aware of their assigned intervention during the trial?	PY
	2.2.Were carers and people delivering the interventions aware of participants' assigned intervention during the trial?	PY
	2.3. If Y/PY/NI to 2.1 or 2.2: Were there deviations from the intended intervention that arose because of the experimental context?	PY
	2.4 If Y/PY to 2.3: Were these deviations likely to have affected the outcome?	PY
	2.5. If Y/PY/NI to 2.4: Were these deviations from intended intervention balanced between groups?	PY
	2.6 Was an appropriate analysis used to estimate the effect of assignment to intervention?	PY
	2.7 If N/PN/NI to 2.6: Was there potential for a substantial impact (on the result) of the failure to analyse participants in the group to which they were randomized?	NA
	**Risk of bias judgement**	**Some concerns**
**Bias due to missing outcome data**	3.1 Were data for this outcome available for all, or nearly all, participants randomized?	PN
	3.2 If N/PN/NI to 3.1: Is there evidence that result was not biased by missing outcome data?	PN
	3.3 If N/PN to 3.2: Could missingness in the outcome depend on its true value?	PY
	3.4 If Y/PY/NI to 3.3: Is it likely that missingness in the outcome depended on its true value?	PN
	**Risk of bias judgement**	**Some concerns**
**Bias in measurement of the outcome**	4.1 Was the method of measuring the outcome inappropriate?	PN
	4.2 Could measurement or ascertainment of the outcome have differed between intervention groups?	PY
	4.3 Were outcome assessors aware of the intervention received by study participants?	NA
	4.4 If Y/PY/NI to 4.3: Could assessment of the outcome have been influenced by knowledge of intervention received?	NA
	4.5 If Y/PY/NI to 4.4: Is it likely that assessment of the outcome was influenced by knowledge of intervention received?	NA
	**Risk of bias judgement**	**High**
**Bias in selection of the reported result**	5.1 Were the data that produced this result analysed in accordance with a pre-specified analysis plan that was finalized before unblinded outcome data were available for analysis?	PY
	5.2 ... multiple eligible outcome measurements (e.g. scales, definitions, time points) within the outcome domain?	NI
	5.3 ... multiple eligible analyses of the data?	NI
	**Risk of bias judgement**	**Some concerns**
**Overall bias**	**Risk of bias judgement**	**High**

Study	Hernandez-Reif 2001	
**Domain**	**Signalling question**	**Response**
**Bias arising from the randomization process**	1.1 Was the allocation sequence random?	Y
	1.2 Was the allocation sequence concealed until participants were enrolled and assigned to interventions?	NI
	1.3 Did baseline differences between intervention groups suggest a problem with the randomization process?	PN
	**Risk of bias judgement**	**Some concerns**
**Bias due to deviations from intended interventions**	2.1.Were participants aware of their assigned intervention during the trial?	PY
	2.2.Were carers and people delivering the interventions aware of participants' assigned intervention during the trial?	PY
	2.3. If Y/PY/NI to 2.1 or 2.2: Were there deviations from the intended intervention that arose because of the experimental context?	PY
	2.4 If Y/PY to 2.3: Were these deviations likely to have affected the outcome?	PN
	2.5. If Y/PY/NI to 2.4: Were these deviations from intended intervention balanced between groups?	NA
	2.6 Was an appropriate analysis used to estimate the effect of assignment to intervention?	PY
	2.7 If N/PN/NI to 2.6: Was there potential for a substantial impact (on the result) of the failure to analyse participants in the group to which they were randomized?	NA
	**Risk of bias judgement**	**Some concerns**
**Bias due to missing outcome data**	3.1 Were data for this outcome available for all, or nearly all, participants randomized?	PY
	3.2 If N/PN/NI to 3.1: Is there evidence that result was not biased by missing outcome data?	NA
	3.3 If N/PN to 3.2: Could missingness in the outcome depend on its true value?	NA
	3.4 If Y/PY/NI to 3.3: Is it likely that missingness in the outcome depended on its true value?	NA
	**Risk of bias judgement**	**Low**
**Bias in measurement of the outcome**	4.1 Was the method of measuring the outcome inappropriate?	PN
	4.2 Could measurement or ascertainment of the outcome have differed between intervention groups?	PN
	4.3 Were outcome assessors aware of the intervention received by study participants?	PY
	4.4 If Y/PY/NI to 4.3: Could assessment of the outcome have been influenced by knowledge of intervention received?	PN
	4.5 If Y/PY/NI to 4.4: Is it likely that assessment of the outcome was influenced by knowledge of intervention received?	NA
	**Risk of bias judgement**	**Low**
**Bias in selection of the reported result**	5.1 Were the data that produced this result analysed in accordance with a pre-specified analysis plan that was finalized before unblinded outcome data were available for analysis?	PY
	5.2 ... multiple eligible outcome measurements (e.g. scales, definitions, time points) within the outcome domain?	NI
	5.3 ... multiple eligible analyses of the data?	NI
	**Risk of bias judgement**	**Some concerns**
**Overall bias**	**Risk of bias judgement**	**Some concerns**

Study	Newton-John 1995	
**Domain**	**Signalling question**	**Response**
**Bias arising from the randomization process**	1.1 Was the allocation sequence random?	PY
	1.2 Was the allocation sequence concealed until participants were enrolled and assigned to interventions?	PY
	1.3 Did baseline differences between intervention groups suggest a problem with the randomization process?	NI
	**Risk of bias judgement**	**Low**
**Bias due to deviations from intended interventions**	2.1.Were participants aware of their assigned intervention during the trial?	PY
	2.2.Were carers and people delivering the interventions aware of participants' assigned intervention during the trial?	PY
	2.3. If Y/PY/NI to 2.1 or 2.2: Were there deviations from the intended intervention that arose because of the experimental context?	PY
	2.4 If Y/PY to 2.3: Were these deviations likely to have affected the outcome?	PY
	2.5. If Y/PY/NI to 2.4: Were these deviations from intended intervention balanced between groups?	PY
	2.6 Was an appropriate analysis used to estimate the effect of assignment to intervention?	PN
	2.7 If N/PN/NI to 2.6: Was there potential for a substantial impact (on the result) of the failure to analyse participants in the group to which they were randomized?	PN
	**Risk of bias judgement**	**Some concerns**
**Bias due to missing outcome data**	3.1 Were data for this outcome available for all, or nearly all, participants randomized?	PN
	3.2 If N/PN/NI to 3.1: Is there evidence that result was not biased by missing outcome data?	PN
	3.3 If N/PN to 3.2: Could missingness in the outcome depend on its true value?	PY
	3.4 If Y/PY/NI to 3.3: Is it likely that missingness in the outcome depended on its true value?	PN
	**Risk of bias judgement**	**Some concerns**
**Bias in measurement of the outcome**	4.1 Was the method of measuring the outcome inappropriate?	PN
	4.2 Could measurement or ascertainment of the outcome have differed between intervention groups?	PN
	4.3 Were outcome assessors aware of the intervention received by study participants?	PY
	4.4 If Y/PY/NI to 4.3: Could assessment of the outcome have been influenced by knowledge of intervention received?	PN
	4.5 If Y/PY/NI to 4.4: Is it likely that assessment of the outcome was influenced by knowledge of intervention received?	NA
	**Risk of bias judgement**	**Low**
**Bias in selection of the reported result**	5.1 Were the data that produced this result analysed in accordance with a pre-specified analysis plan that was finalized before unblinded outcome data were available for analysis?	PY
	5.2 ... multiple eligible outcome measurements (e.g. scales, definitions, time points) within the outcome domain?	NI
	5.3 ... multiple eligible analyses of the data?	NI
	**Risk of bias judgement**	**Some concerns**
**Overall bias**	**Risk of bias judgement**	**Some concerns**

Study	Turner 1993	
**Domain**	**Signalling question**	**Response**
**Bias arising from the randomization process**	1.1 Was the allocation sequence random?	PY
	1.2 Was the allocation sequence concealed until participants were enrolled and assigned to interventions?	PY
	1.3 Did baseline differences between intervention groups suggest a problem with the randomization process?	PY
	**Risk of bias judgement**	**Some concerns**
**Bias due to deviations from intended interventions**	2.1.Were participants aware of their assigned intervention during the trial?	PY
	2.2.Were carers and people delivering the interventions aware of participants' assigned intervention during the trial?	PY
	2.3. If Y/PY/NI to 2.1 or 2.2: Were there deviations from the intended intervention that arose because of the experimental context?	PN
	2.4 If Y/PY to 2.3: Were these deviations likely to have affected the outcome?	NA
	2.5. If Y/PY/NI to 2.4: Were these deviations from intended intervention balanced between groups?	NA
	2.6 Was an appropriate analysis used to estimate the effect of assignment to intervention?	PY
	2.7 If N/PN/NI to 2.6: Was there potential for a substantial impact (on the result) of the failure to analyse participants in the group to which they were randomized?	NA
	**Risk of bias judgement**	**Low**
**Bias due to missing outcome data**	3.1 Were data for this outcome available for all, or nearly all, participants randomized?	PN
	3.2 If N/PN/NI to 3.1: Is there evidence that result was not biased by missing outcome data?	PN
	3.3 If N/PN to 3.2: Could missingness in the outcome depend on its true value?	Y
	3.4 If Y/PY/NI to 3.3: Is it likely that missingness in the outcome depended on its true value?	PY
	**Risk of bias judgement**	**High**
**Bias in measurement of the outcome**	4.1 Was the method of measuring the outcome inappropriate?	PN
	4.2 Could measurement or ascertainment of the outcome have differed between intervention groups?	PN
	4.3 Were outcome assessors aware of the intervention received by study participants?	PY
	4.4 If Y/PY/NI to 4.3: Could assessment of the outcome have been influenced by knowledge of intervention received?	PY
	4.5 If Y/PY/NI to 4.4: Is it likely that assessment of the outcome was influenced by knowledge of intervention received?	PN
	**Risk of bias judgement**	**Some concerns**
**Bias in selection of the reported result**	5.1 Were the data that produced this result analysed in accordance with a pre-specified analysis plan that was finalized before unblinded outcome data were available for analysis?	PY
	5.2 ... multiple eligible outcome measurements (e.g. scales, definitions, time points) within the outcome domain?	NI
	5.3 ... multiple eligible analyses of the data?	NI
	**Risk of bias judgement**	**Some concerns**
**Overall bias**	**Risk of bias judgement**	**High**

Appendix 7. Certainty of evidence ratings on direct evidence of different categories of interventions for depression, anxiety, and mental health

Comparison	SMD 95%(CI)	Risk of bias	Inconsistency	Indirectness	Publication bias	Need to assess indirect	Preliminary rating
**Depression**							
EX vs CO	-0.21 (-0.40, -0.01)	Downgrade two levels	No downgrade	No downgrade	No downgrade	Yes	Low
MBT vs CO	-1.28 (-2.11, -0.44)	No downgrade	Downgrade one level	No downgrade	NA	Yes	Moderate
EDU vs CO	-0.24 (-0.61, 0.13)	Downgrade two levels	Downgrade one level	No downgrade	NA	Yes	Very low
TM vs CO	0.08 (-0.48, 0.63)	Downgrade one level	Downgrade one level	No downgrade	NA	Yes	Low
BA vs CO	-0.42 (-0.73, -0.12)	Downgrade one level	Downgrade one level	No downgrade	No downgrade	Yes	Low
MUI vs CO	-0.13 (-0.29, 0.02)	Downgrade two levels	No downgrade	No downgrade	NA	Yes	Low
PT vs CO	-0.25 (-0.39, -0.10)	Downgrade one level	No downgrade	No downgrade	No downgrade	Yes	Moderate
BA vs EDU	-0.39 (-0.71, -0.07)	Downgrade one level	NA	No downgrade	NA	Yes	Moderate
BA vs EX	0.36 (-0.39, 1.11)	Downgrade two levels	NA	No downgrade	NA	Yes	Low
EX vs MUI	0.34 (-1.25, 1.93)	Downgrade one level	Downgrade one level	No downgrade	NA	Yes	Low
EX vs PT	0.13 (-0.47, 0.74)	Downgrade one level	Downgrade one level	No downgrade	NA	Yes	Low
MUI vs PT	-0.98 (-1.88, -0.09)	Downgrade one level	NA	No downgrade	NA	Yes	Moderate
BA vs MUI	-0.05 (-0.75, 0.65)	Downgrade one level	NA	No downgrade	NA	Yes	Moderate
BA vs MBT	NA	NA	NA	NA	NA	Yes	NA
BA vs PT	NA	NA	NA	NA	NA	Yes	NA
BA vs TM	NA	NA	NA	NA	NA	Yes	NA
EDU vs EX	NA	NA	NA	NA	NA	Yes	NA
EDU vs MBT	NA	NA	NA	NA	NA	Yes	NA
EDU vs MUI	NA	NA	NA	NA	NA	Yes	NA
EDU vs PT	NA	NA	NA	NA	NA	Yes	NA
EDU vs TM	NA	NA	NA	NA	NA	Yes	NA
EX vs MBT	NA	NA	NA	NA	NA	Yes	NA
EX vs TM	NA	NA	NA	NA	NA	Yes	NA
MBT vs MUI	NA	NA	NA	NA	NA	Yes	NA
MBT vs PT	NA	NA	NA	NA	NA	Yes	NA
MBT vs TM	NA	NA	NA	NA	NA	Yes	NA
MUI vs TM	NA	NA	NA	NA	NA	Yes	NA
PT vs TM	NA	NA	NA	NA	NA	Yes	NA
**Anxiety**							
EX vs CO	-0.27 (-0.54, 0.00)	Downgrade one level	No downgrade	No downgrade	NA	Yes	Moderate
MBT vs CO	-1.34 (-1.66, -1.02)	No downgrade	No downgrade	No downgrade	NA	Yes	High
EDU vs CO	-0.55 (-1.04, -0.06)	Downgrade one level	Downgrade one level	No downgrade	NA	Yes	Low
TM vs CO	-0.03 (-0.50, 0.44)	No downgrade	No downgrade	No downgrade	NA	Yes	High
BA vs CO	-0.47 (-0.88, -0.06)	Downgrade one level	Downgrade one level	No downgrade	No downgrade	Yes	Low
MUI vs CO	-0.41 (-0.63, -0.19)	Downgrade one level	No downgrade	No downgrade	NA	Yes	Moderate
PT vs CO	0.33 (-0.13, 0.79)	Downgrade two levels	No downgrade	No downgrade	NA	Yes	Low
EX vs MUI	0.59 (-0.14, 1.33)	Downgrade one level	Downgrade one level	No downgrade	NA	Yes	Low
EX vs PT	0.35 (-0.83, 1.53)	Downgrade one level	Downgrade one level	No downgrade	NA	Yes	Low
BA vs EDU	-0.29 (-0.60, 0.03)	Downgrade one level	NA	No downgrade	NA	Yes	Moderate
MUI vs PT	-1.53 (-2.51, -0.55)	Downgrade one level	NA	No downgrade	NA	Yes	Moderate
BA vs EX	0.04 (-0.36, 0.43)	Downgrade two levels	NA	No downgrade	NA	Yes	Low
BA vs MBT	NA	NA	NA	NA	NA	Yes	NA
BA vs MUI	NA	NA	NA	NA	NA	Yes	NA
BA vs PT	NA	NA	NA	NA	NA	Yes	NA
BA vs TM	NA	NA	NA	NA	NA	Yes	NA
EDU vs EX	NA	NA	NA	NA	NA	Yes	NA
EDU vs MBT	NA	NA	NA	NA	NA	Yes	NA
EDU vs MUI	NA	NA	NA	NA	NA	Yes	NA
EDU vs PT	NA	NA	NA	NA	NA	Yes	NA
EDU vs TM	NA	NA	NA	NA	NA	Yes	NA
EX vs MBT	NA	NA	NA	NA	NA	Yes	NA
EX vs TM	NA	NA	NA	NA	NA	Yes	NA
MBT vs MUI	NA	NA	NA	NA	NA	Yes	NA
MBT vs PT	NA	NA	NA	NA	NA	Yes	NA
MBT vs TM	NA	NA	NA	NA	NA	Yes	NA
MUI vs TM	NA	NA	NA	NA	NA	Yes	NA
PT vs TM	NA	NA	NA	NA	NA	Yes	NA
**Mental health**							
EX vs CO	0.56 (0.01, 1.11)	Downgrade one level	Downgrade one level	No downgrade	NA	Yes	Low
EDU vs CO	0.30 (-0.11, 0.71)	Downgrade two levels	Downgrade one level	No downgrade	NA	Yes	Very low

TM vs CO	0.41 (-0.69, 1.50)	No downgrade	Downgrade one level	No downgrade	NA	Yes	Moderate
BA vs CO	0.43 (-0.29, 1.14)	No downgrade	Downgrade one level	No downgrade	NA	Yes	Moderate
MUI vs CO	0.60 (0.01, 1.19)	Downgrade one level	Downgrade one level	No downgrade	NA	Yes	Low
PT vs CO	0.47 (0.11, 0.83)	Downgrade two levels	Downgrade one level	No downgrade	No downgrade	Yes	Very low
EX vs MUI	-0.47 (-1.31, 0.37)	Downgrade one level	Downgrade one level	No downgrade	NA	Yes	Low
EX vs PT	0.44 (-0.19, 1.08)	Downgrade two levels	NA	No downgrade	NA	Yes	Low
BA vs EDU	NA	NA	NA	NA	NA	Yes	NA
BA vs EX	NA	NA	NA	NA	NA	Yes	NA
BA vs MUI	NA	NA	NA	NA	NA	Yes	NA
BA vs PT	NA	NA	NA	NA	NA	Yes	NA
BA vs TM	NA	NA	NA	NA	NA	Yes	NA
EDU vs EX	NA	NA	NA	NA	NA	Yes	NA
EDU vs MUI	NA	NA	NA	NA	NA	Yes	NA
EDU vs PT	NA	NA	NA	NA	NA	Yes	NA
EDU vs TM	NA	NA	NA	NA	NA	Yes	NA
EX vs TM	NA	NA	NA	NA	NA	Yes	NA
MUI vs PT	NA	NA	NA	NA	NA	Yes	NA
MUI vs TM	NA	NA	NA	NA	NA	Yes	NA
PT vs TM	NA	NA	NA	NA	NA	Yes	NA

Appendix 8. Certainty of evidence ratings on direct evidence of different specific treatments for depression, anxiety, and mental health

Comparison	SMD 95%(CI)	Risk of bias	Inconsistency	Indirectness	Publication bias	Need to assess indirect	Preliminary rating
**Depression**							
AT vs AC	-0.12 (-0.49,0.25)	Downgrade two levels	No downgrade	No downgrade	NA	Yes	Low
CBT vs AC	0.09 (-0.34,0.51)	Downgrade two levels	NA	No downgrade	NA	Yes	Low
EDU vs AC	-1.02 (-1.61,-0.43)	Downgrade one level	NA	No downgrade	NA	Yes	Moderate
ES vs AC	-0.42 (-1.05,0.21)	Downgrade two levels	NA	No downgrade	NA	Yes	Low
Mass vs AC	-0.55 (-1.18,0.09)	Downgrade one level	NA	No downgrade	NA	Yes	Moderate
MBT vs AC	-1.46 (-3.03,0.11)	Downgrade one level	Downgrade one level	No downgrade	NA	Yes	Low
ME vs AC	-0.07 (-0.45,0.32)	Downgrade two levels	NA	No downgrade	NA	Yes	Low
MUI vs AC	0.05 (-0.23,0.32)	Downgrade one level	No downgrade	No downgrade	NA	Yes	Moderate
PI vs AC	-0.43 (-0.82,-0.05)	Downgrade one level	No downgrade	No downgrade	NA	Yes	Moderate
AC vs TM	-0.12 (-1.05,0.80)	Downgrade two levels	Downgrade one level	No downgrade	NA	Yes	Very low
Acup vs UC	-0.41 (-0.84,0.01)	No downgrade	No downgrade	No downgrade	NA	Yes	High
AT vs ME	-0.25 (-1.04,0.54)	Downgrade one level	NA	No downgrade	NA	Yes	Moderate
AT vs SE	0.47 (-0.37,1.31)	Downgrade one level	NA	No downgrade	NA	Yes	Moderate
AT vs UC	-0.58 (-1.44,0.27)	Downgrade one level	NA	No downgrade	NA	Yes	Moderate
CBT vs EDU	-0.27 (-0.64,0.09)	Downgrade one level	NA	No downgrade	NA	Yes	Moderate
CBT vs ME	0.17 (-0.23,0.57)	Downgrade two levels	NA	No downgrade	NA	Yes	Low
CBT vs MUI	-0.05 (-0.75,0.65)	Downgrade two levels	NA	No downgrade	NA	Yes	Low
CBT vs PI	-0.03 (-0.47,0.41)	Downgrade one level	Downgrade one level	No downgrade	NA	Yes	Low
CBT vs RT	0.36 (-0.39,1.11)	Downgrade two levels	NA	No downgrade	NA	Yes	Low
CBT vs UC	-0.82 (-1.59,-0.05)	Downgrade one level	Downgrade one level	No downgrade	NA	Yes	Low
PI vs EDU	-0.50 (-0.86.-0.13)	Downgrade one level	NA	No downgrade	NA	Yes	Moderate
EDU vs UC	-0.08 (-0.24,0.08)	Downgrade one level	No downgrade	No downgrade	NA	Yes	Moderate
ES vs KT	-0.01 (-0.52,0.50)	Downgrade one level	NA	No downgrade	NA	Yes	Moderate
ES vs UC	0.10 (-0.27,0.46)	Downgrade one level	No downgrade	No downgrade	NA	Yes	Moderate
MUI vs KT	-0.98 (-1.88,-0.09)	Downgrade one level	NA	No downgrade	NA	Yes	Moderate
SE vs KT	0.00 (-0.83,0.82)	Downgrade one level	NA	No downgrade	NA	Yes	Moderate
Mass vs RT	-0.76 (-1.59,0.08)	Downgrade one level	NA	No downgrade	NA	Yes	Moderate
Mass vs UC	-0.54 (-1.07,0.00)	Downgrade one level	NA	No downgrade	NA	Yes	Moderate
MBT vs UC	-1.03 (-1.68,-0.37)	Downgrade one level	Downgrade one level	No downgrade	NA	Yes	Low
MUI vs ME	-0.26 (-0.64,0.11)	Downgrade one level	NA	No downgrade	NA	Yes	Moderate
SE vs ME	-0.65 (-1.52,0.22)	Downgrade one level	NA	No downgrade	NA	Yes	Moderate
ME vs UC	-0.34 (0.72,0.04)	Downgrade one level	NA	No downgrade	NA	Yes	Moderate
Medi vs UC	-0.36 (-1.27,0.56)	No downgrade	Downgrade one level	No downgrade	NA	Yes	Moderate
RT vs MUI	-0.45 (-1.16,0.26)	Downgrade two levels	NA	No downgrade	NA	Yes	Low
MUI vs SE	-1.17 (-2.03,-0.31)	Downgrade one level	NA	No downgrade	NA	Yes	Moderate
MUI vs UC	-0.24 (-0.52,0.04)	Downgrade two levels	Downgrade one level	No downgrade	NA	Yes	Very low
PI vs UC	-0.31 (-0.86,0.23)	Downgrade one level	NA	No downgrade	NA	Yes	Moderate
TM vs UC	0.02 (-0.65,0.70)	Downgrade one level	NA	No downgrade	NA	Yes	Moderate
AC vs Acup	NA	NA	NA	NA	NA	Yes	NA
AC vs KT	NA	NA	NA	NA	NA	Yes	NA
AC vs Medi	NA	NA	NA	NA	NA	Yes	NA
AC vs RT	NA	NA	NA	NA	NA	Yes	NA
AC vs SE	NA	NA	NA	NA	NA	Yes	NA
AC vs UC	NA	NA	NA	NA	NA	Yes	NA
Acup vs AT	NA	NA	NA	NA	NA	Yes	NA
Acup vs CBT	NA	NA	NA	NA	NA	Yes	NA
Acup vs EDU	NA	NA	NA	NA	NA	Yes	NA
Acup vs ES	NA	NA	NA	NA	NA	Yes	NA
Acup vs KT	NA	NA	NA	NA	NA	Yes	NA
Acup vs Mass	NA	NA	NA	NA	NA	Yes	NA
Acup vs MBT	NA	NA	NA	NA	NA	Yes	NA
Acup vs ME	NA	NA	NA	NA	NA	Yes	NA
Acup vs Medi	NA	NA	NA	NA	NA	Yes	NA
Acup vs MUI	NA	NA	NA	NA	NA	Yes	NA
Acup vs PI	NA	NA	NA	NA	NA	Yes	NA
Acup vs RT	NA	NA	NA	NA	NA	Yes	NA
Acup vs SE	NA	NA	NA	NA	NA	Yes	NA
Acup vs TM	NA	NA	NA	NA	NA	Yes	NA
AT vs CBT	NA	NA	NA	NA	NA	Yes	NA
AT vs EDU	NA	NA	NA	NA	NA	Yes	NA
AT vs ES	NA	NA	NA	NA	NA	Yes	NA

AT vs KT	NA	NA	NA	NA	NA	Yes	NA
AT vs Mass	NA	NA	NA	NA	NA	Yes	NA
AT vs MBT	NA	NA	NA	NA	NA	Yes	NA
AT vs Medi	NA	NA	NA	NA	NA	Yes	NA
AT vs MUI	NA	NA	NA	NA	NA	Yes	NA
AT vs PI	NA	NA	NA	NA	NA	Yes	NA
AT vs RT	NA	NA	NA	NA	NA	Yes	NA
AT vs TM	NA	NA	NA	NA	NA	Yes	NA
CBT vs ES	NA	NA	NA	NA	NA	Yes	NA
CBT vs KT	NA	NA	NA	NA	NA	Yes	NA
CBT vs Mass	NA	NA	NA	NA	NA	Yes	NA
CBT vs MBT	NA	NA	NA	NA	NA	Yes	NA
CBT vs Medi	NA	NA	NA	NA	NA	Yes	NA
CBT vs SE	NA	NA	NA	NA	NA	Yes	NA
CBT vs TM	NA	NA	NA	NA	NA	Yes	NA
EDU vs ES	NA	NA	NA	NA	NA	Yes	NA
EDU vs KT	NA	NA	NA	NA	NA	Yes	NA
EDU vs Mass	NA	NA	NA	NA	NA	Yes	NA
EDU vs MBT	NA	NA	NA	NA	NA	Yes	NA
EDU vs ME	NA	NA	NA	NA	NA	Yes	NA
EDU vs Medi	NA	NA	NA	NA	NA	Yes	NA
EDU vs MUI	NA	NA	NA	NA	NA	Yes	NA
EDU vs RT	NA	NA	NA	NA	NA	Yes	NA
EDU vs SE	NA	NA	NA	NA	NA	Yes	NA
EDU vs TM	NA	NA	NA	NA	NA	Yes	NA
ES vs Mass	NA	NA	NA	NA	NA	Yes	NA
ES vs MBT	NA	NA	NA	NA	NA	Yes	NA
ES vs ME	NA	NA	NA	NA	NA	Yes	NA
ES vs Medi	NA	NA	NA	NA	NA	Yes	NA
ES vs MUI	NA	NA	NA	NA	NA	Yes	NA
ES vs PI	NA	NA	NA	NA	NA	Yes	NA
ES vs RT	NA	NA	NA	NA	NA	Yes	NA
ES vs SE	NA	NA	NA	NA	NA	Yes	NA
ES vs TM	NA	NA	NA	NA	NA	Yes	NA
KT vs Mass	NA	NA	NA	NA	NA	Yes	NA
KT vs MBT	NA	NA	NA	NA	NA	Yes	NA
KT vs ME	NA	NA	NA	NA	NA	Yes	NA
KT vs Medi	NA	NA	NA	NA	NA	Yes	NA
KT vs PI	NA	NA	NA	NA	NA	Yes	NA
KT vs RT	NA	NA	NA	NA	NA	Yes	NA
KT vs TM	NA	NA	NA	NA	NA	Yes	NA
KT vs UC	NA	NA	NA	NA	NA	Yes	NA
Mass vs MBT	NA	NA	NA	NA	NA	Yes	NA
Mass vs ME	NA	NA	NA	NA	NA	Yes	NA
Mass vs Medi	NA	NA	NA	NA	NA	Yes	NA
Mass vs MUI	NA	NA	NA	NA	NA	Yes	NA
Mass vs PI	NA	NA	NA	NA	NA	Yes	NA
Mass vs SE	NA	NA	NA	NA	NA	Yes	NA
Mass vs TM	NA	NA	NA	NA	NA	Yes	NA
MBT vs ME	NA	NA	NA	NA	NA	Yes	NA
MBT vs Medi	NA	NA	NA	NA	NA	Yes	NA
MBT vs MUI	NA	NA	NA	NA	NA	Yes	NA
MBT vs PI	NA	NA	NA	NA	NA	Yes	NA
MBT vs RT	NA	NA	NA	NA	NA	Yes	NA
MBT vs SE	NA	NA	NA	NA	NA	Yes	NA
MBT vs TM	NA	NA	NA	NA	NA	Yes	NA
ME vs Medi	NA	NA	NA	NA	NA	Yes	NA
ME vs PI	NA	NA	NA	NA	NA	Yes	NA
ME vs RT	NA	NA	NA	NA	NA	Yes	NA
ME vs TM	NA	NA	NA	NA	NA	Yes	NA
Medi vs MUI	NA	NA	NA	NA	NA	Yes	NA
Medi vs PI	NA	NA	NA	NA	NA	Yes	NA
Medi vs RT	NA	NA	NA	NA	NA	Yes	NA
Medi vs SE	NA	NA	NA	NA	NA	Yes	NA
Medi vs TM	NA	NA	NA	NA	NA	Yes	NA
MUI vs PI	NA	NA	NA	NA	NA	Yes	NA
MUI vs TM	NA	NA	NA	NA	NA	Yes	NA

PI vs RT	NA	NA	NA	NA	NA	Yes	NA
PI vs SE	NA	NA	NA	NA	NA	Yes	NA
PI vs TM	NA	NA	NA	NA	NA	Yes	NA
RT vs SE	NA	NA	NA	NA	NA	Yes	NA
RT vs TM	NA	NA	NA	NA	NA	Yes	NA
RT vs UC	NA	NA	NA	NA	NA	Yes	NA
SE vs TM	NA	NA	NA	NA	NA	Yes	NA
SE vs UC	NA	NA	NA	NA	NA	Yes	NA
**Anxiety**							
CBT vs AC	-0.08 (-0.50,0.35)	Downgrade two levels	NA	No downgrade	NA	Yes	Low
EDU vs AC	-0.74 (-1.31,-0.16)	Downgrade one level	NA	No downgrade	NA	Yes	Moderate
MBT vs AC	-1.34 (-1.69,-0.99)	Downgrade one level	No downgrade	No downgrade	NA	Yes	Moderate
ME vs AC	-0.12 (-0.50,0.27)	Downgrade two levels	NA	No downgrade	NA	Yes	Low
MUI vs AC	-0.20 (-0.58,0.17)	Downgrade one level	NA	No downgrade	NA	Yes	Moderate
PI vs AC	-0.27 (-0.73,0.18)	Downgrade one level	NA	No downgrade	NA	Yes	Moderate
AC vs TM	-0.13 (-0.78,0.52)	Downgrade one level	NA	No downgrade	NA	Yes	Moderate
CBT vs EDU	-0.20 (-0.56,0.17)	Downgrade one level	NA	No downgrade	NA	Yes	Moderate
CBT vs ME	0.04 (-0.36,0.43)	Downgrade two levels	NA	No downgrade	NA	Yes	Low
CBT vs PI	0.19 (-0.14,0.51)	Downgrade one level	No downgrade	No downgrade	NA	Yes	Moderate
CBT vs UC	-1.38 (-3.32,0.56)	Downgrade one level	Downgrade one level	No downgrade	NA	Yes	Low
PI vs EDU	-0.38 (-0.74,-0.02)	Downgrade one level	NA	No downgrade	NA	Yes	Moderate
EDU vs UC	-0.46 (-1.24,0.32)	Downgrade one level	Downgrade one level	No downgrade	NA	Yes	Low
ES vs KT	0.05 (-0.47,0.56)	Downgrade one level	NA	No downgrade	NA	Yes	Moderate
UC vs ES	-0.19 (-1.05,0.67)	Downgrade one level	NA	No downgrade	NA	Yes	Moderate
MUI vs KT	-1.53 (-2.51,-0.55)	Downgrade one level	NA	No downgrade	NA	Yes	Moderate
MBT vs UC	-1.33 (-2.13,-0.53)	Downgrade one level	NA	No downgrade	NA	Yes	Moderate
MUI vs ME	-0.31 (-0.68,0.06)	Downgrade one level	NA	No downgrade	NA	Yes	Moderate
ME vs UC	-1.33 (-2.13,-0.53)	Downgrade one level	NA	No downgrade	NA	Yes	Moderate
Medi vs UC	-0.41 (-1.43,0.61)	No downgrade	Downgrade one level	No downgrade	NA	Yes	Moderate
MUI vs UC	-0.51 (-0.78,-0.24)	Downgrade one level	No downgrade	No downgrade	NA	Yes	Moderate
PI vs UC	0.00 (-0.54,0.54)	Downgrade one level	NA	No downgrade	NA	Yes	Moderate
TM vs UC	-0.20 (-0.88,0.47)	Downgrade one level	NA	No downgrade	NA	Yes	Moderate
AC vs ES	NA	NA	NA	NA	NA	Yes	NA
AC vs KT	NA	NA	NA	NA	NA	Yes	NA
AC vs Medi	NA	NA	NA	NA	NA	Yes	NA
AC vs UC	NA	NA	NA	NA	NA	Yes	NA
CBT vs ES	NA	NA	NA	NA	NA	Yes	NA
CBT vs KT	NA	NA	NA	NA	NA	Yes	NA
CBT vs MBT	NA	NA	NA	NA	NA	Yes	NA
CBT vs Medi	NA	NA	NA	NA	NA	Yes	NA
CBT vs MUI	NA	NA	NA	NA	NA	Yes	NA
CBT vs TM	NA	NA	NA	NA	NA	Yes	NA
EDU vs ES	NA	NA	NA	NA	NA	Yes	NA
EDU vs KT	NA	NA	NA	NA	NA	Yes	NA
EDU vs MBT	NA	NA	NA	NA	NA	Yes	NA
EDU vs ME	NA	NA	NA	NA	NA	Yes	NA
EDU vs Medi	NA	NA	NA	NA	NA	Yes	NA
EDU vs MUI	NA	NA	NA	NA	NA	Yes	NA
EDU vs TM	NA	NA	NA	NA	NA	Yes	NA
ES vs MBT	NA	NA	NA	NA	NA	Yes	NA
ES vs ME	NA	NA	NA	NA	NA	Yes	NA
ES vs Medi	NA	NA	NA	NA	NA	Yes	NA
ES vs MUI	NA	NA	NA	NA	NA	Yes	NA
ES vs PI	NA	NA	NA	NA	NA	Yes	NA
ES vs TM	NA	NA	NA	NA	NA	Yes	NA
KT vs MBT	NA	NA	NA	NA	NA	Yes	NA
KT vs ME	NA	NA	NA	NA	NA	Yes	NA
KT vs Medi	NA	NA	NA	NA	NA	Yes	NA
KT vs PI	NA	NA	NA	NA	NA	Yes	NA
KT vs TM	NA	NA	NA	NA	NA	Yes	NA
KT vs UC	NA	NA	NA	NA	NA	Yes	NA
MBT vs ME	NA	NA	NA	NA	NA	Yes	NA
MBT vs Medi	NA	NA	NA	NA	NA	Yes	NA
MBT vs MUI	NA	NA	NA	NA	NA	Yes	NA
MBT vs PI	NA	NA	NA	NA	NA	Yes	NA
MBT vs TM	NA	NA	NA	NA	NA	Yes	NA
ME vs Medi	NA	NA	NA	NA	NA	Yes	NA

ME vs PI	NA	NA	NA	NA	NA	Yes	NA
ME vs TM	NA	NA	NA	NA	NA	Yes	NA
Medi vs MUI	NA	NA	NA	NA	NA	Yes	NA
Medi vs PI	NA	NA	NA	NA	NA	Yes	NA
Medi vs TM	NA	NA	NA	NA	NA	Yes	NA
MUI vs PI	NA	NA	NA	NA	NA	Yes	NA
MUI vs TM	NA	NA	NA	NA	NA	Yes	NA
PI vs TM	NA	NA	NA	NA	NA	Yes	NA
**Mental health**							
AT vs AC	1.03 (0.03,2.02)	Downgrade one level	Downgrade one level	No downgrade	NA	Yes	Low
EDU vs AC	0.57 (-0.01,1.14)	Downgrade two levels	NA	No downgrade	NA	Yes	Low
TM vs AC	0.59 (-1.05,2.23)	Downgrade one level	Downgrade one level	No downgrade	NA	Yes	Low
MUI vs AT	0.02 (-0.54,0.58)	Downgrade two levels	NA	No downgrade	NA	Yes	Low
AT vs UC	-0.04 (-0.61,0.53)	Downgrade two levels	NA	No downgrade	NA	Yes	Low
EDU vs UC	0.24 (-0.26,0.73)	Downgrade one level	Downgrade one level	No downgrade	NA	Yes	Low
Medi vs UC	0.43 (-0.29,1.14)	No downgrade	Downgrade one level	No downgrade	NA	Yes	Moderate
MUI vs UC	0.60 (0.01,1.19)	Downgrade one level	Downgrade one level	No downgrade	NA	Yes	Low
TM vs UC	0.02 (-0.66,0.69)	Downgrade one level	NA	No downgrade	NA	Yes	Moderate
AC vs Medi	NA	NA	NA	NA	NA	Yes	NA
AC vs MUI	NA	NA	NA	NA	NA	Yes	NA
AC vs UC	NA	NA	NA	NA	NA	Yes	NA
AT vs EDU	NA	NA	NA	NA	NA	Yes	NA
AT vs Medi	NA	NA	NA	NA	NA	Yes	NA
AT vs TM	NA	NA	NA	NA	NA	Yes	NA
EDU vs Medi	NA	NA	NA	NA	NA	Yes	NA
EDU vs MUI	NA	NA	NA	NA	NA	Yes	NA
EDU vs TM	NA	NA	NA	NA	NA	Yes	NA
Medi vs MUI	NA	NA	NA	NA	NA	Yes	NA
Medi vs TM	NA	NA	NA	NA	NA	Yes	NA
MUI vs TM	NA	NA	NA	NA	NA	Yes	NA

Appendix 9. Certainty of evidence ratings on indirect and network evidence of different categories of interventions for depression

Comparison	Within-study bias	Reporting bias	Indirectness	Imprecision	Heterogeneity	Incoherence	Confidence rating
**Mixed evidence**							
BA vs CO	Some concerns	Low risk	No concerns	No concerns	Major concerns	No concerns	Very low
BA vs EDU	No concerns	Some concerns	No concerns	Some concerns	No concerns	No concerns	Low
BA vs EX	Major concerns	Some concerns	No concerns	Some concerns	Some concerns	No concerns	Very low
BA vs MUI	Major concerns	Some concerns	No concerns	Major concerns	No concerns	No concerns	Very low
CO vs EDU	Major concerns	Low risk	No concerns	Some concerns	Some concerns	No concerns	Very low
CO vs EX	Major concerns	Low risk	No concerns	Some concerns	Some concerns	No concerns	Very low
CO vs MBT	Some concerns	Low risk	No concerns	No concerns	No concerns	No concerns	Moderate
CO vs MUI	Major concerns	Low risk	No concerns	Some concerns	Some concerns	No concerns	Very low
CO vs PT	Some concerns	Low risk	No concerns	No concerns	Major concerns	No concerns	Very low
CO vs TM	Some concerns	Low risk	No concerns	Major concerns	No concerns	No concerns	Very low
EX vs MUI	Major concerns	Low risk	No concerns	Major concerns	No concerns	No concerns	Very low
EX vs PT	Some concerns	Low risk	No concerns	Major concerns	No concerns	No concerns	Very low
MUI vs PT	Some concerns	Some concerns	No concerns	Major concerns	No concerns	Some concerns	Very low
**Indirect evidence**							
BA vs MBT	Some concerns	Low risk	No concerns	No concerns	Some concerns	No concerns	Low
BA vs PT	Some concerns	Low risk	No concerns	Some concerns	Some concerns	No concerns	Very low
BA vs TM	Some concerns	Some concerns	No concerns	Some concerns	Some concerns	No concerns	Very low
EDU vs EX	Major concerns	Low risk	No concerns	Major concerns	No concerns	No concerns	Very low
EDU vs MBT	Some concerns	Low risk	No concerns	No concerns	No concerns	No concerns	Moderate
EDU vs MUI	Major concerns	Low risk	No concerns	Major concerns	No concerns	No concerns	Very low
EDU vs PT	Major concerns	Low risk	No concerns	Major concerns	No concerns	No concerns	Very low
EDU vs TM	Some concerns	Some concerns	No concerns	Major concerns	No concerns	No concerns	Very low
EX vs MBT	Some concerns	Low risk	No concerns	No concerns	No concerns	No concerns	Moderate
EX vs TM	Some concerns	Some concerns	No concerns	Major concerns	No concerns	No concerns	Very low
MBT vs MUI	Some concerns	Low risk	No concerns	No concerns	No concerns	No concerns	Moderate
MBT vs PT	Some concerns	Low risk	No concerns	No concerns	No concerns	No concerns	Moderate
MBT vs TM	Some concerns	Some concerns	No concerns	No concerns	No concerns	No concerns	Low
MUI vs TM	Some concerns	Some concerns	No concerns	Major concerns	No concerns	No concerns	Very low
PT vs TM	Some concerns	Some concerns	No concerns	Some concerns	No concerns	No concerns	Very low

Appendix 10. Certainty of evidence ratings on indirect and network evidence of different categories of interventions for anxiety

Comparison	Within-study bias	Reporting bias	Indirectness	Imprecision	Heterogeneity	Incoherence	Confidence rating
**Mixed evidence**							
BA vs CO	Some concerns	Low risk	No concerns	No concerns	Major concerns	No concerns	Very low
BA vs EDU	No concerns	Some concerns	No concerns	Major concerns	No concerns	No concerns	Very low
BA vs EX	Major concerns	Some concerns	No concerns	Some concerns	Some concerns	No concerns	Very low
CO vs EDU	Some concerns	Low risk	No concerns	No concerns	Major concerns	No concerns	Very low
CO vs EX	Major concerns	Low risk	No concerns	Major concerns	No concerns	No concerns	Very low
CO vs MBT	Some concerns	Low risk	No concerns	No concerns	No concerns	No concerns	Moderate
CO vs MUI	Some concerns	Low risk	No concerns	No concerns	Major concerns	No concerns	Very low
CO vs PT	Some concerns	Low risk	No concerns	Major concerns	No concerns	No concerns	Very low
CO vs TM	Some concerns	Low risk	No concerns	Major concerns	No concerns	No concerns	Very low
EX vs MUI	No concerns	Low risk	No concerns	Some concerns	Some concerns	No concerns	Low
EX vs PT	Some concerns	Low risk	No concerns	Major concerns	No concerns	Some concerns	Very low
MUI vs PT	Some concerns	Some concerns	No concerns	Some concerns	Some concerns	No concerns	Very low
**Indirect evidence**							
BA vs MBT	Some concerns	Low risk	No concerns	No concerns	Some concerns	No concerns	Low
BA vs MUI	Some concerns	Low risk	No concerns	Major concerns	No concerns	No concerns	Very low
BA vs PT	Some concerns	Some concerns	No concerns	Some concerns	Some concerns	No concerns	Very low
BA vs TM	Some concerns	Some concerns	No concerns	Major concerns	No concerns	No concerns	Very low
EDU vs EX	Major concerns	Low risk	No concerns	Major concerns	No concerns	No concerns	Very low
EDU vs MBT	Some concerns	Low risk	No concerns	No concerns	Some concerns	No concerns	Low
EDU vs MUI	Some concerns	Low risk	No concerns	Major concerns	No concerns	No concerns	Very low
EDU vs PT	Some concerns	Some concerns	No concerns	Some concerns	Some concerns	No concerns	Very low
EDU vs TM	Some concerns	Some concerns	No concerns	Major concerns	No concerns	No concerns	Very low
EX vs MBT	Some concerns	Low risk	No concerns	No concerns	No concerns	No concerns	Moderate
EX vs TM	Some concerns	Some concerns	No concerns	Major concerns	No concerns	No concerns	Very low
MBT vs MUI	Some concerns	Low risk	No concerns	No concerns	Some concerns	No concerns	Low
MBT vs PT	Some concerns	Some concerns	No concerns	No concerns	No concerns	No concerns	Low
MBT vs TM	Some concerns	Some concerns	No concerns	No concerns	No concerns	No concerns	Low
MUI vs TM	Some concerns	Some concerns	No concerns	Major concerns	No concerns	No concerns	Very low
PT vs TM	Some concerns	Some concerns	No concerns	Major concerns	No concerns	No concerns	Very low

Appendix 11. Certainty of evidence ratings on indirect and network evidence of different categories of interventions for mental health

Comparison	Within-study bias	Reporting bias	Indirectness	Imprecision	Heterogeneity	Incoherence	Confidence rating
**Mixed evidence**							
BA vs CO	Some concerns	Low risk	No concerns	Major concerns	No concerns	No concerns	Very low
CO vs EDU	Major concerns	Low risk	No concerns	Major concerns	No concerns	No concerns	Very low
CO vs EX	No concerns	Low risk	No concerns	No concerns	Major concerns	No concerns	Low
CO vs MUI	No concerns	Low risk	No concerns	No concerns	Major concerns	Some concerns	Very low
CO vs PT	Major concerns	Low risk	No concerns	No concerns	Major concerns	No concerns	Very low
CO vs TM	Some concerns	Low risk	No concerns	Major concerns	No concerns	No concerns	Very low
EX vs MUI	No concerns	Low risk	No concerns	Major concerns	No concerns	No concerns	Low
EX vs PT	Major concerns	Some concerns	No concerns	Major concerns	No concerns	No concerns	Very low
**Indirect evidence**							
BA vs EDU	Some concerns	Some concerns	No concerns	Major concerns	No concerns	No concerns	Very low
BA vs EX	Some concerns	Some concerns	No concerns	Major concerns	No concerns	No concerns	Very low
BA vs MUI	Some concerns	Some concerns	No concerns	Major concerns	No concerns	No concerns	Very low
BA vs PT	Some concerns	Some concerns	No concerns	Major concerns	No concerns	No concerns	Very low
BA vs TM	Some concerns	Some concerns	No concerns	Major concerns	No concerns	No concerns	Very low
EDU vs EX	Major concerns	Low risk	No concerns	Major concerns	No concerns	No concerns	Very low
EDU vs MUI	Major concerns	Low risk	No concerns	Major concerns	No concerns	No concerns	Very low
EDU vs PT	Major concerns	Low risk	No concerns	Major concerns	No concerns	No concerns	Very low
EDU vs TM	Some concerns	Some concerns	No concerns	Major concerns	No concerns	No concerns	Very low
EX vs TM	Some concerns	Some concerns	No concerns	Major concerns	No concerns	No concerns	Very low
MUI vs PT	Major concerns	Low risk	No concerns	Major concerns	No concerns	No concerns	Very low
MUI vs TM	Some concerns	Some concerns	No concerns	Major concerns	No concerns	No concerns	Very low
PT vs TM	Some concerns	Some concerns	No concerns	Major concerns	No concerns	No concerns	Very low

Appendix 12. Certainty of evidence ratings on indirect and network evidence of different specific treatments for depression

Comparison	Within-study bias	Reporting bias	Indirectness	Imprecision	Heterogeneity	Incoherence	Confidence rating
**Mixed evidence**							
AC vs AT	Major concerns	Low risk	No concerns	Major concerns	No concerns	No concerns	Very low
AC vs CBT	Major concerns	Some concerns	No concerns	No concerns	Major concerns	No concerns	Very low
AC vs EDU	Some concerns	Some concerns	No concerns	Major concerns	No concerns	Some concerns	Very low
AC vs ES	Major concerns	Some concerns	No concerns	Major concerns	No concerns	No concerns	Very low
AC vs MBT	Some concerns	Low risk	No concerns	Low risk	No concerns	No concerns	Low
AC vs ME	Major concerns	Some concerns	No concerns	Major concerns	No concerns	No concerns	Very low
AC vs MUI	Some concerns	Low risk	No concerns	Some concerns	Some concerns	No concerns	Very low
AC vs Mass	Some concerns	Some concerns	No concerns	No concerns	Major concerns	No concerns	Very low
AC vs PI	No concerns	Low risk	No concerns	Some concerns	Some concerns	No concerns	Low
AC vs TM	Some concerns	Low risk	No concerns	Major concerns	No concerns	No concerns	Very low
AT vs ME	Some concerns	Some concerns	No concerns	Major concerns	No concerns	No concerns	Very low
AT vs SE	Some concerns	Some concerns	Some concerns	Major concerns	No concerns	No concerns	Very low
AT vs UC	Some concerns	Some concerns	No concerns	Major concerns	No concerns	No concerns	Very low
Acup vs UC	No concerns	Low risk	No concerns	Major concerns	No concerns	No concerns	Low
CBT vs EDU	No concerns	Some concerns	No concerns	Major concerns	No concerns	No concerns	Very low
CBT vs ME	Major concerns	Some concerns	No concerns	Major concerns	No concerns	No concerns	Very low
CBT vs MUI	Major concerns	Some concerns	No concerns	Major concerns	No concerns	No concerns	Very low
CBT vs PI	Some concerns	Low risk	No concerns	Major concerns	No concerns	No concerns	Very low
CBT vs RT	Major concerns	Some concerns	No concerns	Major concerns	No concerns	No concerns	Very low
CBT vs UC	Some concerns	Low risk	No concerns	No concerns	Major concerns	No concerns	Very low
EDU vs PI	No concerns	Some concerns	No concerns	Major concerns	No concerns	No concerns	Very low
EDU vs UC	Major concerns	Low risk	No concerns	Some concerns	Some concerns	No concerns	Very low
ES vs KT	Some concerns	Some concerns	No concerns	Major concerns	No concerns	No concerns	Very low
ES vs UC	Some concerns	Low risk	No concerns	Major concerns	No concerns	No concerns	Very low
KT vs MUI	Some concerns	Some concerns	No concerns	Major concerns	No concerns	No concerns	Very low
KT vs SE	Some concerns	Some concerns	No concerns	Major concerns	No concerns	No concerns	Very low
MBT vs UC	Some concerns	Low risk	No concerns	Low risk	No concerns	No concerns	Low
ME vs MUI	No concerns	Some concerns	No concerns	Major concerns	No concerns	No concerns	Very low
ME vs SE	Some concerns	Some concerns	No concerns	Major concerns	No concerns	No concerns	Very low
ME vs UC	No concerns	Some concerns	No concerns	Major concerns	No concerns	No concerns	Very low
MUI vs RT	Major concerns	Some concerns	No concerns	Major concerns	No concerns	No concerns	Very low
MUI vs SE	Some concerns	Some concerns	No concerns	Major concerns	No concerns	Some concerns	Very low
MUI vs UC	Major concerns	Low risk	No concerns	Some concerns	Some concerns	No concerns	Very low
Mass vs RT	Some concerns	Some concerns	No concerns	Major concerns	No concerns	No concerns	Very low
Mass vs UC	Some concerns	Some concerns	No concerns	No concerns	Major concerns	No concerns	Very low
Medi vs UC	Some concerns	Low risk	No concerns	Major concerns	No concerns	No concerns	Very low
PI vs UC	Some concerns	Some concerns	No concerns	Some concerns	Some concerns	No concerns	Very low
TM vs UC	Some concerns	Some concerns	No concerns	Major concerns	No concerns	No concerns	Very low
**Indirect evidence**							
AC vs Acup	Some concerns	Low risk	No concerns	Major concerns	No concerns	No concerns	Very low
AC vs KT	Some concerns	Low risk	No concerns	Major concerns	No concerns	No concerns	Very low
AC vs Medi	Some concerns	Low risk	No concerns	Major concerns	No concerns	No concerns	Very low
AC vs RT	Major concerns	Low risk	No concerns	Major concerns	No concerns	No concerns	Very low
AC vs SE	Some concerns	Low risk	No concerns	Major concerns	No concerns	No concerns	Very low
AC vs UC	Some concerns	Low risk	No concerns	Major concerns	No concerns	No concerns	Very low
Acup vs AT	Some concerns	Low risk	No concerns	Major concerns	No concerns	No concerns	Very low
AT vs CBT	Some concerns	Low risk	No concerns	Major concerns	No concerns	No concerns	Very low
AT vs EDU	Some concerns	Low risk	No concerns	Major concerns	No concerns	No concerns	Very low
AT vs ES	Some concerns	Low risk	No concerns	Major concerns	No concerns	No concerns	Very low
AT vs KT	Some concerns	Some concerns	No concerns	Major concerns	No concerns	No concerns	Very low
AT vs MBT	Some concerns	Low risk	No concerns	No concerns	Some concerns	No concerns	Low
AT vs MUI	Some concerns	Low risk	No concerns	Major concerns	No concerns	No concerns	Very low
AT vs Mass	Some concerns	Low risk	No concerns	Major concerns	No concerns	No concerns	Very low
AT vs Medi	Some concerns	Low risk	No concerns	Major concerns	No concerns	No concerns	Very low
AT vs PI	Some concerns	Low risk	No concerns	Major concerns	No concerns	No concerns	Very low
AT vs RT	Some concerns	Some concerns	No concerns	Major concerns	No concerns	No concerns	Very low
AT vs TM	Some concerns	Low risk	No concerns	Major concerns	No concerns	No concerns	Very low
Acup vs CBT	Some concerns	Low risk	No concerns	Major concerns	No concerns	No concerns	Very low
Acup vs EDU	Some concerns	Low risk	No concerns	Major concerns	No concerns	No concerns	Very low
Acup vs ES	Some concerns	Low risk	No concerns	Major concerns	No concerns	No concerns	Very low
Acup vs KT	Some concerns	Low risk	No concerns	Major concerns	No concerns	No concerns	Very low

Acup vs MBT	Some concerns	Low risk	No concerns	Some concerns	Some concerns	No concerns	Very low
Acup vs ME	No concerns	Low risk	No concerns	Major concerns	No concerns	No concerns	Low
Acup vs MUI	No concerns	Low risk	No concerns	Major concerns	No concerns	No concerns	Low
Acup vs Mass	Some concerns	Low risk	No concerns	Major concerns	No concerns	No concerns	Very low
Acup vs Medi	Some concerns	Low risk	No concerns	Major concerns	No concerns	No concerns	Very low
Acup vs PI	Some concerns	Low risk	No concerns	Major concerns	No concerns	No concerns	Very low
Acup vs RT	Some concerns	Low risk	No concerns	Major concerns	No concerns	No concerns	Very low
Acup vs SE	Some concerns	Some concerns	No concerns	Major concerns	No concerns	No concerns	Very low
Acup vs TM	Some concerns	Low risk	No concerns	Major concerns	No concerns	No concerns	Very low
CBT vs ES	Some concerns	Low risk	No concerns	Major concerns	No concerns	No concerns	Very low
CBT vs KT	Some concerns	Some concerns	No concerns	Major concerns	No concerns	No concerns	Very low
CBT vs MBT	Some concerns	Low risk	No concerns	No concerns	Major concerns	No concerns	Very low
CBT vs Mass	Some concerns	Low risk	No concerns	Major concerns	No concerns	No concerns	Very low
CBT vs Medi	Some concerns	Low risk	No concerns	Major concerns	No concerns	No concerns	Very low
CBT vs SE	Some concerns	Some concerns	No concerns	Major concerns	No concerns	No concerns	Very low
CBT vs TM	Some concerns	Low risk	No concerns	Some concerns	Some concerns	No concerns	Very low
EDU vs ES	Major concerns	Low risk	No concerns	Major concerns	No concerns	No concerns	Very low
EDU vs KT	Some concerns	Some concerns	No concerns	Major concerns	No concerns	No concerns	Very low
EDU vs MBT	Some concerns	Low risk	No concerns	No concerns	Some concerns	No concerns	Low
EDU vs ME	Major concerns	Low risk	No concerns	Major concerns	No concerns	No concerns	Very low
EDU vs MUI	Major concerns	Low risk	No concerns	Major concerns	No concerns	No concerns	Very low
EDU vs Mass	Some concerns	Low risk	No concerns	Major concerns	No concerns	No concerns	Very low
EDU vs Medi	Some concerns	Low risk	No concerns	Major concerns	No concerns	No concerns	Very low
EDU vs RT	Major concerns	Some concerns	No concerns	Major concerns	No concerns	No concerns	Very low
EDU vs SE	Some concerns	Some concerns	No concerns	Major concerns	No concerns	No concerns	Very low
EDU vs TM	Some concerns	Low risk	No concerns	Major concerns	No concerns	No concerns	Very low
ES vs MBT	Some concerns	Low risk	No concerns	No concerns	No concerns	No concerns	Moderate
ES vs ME	Major concerns	Low risk	No concerns	Major concerns	No concerns	No concerns	Very low
ES vs MUI	Some concerns	Low risk	No concerns	Major concerns	No concerns	No concerns	Very low
ES vs Mass	Some concerns	Low risk	No concerns	Some concerns	Some concerns	No concerns	Very low
ES vs Medi	Some concerns	Low risk	No concerns	Major concerns	No concerns	No concerns	Very low
ES vs PI	Some concerns	Low risk	No concerns	Major concerns	No concerns	No concerns	Very low
ES vs RT	Some concerns	Some concerns	No concerns	Major concerns	No concerns	No concerns	Very low
ES vs SE	Some concerns	Some concerns	No concerns	Major concerns	No concerns	No concerns	Very low
ES vs TM	Some concerns	Low risk	No concerns	Major concerns	No concerns	No concerns	Very low
KT vs MBT	Some concerns	Some concerns	No concerns	No concerns	No concerns	No concerns	Low
KT vs ME	Some concerns	Some concerns	No concerns	Major concerns	No concerns	No concerns	Very low
KT vs Mass	Some concerns	Some concerns	No concerns	Some concerns	Some concerns	No concerns	Very low
KT vs Medi	Some concerns	Some concerns	No concerns	Major concerns	No concerns	No concerns	Very low
KT vs PI	Some concerns	Some concerns	No concerns	Major concerns	No concerns	No concerns	Very low
KT vs RT	Some concerns	Some concerns	No concerns	Major concerns	No concerns	No concerns	Very low
KT vs TM	Some concerns	Some concerns	No concerns	Major concerns	No concerns	No concerns	Very low
KT vs UC	Some concerns	Some concerns	No concerns	Major concerns	No concerns	No concerns	Very low
MBT vs ME	Some concerns	Low risk	No concerns	No concerns	No concerns	No concerns	Moderate
MBT vs MUI	Some concerns	Low risk	No concerns	No concerns	Some concerns	No concerns	Low
Mass vs MBT	Some concerns	Low risk	No concerns	Major concerns	No concerns	No concerns	Very low
MBT vs Medi	Some concerns	Low risk	No concerns	No concerns	Major concerns	No concerns	Very low
MBT vs PI	Some concerns	Low risk	No concerns	No concerns	Major concerns	No concerns	Very low
MBT vs RT	Some concerns	Some concerns	No concerns	Some concerns	Some concerns	No concerns	Very low
MBT vs SE	Some concerns	Some concerns	No concerns	No concerns	Some concerns	No concerns	Very low
MBT vs TM	Some concerns	Low risk	No concerns	No concerns	No concerns	No concerns	Moderate
Mass vs ME	Some concerns	Low risk	No concerns	Major concerns	No concerns	No concerns	Very low
ME vs Medi	Some concerns	Low risk	No concerns	Major concerns	No concerns	No concerns	Very low
ME vs PI	Major concerns	Low risk	No concerns	Major concerns	No concerns	No concerns	Very low
ME vs RT	Major concerns	Some concerns	No concerns	Major concerns	No concerns	No concerns	Very low
ME vs TM	Some concerns	Low risk	No concerns	Major concerns	No concerns	No concerns	Very low
Mass vs MUI	Some concerns	Low risk	No concerns	Major concerns	No concerns	No concerns	Very low
Medi vs MUI	Some concerns	Low risk	No concerns	Major concerns	No concerns	No concerns	Very low
MUI vs PI	Major concerns	Low risk	No concerns	Major concerns	No concerns	No concerns	Very low
MUI vs TM	Some concerns	Low risk	No concerns	Major concerns	No concerns	No concerns	Very low
Mass vs Medi	Some concerns	Low risk	No concerns	Major concerns	No concerns	No concerns	Very low
Mass vs PI	Some concerns	Low risk	No concerns	Major concerns	No concerns	No concerns	Very low
Mass vs SE	Some concerns	Some concerns	No concerns	Major concerns	No concerns	No concerns	Very low
Mass vs TM	Some concerns	Low risk	No concerns	Some concerns	Some concerns	No concerns	Very low
Medi vs PI	Some concerns	Low risk	No concerns	Major concerns	No concerns	No concerns	Very low
Medi vs RT	Some concerns	Some concerns	No concerns	Major concerns	No concerns	No concerns	Very low
Medi vs SE	Some concerns	Some concerns	No concerns	Major concerns	No concerns	No concerns	Very low

Medi vs TM	Some concerns	Low risk	No concerns	Major concerns	No concerns	No concerns	Very low
PI vs RT	Major concerns	Some concerns	No concerns	Major concerns	No concerns	No concerns	Very low
PI vs SE	Some concerns	Some concerns	No concerns	Major concerns	No concerns	No concerns	Very low
PI vs TM	Some concerns	Low risk	No concerns	Major concerns	No concerns	No concerns	Very low
RT vs SE	Some concerns	Some concerns	No concerns	Major concerns	No concerns	No concerns	Very low
RT vs TM	Some concerns	Some concerns	No concerns	Major concerns	No concerns	No concerns	Very low
RT vs UC	Major concerns	Some concerns	No concerns	Major concerns	No concerns	No concerns	Very low
SE vs TM	Some concerns	Some concerns	No concerns	Major concerns	No concerns	No concerns	Very low
SE vs UC	Some concerns	Some concerns	No concerns	Major concerns	No concerns	No concerns	Very low

Appendix 13. Certainty of evidence ratings on indirect and network evidence of different specific treatments for anxiety

Comparison	Within-study bias	Reporting bias	Indirectness	Imprecision	Heterogeneity	Incoherence	Confidence rating
			**Mixed evidence**				
AC vs CBT	Major concerns	Some concerns	No concerns	Some concerns	Some concerns	No concerns	Very low
AC vs EDU	Some concerns	Some concerns	No concerns	Major concerns	No concerns	No concerns	Very low
AC vs MBT	Some concerns	Low risk	No concerns	No concerns	No concerns	No concerns	Moderate
AC vs ME	Major concerns	Some concerns	No concerns	Major concerns	No concerns	No concerns	Very low
AC vs MUI	Some concerns	Some concerns	No concerns	Some concerns	Some concerns	No concerns	Very low
AC vs PI	No concerns	Some concerns	No concerns	Some concerns	Some concerns	No concerns	Very low
AC vs TM	Some concerns	Some concerns	No concerns	Major concerns	No concerns	No concerns	Very low
CBT vs EDU	No concerns	Some concerns	No concerns	Major concerns	No concerns	No concerns	Very low
CBT vs ME	Major concerns	Some concerns	No concerns	Major concerns	No concerns	No concerns	Very low
CBT vs PI	No concerns	Low risk	No concerns	Major concerns	No concerns	No concerns	Low
CBT vs UC	Some concerns	Low risk	No concerns	No concerns	Major concerns	Major concerns	Very low
EDU vs PI	No concerns	Some concerns	No concerns	Major concerns	No concerns	No concerns	Very low
EDU vs UC	Some concerns	Low risk	No concerns	No concerns	Major concerns	No concerns	Very low
ES vs KT	Some concerns	Some concerns	No concerns	Major concerns	No concerns	No concerns	Very low
ES vs UC	Some concerns	Some concerns	No concerns	Major concerns	No concerns	No concerns	Very low
KT vs MUI	Some concerns	Some concerns	No concerns	No concerns	Some concerns	No concerns	Very low
MBT vs UC	Some concerns	Some concerns	No concerns	No concerns	No concerns	No concerns	Low
ME vs MUI	No concerns	Some concerns	No concerns	Major concerns	No concerns	No concerns	Very low
ME vs UC	No concerns	Some concerns	No concerns	Some concerns	Some concerns	No concerns	Very low
MUI vs UC	Some concerns	Low risk	No concerns	No concerns	Major concerns	No concerns	Very low
Medi vs UC	Some concerns	Low risk	No concerns	Some concerns	Some concerns	No concerns	Very low
PI vs UC	Some concerns	Some concerns	No concerns	No concerns	Major concerns	No concerns	Very low
TM vs UC	Some concerns	Some concerns	No concerns	Major concerns	No concerns	No concerns	Very low
			**Indirect evidence**				
AC vs ES	Some concerns	Some concerns	No concerns	Major concerns	No concerns	No concerns	Very low
AC vs KT	Some concerns	Some concerns	No concerns	Some concerns	Some concerns	No concerns	Very low
AC vs Medi	Some concerns	Low risk	No concerns	Major concerns	No concerns	No concerns	Very low
AC vs UC	Some concerns	Low risk	No concerns	Major concerns	No concerns	No concerns	Very low
CBT vs ES	Some concerns	Some concerns	No concerns	No concerns	Some concerns	No concerns	Very low
CBT vs KT	Some concerns	Some concerns	No concerns	No concerns	Some concerns	No concerns	Very low
CBT vs MBT	Some concerns	Low risk	No concerns	No concerns	Some concerns	No concerns	Low
CBT vs MUI	Some concerns	Low risk	No concerns	Major concerns	No concerns	No concerns	Very low
CBT vs Medi	Some concerns	Low risk	No concerns	Major concerns	No concerns	No concerns	Very low
CBT vs TM	Some concerns	Some concerns	No concerns	Major concerns	No concerns	No concerns	Very low
EDU vs ES	Some concerns	Some concerns	No concerns	No concerns	Major concerns	No concerns	Very low
EDU vs KT	Some concerns	Some concerns	No concerns	No concerns	Some concerns	No concerns	Very low
EDU vs MBT	Some concerns	Low risk	No concerns	No concerns	Some concerns	No concerns	Low
EDU vs ME	No concerns	Low risk	No concerns	Major concerns	No concerns	No concerns	Low
EDU vs MUI	Some concerns	Low risk	No concerns	Major concerns	No concerns	No concerns	Very low
EDU vs Medi	Some concerns	Low risk	No concerns	Major concerns	No concerns	No concerns	Very low
EDU vs TM	Some concerns	Some concerns	No concerns	Major concerns	No concerns	No concerns	Very low
ES vs MBT	Some concerns	Some concerns	No concerns	No concerns	No concerns	No concerns	Low
ES vs ME	Some concerns	Some concerns	No concerns	Some concerns	Some concerns	No concerns	Very low
ES vs MUI	Some concerns	Some concerns	No concerns	No concerns	Major concerns	No concerns	Very low
ES vs Medi	Some concerns	Some concerns	No concerns	Some concerns	Some concerns	No concerns	Very low
ES vs PI	Some concerns	Some concerns	No concerns	No concerns	Major concerns	No concerns	Very low
ES vs TM	Some concerns	Some concerns	No concerns	Major concerns	No concerns	No concerns	Very low
KT vs MBT	Some concerns	Some concerns	No concerns	No concerns	No concerns	No concerns	Low
KT vs ME	Some concerns	Some concerns	No concerns	No concerns	Major concerns	No concerns	Very low
KT vs Medi	Some concerns	Some concerns	No concerns	Some concerns	Some concerns	No concerns	Very low
KT vs PI	Some concerns	Some concerns	No concerns	No concerns	Some concerns	No concerns	Very low
KT vs TM	Some concerns	Some concerns	No concerns	Major concerns	No concerns	No concerns	Very low
KT vs UC	Some concerns	Some concerns	No concerns	Major concerns	No concerns	No concerns	Very low
MBT vs ME	No concerns	Low risk	No concerns	No concerns	No concerns	No concerns	High
MBT vs MUI	Some concerns	Low risk	No concerns	No concerns	Some concerns	No concerns	Low
MBT vs Medi	Some concerns	Low risk	No concerns	No concerns	Some concerns	No concerns	Low
MBT vs PI	Some concerns	Low risk	No concerns	No concerns	Some concerns	No concerns	Low
MBT vs TM	Some concerns	Some concerns	No concerns	No concerns	No concerns	No concerns	Low
ME vs Medi	Some concerns	Low risk	No concerns	Major concerns	No concerns	No concerns	Very low
ME vs PI	No concerns	Low risk	No concerns	Major concerns	No concerns	No concerns	Low
ME vs TM	Some concerns	Some concerns	No concerns	Major concerns	No concerns	No concerns	Very low

Medi vs MUI	Some concerns	Low risk	No concerns	Major concerns	No concerns	No concerns	Very low
MUI vs PI	Some concerns	Low risk	No concerns	Major concerns	No concerns	No concerns	Very low
MUI vs TM	Some concerns	Some concerns	No concerns	Major concerns	No concerns	No concerns	Very low
Medi vs PI	Some concerns	Low risk	No concerns	Major concerns	No concerns	No concerns	Very low
Medi vs TM	Some concerns	Some concerns	No concerns	Major concerns	No concerns	No concerns	Very low
PI vs TM	Some concerns	Some concerns	No concerns	Major concerns	No concerns	No concerns	Very low

Appendix 14. Certainty of evidence ratings on indirect and network evidence of different specific treatments for mental health

Comparison	Within-study bias	Reporting bias	Indirectness	Imprecision	Heterogeneity	Incoherence	Confidence rating
**Mixed evidence**							
AC vs AT	No concerns	Low risk	No concerns	No concerns	Major concerns	No concerns	Low
AC vs EDU	Major concerns	Some concerns	No concerns	Some concerns	Some concerns	No concerns	Very low
AC vs TM	Some concerns	Low risk	No concerns	Some concerns	Some concerns	No concerns	Very low
AT vs MUI	Major concerns	Some concerns	No concerns	Major concerns	No concerns	No concerns	Very low
AT vs UC	Major concerns	Some concerns	No concerns	Major concerns	No concerns	No concerns	Very low
EDU vs UC	Some concerns	Low risk	No concerns	Major concerns	No concerns	No concerns	Very low
MUI vs UC	Some concerns	Low risk	No concerns	No concerns	Major concerns	No concerns	Very low
Medi vs UC	Some concerns	Low risk	No concerns	Major concerns	No concerns	No concerns	Very low
TM vs UC	Some concerns	Some concerns	No concerns	Major concerns	No concerns	No concerns	Very low
**Indirect evidence**							
AC vs MUI	Major concerns	Low risk	No concerns	No concerns	Major concerns	No concerns	Very low
AC vs Medi	Some concerns	Some concerns	No concerns	Some concerns	Some concerns	No concerns	Very low
AC vs UC	Some concerns	Low risk	No concerns	Major concerns	No concerns	No concerns	Very low
AT vs EDU	Major concerns	Low risk	No concerns	Major concerns	No concerns	No concerns	Very low
AT vs Medi	Some concerns	Some concerns	No concerns	Major concerns	No concerns	No concerns	Very low
AT vs TM	Some concerns	Some concerns	No concerns	Major concerns	No concerns	No concerns	Very low
EDU vs MUI	Some concerns	Low risk	No concerns	Major concerns	No concerns	No concerns	Very low
EDU vs Medi	Some concerns	Some concerns	No concerns	Major concerns	No concerns	No concerns	Very low
EDU vs TM	Some concerns	Some concerns	No concerns	Major concerns	No concerns	No concerns	Very low
Medi vs MUI	Some concerns	Low risk	No concerns	Major concerns	No concerns	No concerns	Very low
MUI vs TM	Some concerns	Some concerns	No concerns	Major concerns	No concerns	No concerns	Very low
Medi vs TM	Some concerns	Some concerns	No concerns	Major concerns	No concerns	No concerns	Very low

Appendix 15. Pooled standardized mean difference and heterogeneity for each direct comparison of different categories of interventions for depression

Comparison	Number of RCTs	Number of participants	(Pooled) SMD (95% CI)	I square (%)	P value
EX vs CO	7	412	-0.21 (-0.40, -0.01)	0	0.040
MBT vs CO	6	321	-1.28 (-2.11, -0.44)	90	0.003
EDU vs CO	4	649	-0.24 (-0.61, 0.13)	71	0.200
TM vs CO	3	131	0.08 (-0.48, 0.63)	59	0.790
BA vs CO	9	645	-0.42 (-0.73, -0.12)	71	0.007
MUI vs CO	5	636	-0.13 (-0.29, 0.02)	47	0.100
PT vs CO	16	785	-0.25 (-0.39, -0.10)	35	＜0.001
BA vs EDU	1	180	-0.39 (-0.71, -0.07)	NA	NA
BA vs EX	1	28	0.36 (-0.39, 1.11)	NA	NA
EX vs MUI	2	57	0.34 (-1.25, 1.93)	88	0.670
EX vs PT	4	123	0.13 (-0.47, 0.74)	63	0.660
MUI vs PT	1	22	-0.98 (-1.88, -0.09)	NA	NA
BA vs MUI	1	32	-0.05 (-0.75, 0.65)	NA	NA

Appendix 16 . Comparative effectiveness of different categories of interventions: surface under the cumulative ranking curves (SUCRA) for depression

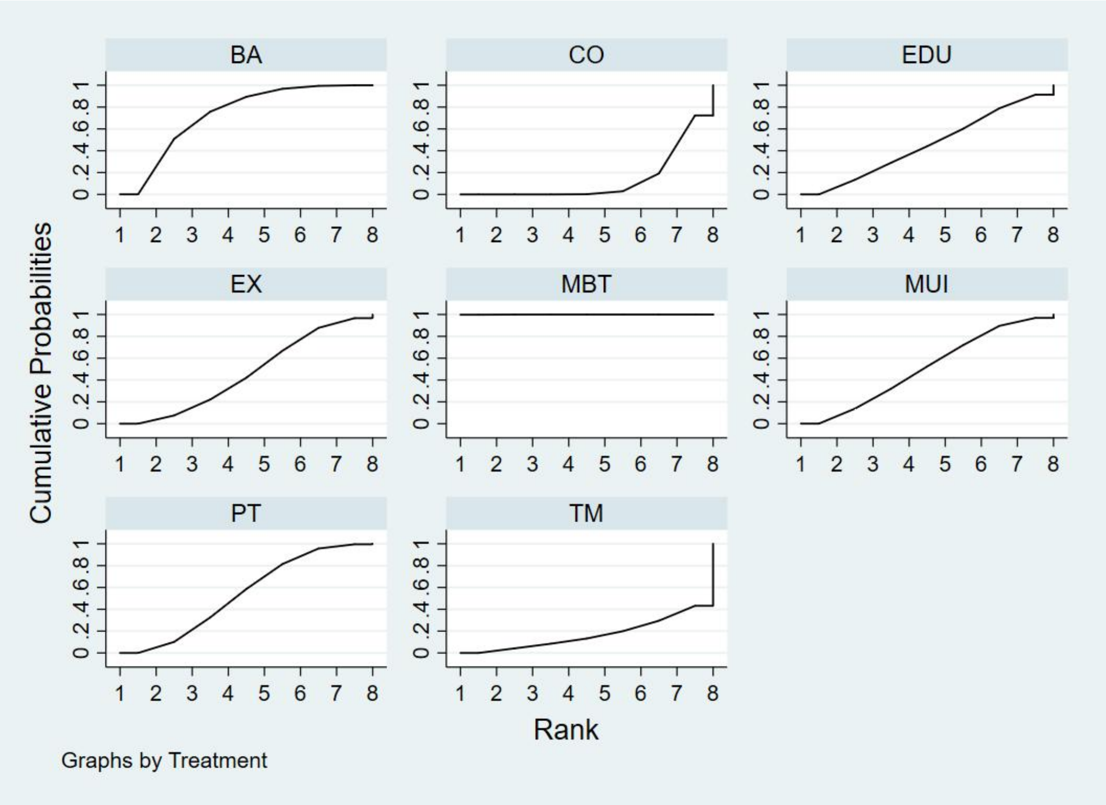

Appendix 17. Network plot of subgroup comparisons in the network meta-analysis of specific treatments for depression. Nodes' sizes and line widths represent the number of randomized patients and controlled trials for each treatment, respectively. The size of the node corresponds to the number of patients randomized to each treatment, whereas the line width indicates the number of randomized controlled trials comparing each pair of treatments. MBT, Mind body therapy; Mass, Massage; CBT, Cognitive-behavioural treatments; PI, Psychosocial intervention; RT, Relaxation training; Acup, Acupuncture; Medi, Meditation; MUI, Multicomponent intervention; EDU, Education; AT, Aerobic training; SE, Stabilization exercise; ME, Mixed exercise training; ES, Electrical stimulation; AC, Active control; UC, Usual care; KT, Kinesiology taping; TM, Telemedicine. Interventions details are described in Appendix 3.

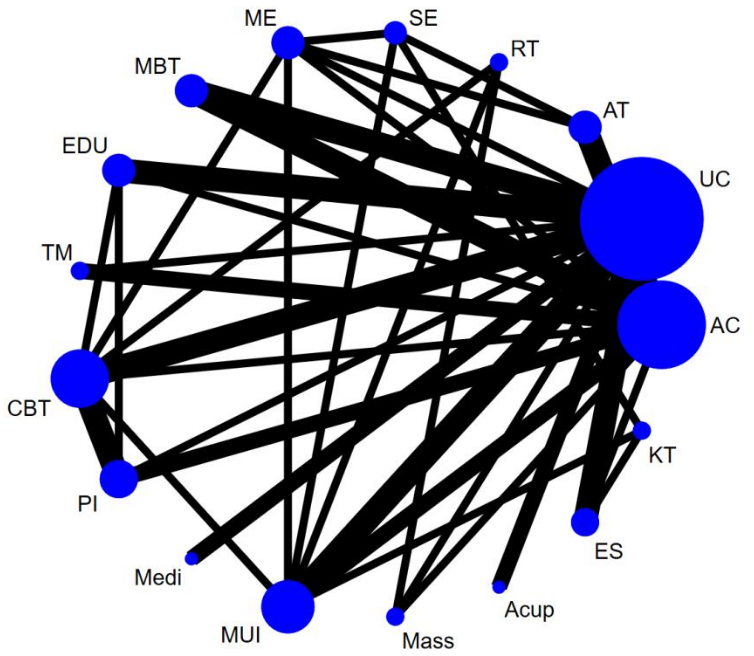

Appendix 18. Subgroup analysis of comparative effectiveness of specific treatments for depression

**MBT**	NA	NA	NA	NA	NA	NA	NA	NA	NA	NA	NA	NA	-1.46 (-3.03,0.11)	**-1.03 (-1.68,-0.37)**	NA	NA
-0.50 (-1.35,0.34)	**Mass**	NA	NA	-0.76 (-1.59,0.08)	NA	NA	NA	NA	NA	NA	NA	NA	-0.55 (-1.18,0.09)	-0.54 (-1.07,0.00)	NA	NA
**-0.77 (-1.39,-0.15)**	-0.27 (-1.05,0.52)	**CBT**	-0.03 (-0.47,0.41)	0.36 (-0.39,1.11)	NA	NA	-0.05 (-0.75,0.65)	-0.27 (-0.64,0.09)	NA	NA	0.17 (-0.23,0.57)	NA	0.09 (-0.34,0.51)	**-0.82 (-1.59,-0.05)**	NA	NA
**-0.82 (-1.48,-0.16)**	-0.31 (-1.14,0.52)	-0.05 (-0.53,0.44)	**PI**	NA	NA	NA	NA	-0.50 (-0.86,-0.13)	NA	NA	NA	NA	**-0.43 (-0.82,-0.05)**	-0.31 (-0.86,0.23)	NA	NA
-0.81 (-1.79,0.17)	-0.30 (-1.20,0.60)	-0.04 (-0.92,0.85)	0.01 (-0.94,0.96)	**RT**	NA	NA	-0.45 (-1.16,0.26)	NA	NA	NA	NA	NA	NA	NA	NA	NA
-0.85 (-1.78,0.09)	-0.34 (-1.41,0.72)	-0.08 (-0.96,0.81)	-0.03 (-0.96,0.89)	-0.04 (-1.21,1.13)	**Acup**	NA	NA	NA	NA	NA	NA	NA	NA	-0.41 (-0.84,0.01)	NA	NA
-0.86 (-1.77,0.05)	-0.36 (-1.40,0.68)	-0.09 (-0.95,0.77)	-0.05 (-0.95,0.86)	-0.06 (-1.21,1.10)	-0.01 (-1.10,1.07)	**Medi**	NA	NA	NA	NA	NA	NA	NA	-0.36 (-1.27,0.56)	NA	NA
**-0.98 (-1.59,-0.36)**	-0.47 (-1.25,0.31)	-0.21 (-0.73,0.32)	-0.16 (-0.75,0.43)	-0.17 (-1.05,0.71)	-0.13 (-1.02,0.76)	-0.12 (-0.98,0.75)	**MUI**	NA	NA	-1.17 (-2.03,-0.31)	-0.26 (-0.64,0.11)	NA	0.05 (-0.23,0.32)	-0.24 (-0.52,0.04)	-0.98 (-1.88,-0.09)	NA
**-1.01 (-1.67,-0.35)**	-0.51 (-1.33,0.32)	-0.24 (-0.79,0.31)	-0.19 (-0.78,0.40)	-0.20 (-1.16,0.76)	-0.16 (-1.07,0.74)	-0.15 (-1.03,0.73)	-0.03 (-0.62,0.56)	**EDU**	NA	NA	NA	NA	-1.02 (-1.61,-0.43)	-0.08 (-0.24,0.08)	NA	NA
**-1.04 (-1.74,-0.34)**	-0.53 (-1.41,0.34)	-0.27 (-0.93,0.39)	-0.22 (-0.92,0.48)	-0.23 (-1.24,0.77)	-0.19 (-1.17,0.79)	-0.18 (-1.13,0.78)	-0.06 (-0.70,0.58)	-0.03 (-0.73,0.68)	**AT**	0.47 (-0.37,1.31)	-0.25 (-1.04,0.54)	NA	-0.12 (-0.49,0.25)	-0.58 (-1.44,0.27)	NA	NA
**-1.09 (-2.05,-0.13)**	-0.58 (-1.67,0.50)	-0.32 (-1.23,0.59)	-0.27 (-1.22,0.68)	-0.28 (-1.46,0.89)	-0.24 (-1.40,0.92)	-0.23 (-1.37,0.91)	-0.11 (-0.96,0.74)	-0.08 (-1.03,0.87)	-0.05 (-0.94,0.84)	**SE**	-0.65 (-1.52,0.22)	NA	NA	NA	0.00 (-0.83,0.82)	NA
**-1.09 (-1.82,-0.36)**	-0.59 (-1.47,0.30)	-0.32 (-0.96,0.31)	-0.28 (-0.98,0.43)	-0.29 (-1.29,0.71)	-0.24 (-1.22,0.74)	-0.23 (-1.18,0.72)	-0.11 (-0.73,0.50)	-0.08 (-0.79,0.63)	-0.05 (-0.76,0.65)	-0.00 (-0.90,0.89)	**ME**	NA	-0.07 (-0.45,0.32)	-0.34 (0.72,0.04)	NA	NA
**-1.16 (-1.88,-0.44)**	-0.66 (-1.54,0.22)	-0.39 (-1.06,0.27)	-0.35 (-1.06,0.36)	-0.36 (-1.36,0.65)	-0.31 (-1.27,0.64)	-0.30 (-1.23,0.63)	-0.19 (-0.83,0.46)	-0.15 (-0.85,0.54)	-0.12 (-0.88,0.63)	-0.07 (-1.02,0.87)	-0.07 (-0.84,0.69)	**ES**	-0.42 (-1.05,0.21)	0.10 (-0.27,0.46)	-0.01 (-0.52,0.50)	NA
**-1.22 (-1.72,-0.72)**	-0.71 (-1.44,0.01)	-0.45 (-0.92,0.02)	-0.40 (-0.91,0.10)	-0.41 (-1.30,0.48)	-0.37 (-1.24,0.50)	-0.36 (-1.20,0.49)	-0.24 (-0.69,0.21)	-0.21 (-0.74,0.32)	-0.18 (-0.71,0.35)	-0.13 (-0.99,0.73)	-0.13 (-0.72,0.47)	-0.06 (-0.66,0.55)	**AC**	NA	NA	-0.12 (-1.05,0.80)
**-1.22 (-1.74,-0.71)**	-0.72 (-1.44,0.00)	**-0.45 (-0.87,-0.03)**	-0.41 (-0.90,0.09)	-0.42 (-1.29,0.46)	-0.37 (-1.15,0.41)	-0.36 (-1.11,0.39)	-0.24 (-0.67,0.18)	-0.21 (-0.67,0.25)	-0.18 (-0.77,0.41)	-0.13 (-0.99,0.73)	-0.13 (-0.72,0.46)	-0.06 (-0.60,0.49)	-0.00 (-0.39,0.38)	**UC**	NA	-0.02 (-0.70,0.65)
**-1.33 (-2.30,-0.35)**	-0.82 (-1.92,0.27)	-0.56 (-1.49,0.37)	-0.51 (-1.48,0.46)	-0.52 (-1.71,0.67)	-0.48 (-1.64,0.69)	-0.47 (-1.61,0.68)	-0.35 (-1.22,0.52)	-0.32 (-1.28,0.65)	-0.29 (-1.27,0.69)	-0.24 (-1.23,0.76)	-0.24 (-1.21,0.74)	-0.16 (-0.99,0.67)	-0.11 (-1.00,0.78)	-0.11 (-0.97,0.76)	**KT**	NA
**-1.30 (-2.10,-0.51)**	-0.80 (-1.75,0.15)	-0.53 (-1.30,0.23)	-0.49 (-1.28,0.31)	-0.50 (-1.57,0.58)	-0.46 (-1.50,0.59)	-0.44 (-1.46,0.58)	-0.33 (-1.08,0.43)	-0.29 (-1.09,0.50)	-0.27 (-1.09,0.56)	-0.22 (-1.27,0.84)	-0.21 (-1.06,0.64)	-0.14 (-0.99,0.71)	-0.08 (-0.73,0.57)	-0.08 (-0.77,0.61)	0.02 (-1.05,1.10)	**TM**

Certainty of Evidence **High** **Moderate** **Low** **Very Low**

Notes: The league tables show the pooled outcomes of the network meta-analyses (lower diagonal) and pairwise meta-analyses (upper diagonal) for subgroup analysis of comparative effectiveness of specific treatments for depression. The relative effect sizes of each approach were measured as a standardized mean difference and 95% confidence intervals. Bold indicates statistical significance. Comparisons between treatments should be read from left to right, and the estimate is in the cell in common between the column-defining treatment and the row-defining treatment. The imprecision for the rating of Certainty of Evidence on direct evidence was not considered. (According to the GRADE, recommended "consideration of imprecision is not necessary when rating the direct and indirect estimates to inform the rating of the network estimates".) The detailed of Certainty of Evidence were presented in Appendices 8 and 12. NA, Not available; MBT, Mind body therapy; Mass, Massage; CBT, Cognitive-behavioural treatments; PI, Psychosocial intervention; RT, Relaxation training; Acup, Acupuncture; Medi, Meditation; MUI, Multicomponent intervention; EDU, Education; AT, Aerobic training; SE, Stabilization exercise; ME, Mixed exercise training; ES, Electrical stimulation; AC, Active control; UC, Usual care; KT, Kinesiology taping; TM, Telemedicine. Interventions details are described in Appendix 3.

Appendix 19. Subgroup analysis of comparative effectiveness of specific treatments: surface under the cumulative ranking curves (SUCRA) for depression

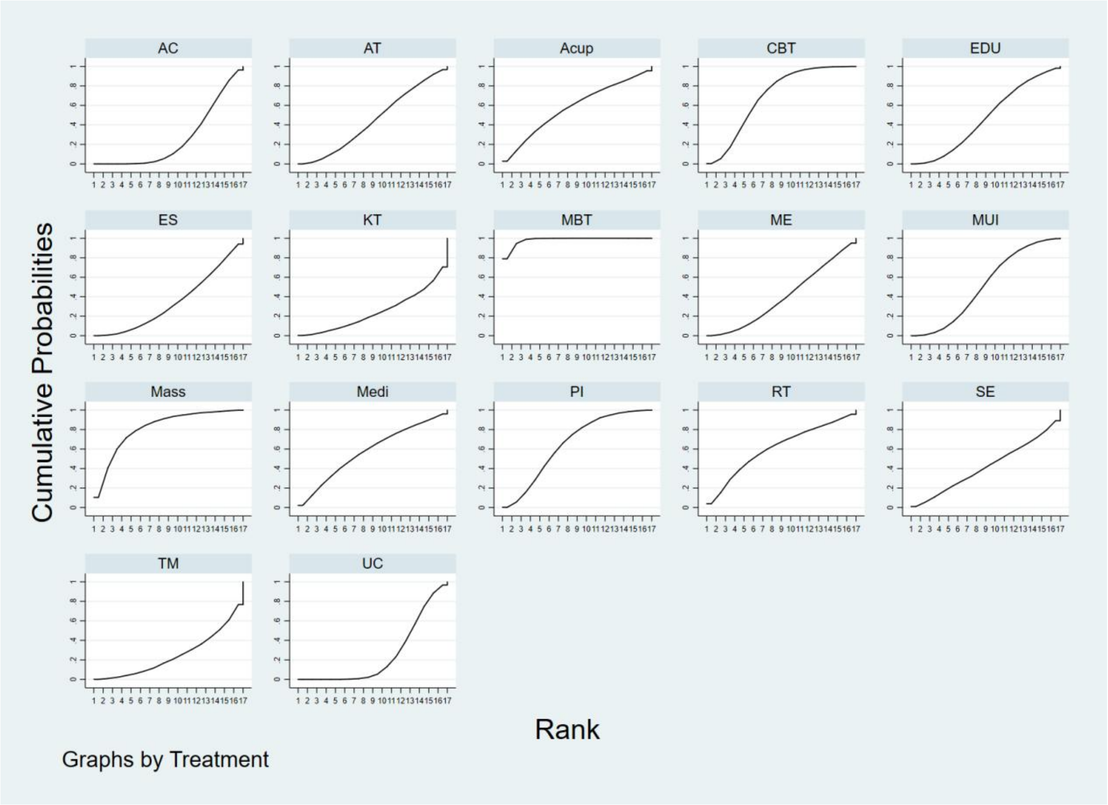

Appendix 20. Pooled standardized mean difference and heterogeneity for each direct comparison of different categories of interventions for anxiety

Comparison	Number of RCTs	Number of participants	(Pooled) SMD (95% CI)	I square (%)	P value
EX vs CO	2	216	-0.27 (-0.54, 0.00)	11	0.050
MBT vs CO	3	187	-1.34 (-1.66, -1.02)	0	＜0.001
EDU vs CO	3	205	-0.55 (-1.04, -0.06)	66	0.030
TE vs CO	2	71	-0.03 (-0.50, 0.44)	0	0.900
BA vs CO	7	499	-0.47 (-0.88, -0.06)	79	0.030
MUI vs CO	4	327	-0.41 (-0.63, -0.19)	24	＜0.001
PT vs CO	2	74	0.33 (-0.13, 0.79)	0	0.160
EX vs MUI	2	138	0.59 (-0.14, 1.33)	63	0.110
EX vs PT	2	47	0.35 (-0.83, 1.53)	74	0.560
BA vs EDU	1	180	-0.29 (-0.60, 0.03)	NA	NA
MUI vs PT	1	22	-1.53 (-2.51, -0.55)	NA	NA
BA vs EX	1	102	0.04 (-0.36, 0.43)	NA	NA

Appendix 21. Comparative effectiveness of different categories of interventions: surface under the cumulative ranking curves (SUCRA) for anxiety

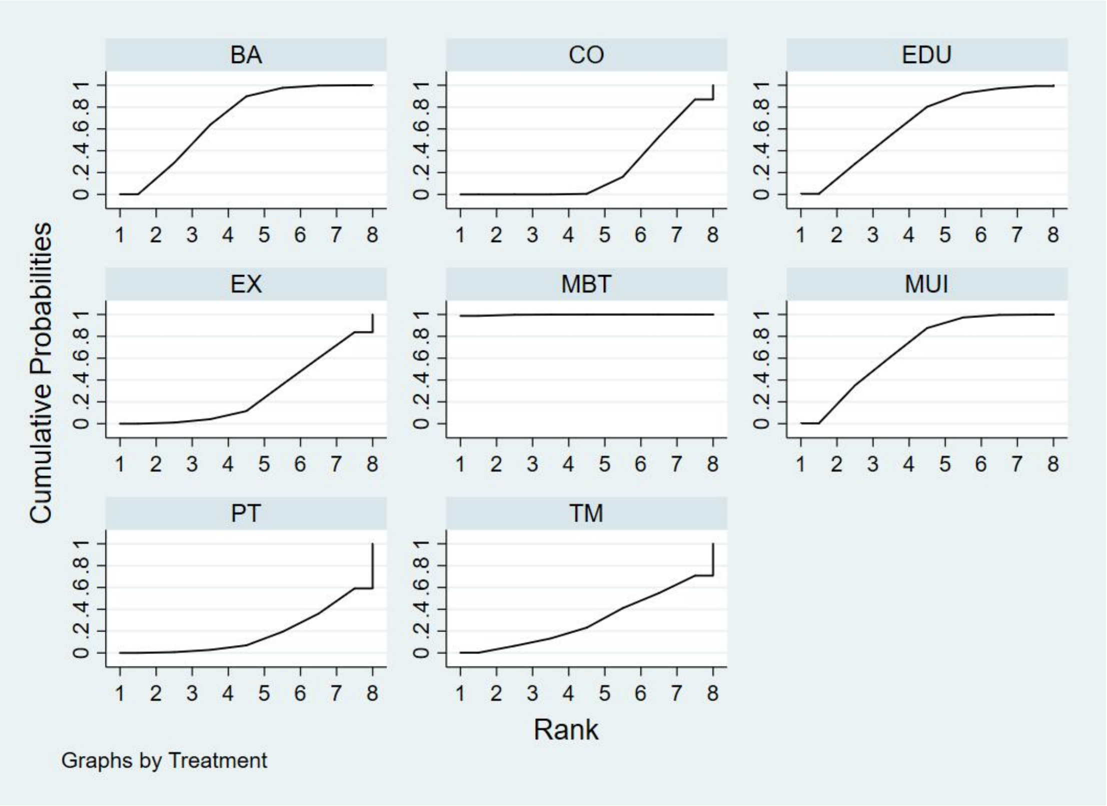

Appendix 22. Network plot of subgroup comparisons in the network meta-analysis of specific treatments for anxiety. Nodes' sizes and line widths represent the number of randomized patients and controlled trials for each treatment, respectively. The size of the node corresponds to the number of patients randomized to each treatment, whereas the line width indicates the number of randomized controlled trials comparing each pair of treatments. MBT, Mind body therapy; CBT, Cognitive-behavioural treatments; PI, Psychosocial intervention; MUI, Multicomponent intervention; EDU, Education; Medi, Meditation; ME, Mixed exercise training; AC, Active control; TM, Telemedicine; UC, Usual care; ES, Electrical stimulation; KT, Kinesiology taping. Interventions details are described in Appendix 3.

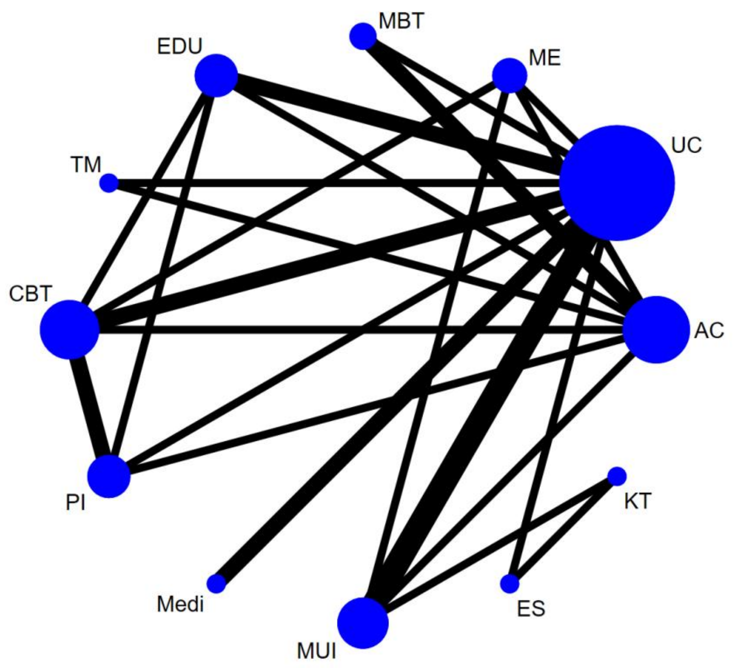

Appendix 23. Subgroup analysis of comparative effectiveness of specific treatments for anxiety

**MBT**	NA	NA	NA	NA	NA	NA	**-1.34 (-1.69,-0.99)**	NA	-1.33 (-2.13,-0.53)	NA	NA
**-0.90 (-1.59,-0.21)**	**CBT**	0.19 (-0.14,0.51)	NA	-0.20 (-0.56,0.17)	NA	0.04 (-0.36,0.43)	-0.08 (-0.50,0.35)	NA	-1.38 (-3.32,0.56)	NA	NA
**-0.95 (-1.65,-0.24)**	-0.05 (-0.54,0.45)	**PI**	NA	-0.38 (-0.74,-0.02)	NA	NA	-0.27 (-0.73,0.18)	NA	0.00 (-0.54,0.54)	NA	NA
**-1.00 (-1.69,-0.31)**	-0.10 (-0.66,0.46)	-0.05 (-0.65,0.55)	**MUI**	NA	NA	-0.31 (-0.68,0.06)	-0.20 (-0.58,0.17)	NA	**-0.51 (-0.78,-0.24)**	NA	-1.53 (-2.51,-0.55)
**-1.01 (-1.70,-0.31)**	-0.11 (-0.62,0.41)	-0.06 (-0.60,0.48)	-0.01 (-0.58,0.57)	**EDU**	NA	NA	-0.74 (-1.31,-0.16)	NA	-0.46 (-1.24,0.32)	NA	NA
**-1.09 (-1.97,-0.22)**	-0.19 (-0.96,0.57)	-0.15 (-0.94,0.64)	-0.10 (-0.84,0.65)	-0.09 (-0.85,0.68)	**Medi**	NA	NA	NA	-0.41 (-1.43,0.61)	NA	NA
**-1.10 (-1.84,-0.35)**	-0.20 (-0.78,0.39)	-0.15 (-0.80,0.50)	-0.10 (-0.67,0.48)	-0.09 (-0.73,0.55)	-0.00 (-0.82,0.82)	**ME**	-0.12 (-0.50,0.27)	NA	-1.33 (-2.13,-0.53)	NA	NA
**-1.30 (-1.83,-0.76)**	-0.40 (-0.90,0.11)	-0.35 (-0.86,0.17)	-0.30 (-0.80,0.20)	-0.29 (-0.80,0.22)	-0.20 (-0.96,0.56)	-0.20 (-0.76,0.37)	**AC**	-0.13 (-0.78,0.52)	NA	NA	NA
**-1.37 (-2.24,-0.51)**	-0.47 (-1.27,0.33)	-0.42 (-1.24,0.39)	-0.37 (-1.16,0.42)	-0.36 (-1.16,0.44)	-0.28 (-1.22,0.67)	-0.27 (-1.12,0.57)	-0.08 (-0.79,0.64)	**TM**	-0.20 (-0.88,0.47)	NA	NA
**-1.52 (-2.14,-0.90)**	**-0.62 (-1.06,-0.17)**	**-0.57 (-1.06,-0.08)**	**-0.52 (-0.94,-0.10)**	**-0.51 (-0.95,-0.06)**	-0.42 (-1.04,0.20)	-0.42 (-0.96,0.12)	-0.22 (-0.66,0.22)	-0.15 (-0.86,0.57)	**UC**	-0.19 (-1.05,0.67)	NA
**-2.01 (-3.09,-0.94)**	**-1.11 (-2.10,-0.12)**	**-1.07 (-2.08,-0.05)**	**-1.01 (-1.94,-0.09)**	**-1.01 (-2.00,-0.01)**	-0.92 (-2.01,0.17)	-0.92 (-1.94,0.11)	-0.72 (-1.69,0.26)	-0.64 (-1.78,0.50)	-0.50 (-1.39,0.40)	**ES**	0.05 (-0.47,0.56)
**-2.15 (-3.26,-1.05)**	**-1.25 (-2.29,-0.22)**	**-1.21 (-2.26,-0.16)**	**-1.16 (-2.09,-0.22)**	**-1.15 (-2.18,-0.11)**	-1.06 (-2.19,0.07)	-1.06 (-2.11,-0.00)	-0.86 (-1.87,0.15)	-0.78 (-1.95,0.39)	-0.64 (-1.58,0.31)	-0.14 (-0.91,0.63)	**KT**

Certainty of Evidence — **High** — **Moderate** — **Low** — **Very Low**

Notes: The league tables show the pooled outcomes of the network meta-analyses (lower diagonal) and pairwise meta-analyses (upper diagonal) for subgroup analysis of comparative effectiveness of specific treatments for anxiety. The relative effect sizes of each approach were measured as a standardized mean difference and 95% confidence intervals. Bold indicates statistical significance. Comparisons between treatments should be read from left to right, and the estimate is in the cell in common between the column-defining treatment and the row-defining treatment. The imprecision for the rating of Certainty of Evidence on direct evidence was not considered. (According to the GRADE, recommended "consideration of imprecision is not necessary when rating the direct and indirect estimates to inform the rating of the network estimates".) The detailed of Certainty of Evidence were presented in Appendices 8 and 13. NA, Not available; MBT, Mind body therapy; CBT, Cognitive-behavioural treatments; PI, Psychosocial intervention; MUI, Multicomponent intervention; EDU, Education; Medi, Meditation; ME, Mixed exercise training; AC, Active control; TM, Telemedicine; UC, Usual care; ES, Electrical stimulation; KT, Kinesiology taping. Interventions details are described in Appendix 3.

Appendix 24. Subgroup analysis of comparative effectiveness of specific treatments: surface under the cumulative ranking curves (SUCRA) for anxiety

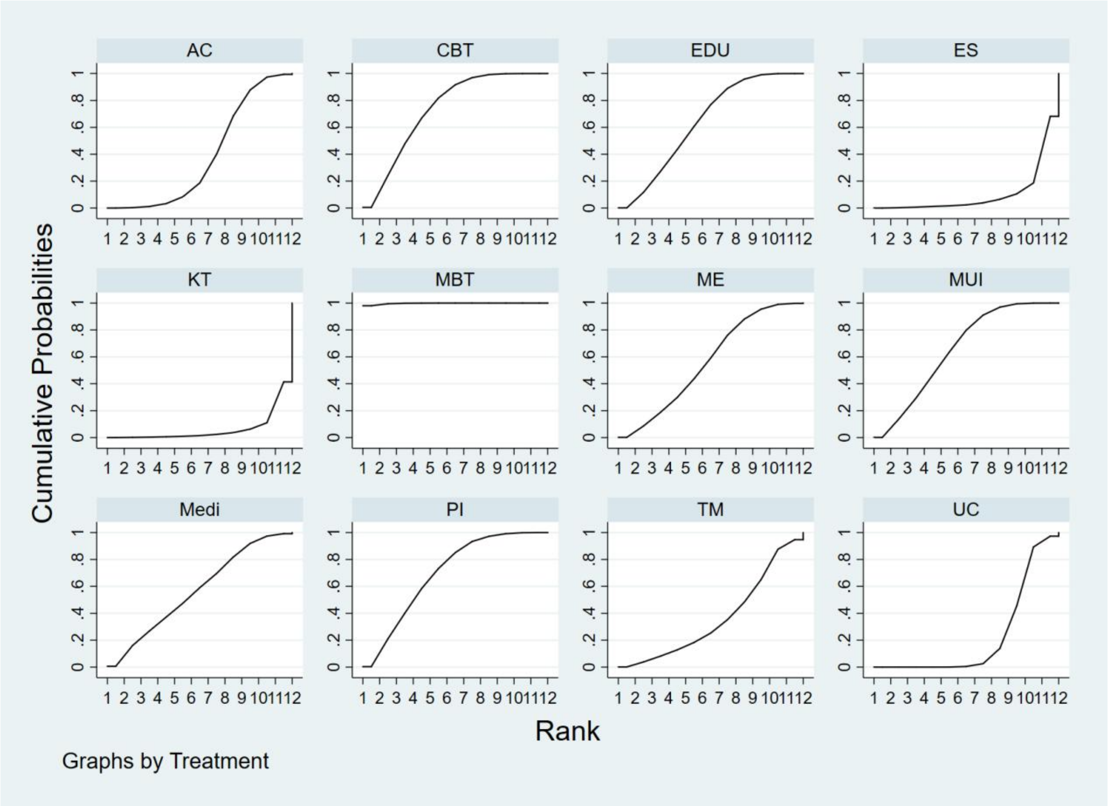

Appendix 25. Pooled standardized mean difference and heterogeneity for each direct comparison of different categories of interventions for mental health

Comparison	Number of RCTs	Number of participants	(Pooled) SMD (95% CI)	I square (%)	P value
EX vs CO	6	327	0.56 (0.01, 1.11)	82	0.050
EDU vs CO	4	39	0.30 (-0.11, 0.71)	76	0.150
TE vs CO	3	145	0.41 (-0.69, 1.50)	90	0.470
BA vs CO	2	119	0.43 (-0.29, 1.14)	73	0.250
MUI vs CO	4	360	0.60 (0.01, 1.19)	83	0.040
PT vs CO	8	562	0.47 (0.11, 0.83)	75	0.010
EX vs MUI	2	162	-0.47 (-1.31, 0.37)	84	0.270
EX vs PT	1	39	0.44 (-0.19, 1.08)	NA	NA

Appendix 26. Comparative effectiveness of different categories of interventions: surface under the cumulative ranking curves (SUCRA) for mental health

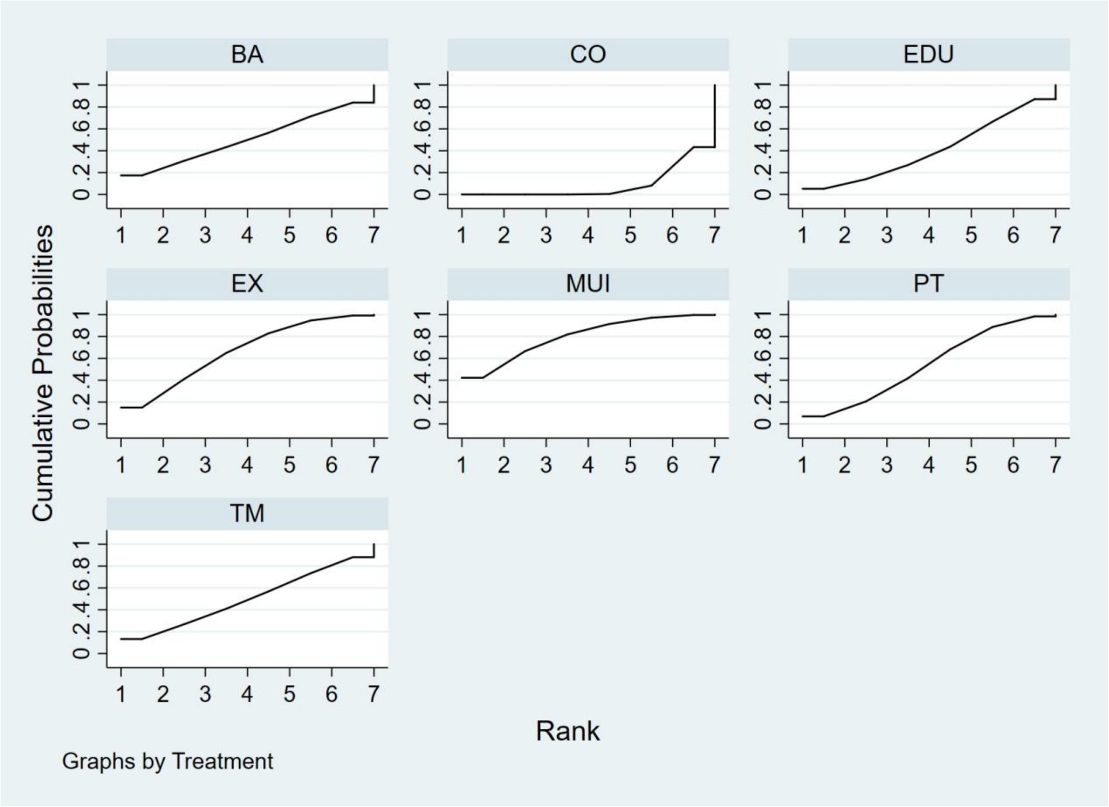

Appendix 27. Network plot of subgroup comparisons in the network meta-analysis of specific treatments for mental health. Nodes' sizes and line widths represent the number of randomized patients and controlled trials for each treatment, respectively. The size of the node corresponds to the number of patients randomized to each treatment, whereas the line width indicates the number of randomized controlled trials comparing each pair of treatments. MUI, Multicomponent intervention; AT, Aerobic training; Medi, Meditation; EDU, Education; TM, Telemedicine; UC, Usual care; AC, Active control. Interventions details are described in Appendix 3.

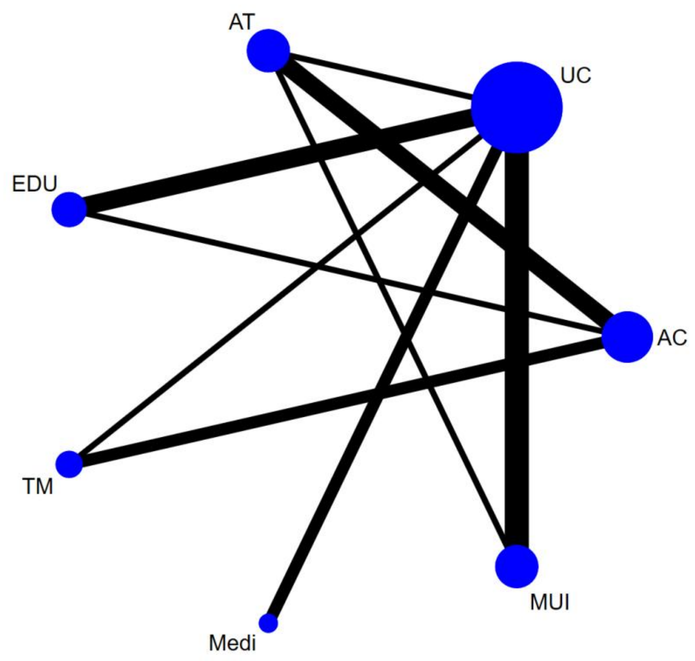

Appendix 28. Subgroup analysis of comparative effectiveness of specific treatments for mental health

**MUI**	0.02 (-0.54,0.58)	NA	NA	NA	**0.60 (0.01,1.19)**	NA
0.23 (-0.76,1.22)	**AT**	NA	NA	NA	-0.04 (-0.61,0.53)	**1.03 (0.03,2.02)**
0.22 (-0.91,1.35)	-0.01 (-1.30,1.27)	**Medi**	NA	NA	0.43 (-0.29,1.14)	NA
0.46 (-0.46,1.37)	0.23 (-0.78,1.23)	0.24 (-0.90,1.37)	**EDU**	NA	0.24 (-0.26,0.73)	0.57 (-0.01,1.14)
0.60 (-0.53,1.73)	0.37 (-0.65,1.39)	0.38 (-0.96,1.72)	0.14 (-0.93,1.22)	**TM**	0.02 (-0.66,0.69)	0.59 (-1.05,2.23)
0.64 (-0.02,1.30)	0.41 (-0.49,1.31)	0.42 (-0.50,1.34)	0.18 (-0.48,0.85)	0.04 (-0.94,1.02)	**UC**	NA
**1.20 (0.19,2.22)**	**0.97 (0.25,1.69)**	0.98 (-0.29,2.26)	0.75 (-0.18,1.67)	0.60 (-0.22,1.42)	0.56 (-0.32,1.44)	**AC**

Certainty of Evidence **Moderate** **Low** **Very Low**

Notes: The league tables show the pooled outcomes of the network meta-analyses (lower diagonal) and pairwise meta-analyses (upper diagonal) for subgroup analysis of comparative effectiveness of specific treatments for mental health. The relative effect sizes of each approach were measured as a standardized mean difference and 95% confidence intervals. Bold indicates statistical significance. Comparisons between treatments should be read from left to right, and the estimate is in the cell in common between the column-defining treatment and the row-defining treatment. The imprecision for the rating of Certainty of Evidence on direct evidence was not considered. (According to the GRADE, recommended "consideration of imprecision is not necessary when rating the direct and indirect estimates to inform the rating of the network estimates".) The detailed of Certainty of Evidence were presented in Appendices 8 and 14. NA, Not available; MUI, Multicomponent intervention; AT, Aerobic training; Medi, Meditation; EDU, Education; TM, Telemedicine; UC, Usual care; AC, Active control. Interventions details are described in Appendix 3.

Appendix 29. Subgroup analysis of comparative effectiveness of specific treatments: surface under the cumulative ranking curves (SUCRA) for mental health

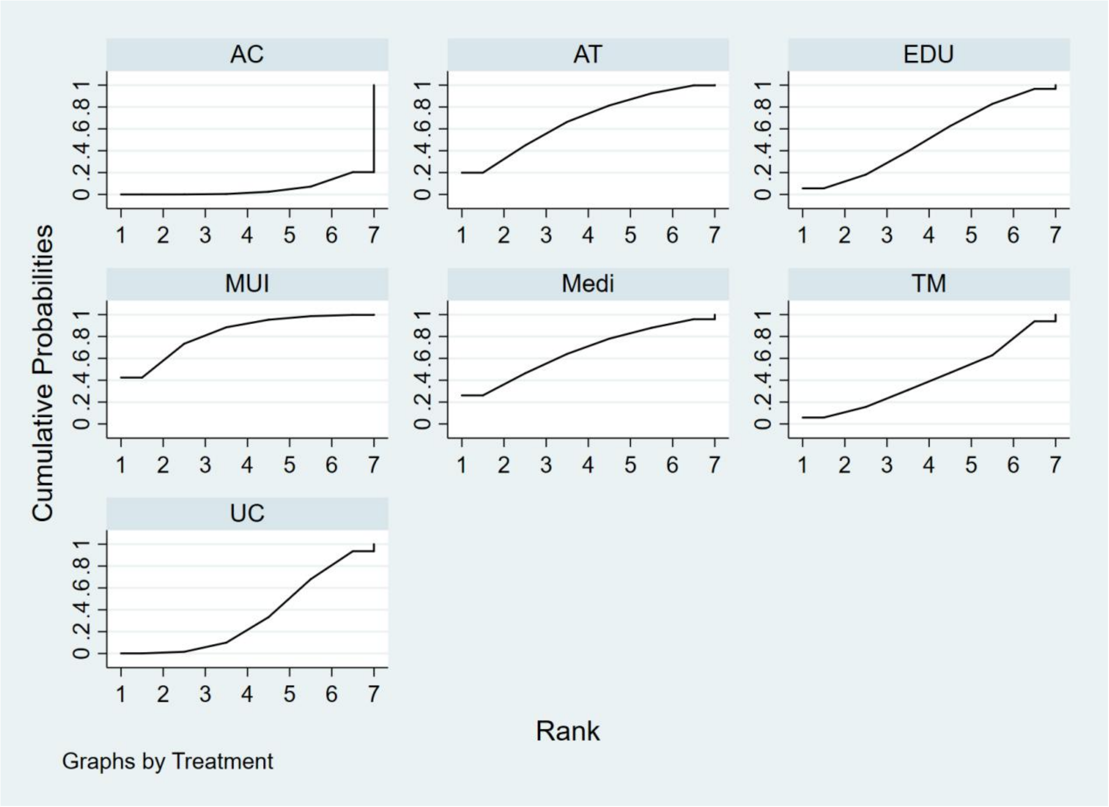

Appendix 30. Evaluation of global inconsistency for different categories of interventions and specific treatments

Psychological symptoms	Chi^2	P value
**Different categories of interventions**		
Depression	8.82	0.7183
Anxiety	9.55	0.2156
Mental health	3.36	0.4991
**Subgroup analyses based on different specific treatments**		
Depression	8.79	0.9645
Anxiety	3.67	0.9785
Mental health	1.15	0.7649

Appendix 31. Node-splitting method in comparison between direct and indirect evidence of different categories of interventions for depression

Comparison	Direct Effect		Indirect Effect		Difference		P value	tau
	Estimate	Standard error	Estimate	Standard error	Estimate	Standard error		
CO vs. EX	-0.2238785	0.191136	-0.1912688	0.2788397	-0.0326097	0.3386809	0.923	0.4135227
CO vs. EDU	-0.2636933	0.2347405	0.0030666	0.4703508	-0.26676	0.5254906	0.612	0.4136125
CO vs. BA	-0.429138	0.1656994	-0.3291986	0.3355936	-0.0999395	0.3743801	0.790	0.4150547
CO vs. MUI	-0.1393617	0.1997302	-0.5853901	0.3465948	0.4460284	0.4001083	0.265	0.4072815
CO vs. PT	-0.2565262	0.128129	-0.2860057	0.3935137	0.0294795	0.4138499	0.943	0.4127692
EX vs. BA	0.2305791	0.3483616	-0.3952676	0.2395163	0.6258467	0.4230191	0.139	0.4008367
EX vs. MUI	-0.2213455	0.297895	0.1350992	0.2887244	-0.3564447	0.4146473	0.390	0.4077096
EX vs. PT	-0.1277523	0.2785884	0.013109	0.236638	-0.1408613	0.3657513	0.700	0.4129977
EDU vs. BA	-0.3893887	0.4440518	-0.1226291	0.2809961	-0.2667596	0.525491	0.612	0.4136127
BA vs. MUI	0.0691692	0.5454251	0.1748182	0.2397463	-0.105649	0.5959014	0.859	0.4131381
MUI vs. PT	0.9598683	0.5959836	-0.132617	0.2115876	1.092485	0.6324493	0.084	0.3951604

Notes: P>0.05 indicates that indirect comparisons were consistent with direct comparisons.

Appendix 32. Node-splitting method in comparison between direct and indirect evidence of different categories of interventions for anxiety

Comparison	Direct Effect		Indirect Effect		Difference		P value	tau
	Estimate	Standard error	Estimate	Standard error	Estimate	Standard error		
CO vs. EX	-0.291343	0.3004364	0.3604447	0.3758668	-0.6517877	0.4827641	0.177	0.3776345
CO vs. EDU	-0.5481524	0.2700621	-0.131147	0.4588453	-0.4170054	0.5326988	0.434	0.3907254
CO vs. BA	-0.4536069	0.1875453	-0.5610049	0.4621838	0.1073979	0.5005185	0.830	0.4078321
CO vs. MUI	-0.3967749	0.2312431	-0.9050779	0.5559385	0.508303	0.6023378	0.399	0.3971664
CO vs. PT	0.3097422	0.3674924	-0.2074823	0.4395846	0.5172245	0.5732524	0.367	0.383523
EX vs. BA	0.0356357	0.4338997	-0.7247304	0.3511723	0.760366	0.5595704	0.174	0.3841477
EX vs. MUI	-0.5369577	0.3561926	-0.3042274	0.4410474	-0.2327303	0.5633674	0.680	0.4040809
EX vs. PT	-0.3355244	0.3889144	0.7033601	0.4214818	-1.038885	0.5715559	0.069	0.3446975
EDU vs. BA	-0.2883337	0.4225842	0.1286711	0.3243312	-0.4170048	0.5326989	0.434	0.3907253
MUI vs. PT	1.31032	0.5925849	0.2750461	0.3710412	1.035274	0.6991315	0.139	0.3721446

Notes: P>0.05 indicates that indirect comparisons were consistent with direct comparisons.

Appendix 33. Node-splitting method in comparison between direct and indirect evidence of different categories of interventions for mental health

Comparison	Direct Effect		Indirect Effect		Difference		P value	tau
	Estimate	Standard error	Estimate	Standard error	Estimate	Standard error		
CO vs. EX	0.5480893	0.2799529	1.137774	0.9171429	-0.5896843	0.9589408	0.539	0.6103202
CO vs. MUI	0.5973917	0.3164986	2.287184	0.9731859	-1.689792	1.023408	0.099	0.5557674
CO vs. PT	0.4789188	0.2376082	0.2024246	1.338763	0.2764941	1.360019	0.839	0.6103269
EX vs. MUI	0.4570806	0.4449786	-0.2984623	0.5676288	0.7555429	0.7216117	0.295	0.5808482
EX vs. PT	-0.4827968	0.6853875	-0.0155989	0.3864681	-0.4671979	0.786849	0.553	0.6040267

Notes: P>0.05 indicates that indirect comparisons were consistent with direct comparisons.

Appendix 34. Subgroup analysis of node-splitting method in comparison between direct and indirect evidence of specific treatments for depression

Comparison	Direct Effect		Indirect Effect		Difference		P value	tau
	Estimate	Standard error	Estimate	Standard error	Estimate	Standard error		
A C	-0.1187627	0.3339613	-0.3101126	0.4797917	0.1913498	0.584577	0.743	0.4781059
A F	-0.0775088	0.5202451	-0.1533492	0.3849653	0.0758404	0.647973	0.907	0.4817116
A G	-1.366394	0.3224237	-0.9677237	0.4229854	-0.3986701	0.5312485	0.453	0.4760746
A H	-1.018301	0.541138	0.038676	0.2975878	-1.056977	0.6175669	0.087	0.4488797
A I	0.112371	0.399749	0.0193135	0.6257298	0.0930575	0.7425004	0.900	0.4799346
A J	0.0897105	0.5150758	-0.598249	0.2720819	0.6879594	0.5824498	0.238	0.4676164
A K	-0.3821205	0.4008368	-0.4205949	0.3476918	0.0384745	0.5307183	0.942	0.4809887
A M	0.0548346	0.3609403	-0.4453897	0.2988508	0.5002243	0.4686132	0.286	0.4699636
A N	-0.5459869	0.5777401	-0.8401922	0.496787	0.2942054	0.7619587	0.699	0.4792416
A P	-0.4179627	0.5716158	0.0923597	0.3666249	-0.5103224	0.6790865	0.452	0.4736406
B C	-0.5815219	0.645387	-0.0717042	0.3427487	-0.5098177	0.7307537	0.485	0.4742472
B F	-0.3760667	0.5152398	0.0038979	0.3789481	-0.3799646	0.6401908	0.553	0.4779912
B G	-1.037162	0.3632355	-1.43583	0.3892946	0.3986678	0.5312489	0.453	0.4760746
B H	-0.064234	0.2974923	-0.4716911	0.3940257	0.4074571	0.4936825	0.409	0.4766075
B I	0.023751	0.590255	0.1168085	0.4504487	-0.0930575	0.7424992	0.900	0.4799331
B J	-0.7604885	0.3193596	-0.1923802	0.2904951	-0.5681083	0.4318811	0.188	0.4726315
B K	-0.3136883	0.5552685	-0.4328527	0.2927825	0.1191644	0.6277299	0.849	0.4807849
B L	-0.3599981	0.3831835	0.0049272	452.6905	-0.3649252	452.6907	0.999	0.4697358
B M	-0.2641326	0.2951732	-0.223504	0.3329853	-0.0406287	0.444907	0.927	0.4812289
B N	-0.5372583	0.5518664	-0.8686697	0.5041027	0.3314114	0.7474464	0.657	0.4790435
B O	-0.373402	0.3985252	0.0041624	453.037	-0.3775644	453.0371	0.999	0.4697358
B P	0.0067436	0.3399792	-0.2045961	0.5036485	0.2113397	0.6076519	0.728	0.478236
C E	-0.3932297	0.6335684	0.5210328	0.6525707	-.9142625	0.9054262	0.313	0.4710477
C F	0.2566499	0.6242505	-0.0485434	0.4453395	0.3051933	0.7668497	0.691	0.4777596
D J	0.3475354	0.6066204	-0.5194874	0.6806421	0.8670229	0.9111296	0.341	0.472892
D M	0.3897667	0.5969657	-0.1186772	0.684615	0.5084438	0.9030627	0.573	0.477009
D N	-0.7538736	0.6347555	0.2038414	0.67107	-0.9577151	0.923715	0.300	0.4706312
E F	0.6785635	0.6349095	-0.6743338	0.6349873	1.352897	0.8952597	0.131	0.4603151
E M	-0.9525685	0.6143637	0.6494272	0.5829833	-1.601996	0.8496062	0.059	0.4492131
E Q	-0.0221164	0.63479	0.7238256	0.8651093	-0.7459421	1.072586	0.487	0.4756612
F J	0.1565691	0.5105287	-0.6418802	0.418972	0.7984492	0.6613809	0.227	0.4688523
F M	-0.218787	0.5161632	-0.0518867	0.4117684	-0.1669004	0.6605774	0.801	0.4804247
H J	-0.2667128	0.5167824	-0.2297135	0.3428007	-0.0369993	0.6200116	0.952	0.4823375
H K	-0.5066013	0.5098171	-0.0223149	0.3783681	-0.4842865	0.634795	0.446	0.4751468
J K	0.0455167	0.3227253	0.0493795	0.4124058	-0.0038628	0.523531	0.994	0.4811065
J M	0.0614462	0.5974452	0.2454279	0.3038233	-0.1839816	0.6702738	0.784	0.4795692
M Q	0.9596908	0.6446798	-0.1978456	0.6104481	1.157536	0.8876927	0.192	0.4653671
P Q	0.0102025	0.544999	0.4141067	0.6927936	-0.4039041	0.8814686	0.647	0.4775536

Notes: P>0.05 indicates that indirect comparisons were consistent with direct comparisons.

Appendix 35. Subgroup analysis of node-splitting method in comparison between direct and indirect evidence of specific treatments for anxiety

Comparison	Direct Effect		Indirect Effect		Difference		P value	tau
	Estimate	Standard error	Estimate	Standard error	Estimate	Standard error		
A C	-0.1224938	0.4320651	-0.280588	0.4381323	0.1580942	0.6163628	0.798	0.3847667
A D	-1.35642	0.3189225	-1.070323	0.6081138	-0.2860963	0.6867321	0.677	0.3737378
A E	-0.736011	0.4519664	-0.0776149	0.3089136	-0.6583961	0.5474497	0.229	0.3439325
A F	0.1299983	0.5003774	0.0070849	0.5668196	0.1229134	0.7560833	0.871	0.3767443
A G	-0.0795166	0.429154	-0.586788	0.3312875	0.5072713	0.5421307	0.349	0.3708672
A H	-0.273675	0.4476622	-0.3997602	0.3522271	0.1260852	0.5696186	0.825	0.3827963
A J	-0.203502	0.4281667	-0.3573732	0.3461043	0.1538712	0.5505587	0.780	0.3822187
B C	-0.4486656	0.4306896	-0.4013693	0.398868	-0.0472963	0.588028	0.936	0.3851077
B D	-1.329564	0.5549841	-1.615659	0.404467	0.2860953	0.6867321	0.677	0.3737375
B E	-0.4659063	0.3214927	-0.5731048	0.3657198	0.1071985	0.4884363	0.826	0.3861141
B F	-0.2019456	0.5105776	-0.0790327	0.5576506	-0.1229129	0.7560844	0.871	0.3767452
B G	-1.042815	0.3623172	-0.2929674	0.3041754	-0.749848	0.4768134	0.116	0.370314
B H	4.82e-09	0.4302436	-0.8156992	0.2880296	0.8156992	0.5177554	0.115	0.3300911
B I	-0.4219795	0.3159586	-0.4498736	438.4633	0.0278941	438.4634	1.000	0.3547302
B J	-0.4713069	0.2647804	-0.6514341	0.4431331	0.1801272	0.5169522	0.728	0.3834386
B K	0.1881408	0.5652564	1.097152	0.789656	-0.9090115	0.9711186	0.349	0.3570957
C G	0.0333105	0.4275759	-0.4653245	0.4548861	0.498635	0.6256702	0.425	0.3769553
C J	-0.2770016	0.4204469	0.1096346	0.4503956	-0.3866362	0.6166988	0.531	0.3755956
E G	-0.1940303	0.422491	-0.0572033	0.3643732	-0.136827	0.5577467	0.806	0.3797101
E H	-0.377868	0.3858067	0.2228328	0.3666633	-0.6007008	0.532197	0.259	0.3391534
G H	-0.2039824	0.3309461	0.4390628	0.4193957	-0.6430451	0.534818	0.229	0.3681215
J L	1.520689	0.6146844	0.6116661	0.7518215	0.9090232	0.9711192	0.349	0.3570958
K L	-0.0466125	0.4432879	0.8624075	0.8640447	-0.90902	0.9711217	0.349	0.3570969

Notes: P>0.05 indicates that indirect comparisons were consistent with direct comparisons.

Appendix 36. Subgroup analysis of node-splitting method in comparison between direct and indirect evidence of specific treatments for mental health

Comparison	Direct Effect		Indirect Effect		Difference		P value	tau
	Estimate	Standard error	Estimate	Standard error	Estimate	Standard error		
A C	1.030816	0.4284866	0.733404	0.8691058	0.2974119	0.9690528	0.759	0.6413474
A D	0.5650587	0.7038773	0.9168649	0.6960842	-0.3518062	0.9899376	0.722	0.6405067
A E	0.6098802	0.5026667	0.5649154	0.919646	0.0449648	1.047919	0.966	0.6456217
B C	-0.0398366	0.6804095	0.8038627	0.6353822	-0.8436993	0.9317061	0.365	0.6160116
B D	0.2370128	0.3881662	-0.1148047	0.9106589	0.3518174	0.9899402	0.722	0.6405068
B E	0.0178501	0.7313605	0.0628118	0.7504985	-0.0449617	1.04792	0.966	0.6456215
B F	0.4209494	0.4692919	-1.120004	446.8165	1.540954	446.8168	0.997	0.6063533
B G	0.6275435	0.3571406	1.222298	1.904698	-0.5947543	1.938101	0.759	0.641347
C G	0.0189256	0.7023984	0.5177156	0.804975	-0.49879	1.068622	0.641	0.6416349

Notes: P>0.05 indicates that indirect comparisons were consistent with direct comparisons.

Appendix 37. Evaluation of inconsistency using loop-specific heterogeneity estimates of different categories of interventions for depression

Loop	IF	seIF	Z value	P value	95%CI	tau2
CO-MUI-PT	1.071	0.515	2.080	0.038	(0.06,2.08)	0.039
EX-MUI-PT	0.892	0.947	0.942	0.346	(0.00,2.75)	0.305
EX-BA-MUI	0.600	0.434	1.381	0.167	(0.00,1.45)	<0.001
CO-EX-BA	0.474	0.354	1.338	0.181	(0.00,1.17)	0.057
CO-EX-MUI	0.237	0.263	0.901	0.368	(0.00,0.75)	0.024
CO-EDU-BA	0.225	0.590	0.382	0.703	(0.00,1.38)	0.131
CO-BA-MUI	0.217	0.629	0.344	0.731	(0.00,1.45)	0.095
CO-EX-PT	0.036	0.255	0.140	0.889	(0.00,0.54)	0.030

Notes: P>0.05 indicates that no inconsistency in the closed loop.

Appendix 38. Evaluation of inconsistency using loop-specific heterogeneity estimates of different categories of interventions for anxiety

Loop	IF	seIF	Z value	P value	95%CI	tau2
EX-MUI-PT	1.861	0.658	2.829	0.005	(0.57,3.15)	0.000
CO-EX-PT	0.916	0.582	1.574	0.116	(0.00,2.06)	0.073
CO-MUI-PT	0.572	0.533	1.073	0.283	(0.00,1.62)	0.002
CO-EX-MUI	0.433	0.267	1.622	0.105	(0.00,0.96)	0.003
CO-EDU-BA	0.369	0.780	0.473	0.636	(0.00,1.90)	0.209
CO-EX-BA	0.294	0.900	0.326	0.744	(0.00,2.06)	0.217

Notes: p>0.05 indicates that no inconsistency in the closed loop.

Appendix 39. Evaluation of inconsistency using loop-specific heterogeneity estimates of different categories of interventions for mental health

Loop	IF	seIF	Z value	P value	95%CI	tau2
CO-EX-MUI	0.722	0.711	1.016	0.310	(0.00,2.12)	0.307
CO-EX-PT	0.483	0.803	0.601	0.548	(0.00,2.06)	0.266

Notes: p>0.05 indicates that no inconsistency in the closed loop.

Appendix 40. Subgroup analysis of evaluation of inconsistency using loop-specific heterogeneity estimates of specific treatments for depression

Loop	IF	seIF	Z value	P value	95%CI	tau2
B-C-E-M	1.706	0.870	1.961	0.050	(0.00,3.41)	0.039
A-C-E-M	1.539	0.641	2.401	0.016	(0.28,2.80)	0.000
A-M-P-Q	1.413	0.625	2.263	0.024	(0.19,2.64)	0.000
A-B-C-H	1.399	0.570	2.454	0.014	(0.28,2.52)	0.000
B-D-J-N	1.385	1.936	0.715	0.474	(0.00,5.18)	0.375
A-H-J	1.377	0.415	3.317	0.001	(0.56,2.19)	0.000
A-B-G-J	1.333	1.589	0.839	0.401	(0.00,4.45)	0.837
A-B-F-H	1.244	0.417	2.983	0.003	(0.43,2.06)	0.000
A-B-H-M	1.238	0.406	3.050	0.002	(0.44,2.03)	0.014
A-H-K	1.091	0.406	2.688	0.007	(0.30,1.89)	0.000
A-B-H-I	0.999	0.720	1.388	0.165	(0.00,2.41)	0.047
A-B-C-P	0.978	0.603	1.622	0.105	(0.00,2.16)	0.000
A-B-J-N	0.920	1.832	0.502	0.616	(0.00,4.51)	0.375
A-B-C-G	0.815	1.400	0.582	0.561	(0.00,3.56)	0.822
A-B-I-J	0.811	1.532	0.529	0.597	(0.00,3.81)	0.371
A-B-F-P	0.769	0.666	1.154	0.249	(0.00,2.08)	0.069
A-B-F-G	0.663	1.896	0.350	0.726	(0.00,4.38)	1.285
A-B-G-M	0.663	1.075	0.616	0.538	(0.00,2.77)	0.383
B-M-P-Q	0.641	0.673	0.953	0.341	(0.00,1.96)	0.045
A-B-G-H	0.608	1.350	0.450	0.653	(0.00,3.25)	0.478
B-F-J	0.604	1.480	0.408	0.683	(0.00,3.50)	0.375
A-B-C-K	0.583	0.586	0.995	0.320	(0.00,1.73)	0.000
A-D-M-N	0.562	0.659	0.853	0.394	(0.00,1.85)	0.000
H-J-K	0.531	0.341	1.558	0.119	(0.00,1.20)	0.000
A-J-K	0.523	0.447	1.169	0.242	(0.00,1.40)	0.048
A-B-C-N	0.472	0.637	0.740	0.459	(0.00,1.72)	0.000
A-B-G-I	0.468	1.613	0.290	0.772	(0.00,3.63)	1.072
A-D-J-N	0.465	0.690	0.674	0.500	(0.00,1.82)	0.000
A-B-H-P	0.442	0.517	0.854	0.393	(0.00,1.45)	0.009
A-B-F-K	0.427	0.438	0.975	0.330	(0.00,1.29)	0.000
B-J-K	0.412	0.943	0.437	0.662	(0.00,2.26)	0.245
A-B-C-I	0.406	0.776	0.523	0.601	(0.00,1.93)	0.075
A-B-K-M	0.400	0.447	0.894	0.371	(0.00,1.28)	0.022
A-B-C-J	0.384	1.189	0.323	0.746	(0.00,2.71)	0.215
A-F-M	0.338	0.307	1.101	0.271	(0.00,0.94)	0.000
A-B-F-N	0.316	0.505	0.626	0.531	(0.00,1.31)	0.000
A-B-F-I	0.305	0.486	0.628	0.530	(0.00,1.26)	0.000
A-B-G-K	0.300	1.547	0.194	0.846	(0.00,3.33)	0.993

A-B-I-M	0.256	0.647	0.395	0.693	(0.00,1.52)	0.063
A-C-F	0.219	0.485	0.451	0.652	(0.00,1.17)	0.000
A-B-C-M	0.179	0.525	0.342	0.733	(0.00,1.21)	0.009
A-J-M	0.097	0.440	0.221	0.825	(0.00,0.96)	0.000
B-C-F	0.063	0.625	0.101	0.919	(0.00,1.29)	0.000
A-B-G-P	0.046	1.423	0.032	0.974	(0.00,2.83)	0.895

Notes: p>0.05 indicates that no inconsistency in the closed loop.

Appendix 41. Subgroup analysis of evaluation of inconsistency using loop-specific heterogeneity estimates of specific treatments for anxiety

Loop	IF	seIF	Z value	P value	95%CI	tau2
B-G-H	1.517	1.365	1.111	0.266	(0.00,4.19)	0.724
A-B-D-G	1.239	1.727	0.718	0.473	(0.00,4.62)	0.605
B-J-K-L	0.876	0.748	1.171	0.242	(0.00,2.34)	0.009
A-B-G-J	0.863	1.280	0.675	0.500	(0.00,3.37)	0.330
A-E-G	0.852	0.409	2.086	0.037	(0.05,1.65)	0.000
A-E-H	0.840	0.417	2.015	0.044	(0.02,1.66)	0.000
B-E-G	0.665	1.452	0.458	0.647	(0.00,3.51)	0.719
A-B-C-H	0.608	0.454	1.340	0.180	(0.00,1.50)	0.000
A-B-H-J	0.573	0.465	1.233	0.218	(0.00,1.48)	0.009
A-B-E-J	0.533	0.663	0.804	0.422	(0.00,1.83)	0.094
A-B-C-E	0.533	0.435	1.225	0.221	(0.00,1.39)	0.000
A-B-E-F	0.530	0.584	0.908	0.364	(0.00,1.67)	0.000
A-B-C-D	0.347	0.525	0.661	0.509	(0.00,1.38)	0.000
A-B-D-F	0.344	0.654	0.527	0.598	(0.00,1.63)	0.000
A-B-D-J	0.309	0.565	0.547	0.584	(0.00,1.42)	0.022
A-B-D-H	0.261	0.574	0.455	0.649	(0.00,1.39)	0.000
A-B-F-G	0.253	0.549	0.460	0.646	(0.00,1.33)	0.000
B-C-G	0.250	0.326	0.768	0.442	(0.00,0.89)	0.000
A-B-D-E	0.244	0.891	0.274	0.784	(0.00,1.99)	0.160
A-C-J	0.191	0.334	0.573	0.567	(0.00,0.85)	0.000
A-B-F-J	0.033	0.560	0.058	0.953	(0.00,1.13)	0.009
A-G-H	0.003	0.358	0.009	0.993	(0.00,0.70)	0.000
A-B-C-F	0.003	0.551	0.005	0.996	(0.00,1.08)	0.000

Notes: p>0.05 indicates that no inconsistency in the closed loop.

Appendix 42. Subgroup analysis of evaluation of inconsistency using loop-specific heterogeneity estimates of specific treatments for mental health

Loop	IF	seIF	Z value	P value	95%CI	tau2
A-B-C-D	0.749	1.102	0.679	0.497	(0.00,2.91)	0.250
B-C-G	0.525	0.423	1.242	0.214	(0.00,1.35)	0.000
A-B-C-E	0.491	1.714	0.287	0.774	(0.00,3.85)	0.934
A-B-D-E	0.270	1.354	0.199	0.842	(0.00,2.92)	0.344

Notes: p>0.05 indicates that no inconsistency in the closed loop.

Appendix 43. Egger's test for the assessment of publication bias of pairwise meta-analysis

Category	Comparison	Number of RCTs	t value	P value
Depression	EX vs CO	7	-0.40	0.707
Depression	BA vs CO	9	-1.30	0.235
Depression	PT vs CO	16	-0.52	0.611
Anxiety	BA vs CO	7	-1.39	0.223
Mental health	PT vs CO	8	-0.17	0.868

Notes: p>0.05 indicates that no publication bias of pairwise meta-analysis.

Appendix 44. Network meta-analysis funnel plots of different categories of interventions for the assessment of publication bias for depression

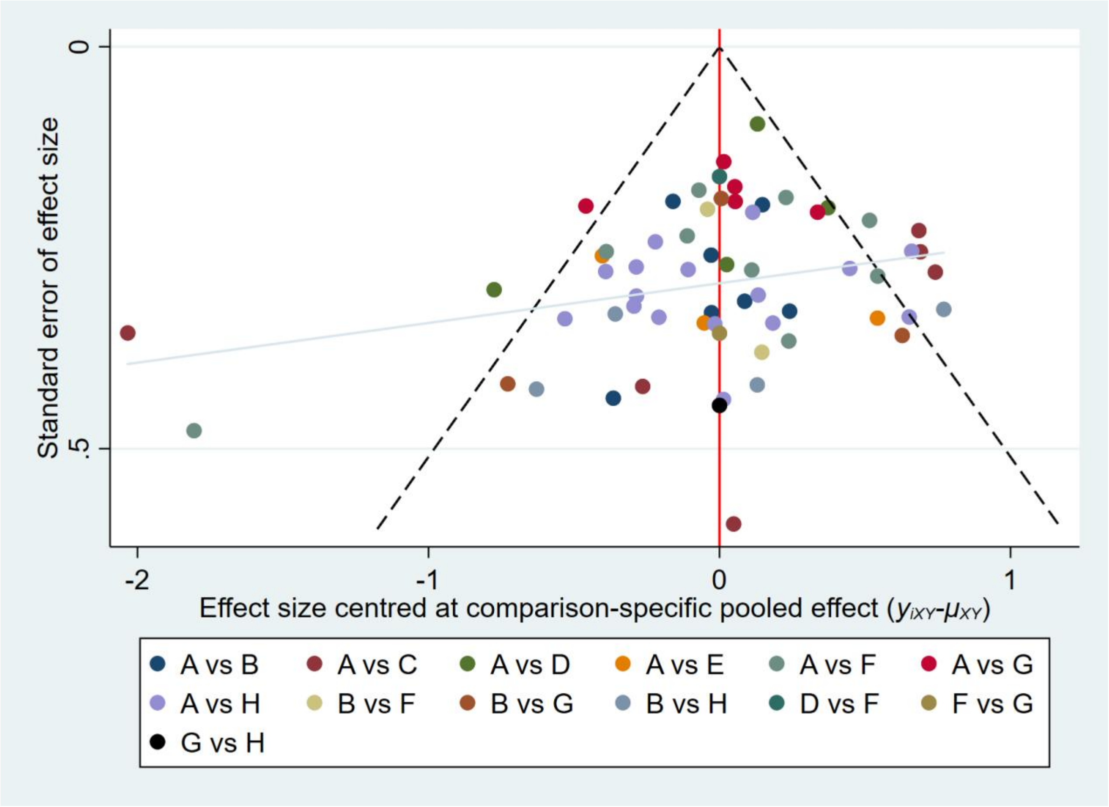

Appendix 45. Network meta-analysis funnel plots of different categories of interventions for the assessment of publication bias for anxiety

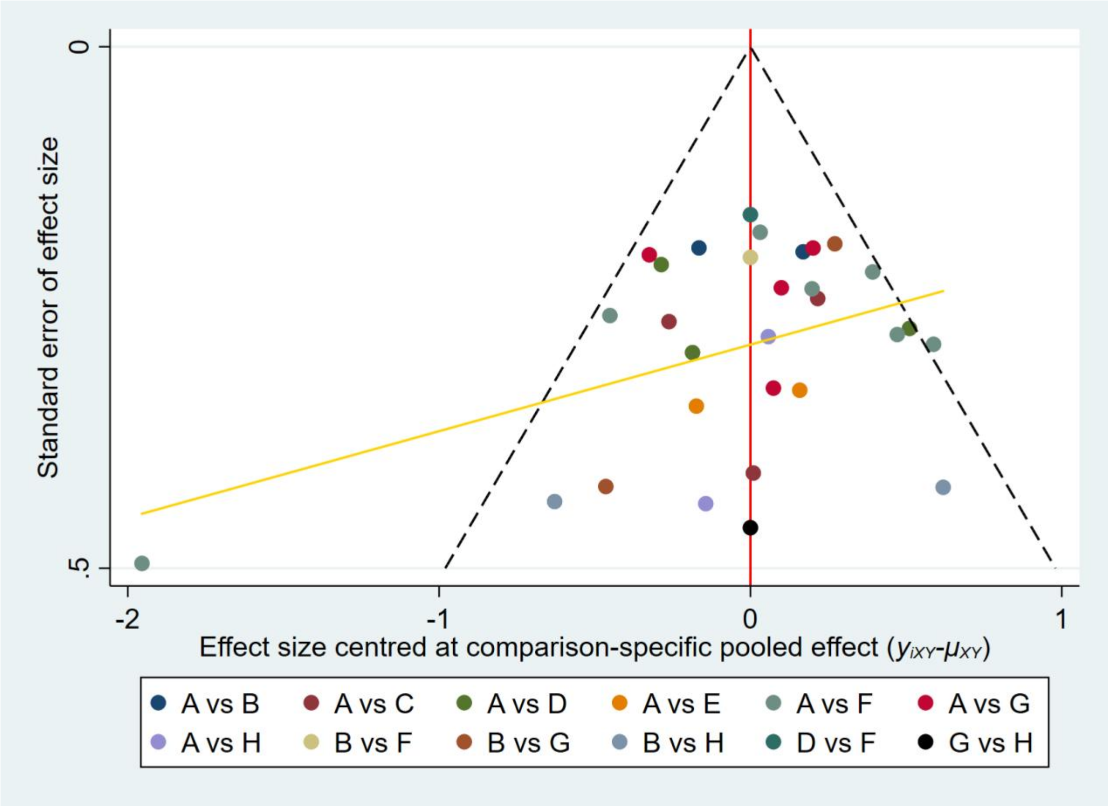

Appendix 46. Network meta-analysis funnel plots of different categories of interventions for the assessment of publication bias for mental health

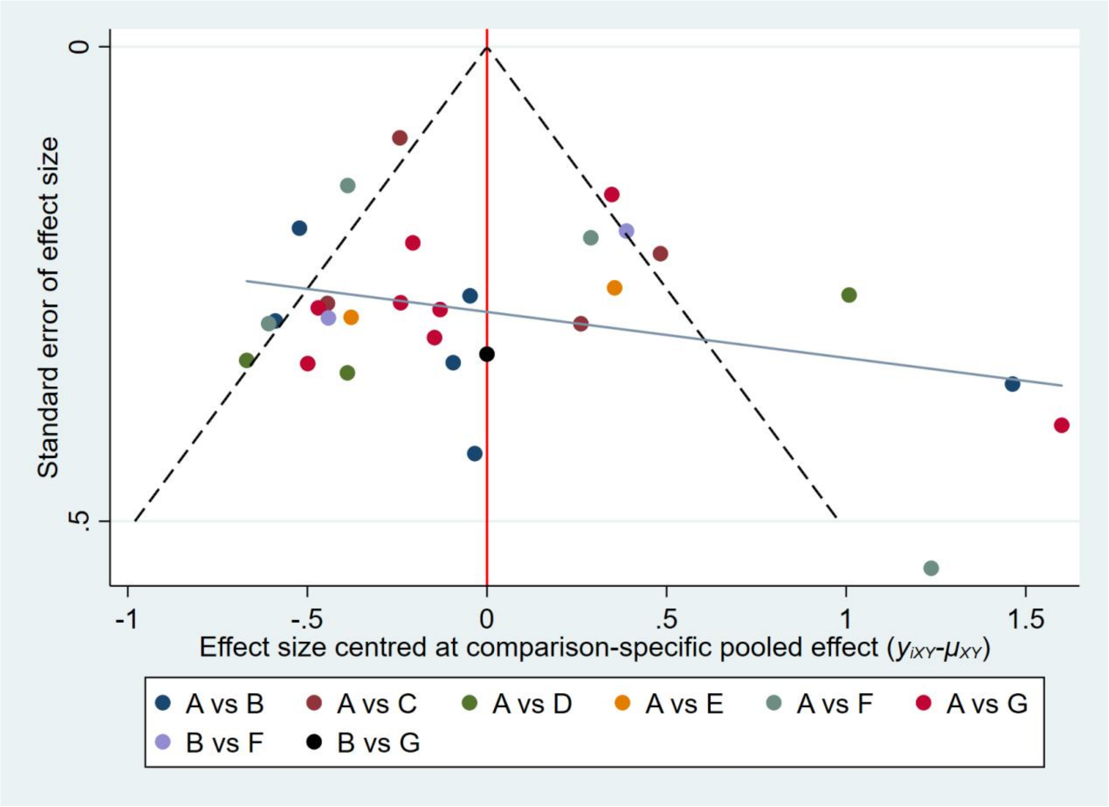

Appendix 47. Subgroup analysis of meta-analysis funnel plots of specific treatments for the assessment of publication bias for depression

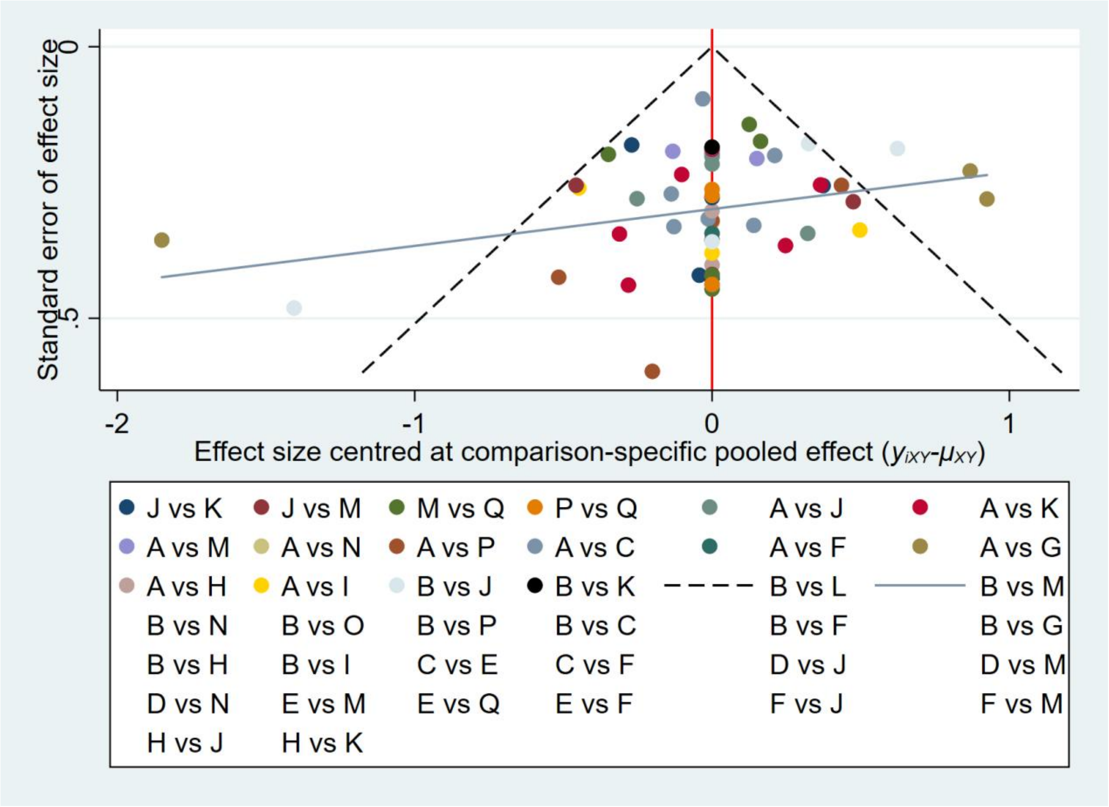

Appendix 48. Subgroup analysis of network meta-analysis funnel plots of specific treatments for the assessment of publication bias for anxiety

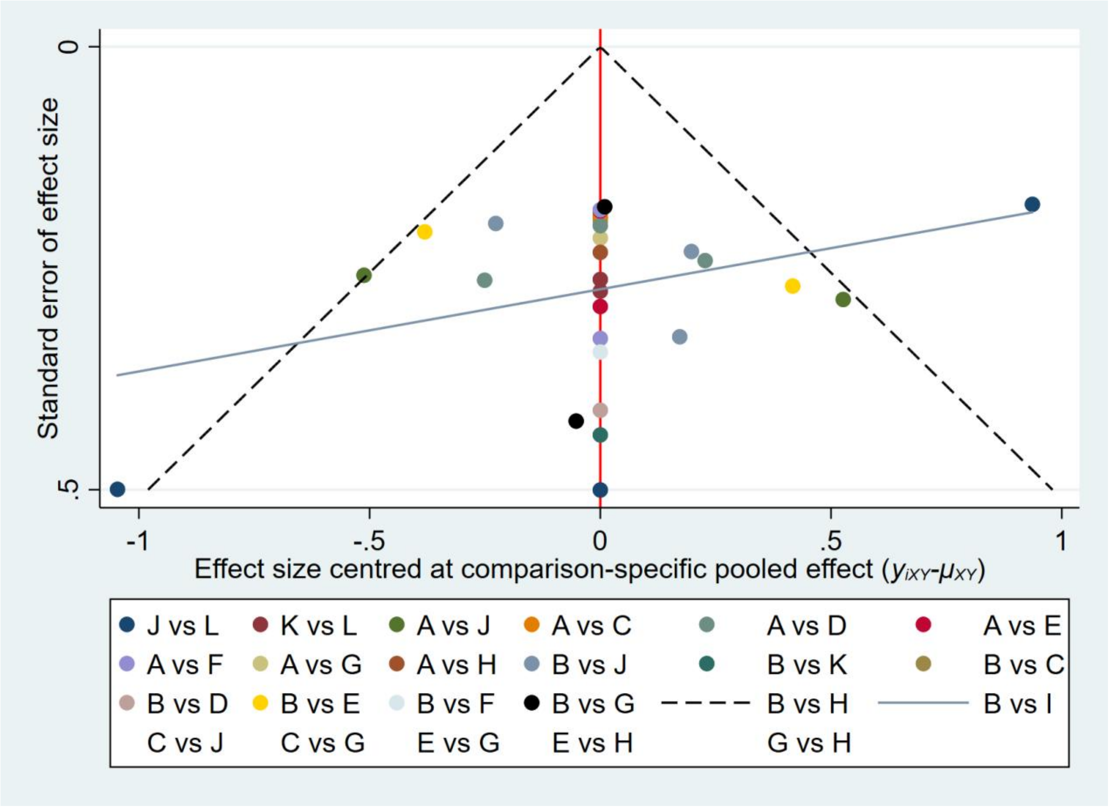

Appendix 49. Subgroup analysis of network meta-analysis funnel plots of specific treatments for the assessment of publication bias for mental health

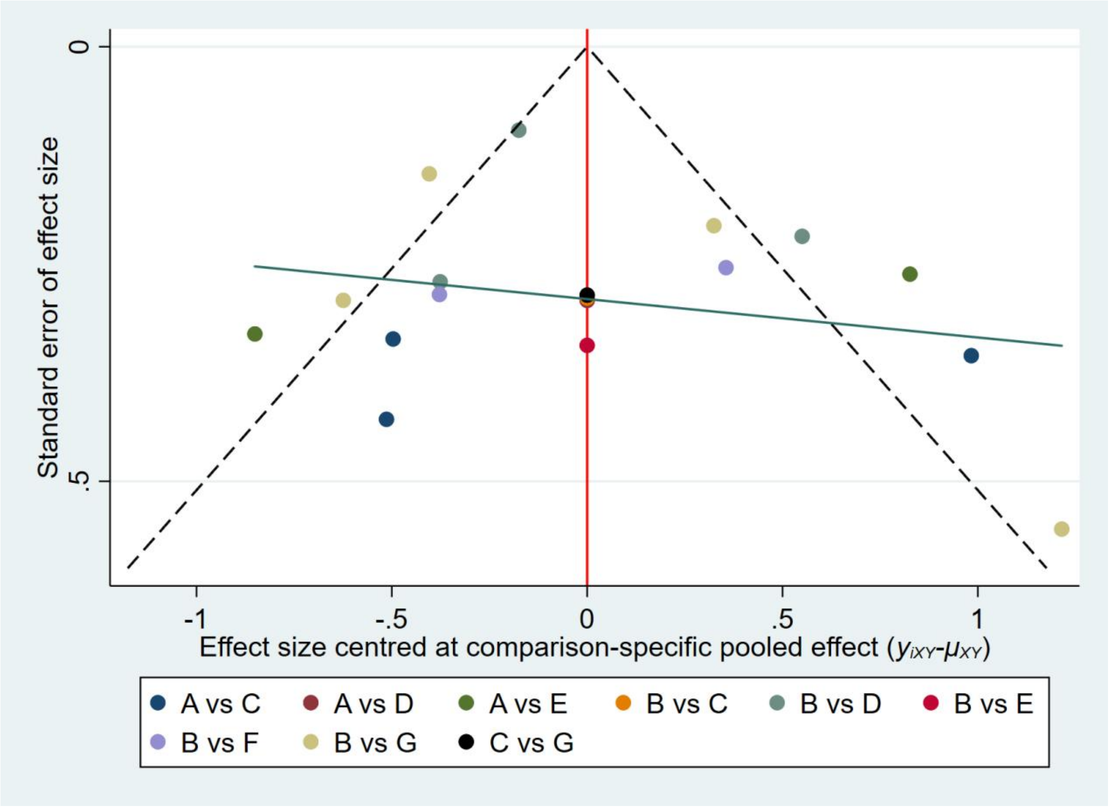

Supplement: SUPPLEMENTARY MATERIAL [file js9-110-478-s005.pdf]
